# Humans vs. Fungi: An Overview of Fungal Pathogens against Humans

**DOI:** 10.3390/pathogens13050426

**Published:** 2024-05-17

**Authors:** Kasun M. Thambugala, Dinushani A. Daranagama, Danushka S. Tennakoon, Dona Pamoda W. Jayatunga, Sinang Hongsanan, Ning Xie

**Affiliations:** 1 Genetics and Molecular Biology Unit, Faculty of Applied Sciences, University of Sri Jayewardenepura, Gangodawila, Nugegoda 10250, Sri Lanka; kasun@sci.sjp.ac.lk (K.M.T.); pamoda@sci.sjp.ac.lk (D.P.W.J.); 2 Center for Biotechnology, Department of Zoology, University of Sri Jayewardenepura, Nugegoda 10250, Sri Lanka; 3 Center for Plant Materials and Herbal Products Research, University of Sri Jayewardenepura, Nugegoda 10250, Sri Lanka; 4 Department of Plant and Molecular Biology, Faculty of Science, University of Kelaniya, Kelaniya 11300, Sri Lanka; anupamad@kln.ac.lk; 5 Bioengineering and Technological Research Centre for Edible and Medicinal Fungi, Jiangxi Agricultural University, Nanchang 330045, China; danushkasandaruwanatm@gmail.com; 6 Shenzhen Key Laboratory of Microbial Genetic Engineering, College of Life Science and Oceanography, Shenzhen University, Shenzhen 518060, China

**Keywords:** ascomycota, human pathogens, medical mycology, phylogeny, taxonomy

## Abstract

Human fungal diseases are infections caused by any fungus that invades human tissues, causing superficial, subcutaneous, or systemic diseases. Fungal infections that enter various human tissues and organs pose a significant threat to millions of individuals with weakened immune systems globally. Over recent decades, the reported cases of invasive fungal infections have increased substantially and research progress in this field has also been rapidly boosted. This review provides a comprehensive list of human fungal pathogens extracted from over 850 recent case reports, and a summary of the relevant disease conditions and their origins. Details of 281 human fungal pathogens belonging to 12 classes and 104 genera in the divisions ascomycota, basidiomycota, entomophthoromycota, and mucoromycota are listed. Among these, *Aspergillus* stands out as the genus with the greatest potential of infecting humans, comprising 16 species known to infect humans. Additionally, three other genera, *Curvularia*, *Exophiala*, and *Trichophyton*, are recognized as significant genera, each comprising 10 or more known human pathogenic species. A phylogenetic analysis based on partial sequences of the 28S nrRNA gene (LSU) of human fungal pathogens was performed to show their phylogenetic relationships and clarify their taxonomies. In addition, this review summarizes the recent advancements in fungal disease diagnosis and therapeutics.

## 1. Introduction

Human fungal pathogens are considered the “hidden killers”, as they can cause numerous infections and create an unprecedented burden on human health [1,2,3,4]. Due to the increasing number of infections and deaths each year, they are considered a rising threat to human health [3,5,6]. Fungi are responsible for 1.5 million annual fatalities and have infected one-third of the human population [3,4,7,8,9]. These accounts exceed the death rates from severe diseases, such as malaria, tuberculosis, and breast cancer [7]. The rapid rise in fungal infections is frequently linked to climate change, the virulence of the pathogens, and the growing number of immunocompromised patients worldwide [1]. However, only a few hundred (nearly 300 species) of the estimated 3.5–5.1 million fungal species are linked to human fungal diseases [10,11]. Of them, species belonging to four genera, including *Aspergillus*, *Candida*, *Cryptococcus*, and *Pneumocystis*, cause the preponderance of fatalities [7,8].

In the battle against human fungal diseases, antifungal agents have become necessary tools, incorporating chemical compounds and natural products in the management and prevention of human fungal diseases [12,13]. Apart from antifungal agents, various methods, strategies, and approaches are currently used in the management of human fungal diseases. Some of these are immunomodulatory therapies, like cytokine therapy and immune checkpoint inhibitors, which are meant to boost the body’s defenses against fungal infections [14]. Additionally, combination therapies involving multiple antifungal agents or the use of alternative treatment options like photodynamic therapy have shown promising results in managing human fungal diseases [12,14,15].

As we delve into the multifaceted realm of human fungal pathogens, it becomes evident that understanding their impact on public health and the strategies employed for their management is of paramount importance. This review aims to provide comprehensive insights into the world of human fungal diseases, shedding light on the latest advancements and challenges in this critical field.

## 2. The Sources and Development of Fungal Infections

**Figure 1:**
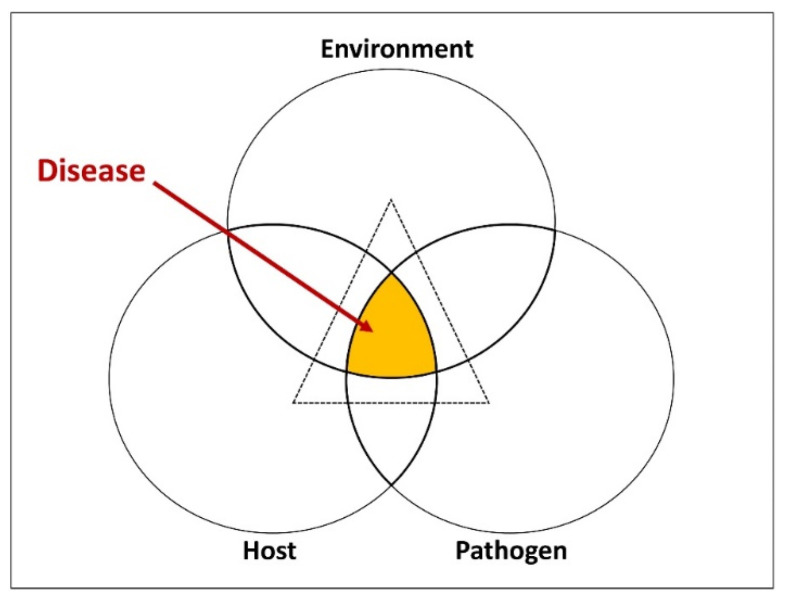
Fungal pathogenesis represented in the form of the disease cycle [19].

**Figure 2:**
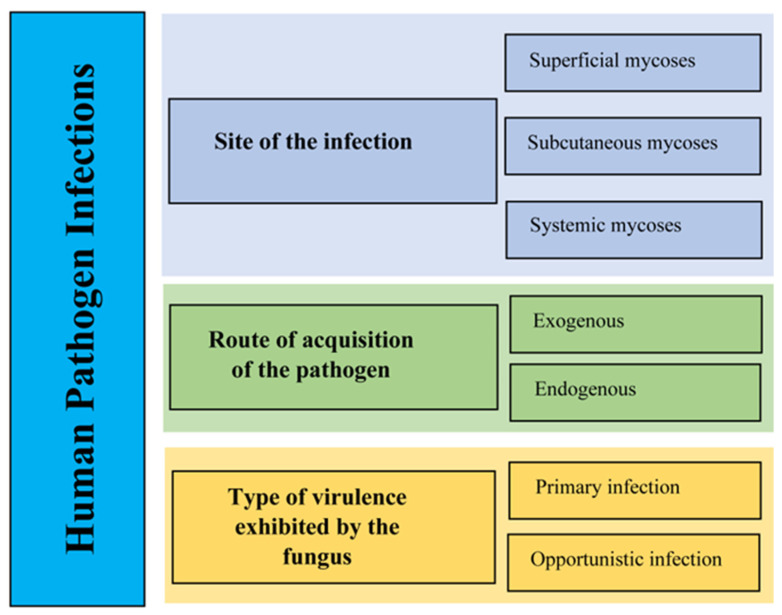
The classification of human pathogen infections [20].

**Figure 3:**
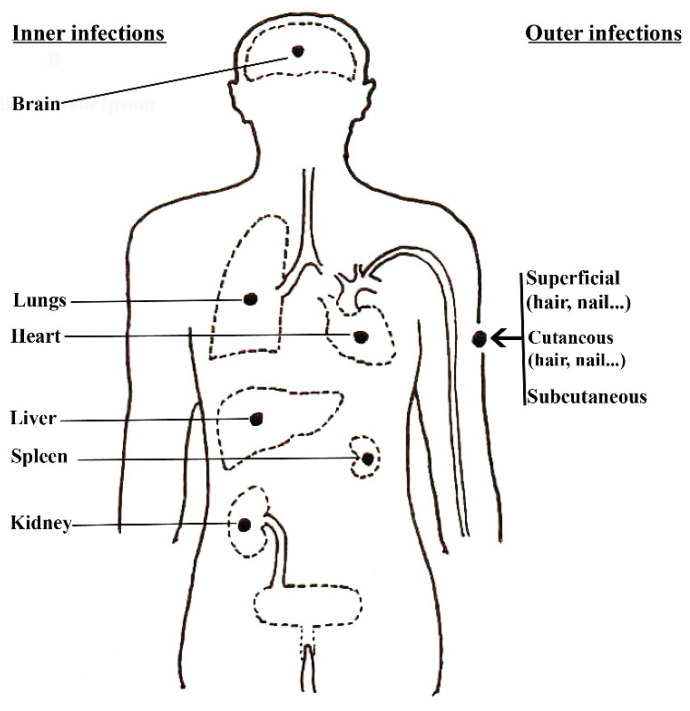
The infection sites of the human body (adapted from [20]).

Fungal pathogens of humans can produce mycotoxins, which can lead to mycotoxicoses and cause allergic reactions. These mycotoxicoses and allergens have the potential to instigate severe diseases commonly referred to as mycoses, particularly in those with weakened immune systems or other health issues [16,17]. Mycoses primarily rely on the intricate interactions between pathogens, susceptible human hosts, and their favorable environments (Figure 1) [4,18]. Over 300 million individuals suffer from fatal diseases because of fungal spores that are airborne, soil-borne, and transmitted through people [14,15]. The development of mycoses can be attributed to several factors, including the entering of pathogens into host tissues, immune system weaknesses, and/or other conditions that facilitate fungal entry and persistence [10,11]. The classification of human pathogen infections can take various approaches, primarily depending on the type of virulence exhibited by the fungus (Figure 2), the pathogen acquisition route, and the site of the infection (Figure 3). These classification methods help in understanding the diverse nature of fungal infections in humans and contribute to diagnostic and treatment strategies.

### 2.1. Site of the Infection

#### 2.1.1. Superficial/Cutaneous Mycoses

**Figure 4:**
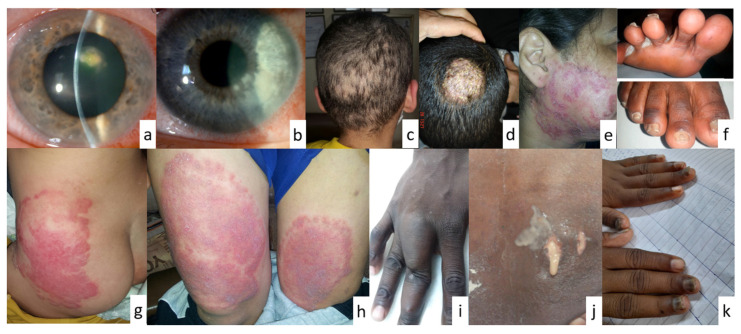
Clinical images showing fungal infections in different sites of the human body. (**a**,**b**). keratitis, (**c**). moth-eaten alopecia, (**d**). tinea capitis, (**e**,**h**). psoriasis-like tinea, (**f**). tinea pedis, (**g**). tinea capitis, (**i**). mycetoma, (**j**). blastomycosis (purulent discharge from a cutaneous sinus tract at the T5 vertebral level), (**k**). nail infection (images courtesy of Khalifa E. Sharquie, Sarah A. Ahmed and Mawahib A.I. Ismail, Darren Ting, and Khadim Diongue; images (**a**,**b**,**f**,**j**) were reproduced with permission from Diongue et al. [58], Maphanga et al. [59], Ting et al. [60]).

The human body hosts a variety of environmental microbes and their infections, some of which are commensal, posing no harmful or no disease-causing potential, while others have the potential to cause infections in various parts of the human body [1,21] (Figure 4). However, the keratinized epithelia of the human body serve as a natural barrier for microbes and assist the body in defending itself against many fungal infections [16]. This layer prevents the invasion of microbes to deeper tissues, which can cause more severe diseases. As well, the skin secretes various substances (e.g., sweat, sebum, transferrin, antimicrobial peptides) which can prevent the growth of microbes [17]. Nevertheless, some fungal species possess the capability of breaking through the defensive mechanisms of the human body, colonizing the surface skins, and causing infections. The infections of the skin, hair, and nails that are confined to the keratinized layers are considered superficial or cutaneous mycoses [1]. Dermatophytosis fungal illnesses, often known as tinea infections, are the most prevalent type of infection with high recurrence and can affect the entire body. They can be classified into three distinct genera, specifically *Trichophyton*, *Microsporum*, and *Epidermophyton* [22]. They can be spread from human to human (anthropophilic), animal to human (zoophilic), or soil to human (geophilic) [23]. These fungi thrive in warm and moist environments, making them more prevalent in tropical and subtropical regions. In addition, black piedra and white piedra also have fungal infections of the hair shafts caused by *Piedraia hortae* and *Trichosporon beigelii*, respectively [24,25]. Symptoms of these diseases can vary in appearance, including inflammation, swelling, and vesicles. The nails may be brittle, raised, discolored, and thicker if they have a fungus infection [26,27]. Some of the superficial mycoses and their causal fungal species are listed in Table 1.

#### 2.1.2. Subcutaneous Mycoses

Subcutaneous mycoses typically occur when the fungus is being implanted through a cut or lesions on the skin [33]. Most often, barefoot workers, including farmers, gardeners, and children, are susceptible to these infections. The common subcutaneous infections are chromoblastomycosis, hyalohyphomycosis, phaeohyphomycosis, mycetoma [28,29,33], and sporotrichosis [34,35]. The symptoms of subcutaneous infections can vary with the disease, but they typically present as fistulae, localized nodules, granulomatous tissue, subcutaneous masses with abscesses, and ulcerations [36]. Some of the subcutaneous mycoses and their causal fungal species are listed in Table 2. The most common subcutaneous mycoses are chromoblastomycosis, entomophthoromycosis, mycetoma, phaeohyphomycosis and sporotrichosis.

#### 2.1.3. Systemic Mycoses

The fungal infections known as systemic mycoses can affect internal organs, including the lungs and brain, subsequently affecting the whole body [40]. Mainly, these infections occur by inhaling spores or hyphae and are disseminated via the bloodstream to multiple organs [37,41]. The severity of the infection depends on the clinical status of the patient, and fever, cough, and loss of appetite are the common symptoms. There are two types of systemic mycosis, including endemic respiratory infections and opportunistic infections [20]. Although endemic respiratory infections affect both immunocompetent and immunocompromised hosts, immunocompromised patients are more at risk of opportunistic infections. Some of the most common systemic mycoses are aspergillosis, blastomycosis, coccidiodomycosis, histoplasmosis, and paracoccidiodomycosis (Table 2) [42,43,44,45,46].

#### 2.1.4. Opportunistic Mycoses

Opportunistic mycoses are fungal infections that typically do not cause disease in healthy persons but can lead to illness in people with weakened immune systems [47]. However, the virulence and pathogenicity of these fungi are explained by their ability to survive and reproduce in conditions that are not favorable for their growth. The human immune system recognizes and defends itself against various infections, but immunocompromised individuals are more vulnerable to severe diseases. There are many risk factors for opportunistic fungal infections [48], such as HIV infections, anticancer chemotherapy, solid-organ transplantation, granulocytopenia, old age, premature birth, the use of broad-spectrum antibiotics, gastro-intestinal surgery, and central vascular catheters [49,50]. In addition, some chronic diseases and other debilitating situations afford suitable environmental conditions for the metabolism of fungi, such as malignant tumors, tuberculosis, amebic abscess of the liver, and surgical procedures. Some of the main opportunistic mycoses are aspergillosis, candidiasis, cryptococcosis, fusariosis, hyalohyphomycosis, mucormycosis, penicilliosis, phaeohyphomycosis, pneumocystosis, scedosporiosis, and zygomycosis (Table 2).

Mycoses, as defined and explained above, affect humans, and present diverse clinical manifestations essential for accurate diagnosis and effective treatment. Understanding these aspects is crucial in clinical practice [51]. Manifestations vary based on the causative agent and infection site [52] (Figure 4). Superficial mycoses show localized skin lesions (e.g., tinea pedis). Cutaneous mycoses present as inflammatory or non-inflammatory lesions (e.g., candidiasis). Subcutaneous mycoses cause chronic localized infections, while systemic mycoses lead to systemic symptoms [53]. Diagnostic methods include clinical examination, microscopy, culture, and molecular techniques [54]. Superficial and cutaneous mycoses’ diagnosis often involves microscopy and the culture of skin samples. Systemic mycoses’ diagnosis may employ blood cultures and serological tests [55]. Treatment varies depending on the infection type and severity [56]. Antifungal agents like azoles and polyenes are common. Topical antifungals suffice for superficial infections, while systemic infections require systemic therapy [57].

### 2.2. Route of Acquisition of the Pathogen

There are two main routes of fungal infections, including exogenous and endogenous origins of disease [61,62,63]. Exogenous mycoses are those that can transmit disease to individuals through an external route, such as airborne, cutaneous, or per-cutaneous contacts [62]. For instance, coccidioidomycosis (valley fever) caused by *Coccidioides immitis* and *C*. *posadasii* can be inhaled by humans when the spores rise in dust storms. Subsequently, it can infect the lungs and surrounding tissues, and various symptoms can occur (i.e., cough, fatigue, fever, headache, muscle aches or joint pains, rashes on the upper body or legs). In addition, paracoccidiomycosis caused by *Paracoccidioides brasiliensis* also occurs by the inhalation of spores and can affect the skin, lungs, lymph nodes, and internal organs [64,65]. In contrast, endogenous mycoses originate from fungi that are part of the normal human microbiota. For instance, *Candida* species exist harmlessly in the body under normal conditions, but when the balance of the immune system or microbiota is disrupted, they can cause infections. In severe cases, *Candida* infections can spread to the veins, leading to potentially life-threatening systemic candidiasis. In particular, candidiasis is the most common endogenous mycosis, affecting mucous membranes, skin folds, and other areas of the body [66,67]. In addition, *Cryptococcus neoformans* infects the lungs or the central nervous system and can cause a pneumonia-like illness with fever, cough, shortness of breath, and chest pains [68,69,70].

### 2.3. Type of Virulence Exhibited by the Fungus

The types of virulence exhibited by fungi can be classified in two ways, including primary infections and opportunistic infections [20,50,71]. Primary infections occur in an immunologically normal host (healthy host) and usually result from the inhalation of fungal spores, which can lead to pneumonia as the primary symptom. For instance, histoplasma capsulatum, which infects humans through the inhalation of infected propagules by healthy, normal individuals, is the cause of histoplasmosis [72,73]. The infection often causes only moderate flu-like symptoms in healthy hosts, but it can be devastating in people with weakened immune systems (e.g., HIV-infected patients). Opportunistic infections occur almost exclusively in immunocompromised patients with weakened immune defense mechanisms [74]. The most common opportunistic infections in immunocompromised patients are *Aspergillus*, *Candida*, *Cryptococcus*, *Fusarium*, and *Pneumocystis* species [75,76].

## 3. Taxonomy of Human Fungal Pathogens

The taxonomy of human fungal pathogens is the systematic classification and identification of fungal species that serve as the causative agents of various fungal infections specifically affecting humans. Notably, fungal taxonomy remains a growing field, evolving continuously with the discovery of new fungal species and the refinement of our comprehension of their genetic similarities [77,78,79,80]. Fungal taxonomy has historically relied on morphological characteristics, such as the shape and size of spores or their hyphal structures, to identify and classify species. While these features are valuable, they can be misleading, as some fungi may show similar morphological traits despite significant genetic differences. Advanced molecular methodologies, such as DNA sequencing including the next generation sequencing (NGS) technologies, have led to significant advancements in fungal taxonomy [81] and revealed hidden diversity within many fungal species. These techniques provide the means to achieve the accurate identification and classification of fungal pathogens [82,83]. Moreover, certain human fungal pathogens demonstrate distinctive characteristics that set them apart from their taxonomic groups. For example, cryptococcus neoformans, a pathogenic belonging to the division basidiomycota, is a significant cause of illness among individuals living with HIV/AIDS, with approximately 152,000 cases of cryptococcal meningitis occurring worldwide each year [84]. Furthermore, the taxonomy of human fungal pathogens provides well-organized classification based on their evolutionary relationships, and the classification of human fungal pathogens holds significant importance in the field of medical mycology for several reasons. Accurate classification allows for the precise identification of fungal pathogens responsible for human infections. This is crucial for effective diagnosis, as different fungal species may require distinct treatment strategies. Misidentification can lead to inappropriate treatments, prolonged illnesses, or even fatal outcomes [85]. Nevertheless, by classifying fungal pathogens, scientists including medical practitioners can better understand the epidemiology of fungal infections. This includes tracking the distribution of specific pathogens, identifying emerging threats, and recognizing and predicting patterns of infection spread [86]. To improve patient care and provide tailored treatment plans, it is important to understand the virulence factors, susceptibilities to antifungals, and resistance mechanisms of various fungal species. Such information can be gained by classification of the pathogenic fungi [87]. To prevent and control diseases, knowledge of fungal taxonomy is critical. A well-defined taxonomy facilitates research into fungal biology, genetics, and pathogenesis. Understanding the evolutionary relationships between fungal species can guide the development of new diagnostic tools, therapies, and vaccines [88]. Therefore, the classification of human fungal pathogens is essential for accurate diagnosis, epidemiological surveillance, treatment optimization, prevention and control efforts, research advancements, and public health interventions. The continuous refinement of fungal taxonomy through advanced molecular techniques ensures that our understanding of fungal diversity and pathogenicity remains up to date, ultimately improving outcomes for patients affected by fungal infections.

**Figure 5:**
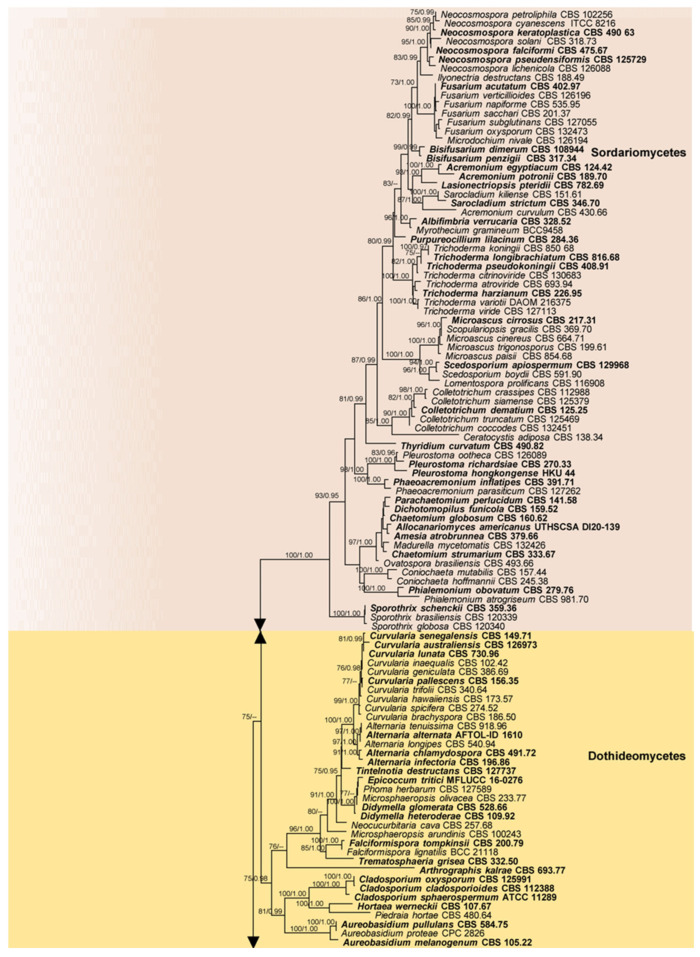

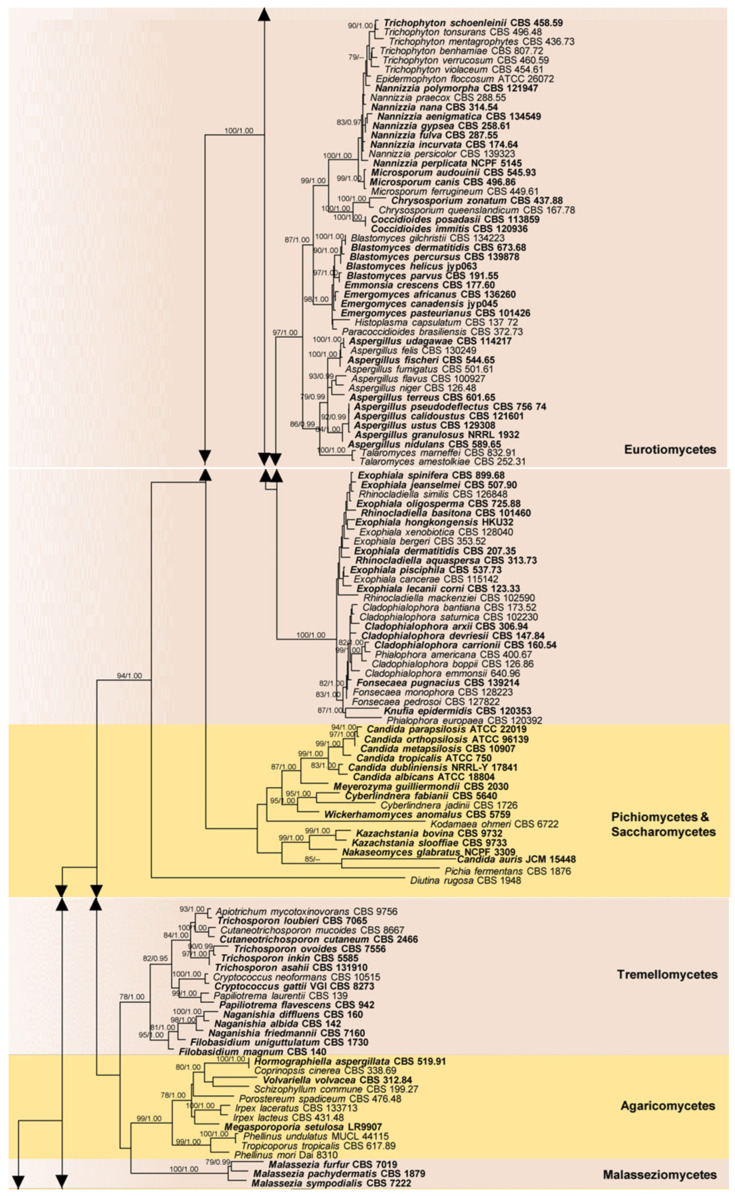

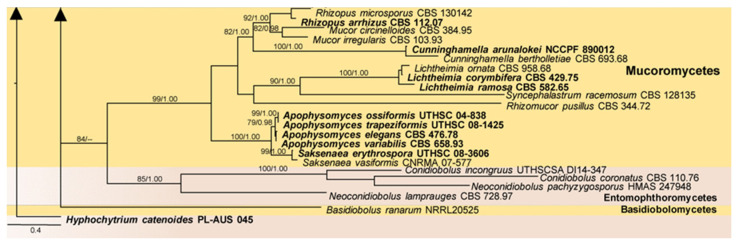
Phylogram resulting from the maximum likelihood (RAxML) analysis of sequence alignment of the 28S nrRNA gene (LSU) sequences of human pathogenic fungi (Supplementary Table S1). ML bootstrap values (MLBS) ≥ 70% and Bayesian posterior probabilities (PP) ≥ 0.90 are at each node. The tree was rooted to *Hyphochytrium catenoides* (PL AUS 045). Classes are indicated with colored blocks to the right of the tree.

The present phylogenetic analysis (Figure 5) of 28S nrRNA gene (LSU) sequence data downloaded from GenBank (Supplementary Table S1) comprises 248 human fungal pathogens distributed in 11 classes and four divisions (ascomycota, basidiomycota, entomophthoromycota, and mucoromycota) of fungi. Current species names have been used in this phylogenetic tree. No type or authentic sequence data are available for the human pathogenic species in pneumocystidomycetes. The results further show the phylogenetic placement of the various human fungal pathogens within the kingdom of fungi and confirm their current taxonomic placements. Most of the fungal pathogens are shown to belong to the class eurotiomycetes.

## 4. Tools and Techniques for the Diagnosis of Fungal Human Infections

Accurate diagnosis is indispensable for the effective management of fungal diseases in humans. This, in turn, requires higher values of sensitivity and specificity. Sensitivity is the ability of a diagnostic tool to identify the presence of an infection and specificity is the ability of the tool to rule out the absence of an infection. While these values vary to a great extent, there is a plethora of techniques used for diagnosing human fungal infections. Among these, the suitable methods of diagnosis depend on the site and the type of infection. In this section, we briefly introduce various types of techniques used to diagnose fungal infections in humans. These methods discussed below also have a prognostic role following treatments.

### 4.1. Fungal Culture

Tissue Biopsies

A biopsy is an examination of tissue removed from a patient. If the infection is associated with peripheral tissues including skin, these tissues are collected and stored in sterile conditions in a laboratory. The biopsy sample is then further investigated using microscopy or culture to verify a fungal infection. A wet skin sample can be obtained using several methods; scrapings of scales can be taken from the edge of a rash, brushings can be taken from an area of the scalp, or adhesive tape could be used to strip off the area of skin to be mounted on a glass slide. Moist swabs can be obtained from mucosal surfaces or bumps and the skin beneath a nail could be used for examination. The tissues in hair roots can also be used to culture fungi.

Human Body Fluids

The analysis of body fluids is a crucial step for the diagnosis of invasive fungal infections (IFIs). However, blood tests are not useful for the diagnosis of superficial fungal infections. In subcutaneous and systemic mycoses, several tests in serum or whole blood samples may be helpful. Urine is also an important specimen that may be informative on urinary tract infections caused by yeast of *Candida* spp. The pleural fluid obtained in many cases of fungal pleuritis is a reliable specimen for the diagnosis of pulmonary fungal infections. Pulmonary aspergillosis is a type of pulmonary fungal infection that is routinely diagnosed by the examination of bronchoalveolar lavage fluid (BALF). In immunocompromised patients, fungal infections in the central nervous system or IFI are normal. These conditions can be diagnosed with direct measurements in cerebrospinal fluid (CSF).

Tissue biopsy or biological fluid samples from the site of infection can be used to culture the fungi that are responsible for the infection in a specific fungal media. Furthermore, culturing is used to select the most suitable treatment options. Growing the fungus in culture may take several weeks, and it is usually incubated at 25–30 °C.

### 4.2. Direct Microscopic Examination

Cultured fungi can be further examined by microscopy as dried smears or wet mounts with or without specific staining. Direct microscopy can identify fungal features such as fungal hyphae or pseudohyphae that make a mycelium or pseudomycelium and fungal spores. A yeast infection can be identified by the presence of round-shaped yeast cells. There are techniques such as laser capture microdissection (LCM) used to acquire fungal cells from the infected tissues under direct microscopic visualization [89,90]. In patients with fungal peritonitis, a direct microscopic examination of peritoneal fluid is used as a method of confirmation.

### 4.3. Serological Methods

Serological methods determine the antibody response in the body against fungal infections. There are five main serological methods: latex antigen method, beta glucan assay, galactomannan test, laser capture microdissection, and enzyme-linked immunosorbent assay (ELISA). The latex agglutination test (LAT) is a rapid test often performed to confirm or type a fungal infection. It detects fungal antigens in patients with systemic fungal infections. Test kits are commercially available with a surface of latex particles coated with antibodies of specific types of fungi and, upon an encounter of the matching antigen from the patient sample, visible agglutination of the latex occurs. Patient samples of saliva, urine, serum, or CSF can be used for the LAT. The detection of an abundant fungal cell wall polysaccharide, Beta-(1,3)-d-glucan (BDG), is an important tool to diagnose IFIs. The serial analysis of BDG can significantly enhance the clinical performance of the assay [91,92]. The galactomannan (GM) test also detects the fungal cell wall antigen galactomannan from patient specimens such as serum, CSF, and BAL. It is an enzyme immunoassay (EIA) that can be used for the early diagnosis of invasive aspergillosis [93].

### 4.4. Imaging Methods

In immunosuppressed patients, taking biopsies routinely is not recommended. Therefore, imaging methods play a pivotal role as they are informative and non-invasive. Diagnostic imaging in fungal infections requires structural changes that may occur at a relatively later phase in the host–fungal interaction process, mostly with established infections. Furthermore, radionuclide imaging is best suited for later phases during recovery compared to early diagnosis [94]. However, immediate inflammation caused by the host response can also cause certain metabolic changes that are imageable and measurable with radionuclide-based tracers. Investigations of fungal infections are conducted mostly using chest X-ray/plain radiography, computerized tomography (CT) scanning, and magnetic resonance imaging (MRI). Ultrasound (US) scans are also useful for cardiac echocardiography (endocarditis). Furthermore, direct observations of fungi are also possible using a bronchoscope (tracheobronchitis) or an endoscope (oesphageal candidiasis or acute invasive fungal rhinosinusitis) [95]. Imaging is important for both initial diagnosis and prognosis over time. In some patients, an enlargement of abnormal areas does not always imply clinical failure, it would rather provide insight into immune reconstitution.

One assay has been developed and FDA-approved for the diagnosis of common *Can-dida* species (T2Candida^®^). Some caveats of using imaging techniques for fungal disease diagnosis can be the lack of radiological expertise to set correct parameters in both CT and MRI methods. Moreover, radiological imaging alone cannot make a precise mycological diagnosis but usually contributes greatly to a full understanding of the extent of the disease and its treatments. However, fluorine-18 fluorodeoxyglucose ([18F]FDG) PET/CT has shown a greater diagnostic performance over stand-alone CT in patients with IFI [96]. The ([18F]FDG) PET/CT has also been reported to identify a mucormycosis fungus ball in the intestine [97].

### 4.5. Molecular-Based Techniques

With the advent of molecular biological techniques, these techniques have greatly replaced the serological detection of systemic IFI in serum and whole-body samples. The culture and isolation of fungi are initially required for the progress of these techniques. Moreover, appropriate reference fungal samples are needed for verification processes. Major molecular-based techniques include PCR, pulse-field gel electrophoresis, restriction enzyme analysis, random amplified polymorphic DNA (RAPD), and amplified fragment length polymorphism (AFLP). 

Variations of PCR including PCR-ELISA and nested and real-time PCR (RT-PCR) are used to detect fungal ribosomal DNA (rDNA) in blood samples. The PCR-ELISA assays, where a quantification step is performed after the PCR, can become negative after a fortnight of therapy. The main advantages of using PCR are the feasibility of conducting tests routinely using easily obtainable serum samples and the reduced detection time, which is generally 4–5 h. Another crucial feature is that PCR results indicate positive even before the symptoms appear. However, the major drawback of PCR is the limited fungal load in samples compared to other bacterial and viral pathogens. 

Nucleic acid sequence-based amplification (NASBA) is another quick method used to differentiate between *Candida* species despite its high cost [98]. Fluorescence in situ hybridization (FISH) is a routinely used detection method for yeast. The accuracy of the FISH technique can be further enhanced using more specific peptide nucleic acid (PAN) probes; thus, it is used to delineate *Candida* species. Microsatellite length polymorphism (MLP) typing, multi-locus sequence typing (MLST), and DNA microarrays are also used to identify different *Candida* species [99]. DNA sequencing is also a useful tool for fungal detection in human samples, though it is not a routinely conducted technique for fungal detection in human samples. From the 18S and 28S ribosomal subunits, 28S has diagnostic values specifically for fungi [100]. 

### 4.6. Challenges in the Diagnosis of Fungal Diseases

Despite all these advanced diagnostic methods, a definite diagnosis of fungal diseases can be challenging at times. The reasons behind unsuccessful cultures may be many. The negative cultures need to be ruled out if: the condition is not a fungal infection, the specimen has not been collected appropriately, the patient had started anti-fungal medications before specimen collection, the fungal strain has a very slow growth rate, or the culture conditions were not optimal for fungal growth. Additionally, some fungal species may be difficult to isolate or identify using traditional culture methods, requiring more specialized techniques such as molecular testing or histopathology. Therefore, alternative diagnostic approaches should be explored to improve the management of fungal diseases.

## 5. Therapeutics for Fungal Diseases

While antifungal agents typically eradicate fungal pathogens without causing toxic effects in humans, some compounds may still exhibit toxicity. There are two types of therapeutics available for fungal diseases. First, traditional antifungal drugs are usually prescribed for mild or severe cases such as skin rashes and systemic infections, respectively. Second is the new antifungal options that have been recently introduced for the treatment of human fungal diseases.

### 5.1. Traditional Antifungal Therapies

There are two types of traditional antifungal drugs: topical and oral. Topical anti-fungal medications are usually over-the-counter drugs in the form of cream, lotion, gel, or spray that can be easily applied to the affected area in the skin. On the other hand, oral antifungal medications are prescribed for more severe infections that resist topical therapies. The main groups of antifungal drugs are polyenes, azoles, allylamine and morpholine, and antimetabolites [20]. These drugs disturb the main survival mechanisms in one species or a broad spectrum of fungi species.

### 5.2. Novel Antifungal Therapies

In recent years, several new antifungal agents and formulations have been developed and introduced. For instance, some of the newly introduced azoles are efinacon-azole, luliconazole, tavaborole, pramiconazole, and eberconazole [101]. In addition to azoles, polyenes, echinocandins, and flucytosine are also used for the treatment of invasive fungal infections. Amphotericin B and its newer lipid formulations are polyene antifungals that target the fungal plasma membrane, and it is the first-ever FDA-approved antifungal for the treatment of invasive fungal infections [102,103]. The echinocandins are the most recently approved class of antifungals and, currently, three echinocandins (caspofungin, micafungin, and anidulafungin) have been developed for clinical usage [102,104]. Flucytosine is a pyrimidine, known to inhibit DNA and RNA synthesis in fungi by incorporating it into the growing nucleic acid chain, preventing further extension [102,105]. Combinations of flucytosine with other antifungals are recommended in therapies of refractory cases including the treatment of infections with resistant fungi, and invasive fungal infections which form biofilms and vegetation.

Herbal treatments from plants such as *Curcuma longa* and *Withania somnifera* have also been proven to be effective in superficial fungal infections. Propolis is another compound that has been shown to be effective [101]. Not only various phytochemicals but also a variety of bioactive compounds produced by microorganisms including fungi are reported to be effective against fungal infections [13]. Further, different drugs in combination are also broadly used for various fungal infections [106]. Synergistic drug combinations increase the effectiveness of drugs while reducing drug resistance [107]. Moreover, there are antifungal vaccines developed for infections of specific fungal or fungal-like species such as *Pythium insidiosum* [108].

There are published treatment regimens for certain fungal infections such as onychomycosis. As systemic treatment recommendations in adults, terbinafine and itraconazole are the first line of treatment for dermatophyte onychomycosis. Fluconazole is a useful alternative in patients who are unable to tolerate the former two medications. If topical monotherapy is ineffective, combination treatment is recommended. As topical treatments for adults, amorolfine or tioconazole are recommended for superficial and distal onychomycosis [109]. Furthermore, there are published global guidelines for the diagnosis and management of infections including cryptococcosis [110], mucormycosis [111], and invasive aspergillosis [112]. However, the access to or the true implementation of these disease diagnosis and management techniques will vary depending on the availability of facilities and funds in different countries.

Invasive fungal infections can be considered a major cause of mortality in the immunocompromised. The effectiveness of antifungal therapy is dependent on the immune status of an individual; thus, antifungal medications are ineffective in the immunosuppressed. Therefore, immunomodulation via immunotherapy increases immune function, and it is a preferred type of treatment for fungal infections in these patients [113,114]. The transfusion of leukocytes, dendritic cells, and neutrophils are different types of cellular therapies and administering growth factors, cytokines (humanIFN-γ), and specific antibodies are augmentative therapies [108]. Gene therapy is also a potential treatment option in immune-deficient individuals with IFIs [15]. 

Despite the wide array, these therapeutic regimes have limitations such as a narrow spectrum of activity, developing resistance, limited efficacy, and toxicity. In this regard, nanotechnology-based formulations address these issues favorably and are reported as a promising therapy against fungal infections [12]. Nanocarriers in the form of liposomes, transferosomes, ethosomes, transethosomes, niosomes, spanlastics, nanoparticles, nanoemulsions, carbon nanotubes, and dendrimers effectively deliver drugs to the diseased site. Nanoemulsions, submicron-sized drug particle transporters, are utilized against the fungal species *Trichophyton rubrum* and *Candida albicans*. By nature, they do not cause drug resistance in fungal species [115]. 

Newer therapeutic options including RNA-based therapies [116] are currently being studied for their potential use in the treatment of fungal infections. RNAi-based therapies have been investigated for various intracellular infections mediated by fungi, while the nanocarrier-mediated delivery of siRNA and shRNA molecules has also been found to overcome the various delivery challenges of these biotherapeutics [117].

## 6. Discussion

Studying fungal pathogens holds paramount importance in the field of microbiology and pathology due to the significant impact of fungal infections on various organisms, including plants, animals, and humans. In the medical realm, fungal pathogens pose a considerable threat to human health, particularly in immunocompromised individuals. Human fungal pathogens can cause a range of infections, from superficial skin conditions to life-threatening systemic diseases. As the incidence of immunocompromised individuals increases due to factors such as HIV/AIDS, organ transplantation, and chemotherapy, the prevalence of opportunistic fungal infections rises. Comprehensive research is essential to developing effective preventive measures, diagnostics, and treatments [118].

Invasive fungal infections, such as those caused by *Candida*, *Aspergillus*, and *Cryp-tococcus* species, are associated with substantial morbidity and mortality, particularly in vulnerable populations [119]. Investigating the virulence factors, host immune responses, and antifungal resistance mechanisms is crucial for improving patient outcomes and reducing the global burden of fungal diseases [120]. The rise of antifungal resistance underscores the need for ongoing research to develop new and improved antifungal drugs. Understanding the molecular mechanisms of resistance and identifying novel drug targets are essential for staying ahead of evolving fungal threats and ensuring effective treatment options [121].

Studying human fungal pathogens provides insights into host–pathogen interactions and immune responses. Fungi have evolved intricate strategies to evade host defenses, and understanding these mechanisms is crucial for developing immunotherapies and vaccines [122]. The accurate and timely diagnosis of fungal infections is essential for initiating appropriate treatment. Research in this field contributes to the development of advanced diagnostic tools, including molecular methods, biomarkers, and imaging techniques, improving our ability to identify and manage fungal diseases [123]. Fungal infections can have significant economic implications and strain healthcare systems. By studying human fungal pathogens, researchers contribute to global health security by enhancing our ability to respond to emerging fungal threats and developing strategies for outbreak control [124].

This review provides a comprehensive list of human fungal pathogens extracted from over 850 recent case reports, and a summary of the relevant disease conditions and their origins (Supplementary Table S2). Only 2–10 of most prominent cases from each pathogen with a confirmed diagnosis of the specific fungal infections based on established diagnostic criteria such as laboratory tests, imaging studies, or clinical findings were selected for this summary. Furthermore, we tried to list the pathogens’ different types of diseases or medical conditions as much as possible. Repeated cases from the same country were excluded. In addition, the present study establishes phylogenetic relationships of the listed pathogens using a phylogenetic analysis based on the available authentic 28S nrRNA gene (LSU) sequence data (Figure 5). This will help clarify the currently correct names for the species based on modern taxonomic concepts since they have often been wrongly quoted and interpreted in the literature and case studies. This will further help with searches for the species in the earlier literature where the old names were used. Additionally, the phylogenetic analysis allows for a better understanding of the evolutionary relationships among these fungal pathogens, which can aid in the development of targeted treatments and diagnostic tools. Furthermore, by providing an updated and accurate list of human fungal pathogens, this study will contribute to the improvement of public health efforts aimed at preventing and controlling these infections globally.

The findings of this review corroborated the taxonomic classification of the numerous human fungal pathogens, with most of the pathogens belonging to the class eurotiomycetes (Table 3). However, authentic LSU sequences for several of the taxa are currently unavailable, so sequence data from the type or credible strains is required to establish their taxonomy. When preparing this evaluation, we discovered that most articles did not employ an acceptable identification strategy to identify fungal diseases, instead relying on traditional identification methods that emphasize morphology. Considering the limitations associated with traditional morphological identification methods, the incorporation of molecular DNA sequence data and phylogenetic techniques in recent studies has significantly advanced the accuracy and reliability of identifying human fungal pathogens [125,126,127,128,129,130,131] (Supplementary Table S2). This step is fundamental for establishing a robust taxonomy and improving our understanding of the diversity and evolutionary relationships among human fungal pathogens. These studies demonstrate how to use DNA sequence data and phylogenetic analysis to reliably identify infections, demonstrating the promise of contemporary molecular approaches to improve diagnostic precision. The incorporation of these approaches into future research endeavors is critical for developing a complete and reliable database of fungal pathogens, which will eventually contribute to more effective disease management and treatment strategies. By using DNA sequence data and phylogenetic tools, researchers can obtain a more precise understanding of the taxonomy of these pathogens. This highlights the importance of incorporating molecular techniques into future studies for the accurate identification and classification of fungal strains. Many mycology laboratories face significant skill shortages, lacking personnel proficient in fungal identification through traditional morphological assessments. The absence of these foundational skills makes it even less likely that such labs would have expertise in cutting-edge molecular diagnostics, further limiting their ability to accurately diagnose and study fungal pathogens. While these methods have historical value, the introduction of molecular tools not only addresses the deficiencies of morphological identification but also provides for more advanced knowledge of fungal pathogen genetic diversity and linkages. Moving forward, the merging of morphological and molecular techniques in fungal taxonomy and identification will most certainly give more precise and complete results, expanding our understanding of the diverse range of human fungal infections.

The nomenclature and classification of many fungal species have been subjected to change during the past few decades due to modern taxonomic approaches and views. Hence, corrections on those changes are mandatory to avoid misinterpretations. In this review, we have given the current names of the fungal species where appropriate, and the data analysis was done based on the current names (Supplementary Table S2). An assay based on modern taxonomic and molecular phylogenetic approaches is highly recommended to identify a given pathogen correctly. These practices will largely minimize the confusion related to this field in the future. Upon analyzing more than 850 articles (Supplementary Table S2) for this study, we discovered that only around 3% of cases contained a thorough examination and the accurate identification of pathogens. The remaining articles either failed to properly study the pathogens or overlooked them entirely, highlighting a significant gap in the accurate identification of pathogens. Furthermore, only a few countries of the world are continuously assessing and publishing cases and data related to human fungal infections. Hence, the actual number of potential disease-causing fungal species and their impact on humans are still understudied. It is crucial to invest in further research and surveillance efforts to accurately identify and document the vast number of fungal species that can cause diseases in humans. This will enable us to better understand the impact of these pathogens on public health and develop effective strategies for prevention and treatment. Additionally, collaboration between scientists, healthcare professionals, and policymakers is essential to ensure that comprehensive data on fungal infections are collected, analyzed, and shared globally. 

### Future Directions

The diagnosis and therapeutics of human fungal diseases are problematic and raise substantial challenges. Accurate diagnosis is crucial and leads to the effective management of fungal diseases in humans. There are a variety of diagnostic tools to identify human fungal infections. With the changing demands of clinical mycology, the field of fungal diagnostics has evolved and both traditional and more advanced approaches such as novel PCR assays, microfluidic chip technology, next generation sequencing, microsatellite length polymorphism (MLP) typing, multi-locus sequence typing (MLST), nanotechnology-based tools, and artificial intelligence-based models [132]. Despite the many antifungal drugs available to date, new therapeutic approaches are urgently needed due to emerging fungal pathogens and increasing antifungal drug resistance. Economic or geographical factors may also play a key role in the incidence and clinical handling of these diseases [133,134,135]. To address these challenges, it is crucial to prioritize research and funding towards studying fungal infections and developing innovative treatment options. Additionally, raising awareness among healthcare professionals and the public about the im-portance of the early detection and proper management of fungal infections can help mitigate their impact on human health. 

The World Health Organization (WHO) released the first-ever fungal priority pathogens list (WHO FPPL) in 2022 to guide the research, development, and public health action regarding fungal infections in order to raise concerns in the population and obtain sufficient fungal infection data. According to the WHO FPPL, establishing an effective fungal pathogen surveillance network, enhancing public health interventions, and providing sustainable support for fungal pathogen infection R&D and innovation should be applied to manage the emergence of fungal infections. It also provides details on the implementation of policy and management interventions to enhance the prevention and treatment of fungal infections [136].

## Figures and Tables

**Table 1:** Examples of cutaneous and subcutaneous mycoses [1,21,28,29,30,31,32].

Site of Infection	Disease	Causal Fungus
Cutaneous tissues	Tinea nigra	*Exophiala werneckii*
	Dermatophytosis	*Microsporum* spp., *Trichophyton* spp., *Epidermophyton* spp.
	Black piedra	*Piedraia hortai*
	White piedra	*Trichosporon beigelii*
Subcutaneous tissues	Sporotrichosis	*Sporothrix schenckii*
	Chromoblastomycosis	*Cladophialophora carrionii*, *Fonsecaea compacta*, *F. pedrosoi*, *Phialophora verrucosa*
	Phaeohyphomycosis	*Cladophialophora* sp., *Exophiala* sp., *Curvularia*, *Exserohilum* spp.
	Mycetoma	*Nocardia brasiliensis*, *Pseudallescheria boydii*
	Entomophthoromycosis	*Basidiobolus ranarum*, *Conidiobolus coronatus*

**Table 2:** Examples of systemic and opportunistic mycoses [20,30,37,38,39].

Mycoses	Disease	Causal Fungus
Systemic mycoses	Aspergillosis	*Aspergillus* spp.
	Blastomycosis	*Blastomyces dermatitidis*
	Candidiasis	*Candida* spp.
	Coccidiodomycosis	*Coccidioides immitis*
	Histoplasmosis	*Histoplasma capsulatum*
	Paracoccidiodomycosis	*Paracoccidioides brasiliensis*
Opportunistic mycoses	Aspergillosis	*Aspergillus fumigatus* *Aspergillus niger* *Aspergillus flavus*
	Candidiasis	*Candida* spp., *Debaryomyces* spp., *Kluyveromyces* spp., *Meyerozyma* spp., *Pichia* spp.
	Cryptococcosis	*Cryptococcus neoformans*
	Fusariosis	*Fusarium* spp.
	Hyalohyphomycosis	*Penicillium* spp., *Paecilomyces* spp., *Beauveria* spp., *Fusarium* spp., *Scopulariopsis* spp.
	Mucormycosis	*Rhizopus* spp. *Mucor* spp. *Absidia* spp.
	Penicilliosis	*Penicillium marneffei*
	Phaeohyphomycosis	*Cladophialophora* spp., *Exophiala* spp., *Bipolaris* spp., *Exserohilum* spp.
	Pneumocystosis	*Pneumocystis jirovecii*
	Scedosporiosis (Pseudallescheriasis)	*Scedosporium* spp., *Lomentospora* spp.
	Mucormycosis	*Rhizopus* spp., *Mucor* spp., *Rhizomucor* spp., *Lichtheimia* spp.

**Table 3:** Classification and number of known human pathogenic fungal species in each genus.

Phylum	Class	Genus	Number of Known Human Pathogenic Species
Ascomycota	Dothideomycetes	*Alternaria*	05
		*Arthrographis*	01
		*Aureobasidium*	03
		*Cladosporium*	03
		*Curvularia*	**14**
		*Didymella*	02
		*Ectophoma*	01
		*Epicoccum*	01
		*Falciformispora*	02
		*Hortaea*	01
		*Microsphaeropsis*	02
		*Neocucurbitaria*	01
		*Phoma*	01
		*Piedraia*	01
		*Tintelnotia*	01
		*Trematosphaeria*	01
	Eurotiomycetes	*Aspergillus*	**16**
		*Blastomyces*	06
		*Chrysosporium*	02
		*Cladophialophora*	07
		*Coccidioides*	02
		*Emergomyces*	05
		*Epidermophyton*	01
		*Exophiala*	**11**
		*Fonsecaea*	04
		*Histoplasma*	01
		*Knufia*	01
		*Microsporum*	03
		*Nannizzia*	09
		*Paecilomyces*	02
		*Paracoccidioides*	03
		*Phialophora*	05
		*Rhinocladiella*	04
		*Talaromyces*	03
		*Trichophyton*	**11**
	Pichiomycetes	*Candida*	07
		*Debaryomyces*	01
		*Diutina*	01
		*Kodamaea*	01
		*Meyerozyma*	01
		*Pichia*	02
	Pneumocystidomycetes	*Pneumocystis*	02
	Saccharomycetes	*Cyberlindnera*	02
		*Nakaseomyces*	01
	Sordariomycetes	*Acremonium*	02
		*Albifimbria*	01
		*Allocanariomyces*	01
		*Amesia*	01
		*Bisifusarium*	02
		*Catunica*	01
		*Chaetomium*	02
		*Chlamydocillium*	01
		*Colletotrichum*	09
		*Coniochaeta*	03
		*Dichotomopilus*	01
		*Fusarium*	07
		*Lasionectriopsis*	01
		*Ilyonectria*	01
		*Kazachstania*	02
		*Lomentospora*	01
		*Madurella*	01
		*Microascus*	06
		*Microdochium*	01
		*Myrothecium*	01
		*Neocosmospora*	07
		*Ovatospora*	01
		*Parachaetomium*	01
		*Phaeoacremonium*	02
		*Phialemonium*	02
		*Pleurostoma*	02
		*Sarocladium*	02
		*Scedosporium*	02
		*Sporothrix*	03
		*Thyridium*	01
		*Trichoderma*	07
		*Wickerhamomyces*	01
		*Xenoacremonium*	01
Basidiomycota	Agaricomycetes	*Coprinopsis*	01
		*Hormographiella*	01
		*Irpex*	02
		*Phellinus*	02
		*Megasporoporia*	01
		*Porostereum*	01
		*Schizophyllum*	01
		*Tropicoporus*	01
		*Volvariella*	01
	Basidiobolomycetes	*Basidiobolus*	01
	Malasseziomycetes	*Malassezia*	03
	Tremellomycetes	*Apiotrichum*	02
		*Cryptococcus*	03
		*Cutaneotrichosporon*	02
		*Filobasidium*	02
		*Naganishia*	03
		*Papiliotrema*	02
Entomophthoromycota	Entomophthoromycetes	*Conidiobolus*	02
		*Neoconidiobolus*	02
Mucoromycota	Mucoromycetes	*Apophysomyces*	04
		*Cunninghamella*	02
		*Lichtheimia*	03
		*Mucor*	02
		*Rhizomucor*	01
		*Rhizopus*	02
		*Saksenaea*	02
		*Syncephalastrum*	01

The numbers in bold indicate the genera comprising 10 or more known human pathogenic species.

## Data Availability

Data are contained within the article and Supplementary Materials.

## Supplementary Materials

The following supporting information can be downloaded at: https://www.mdpi.com/article/10.3390/pathogens13050426/s1, Table S1: GenBank and culture collection accession numbers of human pathogenic species treated in the phylogenetic analysis.; Table S2: A synopsis of human fungal pathogens and relevant disease conditions extracted from case studies.

## References

[1] Brown G.D., Denning D.W., Gow N.A., Levitz S.M., Netea M.G., White T.C. (2012). Hidden killers: Human fungal infections. Sci. Transl. Med..

[2] Bhetariya P.J., Sharma N., Singh P., Tripathi P., Upadhyay S.K., Gautam P. (2017). Human fungal pathogens and drug resistance against azole drugs. Drug Resistance in Bacteria, Fungi, Malaria, and Cancer.

[3] Bastos R.W., Rossato L., Goldman G.H., Santos D.A. (2021). Fungicide effects on human fungal pathogens: Cross-resistance to medical drugs and beyond. PLoS Pathog..

[4] Rokas A. (2022). Evolution of the human pathogenic lifestyle in fungi. Nat. Microbiol..

[5] Fillinger R.J., Anderson M.Z. (2019). Seasons of change: Mechanisms of genome evolution in human fungal pathogens. Infect. Genet. Evol..

[6] Denning D.W. (2024). Global incidence and mortality of severe fungal disease. Lancet Infect. Dis..

[7] Kollath D.R., Melo Teixeira M.D., Barker B.M. (2018). Advances in genomics of human fungal pathogens. Population Genomics: Microorganisms.

[8] Buscaino A. (2019). Chromatin-mediated regulation of genome plasticity in human fungal pathogens. Genes.

[9] Bongomin F., Gago S., Oladele R.O., Denning D.W. (2017). Global and multi-national prevalence of fungal diseases—Estimate precision. J. Fungi.

[10] O’Brien H.E., Parrent J.L., Jackson J.A., Moncalvo J.M., Vilgalys R. (2005). Fungal community analysis by large-scale sequencing of environmental samples. Appl. Environ. Microbiol..

[11] Kim J.Y. (2016). Human fungal pathogens: Why should we learn?. J. Microbiol..

[12] Mishra V., Singh M., Mishra Y., Charbe N., Nayak P., Sudhakar K., Aljabali A.A., Shahcheraghi S.H., Bakshi H., Serrano-Aroca Á. (2021). Nanoarchitectures in management of fungal diseases: An overview. Appl. Sci..

[13] Akhtar N., Ayoubi R., Kour V., Gautam U., Mannan A.U. (2022). Natural products for fungal diseases management and prevention. J. Nat. Prod..

[14] Formanek P.E., Dilling D.F. (2019). Advances in the diagnosis and management of invasive fungal disease. Chest.

[15] Nami S., Aghebati-Maleki A., Morovati H., Aghebati-Maleki L. (2019). Current antifungal drugs and immunotherapeutic approaches as promising strategies to treatment of fungal diseases. Biomed. Pharmacother..

[16] Borman A.M., Linton C.J., Miles S.J., Johnson E.M. (2008). Molecular identification of pathogenic fungi. J. Antimicrob. Chemother..

[17] Campbell C.K., Johnson E.M. (2013). Identification of Pathogenic Fungi.

[18] Hogan L.H., Klein B.S., Levitz S.M. (1996). Virulence factors of medically important fungi. Clin. Microbiol. Rev..

[19] Coleman M., Elkins C., Gutting B., Mongodin E., Solano-Aguilar G., Walls I. (2018). Microbiota and dose response: Evolving paradigm of health triangle. Risk Anal..

[20] Walsh T.J., Dixon D.M. (1996). Spectrum of mycoses. Med. Microbiol..

[21] Köhler J.R., Hube B., Puccia R., Casadevall A., Perfect J.R. (2017). Fungi that infect humans. Microbiol. Spectr..

[22] Keshwania P., Kaur N., Chauhan J., Sharma G., Afzal O., Alfawaz Altamimi A.S., Almalki W.H. (2023). Superficial Dermatophytosis across the World’s Populations: Potential Benefits from Nanocarrier-Based Therapies and Rising Challenges. ACS Omega.

[23] Hainer B.L. (2003). Dermatophyte infections. Am. Fam. Physician.

[24] Elewski B.E. (2000). Tinea capitis: A current perspective. J. Am. Acad. Dermatol..

[25] Alharbi K.S., Joshi N., Singh Y., Kazmi I., Al-Abbasi F.A., Alzarea S.I., Afzal O., Altamimi A.S.A., Gupta G. (2022). Molecular exploration of hidden pleiotropic activities of azoles on dermatophytes in human tinea corporis infection. J. Mycol. Med..

[26] Iorizzo M., Piraccini B.M., Tosti A. (2007). New fungal nail infections. Curr. Opin. Infect. Dis..

[27] Eisman S., Sinclair R. (2014). Fungal nail infection: Diagnosis and management. BMJ.

[28] Bonifaz A., Vázquez-González D., Perusquía-Ortiz A.M. (2010). Subcutaneous mycoses: Chromoblastomycosis, sporotrichosis and mycetoma. J. Dtsch. Dermatol. Ges..

[29] La Hoz R.M., Baddley J.W. (2012). Subcutaneous fungal infections. Curr. Infect. Dis. Rep..

[30] Prasad N., Gupta A. (2005). Fungal peritonitis in peritoneal dialysis patients. Perit. Dial. Int..

[31] Skiada A., Drogari-Apiranthitou M., Pavleas I., Daikou E., Petrikkos G. (2022). Global cutaneous mucormycosis: A systematic review. J. Fungus.

[32] Garcia-Hermoso D., Alanio A., Lortholary O., Dromer F., Jorgensen J.H., Carroll K.C., Funke G., Pfaller M.A., Landry M.L., Richter S.S., Warnock D.W. (2015). Agents of systemic and subcutaneous mucormycosis and entomophthoromycosis. Manual of Clinical Microbiology.

[33] Queiroz-Telles F., McGinnis M.R., Salkin I., Graybill J.R. (2003). Subcutaneous mycoses. Infect. Dis. Clin. N. Am..

[34] Cai Q., Lv G.X., Jiang Y.Q., Mei H., Hu S.Q., Xu H.B., Wu X.F., Shen Y.N., Liu W.D. (2013). The first case of phaeohyphomycosis caused by *Rhinocladiella basitona* in an immunocompetent child in China. Mycopathologia.

[35] Sharma A., Hazarika N.K., Barua P., Shivaprakash M.R., Chakrabarti A. (2013). *Acremonium strictum*: Report of a rare emerging agent of cutaneous hyalohyphomycosis with review of literatures. Mycopathologia.

[36] Pang K.R., Wu J.J., Huang D.B., Tyring S.K. (2004). Subcutaneous fungal infections. Dermatol. Ther..

[37] Ramos-e-Silva M., Lima C.M.O., Schechtman R.C., Trope B.M., Carneiro S. (2012). Systemic mycoses in immunodepressed patients (AIDS). Clin. Dermatol..

[38] Segal B.H. (2009). Aspergillosis. NEJM.

[39] Lionakis M.S. (2014). New insights into innate immune control of systemic candidiasis. Med. Mycol. J..

[40] Turakulovich T.A., Abdullaevich A.F., Sodikmuratovich N.B. (2022). Distribution of Fungi Diseases in Hot Climate Conditions. CCAJMNS.

[41] Razzuk M.A., Urschel H.C., Paulson D.L. (1973). Systemic mycoses—Primary pathogenic fungi. Ann. Thorac. Surg..

[42] Durkin M., Witt J., LeMonte A., Wheat B., Connolly P. (2004). Antigen assay with the potential to aid in diagnosis of blastomycosis. J. Clin. Microbiol..

[43] Wheat L.J. (2006). Antigen detection, serology, and molecular diagnosis of invasive mycoses in the immunocompromised host. Transpl. Infect. Dis..

[44] Brischetto A., Kidd S., Baird R. (2015). Case Report: First reported australian case of *Cladophilophora arxii*: Features consistent with possible primary pulmonary chromoblastomycosis. Am. J. Trop. Med. Hyg..

[45] Benítez I., Rodríguez M., Lezcano V., Morel Z., Pereira J., Brizuela S., Galleano H. (2019). Disseminated histoplasmosis with skin lesions and osteomyelitis in a child with acute lymphoblastic leukemia undergoing maintenance treatment. Pediatría.

[46] Abolghasemi S., Hakamifard A., Sharifynia S., Pourabdollah Toutkaboni M., Azhdari Tehrani H. (2021). Fatal invasive pulmonary aspergillosis in an immunocompetent patient with COVID-19 due to *Aspergillus terreus*: A case study. Clin. Case Rep..

[47] Singh N. (2003). Treatment of opportunistic mycoses: How long is long enough?. Lancet Infect. Dis..

[48] Maertens J., Vrebos M., Boogaerts M. (2001). Assessing risk factors for systemic fungal infections. Eur. J. Cancer Care.

[49] Pana Z.D., Farmaki E., Roilides E. (2014). Host genetics and opportunistic fungal infections. Clin. Microbiol. Infect..

[50] Gnat S., Łagowski D., Nowakiewicz A., Dyląg M. (2021). A global view on fungal infections in humans and animals: Opportunistic infections and microsporidioses. J. Appl. Microbiol..

[51] Kwon-Chung K.J. (1992). Cryptococcosis. Med. Mycol..

[52] Wheat L.J., Freifeld A.G., Kleiman M.B., Baddley J.W., McKinsey D.S., Loyd J.E., Kauffman C.A. (2007). Clinical practice guidelines for the management of patients with histoplasmosis: 2007 update by the Infectious Diseases Society of America. Clin. Infect. Dis..

[53] Perfect J.R., Bicanic T. (2015). Cryptococcosis diagnosis and treatment: What do we know now. Fungal Genet. Biol..

[54] Larone D.H., Larone D.H. (1987). Medically Important Fungi: A Guide to Identification.

[55] Guarner J., Brandt M.E. (2011). Histopathologic diagnosis of fungal infections in the 21st century. Clin. Microbiol. Rev..

[56] Pfaller M.A., Diekema D. (2007). Epidemiology of invasive candidiasis: A persistent public health problem. Clin. Microbiol. Rev..

[57] Denning D.W. (2003). Echinocandin antifungal drugs. Lancet.

[58] Diongue K., Diallo M.A., Seck M.C., Ndiaye M., Badiane A.S., Diop A., Ndiaye Y.D., Ndir O., Ndiaye D. (2016). Tinea pedis due to *Cylindrocarpon lichenicola* beginning onycholysis. Med. Mycol. Case Rep..

[59] Maphanga T.G., Birkhead M., Muñoz J.F., Allam M., Zulu T.G., Cuomo C.A., Schwartz I.S., Ismail A., Naicker S.D., Mpembe R.S. (2020). Human blastomycosis in South Africa caused by *Blastomyces percursus* and *Blastomyces emzantsi* sp. nov., 1967 to 2014. J. Clin. Microbiol..

[60] Ting D.S.J., Galal M., Kulkarni B., Elalfy M.S., Lake D., Hamada S., Said D.G., Dua H.S. (2021). Clinical characteristics and outcomes of fungal keratitis in the United Kingdom 2011–2020: A 10-year study. J. Fungus.

[61] Hariprasad S.M., Mieler W.F., Holz E.R., Gao H., Kim J.E., Chi J., Prince R.A. (2004). Determination of vitreous, aqueous, and plasma concentration of Orally Administered voriconazole in humans. Arch. Ophthalmol..

[62] Sornette D., Yukalov V.I., Yukalova E.P., Henry J.Y., Schwab D., Cobb J.P. (2009). Endogenous versus exogenous origins of diseases. J. Biol. Syst..

[63] Sirisinha S. (2011). Insight into the mechanisms regulating immune homeostasis in health and disease. Asian Pac. J. Allergy Immunol..

[64] Marques S.A. (2012). Paracoccidioidomycosis. Clin. Dermatol..

[65] Peçanha P.M., Bahiense I.C., Kruschewsky W.L.L., Biasutti C., Júnior C.U.G.F., Pinheiro B.G., Maifrede S.B., de Camargo Z.P., Rodrigues A.M., Grão-Velloso T.R. (2021). Paracoccidioidomycosis due to *Paracoccidioides brasiliensis* S1 associated with acquired immunodeficiency syndrome: A case report. Rev. Iberoam. Micol..

[66] Abe M., Katano H., Nagi M., Higashi Y., Sato Y., Kikuchi K., Hasegawa H., Miyazaki Y. (2020). Potency of gastroin-testinal colonization and virulence of *Candida auris* in a murine endogenous candidiasis. PLoS ONE.

[67] Jahagirdar V.L., Davane M.S., Aradhye S.C., Nagoba B.S. (2018). *Candida* species as potential nosocomial pathogens—A review. Electron. J. Gen. Med..

[68] Chang W.C., Tzao C., Hsu H.H., Lee S.C., Huang K.L., Tung H.J., Chen C.Y. (2006). Pulmonary cryptococcosis: Comparison of clinical and radiographic characteristics in immunocompetent and immunocompromised patients. Chest.

[69] Mukaremera L., Nielsen K. (2017). Adaptive immunity to *Cryptococcus neoformans* infections. J. Fungi.

[70] Alegre-González D., Herrera S., Bernal J., Soriano A., Bodro M. (2021). Disseminated *Cryptococcus neoformans* infection associated to COVID-19. Med. Mycol. Case Rep..

[71] Polvi E.J., Li X., O’Meara T.R., Leach M.D., Cowen L.E. (2015). Opportunistic yeast pathogens: Reservoirs, virulence mechanisms, and therapeutic strategies. Cell. Mol. Life Sci..

[72] Conces D.J. (1999). Endemic fungal pneumonia in immunocompromised patients. J. Thorac. Imaging.

[73] Develoux M., Amona F.M., Hennequin C. (2021). Histoplasmosis caused by *Histoplasma capsulatum* var. duboisii: A comprehensive review of cases from 1993 to 2019. Clin. Infect. Dis..

[74] Singh N., Perfect J.R. (2007). Immune reconstitution syndrome associated with opportunistic mycoses. Lancet Infect. Dis..

[75] Tlamçani Z., Er-Rami M. (2013). Fungal opportunist infection: Common and emerging fungi in immunocompromised patients. J. Immunol. Tech. Infect. Dis..

[76] Chakrabarti A., Chatterjee S.S., MR S. (2008). Overview of opportunistic fungal infections in India. Nippon Ishinkin Gakkai Zasshi.

[77] Hongsanan S., Hyde K.D., Phookamsak R., Wanasinghe D.N., Mckenzie E.H.C., Sarma V.V., Boonmee S., Lücking R., Bhat D.J., Liu N.G. (2020). Refined families of Dothideomycetes: Dothideomycetidae and Pleosporomycetidae. Mycosphere.

[78] Hongsanan S., Hyde K.D., Phookamsak R., Wanasinghe D.N., Mckenzie E.H.C., Sarma V.V., Boonmee S., Lücking R., Bhat D.J., Liu N.G. (2020). Refined families of Dothideomycetes: Orders and families *incertae sedis* in Dothideomycetes. Fungal Divers..

[79] Hyde K.D., Norphanphoun C., Maharachchikumbura S.S.N., Bhat D.J., Jones E.B.G., Bundhun D., Chen Y.J., Bao D.F., Boonmee S., Calabon M.S. (2020). Refined families of Sordariomycetes. Mycosphere.

[80] Bhunjun C.S., Niskanen T., Suwannarach N., Wannathes N., Chen Y.J., McKenzie E.H.C., Maharachchikumbura S.S.N., Buyck B., Zhao C.L., Fan Y.G. (2022). The numbers of fungi: Are the most speciose genera truly diverse?. Fungal Divers..

[81] Hongsanan S., Jeewon R., Purahong W., Xie N., Liu J.K., Jayawardena R.S., Ekanayaka A.H., Dissanayake A., Raspé O., Hyde K.D. (2018). Can we use environmental DNA as holotypes?. Fungal Divers..

[82] Lücking R., Aime M.C., Robbertse B., Miller A.N., Aoki T., Ariyawansa H.A., Cardinali G., Crous P.W., Druzhinina I.S., Geiser D.M. (2021). Fungal taxonomy and sequence-based nomenclature. Nat. Microbiol..

[83] Thiele K.R., Applequist W.L., Renner S.S., May T.W., Dönmez A.A., Groom Q., Lehtonen S., Maggs C.A., Malécot V., Yoon H.S. (2023). DNA sequences as types: A discussion paper from the Special-purpose Committee established at the XIX International Botanical Congress in Shenzhen, China. TAXON.

[84] Rajasingham R., Govender N.P., Jordan A., Loyse A., Shroufi A., Denning D.W., Meya D.B., Chiller T.M., Boulware D.R. (2022). The global burden of HIV-associated cryptococcal infection in adults in 2020: A modelling analysis. Lancet Infect. Dis..

[85] Fisher M.C., Hawkins N.J., Sanglard D., Gurr S.J. (2018). Worldwide emergence of resistance to antifungal drugs challenges human health and food security. Science.

[86] Vallabhaneni S., Mody R.K., Walker T., Chiller T. (2016). The global burden of fungal diseases. Infect. Dis. Clin..

[87] Pappas P.G., Lionakis M.S., Arendrup M.C., Ostrosky-Zeichner L., Kullberg B.J. (2018). Invasive candidiasis. Nat. Rev. Dis. Primers.

[88] Benedict K., Jackson B.R., Chiller T., Beer K.D. (2019). Estimation of direct healthcare costs of fungal diseases in the United States. Clin. Infect. Dis..

[89] Espina V., Wulfkuhle J.D., Calvert V.S., VanMeter A., Zhou W., Coukos G., Geho D.H., Petricoin E.F., Liotta L.A. (2006). Laser-capture microdissection. Nat. Protoc..

[90] Westwater C., Schofield D.A. (2012). Laser capture microdissection of Candida albicans from host tissue. Host-Fungus Interactions: Methods and Protocols.

[91] Theel E.S., Doern C.D. (2013). β-D-glucan testing is important for diagnosis of invasive fungal infections. J. Clin. Microbiol..

[92] Wright W.F., Overman S.B., Ribes J.A. (2011). (1–3)-β-D-Glucan Assay: A Review of its Laboratory and Clinical Application. Lab. Med..

[93] Zhou W., Li H., Zhang Y., Huang M., He Q., Li P., Zhang F., Shi Y., Su X. (2017). Diagnostic value of galactomannan antigen test in serum and bronchoalveolar lavage fluid samples from patients with nonneutropenic invasive pulmonary aspergillosis. J. Clin. Microbiol..

[94] Lawal I.O., Mokoala K.M.G., Kgatle M.M., Dierckx R.A.J.O., Glaudemans A.W.J.M., Sathekge M.M., Ankrah A.O. (2021). Radionuclide imaging of invasive fungal disease in immunocompromised hosts. Diagnostics.

[95] Moçin Ö.Y., Karakurt Z., Aksoy F., Güngör G., Partal M., Adigüzel N., Acartürk E., Kutlu S.B., Baran R., Erdem H. (2013). Bronchoscopy as an indicator of tracheobronchial fungal infection in non-neutropenic intensive-care unit patients. Clin. Microbiol. Infect..

[96] Ankrah A.O., Creemers-Schild D., de Keizer B., Klein H.C., Dierckx R.A.J.O., Kwee T.C., Span L.F.R., de Jong P.A., Sathekge M.M., Glaudemans A.W.J.M. (2021). The Added Value of [18F]FDG PET/CT in the Management of Invasive Fungal Infections. Diagnostics.

[97] Gallo F., Vija L., Le Grand S., Moukarbel N., Mortele K., Gabiache E., Courbon F., Tavitian S., Dierick O.D. (2019). Diagnosis of an intestinal mucormycosis ‘fungus ball’ located with PET/CT with [18F] FDG-PET/CT. Eur. J. Hybrid Imaging.

[98] Widjojoatmodjo M.N., Borst A., Schukkink R.A.F., Box A.T.A., Tacken N.M.M., Gemen B.V., Verhoef J., Top B., Fluit A.C. (1999). Nucleic acid sequence-based amplifcation (NASBA) detection of medically important Candida species. J. Microbiol. Methods.

[99] Arafa S.H., Elbanna K., Osman G.E.H., Abulreesh H.H. (2023). *Candida* diagnostic techniques: A review. J. Umm Al-Qura Univ. Appl. Sci..

[100] Wickes B.L., Wiederhold N.P. (2018). Molecular diagnostics in medical mycology. Nat. Commun..

[101] Demirseren D.D. (2020). New therapeutic options in the management of superficial fungal diseases. Dermatol. Ther..

[102] Pianalto K.M., Alspaugh J.A. (2016). New horizons in antifungal therapy. J. Fungi.

[103] Wall G., Lopez-Ribot J.L. (2020). Current antimycotics, new prospects, and future approaches to antifungal therapy. Antibiotics.

[104] Chen S.C.A., Slavin M.A., Sorrell T.C. (2011). Echinocandin antifungal drugs in fungal infections: A comparison. Drugs.

[105] Sigera L.S.M., Denning D.W. (2023). Flucytosine and its clinical usage. Ther. Adv. Infect. Dis..

[106] Campitelli M., Zeineddine N., Samaha G., Maslak S. (2017). Combination Antifungal Therapy: A review of current data. J. Clin. Med. Res..

[107] Vitale R.G. (2021). Role of antifungal combinations in difficult to treat Candida infections. J. Fungus.

[108] Casadevall A., Pirofski L. (2001). Adjunctive immune therapy for fungal infections. Clin. Infect. Dis..

[109] Ameen M., Lear J.T., Madan V., Mohd Mustapa M.F., Richardson M., Hughes J.R., Sahota A., Griffiths M., McDonagh A.J., Punjabi S. (2014). British Association of Dermatologists’ guidelines for the management of onychomycosis 2014. Br. J. Dermatol..

[110] Chang C.C., Harrison T.S., Bicanic T.A., Chayakulkeeree M., Sorrell T.C., Warris A., Hagen F., Spec A., Oladele R., Govender N.P. (2024). Global guideline for the diagnosis and management of cryptococcosis: An initiative of the ECMM and ISHAM in cooperation with the ASM. Lancet Infect. Dis..

[111] Cornely O.A., Alastruey-Izquierdo A., Arenz D., Chen S.C., Dannaoui E., Hochhegger B., Hoenigl M., Jensen H.E., Lagrou K., Lewis R.E. (2019). Global guideline for the diagnosis and management of mucormycosis: An initiative of the European Confederati;on of Medical Mycology in cooperation with the Mycoses Study Group Education and Research Consortium. Lancet Infect. Dis..

[112] Warris A., Lehrnbecher T., Roilides E., Castagnola E., Brüggemann R.J., Groll A.H. (2019). ESCMID-ECMM guideline: Diagnosis and management of invasive aspergillosis in neonates and children. Clin. Microbiol. Infect..

[113] Sam Q.H., Yew W.S., Seneviratne C.J., Chang M.W., Chai L.Y.A. (2018). Immunomodulation as therapy for fungal infection: Are we closer?. Front. Microbiol..

[114] Zhang Z., Bills G.F., An Z. (2023). Advances in the treatment of invasive fungal disease. PLoS Pathog..

[115] Garcia A., Fan Y.Y., Vellanki S., Huh E.Y., Vanegas D., Wang S.H., Lee S.C. (2019). Nanoemulsion as an effective treatment against human-pathogenic fungi. mSphere.

[116] Bruch A., Kenai A.A., Blango M.G. (2022). RNA-based therapeutics to treat human fungal infections. Trends Microbiol..

[117] Dyawanapelly S., Ghodke S.B., Vishwanathan R., Dandekar P., Jain R. (2014). RNA interference-based therapeutics: Molecular platforms for infectious diseases. J. Biomed. Sci..

[118] Badiee P., Hashemizadeh Z. (2014). Opportunistic invasive fungal infections: Diagnosis & clinical management. Indian J. Med. Res..

[119] Enoch D.A., Yang H., Aliyu S.H., Micallef C. (2017). The changing epidemiology of invasive fungal infections. Human Fungal Pathogen Identification: Methods and Protocols.

[120] Pathakumari B., Liang G., Liu W. (2020). Immune defence to invasive fungal infections: A comprehensive review. Biomed. Pharmacother..

[121] Arastehfar A., Lass-Flörl C., Garcia-Rubio R., Daneshnia F., Ilkit M., Boekhout T., Gabaldon T., Perlin D.S. (2020). The quiet and underappreciated rise of drug-resistant invasive fungal pathogens. J. Fungus.

[122] García-Carnero L.C., Pérez-García L.A., Martínez-Álvarez J.A., Reyes-Martínez J.E., Mora-Montes H.M. (2018). Current trends to control fungal pathogens: Exploiting our knowledge in the host-pathogen interaction. Infect. Drug Resist..

[123] Drew R.H., Townsend M.L., Pound M.W., Johnson S.W., Perfect J.R. (2013). Recent advances in the treatment of life-threatening, invasive fungal infections. Expert Opin. Pharmacother..

[124] Ferri M., Ranucci E., Romagnoli P., Giaccone V. (2017). Antimicrobial resistance: A global emerging threat to public health systems. Crit. Rev. Food Sci. Nutr..

[125] Daboit T.C., Magagnin C.M., Heidrich D., Castrillon M.R., Mendes S.D.C., Vettorato G., Valente P., Scroferneker M.L. (2013). A case of relapsed chromoblastomycosis due to *Fonsecaea monophora*: Antifungal susceptibility and phylogenetic analysis. Mycopathologia.

[126] Aguirre C., Euliarte C., Finquelievich J., de los Ángeles Sosa M., Giusiano G. (2015). Fungemia and interstitial lung compromise caused by *Malassezia sympodialis* in a pediatric patient. Rev. Iberoam. Micol..

[127] Borman A.M., Szekely A., Fraser M., Lovegrove S., Johnson E.M. (2019). A novel dermatophyte relative, *Nannizzia perplicata* sp. nov., isolated from a case of tinea corporis in the United Kingdom. Med. Mycol..

[128] Boan P., Pang S., Gardam D.J., Darragh H., Wright M., Coombs G.W. (2020). Investigation of a Lomentospora prolificans case cluster with whole genome sequencing. Med. Mycol. Case Rep..

[129] Rasamoelina T., Maubon D., Andrianarison M., Ranaivo I., Sendrasoa F., Rakotozandrindrainy N., Rakotomalala F.A., Bailly S., Rakotonirina B., Andriantsimahavandy A. (2020). Endemic chromoblastomycosis caused predominantly by *Fonsecaea nubica*, Madagascar. Emerg. Infect. Dis..

[130] Tsang C.C., Chan K.F., Chan W., Chan J.F., Au-Yeung R.K., Ngan A.H., Lin K.P., Lau S.K., Woo P.C. (2021). Hepatic phaeohyphomycosis due to a novel dematiaceous fungus, *Pleurostoma hongkongense* sp. nov., and importance of antifungal susceptibility testing. Emerg. Microbes Infect..

[131] Qiu F., Zhang C.H., Wang J.D., Fan Y.M. (2022). Scrotal tinea caused by *Nannizzia incurvata* in two men using molecular identification. J. Eur. Acad. Dermatol. Venereol..

[132] Fang W., Wu J., Cheng M., Zhu X., Du M., Chen C., Liao W., Zhi K., Pan W. (2023). Diagnosis of invasive fungal infections: Challenges and recent developments. J. Biomed. Sci..

[133] Salmanton-García J., Au W.Y., Hoenigl M., Chai L.Y., Badali H., Basher A., Brockhoff R.A., Chen S.C., Chindamporn A., Chowdhary A. (2023). The current state of laboratory mycology in Asia/Pacific: A survey from the European Confederation of Medical Mycology (ECMM) and International Society for Human and Animal Mycology (ISHAM). Int. J. Antimicrob. Agents.

[134] Salmanton-García J., Hoenigl M., Gangneux J.P., Segal E., Alastruey-Izquierdo A., Akdagli S.A., Lagrou K., Özenci V., Vena A., Cornely O.A. (2023). The current state of laboratory mycology and access to antifungal treatment in Europe: A European Confederation of Medical Mycology survey. Lancet Microbe.

[135] Falci D.R., Pasqualotto A.C. (2019). Clinical mycology in Latin America and the Caribbean: A snapshot of diagnostic and therapeutic capabilities. Mycoses.

[136] Zou G., Wei Y. (2023). World Health Organization’s first-ever release of a fungal priority pathogens list: A reply action proposal for the prevention and treatment of fungal pathogens. Eco-Environ. Health.

