# Supplementary material for: Humans vs. Fungi: An Overview of Fungal Pathogens against Humans
Source: Pathogens. 2024 May 17;13(5):426. doi: 10.3390/pathogens13050426 (PMC11124197; doi:10.3390/pathogens13050426)
Supplement: Supplementary file 1 [file pathogens-13-00426-s001.zip › Supplementary Table 2.pdf]

Supplementary Table 2 A synopsis of human fungal pathogens and relevant disease conditions extracted from case studies.

Current Name	Fungal Pathogen	Disease or medical condition	Site/tissue of infection	Origin	References
Acremonium egyptiacum	*Acremonium sclerotigenum*	Onychomycosis	Toenail	France & Netherlands	Perdomo et al. 2011
Acremonium potronii	*Acremonium potronii*	Keratitis	left eye	Spain	Rodriguez-Ares et al. 2000
		Pneumonia	lungs	Turkey	Civelek et al. 2016
Albifimbria verrucaria	*Myrothecium verrucaria*	Keratomycosis	eye	China & India	Rameshkumar et al. 2019, Liu et al. 2021
Allocanariomyces americanus	*Pseudocanariomyces americanus*	Prosthetic hipinfection	hip	USA	Ryan et al. 2021
		Prosthetic Hip Infection	hip	USA	Ryan et al. 2021
Alternaria Alternata	*Alternaria Alternata*	Cutaneous phaeohyphomycosis,	right knee	Italy, Germany	Mayser et al. 2002, Farina et al. 2007
		Soft tissue infection	thigh	Austria	Bonatti et al. 2007
		Keratomycosis	eye	Malta	Zahra et al. 2002
		Keratitis	eye	Greece	Konidaris et al. 2013
		Cutaneous phaeohyphomycosis	left hand and wrist	Italy	Romano et al. 1997
		Osteomyelitis	maxilla	India	Chhabra et al. 2013
		Bronchopulmonary mycosis	Lungs	India	Chowdhary et al. 2012
		Eosinophilic pneumonia	lungs	Japan	Ogawa et al. 1997
Alternaria chlamydospora	*Alternaria chlamydospora*	malignant otitis externa	ear	Greece	Korres et al. 2015
		Ungual and cutaneous phaeohyphomycosis	toenail and the plantar area of the toe	India	Singh et al. 1990
Alternaria infectoria	*Alternaria infectoria*	Cutaneous phaeohyphomycosis	right forearm, right leg, right knee & right foot	France, Germany, Netherlands, & Portugal	Dubois et al. 2005, Nulens et al. 2006, Brasch et al. 2008, Cunha et al. 2012, Robert et al. 2012, Kieselová et al. 2021
		Cutaneous infection	right arm, third finger of the right hand, & legs	Italy, & Portugal	Lo Cascio et al. 2004, Ara et al. 2006, Lopes et al. 2013
		Invasive Rhinosinusitis	nose	France	Paccoud et al. 2022

		Phaeohyphomycotic brain abscess	brain	Portugal	Hipolito et al. 2009
		Posttraumatic keratitis and endophthalmitis	eye	Spain	Ferrer et al. 2003
Alternaria longipes	*Alternaria longipes*	Cutaneous phaeohyphomycosis	left forearm & right foot	Spain	Gene et al. 1995
Alternaria tenuissima	*Alternaria tenuissima*	cutaneous phaeohyphomycosis,	right leg	Italy	Romano et al. 1997
		Cutaneous infection	right knee	UK	Robertshaw and Higgins 2005
		Cutaneous phaeohyphomycosis	left elbow	Italy	Romano et al. 1996
Amesia atrobrunnea	*Chaetomium atrobrunneum*	Cerebral abscess	left parietal lobe	USA	Guppy et al. 1998
		Eumycetoma	left foot	Sudan	Mhmoud et al. 2019
		Keratitis	surface epithelium of the cornea, eyelid	india , china	Balne et al. 2012, Zhang et al. 2010
		Cerebral abscess	left parietal lobe,	USA	Guppy et al. 1998
		Eumycetoma	left foot	Sudan	Mhmoud et al. 2019
		Fungal Keratitis	surface epithelium of the cornea, eyelid	india , china	Balne et al. 2012, Zhang et al. 2010
Apiotrichum loubieri	*Trichosporon loubieri*	Disseminated infection	blood	spleen & liver	Marty et al. 2003
		Urinary tract infection	Urinary tract	India	Premamalini et al. 2019
		Kidney infection	Kidney	India	Padhye et al. 2003
		Fungemia	blood	USA	Bhaskaran et al. 2016
		bloodstream infection	lungs & spleen	China	Hu et al. 2021
Apiotrichum mycotoxinovorans	*Trichosporon mycotoxinivorans*	Disseminated Infections	respiratory tract	USA	Shah et al. 2014
Apophysomyces elegans	*Apophysomyces elegans*	Posttraumatic Necrotizing Fasciitis	left arm & right clavicle	Saudi Arabia	Kordy et al. 2004
		Cutaneous infection	left upper arm	India	Chakrabarti et al. 1997
		Rhino-Orbitocerebral infection	right eye, right maxillary sinus, right maxillary region, infraorbital foramen, floor of orbit, & right	India & USA	Garcia-Covarrubias et al. 2001, Liang et al. 2006, Parsi et al. 2013

			cavernous sinus		
		Osteomyelitis	left humerus & sternum	Georgia & Netherlands	Eaton et al. 1994, Meis et al. 1994
		Sinoorbital infections	frontal, maxillary, ethmoid, & sphenoid sinuses	India	Rao et al. 2006
		Renal infection	right kidney	India	Okhuysen et al. 1994, Thomas et al. 2008
Apophysomyces ossiformis	*Apophysomyces ossiformis*	Rhino-orbital infection	left eye & maxillary sinuses	Mexico	Martínez-Herrera et al. 2020
Apophysomyces trapeziformis	*Apophysomyces trapeziformis*	Necrotizing soft tissue infection	right arm	USA	Echaiz et al. 2013
		Septic arthritis	left knee	USA	Bertumen et al. 2016
		Necrotizing Cutaneous infection	flank, legs, scalp, thigh & hip	USA	Neblett Fanfair et al. 2012
Apophysomyces variabilis	*Apophysomyces variabilis*	Chronic orbital and calvarial infection	left eye, calvarial bone, & left maxillary bone	USA	Wolkow et al. 2017
		Necrotizing fasciitis	upper & lateral region of thigh and right gluteus to the upper region of the left gluteus, & right leg	Colombia & India	Rodríguez et al. 2018, Samaddar et al. 2019
		Cutaneous infection	left leg	Saudi Arabia	Al-Zaydani et al. 2015
		Maxillary Osteomyelitis	maxilla	India	Sharma et al. 2014
		Unrecognized Infection	_	Australia	Boan et al. 2022
Arthrographis kalrae	*Arthrographis kalrae*	Mycetoma	left hand	France	Degavre et al. 1997
Aspergillus calidoustus	*Aspergillus calidoustus*	Pulmonary infections	lungs	Canada	Egli et al. 2012
		Mediastinitis	mediastinum	USA	Elsawy et al. 2015
		Intestinal mycosis	colon	Japan	Kaneko et al. 2018
		Disseminated infection	brain, skin, & lung	USA	CamMendoza et al. 2020, Camargo et al. 2022
		Pulmonary infection	lungs	Spain	Pelaez et al. 2013
Aspergillus felis	*Aspergillus felis*	Pulmonary aspergillosis	lungs	Japan & Spain	Barrs et al. 2013, Yamairi et al. 2019
		Invasive pulmonary aspergillosis and allergic	lungs	France	Paccoud et al. 2019

		bronchopulmonary aspergillosis			
		Invasive rhinosinusitis with secondary cerebral abscesses	brain, clivus	Australia	Parkes-Smith et al. 2022
		Cranial aspergillosis	brain (left parieto-temporal region)	Australia	Beresford et al. 2019
Aspergillus fischeri	*Aspergillus fischeri*	Pneumonia	lungs	Hong Kong	Chim et al. 1998
		Disseminated infection	lung, heart, spleen, thyroid, skin, & brain	USA	Lonial et al. 1997
		Skin graft infection	upper right extremity	Czech Republic	Lipový et al. 2021
Aspergillus flavus	*Aspergillus flavus*	Keratitis	eye	India	Sridhar et al. 2000
		Endocarditis	mitral valve, right ventricular wall and near the mitral and tricuspid valve leafets	France & UK	Kennedy et al. 1998, Demaria et al. 2000, Rao and Saha 2000, Irles et al. 2004
		Endocarditis & meningitis,	anterior mitral leaflet hinge point, atrial septum	Iran	Fattahi et al. 2020
		Invasive stomatitis	oral cavity	Japan	Myoken et al. 2003
		Anuria	ureters	USA	Kueter et al. 2002
		Cutaneous aspergillosis	abdominal skin	USA	James et al. 2000
		Isolated nasopharyngeal aspergillosis	nasopharynx	USA	Dogan et al. 2004
		Eumycetoma	left foot	Sudan	Ahmed et al. 2015
Aspergillus fumigatus	*Aspergillus fumigatus*	Invasive aspergillosis	lungs, sinuses, & brain	France	Pinel et al. 2003
Aspergillus granulosus	*Aspergillus granulosus*	Disseminated skin infection	right arm & right leg	USA	Fakih et al. 1995
Aspergillus lentulus	*Aspergillus lentulus*	Invasive pulmonary aspergillosis	lungs	Brazil, China, & Japan	de Azevedo Bastos et al. 2015, Yoshida et al. 2015, Yagi et al. 2019, Yu et al. 2020
		Pneumonia	right lung	Turkey	Gürcan et al. 2013
		Disseminated infection (pulmonary disease, endophthalmitis, and a cerebral abscess)	brain, lungs, & eye	Australia	Shivasabesan et al. 2022

Aspergillus nidulans	*Aspergillus nidulans*	Vertebral osteomyelitis and epidural absces	cervicothoracic spine	China	Jiang et al. 2013
		Invasive aspergillosis	spinal cord	Iran	Tavakoli et al. 2020
		Osteomyelitis	left femur	Greece	Dotis et al. 2003
		White granule mycetoma	right foot and leg	India	Prasanna et al. 2016
		Brain infection	brain	India	Chakrabarti et al. 2006
		Spinal cord infection	spinal cord	Saudi Arabia	Bukhari and Alrabiaah 2009
Aspergillus niger	*Aspergillus niger*	Aspergillosis	lungs	Belgium, Italy, & USA	Fianchi et al. 2004, Person et al. 2010, Vermeulen et al. 2014
		Onychomycosis	Fingernails	South Korea	Kim et al. 2012
		Disseminated infection	liver, skin, spleen, sclera, inguinal sites	Turkey	Ergene et al. 2013
Aspergillus novofumigatus	*Aspergillus novofumigatus*	Invasive pulmonary aspergillosis	lungs	Spain	Pelaez et al. 2013
Aspergillus pseudodeflectus	*Aspergillus pseudodeflectus*	Invasive Pulmonary aspergillosis	lungs	France	Aït-Ammar et al. 2018
Aspergillus reptans	*Aspergillus repens*	Onychomycosis	toe-nails of left foot	Korea	Lee et al. 1981
Aspergillus terreus	*Aspergillus terreus*	Pulmonary aspergillosis	lungs	Iran & UK	Iwen et al. 1998, Steinbach et al. 2004, Abolghasemi et al. 2021
		Aneurysm	aortic arch	USA	Silva et al. 2000
		Sinusitis	paranasal sinuses	Morocco	Akhaddar et al. 2008
		Meningitis	ethmoidal, sphenoid, and maxillary sinuses	Saudi Arabia	Elsawy et al. 2015
		Spondylodiscitis	spine	France	Comacle et al. 2016
		Hip joint infection	hip joint	USA	Bartash et al. 2017
		Cutaneous infection	left leg	Turkey	Ozer et al. 2009
Aspergillus udagawae	*Aspergillus udagawae*	Bronchial aspergillosis	Right bronchus	Japan	Gyotoku et al. 2012
		Endocarditis	posterior leaflet of the mitral valve	Japan	Seki et al. 2017
	Neosartorya udagawae	Pneumonia and Dissaminated infection	lungs, chest wall, & liver	USA	Vinh et al. 2009a
Aspergillus ustus	*Aspergillus ustus*	Cutaneous infection	right cheek, right leg, & left wrist	Japan, Spain, & USA	Gené et al. 2001, Nakai et al. 2002, Vagefi et al. 2008
		Disseminated infection	left arm	USA	Olorunnipa et al. 2010
		Cerebral aspergillosis	brain	USA	Florescu et al. 2009

		Endophthalmitis	eye	USA	Taner Yildiran et al. 2006
Aspergillus viridinutans	*Aspergillus viridinutans*	Disseminated infection	superior vena cava and anterior pericardium, lungs,diaphragm, & spleen	USA	Vinh et al. 2009
		Keratomycosis	eye (contact lens)	Japan	Shigeyasu et al. 2012
		Lung infection	left lower lobe	Japan	Kitaura et al. 2014
		Chronic invasive aspergillosis	upper lobe of the right lung	Portugal	Coelho et al. 2011
Aureobasidium melanogenum	*Aureobasidium melanogenum*	Superficial Phaeohyphomycosis	skin	Taiwan	Chen et al. 2016
		Neonatal fungemia	blood	India	Samaddar et al. 2023
		Bloodstream infection	blood	Japan	Yamamoto et al. 2022
		Superficial Phaeohyphomycosis	skin	Taiwan	Chen et al. 2016
		Neonatal fungemia	blood	India	Samaddar et al. 2023
		Bloodstream infection	blood	Japan	Yamamoto et al. 2022
Aureobasidium proteae	*Aureobasidium proteae*	Chronic fungal meningitis	brain	Croatia	Kutleša et al. 2012
		Chronic fungal meningitis	brain	Croatia	Kutleša et al. 2012
Aureobasidium pullulans	*Aureobasidium pullulans*	Fungemia	blood	canada	Hawkes et al. 2005
		Peritonitis	peritoneal fluid	Japan	Mise et al. 2008
		Septicemia	blood, catheter tip	Taiwan	Huang et al. 2008
		Fungemia	blood	canada	Hawkes et al. 2005
		Peritonitis	peritoneal fluid	Japan	Mise et al. 2008
		Septicemia	blood, catheter tip	Taiwan	Huang et al. 2008
Basidiobolus ranarum	*Basidiobolus ranarum*	Subcutaneous infection	left thigh & buttocks	India	Sujatha et al. 2003, Anand et al. 2010, Mendiratta et al. 2012
		Entomophthoromycosis	abdomen	Australia	Davis et al. 1994
		Gastrointestinal infection	cecum, appendix, right colon, liver, & abdominal lymph nodes	Saudi Arabia	Yusuf et al. 2003
		Retroperitoneal infection	upper abdomen	Pakistan	Nazir et al. 1997

Blastomyces gilchristii	*Blastomyces gilchristii*	Blastomycosis, acute respiratory distress syndrome	lungs	Canada	Dalcin et al. 2016
	Blastomyces dermatitidis	Osteomyelitis, USA	elbow, Left distal femur, right proximal radius, manubrium & sternoclavicular joint	USA	Saiz et al. 2004
		otitis media	middle ear	USA	Istorico et al. 1992
		Meningoencephalitis & central nervous system infection	CNS	Canada & USA	Hardjasudarma et al. 1995, Wylen and Nanda 1999, Friedman et al. 2000, Chowfin et al. 2000, Madigan et al. 2017, Walkty et al. 2018, Slomka and Doub 2020
		endophthalmitis & Orbital cellulitis	eye	Canada & USA	Safneck et al. 1990, Li et al. 1998
		Cutaneous infections	scalp, forehead, neck, and ears, trunk, right leg and proximal extremities	Canada, Tunisia, South Africa, & USA	Ross and Keeling 2000, Walker et al. 2002, Motswaledi et al. 2012, Salem et al. 2017
		Optic Neuropathy	left eye	USA	Pariseau et al. 2007
		Laryngeal Blastomycosis	vocal folds	Georgia & USA	Ebeo et al. 2002, Ballestas et al. 2020
		Genitourinary tract infection	genitourinary tract	USA	Eickenberg et al. 1975, Vahidi et al. 2018
		Meningitis	brain	USA	Brown et al. 2015
		Pulmonary blastomycosis	lungs	India, Mexico, & USA	Randhawa et al. 1983,Guccion et al. 1996, De Groote et al. 2000, Velázquez et al. 2003
		Cholangitis	bile ducts	USA	Ryan et al. 1989
		Splenic abscess	spleen	Canada	MacDonald et al. 1992
		Peritonitis	abdomen	USA	Barocas and Gauthier 2014
Blastomyces emzantsi	*Blastomyces emzantsi*	Blastomycosis	lungs, vertebrae, & skin	South Africa	Maphanga et al. 2020
Blastomyces helicus	*Blastomyces helicus*	Pulmonary and Systemic Disease	lungs, CNS, & blood	USA	Schwartz et al. 2019
Blastomyces parvus	*Chrysosporium parvum*	Keratomycosis	left eye	USA	Wagoner et al. 1999
		Endophthalmitis	right eye	South Korea	Park et al. 2006

Blastomyces percursus	*Blastomyces percursus*	Blastomycosis	tongue, lungs, cutaneous lesions, ver, lymph nodes, kidney, brain, & spleen	Angola, Morocco, Mozambique, South Africa, & Uganda	Maphanga et al. 2020, Schwartz et al. 2021
Candida albicans	*Candida albicans*	Candidemia	blood	Japan, USA	Bodey et al. 2002, Nakamura and Takahashi 2006, Chow et al. 2008
		Sternal osteomyelitis	sternum	Greece	Petrikkos et al. 2001
		Endocarditis	ventricular endocardium	France	Roger et al. 2000
		Pacemaker endocarditis	right atrium	France	Joly et al. 1997
		Mycotic arteritis	arterial walls	France	Mai et al. 2006
		Vulvovaginal candidiasis	vagina	USA	Geiger et al. 1995
		Osteomyelitis	talus	Venezuela	Arias et al. 2004
		Vertebral osteomyelitis and Diskitis	Vertebral column	France, UK & USA	Hennequin et al. 1996, Miller and Mejicano 2001, Parry et al. 2001, Khazim et al. 2006
Candida auris	*Candida auris*	Ear infection	external ear canal	Japan & USA	Satoh et al. 2009, Vallabhaneni et al. 2016, Ruiz & Lorenz 2021
		Fungaemia	blood	Colombia, India, Kuwait, Netherlands, Oman, Pakistan, South Africa, South Korea, Spain, UK, & USA	Lee et al. 2011, Magobo et al. 2014, Emara et al. 2015, Calvo et al. 2016, Schelenz et al. 2016, Vallabhaneni et al. 2016, Gaitán et al. 2017, Mohsin et al. 2017, Rudramurthy et al. 2017, Adams et al. 2018, Govender et al. 2018, Mathur et al. 2018, Parra-Giraldo et al. 2018, Vogelzang et al. 2019, Moin et al. 2021
		Otomastoiditis	temporal bones	South Korea	Choi et al. 2017
		Disseminated infections	blood, urine and tracheal aspirate, central line tip, respiratory tract, & skin	Lebanon, Oman, Russia, & USA	Vallabhaneni et al. 2016, Barantsevich et al. 2019, Al-Rashdi et al. 2021, Allaw et al. 2021
		Pneumonia, fungaemia, & disseminated infections	blood, axilla, respiratory tract, groin, auditory canal, urinary tract, throat, urine, nose	Qatar, & Switzerland	Riat et al. 2018, Shaukat et al. 2021
Candida dubliniensis	*Candida dubliniensis*	Fungaemia	blood	USA	Brandt et al. 2000

Candida metapsilosis	*Candida metapsilosis*	Candidemia	blood	China, Spain, & USA	Tavanti et al. 2005, Kosa et al. 2006, Gacser et al. 2007, Cantón et al. 2011, Guo et al. 2021
		Vulvovaginal candidiasis	vagina	China	Zhu et al. 2015
Candida orthopsilosis	*Candida orthopsilosis*	Candidemia	blood	China, Jamaica, Spain, USA	Tavanti et al. 2005, Kosa et al. 2006, Gacser et al. 2007, Trofa et al. 2008, Cantón et al. 2011, Guo et al. 2021
		Septic arthritis	joints	Jamaica	Heslop et al. 2015
		Vulvovaginal candidiasis	vagina	China	Zhu et al. 2015
Candida parapsilosis	*Candida parapsilosis*	Candidemia & invasive candidiasis	blood	Brazil, China, Germany Hungary, India, Japan, & Spain	Brito et al. 2006, Medrano et al. 2006, Nakamura and Takahashi 2006, Kocsube et al. 2007, Xess et al. 2007, Gacser et al. 2005, Cantón et al. 2011, Guo et al. 2021
		Vaginitis & vulvovaginal candidiasis	vagina	Italy	De Bernardis et al. 1989
		Superficial candidiasis (Cutaneous & Onychomycotic infections)	fingernail, toenail, groin, neck & folds of genitals	China	Feng et al. 2012
Candida tropicalis	*Candida tropicalis*	Osteomyelitis, Vertebral bone,	vertebrae	Australia	Eisen et al. 2000
		Septic arthritis	right knee	China, & Netherlands	Weers-Pothoff et al. 1997, Wang et al. 2007
Catunica adiposa	*Ceratocystis adiposa*	Rhinosinusitis	paranasal sinuses	India	Agarwal et al. 2014
Chaetomium globosum	*Chaetomium globosum*	Pulmonary mycosis	lung tissues and bronchoalveolar lavage fluid	India	Capoor et al. 2016
		Subcutaneous phaeohyphomycosis	face, Skin and nail samples	China	Yu et al. 2006, Tullio et al. 2010
		Onychomycosis	finger nails & great toenail	India & Spain	Aspiroz et al. 2007, Latha et al. 2010
		Invasive pulmonary mycosis	lungs	India	Capoor et al. 2015
		Onychomycosis	right toenails and left toenails, right hand nail plates	India & Korea	Latha et al. 2010, Kim et al. 2013
		Pulmonary mycosis	lung tissues and bronchoalveolar lavage fluid	India	Capoor et al. 2016
		Subcutaneous phaeohyphomycosis	face, Skin and nail samples	China	Yu et al. 2006, Tullio et al. 2010

		Onychomycosis	right toenails and left toenails, right hand nail plates	Korea, India	Latha et al. 2010, Kim et al. 2013
		Cerebral phaeohyphomycosis	lung tissue	USA	Barron et al. 2003
		Cerebral Phaeohyphomycosis	lung tissue	USA	Barron et al. 2003
Chaetomium strumarium	*Chaetomium strumarium*	Cerebral infection	brain	USA	Aribandi et al. 2005
		Corneal ulcer	right eye	IndiA	Reddy et al. 2017
		Subcutaneous mycosis	left upper arm and shoulder and the adjoining left side of his chest and upper back	India	Verma et al. 2015
		cerebral infection	brain	USA	Aribandi et al. 2005
		Corneal ulcer	right eye	IndiA	Reddy et al.2017
Chaetomium strumarium	*Chaetomium strumarium*	subcutaneous mycosis	left upper arm and shoulder and the adjoining left side of his chest and upper back	India	Verma et al. 2015
Chlamydocillium curvulum	*Acremonium curvulum*	Chronic osteomyelitis	tibia	India	Khan et al. 2019
Chrysosporium queenslandicum	*Chrysosporium articulatum*	Pulmonary infection	lungs	Thailand	Suankratay et al. 20156
Chrysosporium zonatum	*Chrysosporium zonatum*	Disseminated Infection	lunga & bones	Canada	Roilides et al. 1999
		Pulmonary infection	lungs	Japan & Mexico	Siddiqui and Zimmerman 2016, Matsuzaki et al. 2021
Cladophialophora arxii	*Cladophialophora arxii*	Femoral Osteomyelitis	left femur	Japan	Shigemura et al. 2009
		Pulmonary infection	right lung	Australia	Brischetto et al. 2015
Cladophialophora bantiana	*Cladophialophora bantiana*	Brain abscess	cerebellum, parietal lobe, & left frontal lobe	China, India, & USA	Arunkumar et al. 2000, Levin et al. 2004, Borkar et al. 2008, Huang et al. 2011, Aljuboori et al. 2017
		Disseminated infection	skin (left shoulder),	Germany & USA	Keyser et al. 2002, Mansour et al. 2014

			brain (right cerebellar hemisphere), lungs, & CNS		
Cladophialophora bantiana	*Cladophialophora bantiana*	Osteomyelitis	left lower limb	Belgium	Desmet et al. 2016
Cladophialophora boppii	*Cladophialophora boppii*	Pulmonary infection	parenchymal & bronchial infection	Italy	Lastoria et al. 2009
		Cerebral infection	brain (left hemisphere)	Turkey	Kantarcioglu et al. 2016
		Toenail infection	left big toenail	Germany	Brasch et al. 2011
Cladophialophora carrionii	*Cladophialophora carrionii*	Chromoblastomycosis	left arm, lower left limb, & lower back	India & Mexico	Pradeepkumar and Joseph 2011, Rojas et al. 2015
		Subcutaneous infection	right foot	Austria	Parente et al. 2011
Cladophialophora devriesii	*Cladophialophora devriesii*	Sub-cutaneous infection	right forearm	UK	Howard et al. 2006
Cladophialophora emmonsii	*Xylohypha emmonsii*	Subcutaneous infection	left arm	USA	Padhye et al. 1988
Cladophialophora saturnica	*Cladophialophora saturnica*	Cutaneous infection	Interdigital webs	Brazil	Badali et al. 2009
Cladosporium cladosporioides	*Cladosporium cladosporioides*	Keratomycosis	left eye	Malaysia	Fiona et al. 2009
		Acne-like subcutaneous phaeohyphomycosis	face and neck	China	Zhou et al. 2016
		Hemorrhagic pneumonia	lung	Brazil	Grava et al. 2016
		Phaeohyphomycosis	subcutaneous asymptomatic nodule in the left thigh	Portugal	Vieira et al. 2001
		keratomycosis	left eye	Malaysia	Fiona, et al. 2009
		Acne-Like Subcutaneous Phaeohyphomycosis	face and neck	China	Zhou et al. 2016
		hemorrhagic pneumonia	lung	Brazil	Grava et al. 2016
Cladosporium cladosporioides	*Cladosporium cladosporioides*	Phaeohyphomycosis	subcutaneous asymptomatic nodule in the left thigh	Portugal	Vieira et al. 2001

Cladosporium oxysporum	*Cladosporium oxysporum*	Cutaneous phaeohyphomycosis	right leg, left foot	Italy, India	Romano et al. 1999, Gugnani et al. 2006
		Unilateral Cervical Lymphadenopathy	neck	Sri Lanka	Jayasinghe et al. 2017
		Cutaneous phaeohyphomycosis	right leg, left foot	Italy, India	Romano et al. 1999, Gugnani et al. 2006
		Unilateral Cervical Lymphadenopathy	neck	Sri Lanka	Jayasinghe et al. 2017
Cladosporium sphaerospermum	*Cladosporium sphaerospermum*	Cerebral abscess	brain	India	Batra et al. 2019
		Subcutaneous infection	dorsum of right hand	India	Maduri et al. 2015
		Invasive lung infection	respiratory tract	USA	Villanueva et al. 2022
		Acute Meningitis	cerebral spinal fluid	Taiwan	Lai et al. 2013
		Cerebral abscess	brain	India	Batra et al. 2019
		Subcutaneous infection	dorsum of right hand	India	Maduri et al. 2015
		Cladosporium sphaerospermum-induced invasive lung infection	respiratory tract	USA	Villanueva et al. 2022
		Acute Meningitis	cerebral spinal fluid	Taiwan	Lai et al. 2013
Coccidioides immitis	*Coccidioides immitis*	Vasculitis, Coccidioidal Meningitis (CM)	CNS, blood vessels & brain	Mexico	Williams et al. 1992
		Pneumonia	lungs	USA	Swartz et al. 2009
		Disseminated osteomyelitis	bones or joints	USA	Holley et al. 2002
		Coccidioidomycosis (disseminated infection)	lungs & skin	USA	Linsangan and Ross 1999
		Coccidioidomycosis	lungs	China & Lithuania	Kwok et al. 2009, Alčauskas 2022
		Coccidioidomycosis Osteomyelitis	right ischium and manubrium	USA	Caraway et al. 2003
Coccidioides posadasii	*Coccidioides posadasii*	Meningoencephalitis	brain	Canada	Lang et al. 2019
		Coccidioidal meningitis	brain	China	Mao et al. 2021
		Pulmonary coccidioidomycosis	lower right lung	China	Liang et al. 2018
Colletotrichum asianum	*Colletotrichum asianum*	Keratitis	eye	India	Ghorpade et al. 2022

Colletotrichum chlorophyti	*Colletotrichum chlorophyti*	Keratomycosis	eye	USA	Paniz-Mondolfi et al. 2021
Colletotrichum coccodes	*Colletotrichum coccodes*	Phaeohyphomycosis	left arm	USA	O'Quinn et al. 2001
Colletotrichum crassipes	*Colletotrichum crassipes*	Phaeohyphomycotic Cyst	right leg	Brazil	Martins Castro et al. 2001
Colletotrichum dematium	*Colletotrichum dematium*	Keratitis and endophthalmitis	eye	Czech Republic & India	Chakrabarti et al. 2008, Buchta et al. 2019, Wankhade et al. 2021
Colletotrichum gloeosporioides	*Colletotrichum gloeosporioides*	Keratitis	eye	India & Japan	Shiraishi et al. 2011, Pote et al. 2017
		Subcutaneous Hyalohyphomycosis	left forearm & elbow	Spain	Guarro et al. 1998
Colletotrichum graminicola	*Colletotrichum graminicola*	Keratitis	eye	India & USA	Ritterband et al. 1997, Yegneswaran et al. 2010
Colletotrichum siamense	*Colletotrichum siamense*	Cutaneous infection	right fourth finger	USA	Werbel et al. 2019
Colletotrichum truncatum	*Colletotrichum truncatum*	Keratitis and Endophthalmitis	eye	India	Shivaprakash et al. 2011
Conidiobolus coronatus	*Conidiobolus coronatus*	Rhinoentomophthoromycosi s	nose	India, Brazil, Switzerland, & United Arab Emirates (UAE)	Valle et al. 2001, Fischer et al. 2008, Chowdhary et al. 2010
		Disseminated infection	lungs & pulmonary vessel walls	USA	Walker et al. 1992, Deak et al. 2018
		Vaginal infection	cervix and vagina	USA	Subramanian and Sobel 2011
Conidiobolus incongruus	*Conidiobolus incongruus*	rhino-orbitocerebral entomophthoramycosis	nose & Germany	midface	Wüppenhorst et al. 2010
Conidiobolus lamprauges	*Conidiobolus lamprauges*	Disseminated infectionlungs	kidneys & spleen	Japan	Kimura et al. 2011
Coniochaeta massiliensis	*Coniochaeta massiliensis*	Skin infection	abscess of the hand	France	Kabtani et al. 2022
		Skin infection	abscess of the hand	France	Kabtani et al. 2022
Coniochaeta mutabilis	*Coniochaeta mutabilis*	Keratitis	left eye	USA	Oremosu et al. 2023, Bellanda et al. 2023
		Keratitis	left eye	USA	Bellanda et al. 2023, Oremosu et al. 2023
Coprinopsis cinerea	*Coprinopsis cinerea*	Skin and soft tissue infection	head	Germany	Correa-Martinez et al. 2018
		Pneumonia	respiratory Tract Infection	Korea	Lee et al. 2020
Cryptococcus decagattii	*Cryptococcus*	Cryptococcosis	CNS	Argentina	Berejnoi et al. 2019

	decagattii				
Cryptococcus gattii	*Cryptococcus gattii*	Cryptococcosis	lungs, central nervous system (CNS)	China	Xue et al. 2020
		Cutaneous cryptococcosis	right forearm	Brazil	Nascimento et al. 2014
Cryptococcus neoformans	*Cryptococcus neoformans*	Pulmonary infection	lungs	USA	Young et al. 1980
		acute and chronic inflammation, meningitis	cerebrospinal fluid (CSF)	USA	Williamson et al. 1996
		Cryptococcosis (Disseminated infection)	right thigh, mediastinum, skin, lungs, spine and iliac fossa	China & India	Suchitha et al. 2012, Ruan et al. 2017
		Disseminated infections	lungs	Spain	Alegre-González et al. 2021
		Osteomyelitis	right elbow	Taiwan	Liu et al. 1998
		Prosthetic valve endocarditis	brachial artery	India	Banerjee et al. 1997
	Cryptococcus neoformans var. grubii	Cellulitis	legs	China	Song et al. 2021
Cunninghamella arunalokei	*Cunninghamella arunalokei*	Chronic rhino-orbital-cerebral mucormycosis	nose and swelling in the left mid-half of the face	India	Hallur et al.2021
Cunninghamella bertholletiae	*Cunninghamella bertholletiae*	Cutaneous infection	right hip	France	Quinio et al. 2004
		Paranasal sinusitis	maxillary antrum	UK	Ng et al. 1994
		Disseminated infection	lungs, forehead, & right big toe	Taiwan	Hsieh et al. 2013
		Pneumonia/ Pulmonary infection	lungs	Germany, Greece, & USA	Rickerts et al. 2000, Garey et al. 2001, Bibashi et al. 2008
		Endocarditis and Hemorrhagic Stroke	left upper lobe of the lung, myocardium and endocardium, mitral valve	Georgia	Zhang et al. 2002
		Peritonitis	abdomen	Australia	Pimentel et al. 2006
		Nosocomial Invasive Infection	blood	Brazil	Passos et al. 2006
Curvularia australiensis	*Curvularia*	Keratomycoses	eye	India	Krizsan et al. 2015

	australiensis				
Curvularia brachyspora	*Curvularia brachyspora*	Keratitis	eye	South Africa	Marcuset al. 1992
		Cutaneous infection	thighs	Australia	Torda et al. 1997
		Cutaneous infection	thighs	Australia	Torda and Jones 1997
Curvularia clavata	*Curvularia clavata*	Cutaneous phaeohyphomycosis	left foot, face and left upper arm	China	Wang et al. 2003, Fan et al. 2009
		Invasive sinusitis and cerebritis	sinuses and brain, maxillary, frontal, & left sphenoid sinuses	USA	Ebright et al. 1999
Curvularia coimbatorensis	*Curvularia coimbatorensis*	Keratitis	eye	India	Kiss et al. 1999
Curvularia geniculata	*Curvularia geniculata*	Peritonitis	abdomen	USA	Vachharajani et al. 2005
Curvularia hawaiiensis	*Curvularia hawaiiensis*	Keratomycoses	eye	India	Krizsan et al. 2015
		Keratomycosis and Endophthalmitis	eye	India	Bashir et al. 2009
		Allergic bronchopulmonary mycosis	left main bronchus and left upper & lower lobes	India & USA	Saenz et al. 2001, Chowdhary et al. 2011
		Rhinosinusitis	Left & right nasoethmoidal polyp	India & Qatar	Taj-Aldeen et al. 2004
Curvularia inaequalis	*Curvularia inaequalis*	Peritonitis	abdomen	Australia	Pimentel et al. 2005
		Rhinosinusitis	right maxillary and ethmoid sinuses, Right frontal and sphenoid sinuses	Italy	Posteraro et al. 2010
Curvularia lunata	*Curvularia lunata*	Subcutaneous phaeohyphomycosis	left leg	Mexico	Vásquez-del-Mercado et al. 2013
		Cerebral Phaeohyphomycosis	brain	USA	Carter and Boudreaux 2004
		Endophthalmitis	left eye	India, USA	Kaushik et al. 2001, Alex et al. 2013
		Endophthalmitis	left eye	Colombia, Brazil	Berbel et al. 2011, Jaramillo and Varon 2013
		Rhinosinusitis	paranasal sinus	India, Italy	Cavanna et al. 2014, Gupta et al. 2017
		Peritonitis	abdomen	Brazil	Lopes et al. 1994

		Cutaneous infectious granuloma	nose	China	Wang et al. 2023
		Rhinosinusitis with orbital cellulitis	eye	India	Narula et al. 2020
		Paronychia and black discoloration	thumb nail	India	Kamalam et al. 1992
		Cutaneous phaeohyphomycosis	foot web	India	Rasheeduddin et al. 2017
Curvularia pallescens	*Curvularia pallescens*	Cutaneous infection	right leg, ankle region of both feet & right thigh	USA	Agrawal and Singh 1995, Berg et al. 1995
		Pulmonary and cerebral mycetoma	Lungs & brain	USA	Lampert et al. 1997
		Keratitis	eye	USA	Wilhelmus and Jones 2001
Curvularia senegalensis	*Curvularia senegalensis*	keratitis	eye	Brazil	Guarro et al. 1999
Curvularia spicifera	*Curvularia spicifera*	Keratomycoses	eye	India	Krizsan et al. 2015
		Nasal phaeohyphomycosis	nasal septum	Chile	Bay et al. 2017
		Disseminated infection	lungs, liver, brain, & neck to abdominal regions	Japan	Kobayashi et al. 2008
		Allergic fungal sinusitis	paranasal sinuses	Japan	Taguchi et al. 2007
Curvularia tamilnaduensis	*Curvularia tamilnaduensis*	Keratitis	eye	India	Kiss et al. 1999
Curvularia trifolii	*Curvularia trifolii*	Cutaneous infection	right forearm	Japan	Kiryu 1985
Curvularia warraberensis	*Curvularia warraberensis*	Invasive sinusitis	sphenoid and ethmoid sinuses	India	Samaddar et al. 2023
Cyberlindnera fabianii	*Candida fabianii*	Septicaemia	blood	Germany	Valenza et al. 2006
	Pichia fabianii	Endocarditis	aortic valve	China & Czech Republic	Hamal et al. 2008, Wu et al. 2013
	Lindnera fabianii	Fungemia	bood	France	Gabriel et al. 2012
	Cyberlindnera fabianii	Fungemia	blood	Kuwait & China	Wu et al. 2013, Al-Sweih et al. 2019
		Vulvovaginitis	vulva	Iraq	Zghair 2020
		Ventriculoperitoneal Shunt Infection	central nervous system (CNS)	USA	Baghdadi et al. 2015
		Urinary tract & Fungemia	urinary tract & blood infections	Croatia	Mlinarić-Missoni et al. 2015

Cyberlindnera jadinii	*Candida utilis*	Fungaemia	blood	Croatia, France, & India	Lukić-Grlić et al. 2011, Shivadasan et al. 2016, Gaisne et al. 2018
		Chronic urinary tract infection	urinary tract	USA	Hazen et al. 1999
		Keratitis	eye	China	Shih et al. 1999
	Cyberlindnera jadinii	Candidaemia	blood	France	Treguier et al. 2018
Debaryomyces hansenii	*Debaryomyces hansenii*	Bone infection	distal tibia	canada	Wong et al. 1982
		Extrinsic allergic alveolitis	extrinsic allergic alveolitis	Japan	Yamamoto et al. 2002
		Bone infection	distal tibia	Canada	Wong et al. 1982
		Extrinsic allergic alveolitis	extrinsic allergic alveolitis	Japan	Yamamoto et al. 2002
Dichotomopilus funicola	*Chaetomium funicola*	Chromoblastomycosis	skin on the dorsum of right hand	Western Panama	Piepenbring et al. 2007
		Chromoblastomycosis (skin lesions)	dorsum of the right hand	Panama	Piepenbring et al. 2007
		Keratitis	eye	India	Balne et al. 2012
		Eumycetoma	left foot	Sudan	Mhmoud et al. 2019
		Chromoblastomycosis	skin on the dorsum of right hand	Western Panama	Piepenbring et al. 2007
Didymella glomerata	*Phoma glomerata*	Onychomycosis	toenail	Iran	Zarei et al. 2023
		Keratitis	left eye	Korea	Jun et al. 2014
Didymella heteroderae	*Didymella heteroderae*	Keratitis	eye and contact lens	Japan	Todokoro et al. 2021
		Fungal keratitis	eye and contact lens	Japan	Todokoro et al. 2021
Diutina rugosa	*Candida rugosa*	Candidaemia, blood,	blood	India & USA	Minces et al. 2009, Behera et al. 2010
		Peritonitis	abdomen	Turkey	Kocyigit et al. 2010
Ectophoma insulana	*Phoma insulana*	Chromoblastomycosis (subcutaneous infection)	foot	Mexico	Hernández-Hernández et al. 2018
Emergomyces africanus	*Emergomyces africanus*	Disseminated Emergomycosis	skin	South Africa	Govender and Grayson 2019
Emergomyces canadensis	*Emergomyces canadensis*	Disseminated infections	blood, skin, cervix, lung, & lymph node	USA	Schwartz et al. 2018
Emergomyces orientalis	*Emergomyces orientalis*	Disseminated infections	lungs, torso, & legs	China	Wang et al. 2017
		Disseminated	lungs	Tibet	He et al. 2021

		Emergomycosis			
Emergomyces pasteurianus	*Emmonsia pasteuriana*	Disseminated infection	skin lesions (abdomen, head, nose and left thigh, neck, forehead, & arm), & lungs	China & India	Feng et al. 2015, Malik et al. 2016
		Pneumonia	lungs	Hong Kong	Chik and To 2020
		pulmonary and subcutaneous lesions	lungs & left leg	Netherlands	Gast et al. 2019
Emmonsia crescens	*Emmonsia crescens*	Disseminated Adiaspiromycosis	lungs	France	Dot et al. 2009
Epicoccum tritici	*Epicoccum tritici*	Cutaneous phaeohyphomycosis	skin	India	Sharma et al. 2021
		Cutaneous phaeohyphomycosis	skin	India	Sharma et al. 2021
Epidermophyton floccosum	*Epidermophyton floccosum*	Dermatophytosis (Tinea pedis)	toes	Iran & Nigeria	Enweani et al. 1996, Pakshir and Hashemi 2006
		Disseminated tinea	legs, groin, penis and lower abdomen	China	Qiangqiang et al. 2001
		Nonepidermolytic hereditary palmoplantar keratoderma	palms & soles	Japan	Maruyama et al. 1999
Exophiala asiatica	*Exophiala asiatica*	infection	pharynx	China	Li et al. 2009
Exophiala bergeri	*Exophiala bergeri*	Onychomycosis	toe nail	Hong Kong	Woo et al. 2013
Exophiala cancerae	*Exophiala cancerae*	Gastrointestinal tract infection	gastrointestinal tract	Hong Kong	Woo et al. 2013
Exophiala dermatitidis	*Exophiala dermatitidis*	Endophthalmitis	left eye	India	Homa et al. 2018
		Meningitis	CSF (cerebral spinal fluid)	China	Wang et al. 1991, Li et al. 2011
		Pneumonia	lungs	Germany	Kusenbach et al. 1992
Exophiala hongkongensis	*Exophiala hongkongensis*	Onychomycosis	right big toe	Hong Kong	Woo et al. 2013
Exophiala jeanselmei	*Exophiala jeanselmei*	Subcutaneous infection	right arm	Austria	Parente et al. 2011
		Fungemia	blood	Brazil	Marcio et al. 2002
		Keratitis	left eye	USA	Saeedi et al. 2013
		Finger nodule	right middle finger	Hong Kong	Woo et al. 2013

		Skin nodules	left arm & right third finger	USA	Lief et al. 2011
Exophiala lecanii-corni	*Exophiala lecanii-corni*	Chronic skin infection	Skin	Hong Kong	Woo et al. 2013
Exophiala oligosperma	*Exophiala oligosperma*	Fungemia	blood	Kuwait	Al-Obaid et al. 2006
		Cutaneous lesions	legs	USA	Rimawi et al. 2013
		Subcutaneous phaeohyphomycotic cyst	right elbow	India	Venkateshwar et al. 2014
		Onychomycosis	toe nail	Taiwan	Wen et al. 2016
Exophiala pisciphila	*Exophiala pisciphila*	Satellite papules	above the left medial malleolus	USA	Sughayer et al. 1991
		Allergic bronchopulmonary mycosis (ABPM)	lungs	USA	Kebbe and Mador 2016
Exophiala spinifera	*Exophiala spinifera*	Phaeohyphomycosis	face, chest, arms, & thighs	India	Rajendran et al. 2003
		Phaeohyphomycosis	right shin	USA	Harris et al. 2009
		Pheohyphomycosis	face, legs, arms, & upperback	India	Singal et al. 2008
		Disseminated Phaeohyphomycosis	Skin	Argentina	Negroni et al. 2004
		Chromoblastomycosis	right elbow & forearm	USA	Barba-Gómez et al. 1992
Exophiala xenobiotica	*Exophiala xenobiotica*	Peritonitis	abdomen	Hong Kong	Woo et al. 2013
Falciformispora lignatilis	*Falciformispora lignatilis*	eumycetoma	elbow	Philipines	Olenski et al. 2021
Falciformispora tompkinsii	*Leptosphaeria tompkinsii*	Mycetoma, _,	_	Mauritania	El-Ani et al. 1966
Filobasidium magnum	*Filobasidium magnum*	Otomycosis	ear canal	Iran	Aboutalebian et al. 2020
Filobasidium uniguttulatum	*Filobasidium uniguttulatum*	Meningitis	CNS	China	Pan et al. 2012
		Corneal abscess	eye	Spain	Vecilla et al. 2023
Fonsecaea monophora	*Fonsecaea monophora*	Cerebral phaeohyphomycosis	brain (left frontal lobe)	Turkey, UK, & USA	Surash et al. 2005, Koo et al. 2010, Doymaz et al. 2015
		Chromoblastomycosis	left leg, right lower leg, face, right hand, & left side of the	Brazil, China, & Cuba	Xi et al. 2009, Zhang et al. 2009, Badali et al. 2013, Daboit et al. 2013

			abdomen		
Fonsecaea nubica	*Fonsecaea nubica*	Chromoblastomycosis (subcutaneous lesions)	right back, hands and legs	Madagascar	Najafzadeh et al. 2010, You et al. 2019, Rasamoelina et al. 2020
Fonsecaea pedrosoi	*Fonsecaea pedrosoi*	Chromomycosis	left buttock	Japan	Kondo et al. 2005
		Chromoblastomycosis	right arm & left side of the abdomen	Cuba	Badali et al. 2013
		Corneal infection	eye	Brazil	Höfling-Lima et al. 2005
Fonsecaea pugnacius	*Fonsecaea pugnacius*	Disseminated Chromoblastomycosis	right arm, he left hemifacial area, & the nose, brain	Brazil	de Azevedo et al. 2015
Fusarium acutatum	*Fusarium acutatum*	Gangrenous necrosis	foot	Qatar	Taj-Aldeen et al. 2006
Fusarium dimerum	*Fusarium dimerum*	Eye infection	Eye	South Africa	Vismer et al. 2002
		Soft-tissue infection and pulmonary infection	foot & lungs	UK	Bigley et al. 2004
		Fungaemia	blood	Slovakia	Krcmery et al. 1997
		Onychomycosis	nails (hand and toes)	Sri Lanka	Ranawaka et al. 2015
		Disseminated Infection	blood & skin (legs)	France, & UK	Austen et al. 2001, Letscher-Bru et al. 2002
		Bloodstream Infection	blood	Saudi Arabia	Alshaya et al. 2021
		Burn wound infection	head, neck, hands, bilateral forearms, anterior chest, part of lower limbs, trunk, thighs and perineum and legs	Pakistan	Khalid et al. 2021
		Onychomycosis	fingers and toe nails	India	Ray et al. 2016
		Endophthalmitis	eye	France & India	Khan et al. 2012, Simon et al. 2018
		Cutaneous infection	legs feet, face and knee	Morocco & Spain	Collado et al. 2013, Sebbane et al. 2022
Fusarium napiforme	*Fusarium napiforme*	Disseminated infections	blood & skin	Brazil	de Souza et al. 2014
Fusarium oxysporum	*Fusarium oxysporum*	Fungaemia	blood	Slovakia & USA	Raad and Hachem 1995,Krcmery et al. 1997
		Onychomycosis	nails (hand and toes)	Brazil, Italy, & Sri Lanka	Romano et al. 1998, Godoy et al. 2004, Carvalho et al. 2014, Ranawaka et al. 2015
		Localized Cutaneous Infection	finger of the left hand	Italy	Romano et al. 2010
		Pneumonia	lungs	USA	Gorman et al. 2006

		Chronic infection	right ear and preauricular area	Spain	Pereiro et al. 2001
		Endophthalmitis	eye	Czech Republic	Buchta et al. 2014
		Keratitis	eye	India & Israel	Hemo et al. 1989, Vijaya 2001
		Disseminated cutaneous infections	skin (right upper leg & right elbow)	USA	Rippon et al. 1988, Albisetti et al. 2004
		Disseminated infections	pharynx, epiglottis, trachea, and oesophagus and spleen, lungs and both kidneys	Germany	Peltroche-Llacsahuanga et al. 2000
		Disseminated infections	skin, lung, blood, brain, & sinuses	Canada	Schwartz et al. 2015
Fusarium penzigii	*Fusarium penzigii*	Keratitis	right eye	Portugal	Do Carmo et al. 2016
Fusarium sacchari	*Fusarium sacchari*	Fungemia	blood	Spain	Guarro et al. 2000
Fusarium subglutinans	*Fusarium subglutinans*	Eumycetoma	left leg and in the ankle	México	Campos-Macías et al. 2013
Fusarium temperatum	*Fusarium temperatum*	Keratitis	eye	Netherlands	Al-Hatmi et al. 2014
Fusarium verticillioides	*Fusarium verticillioides*	Disseminated infections (fusariosis)	blood	Italy	Fanci et al. 2013
		Pneumonia	lungs	France	Herbrecht et al. 2004
		Erythema and swelling	fingers	USA	Palmore et al. 2010
		Mycetoma	right foot and ankle	Italy	Ajello et al. 1985
		Disseminated cutaneous infections	face and lower extremities	Taiwan	Chi and Wang 2007
		Superficial Suppurative Thrombophlebitis	veins	USA	Murray et al. 2003
Histoplasma capsulatum	*Histoplasma capsulatum*	Disseminated Histoplasmosis	blood, lungs, metatarsal bones & skin	Paraguay & Philippines	Brilhante et al. 2012, Benítez et al. 2019
		Histoplasmosis	lungs	South Africa	Pillay et al. 1997
Histoplasma capsulatum var. capsulatum	*Histoplasma capsulatum var. capsulatum*	Histoplasmosis	brain, lungs, & skin	Argentina, & USA	Wheat et al. 1992, Bracca et al. 2003
Histoplasma capsulatum var. duboisii	*Histoplasma capsulatum var. duboisii*	Histoplasmosis	brain, lungs, skin, & spine	Japan, Mali, & The Republic of Congo,	Sharmin et al. 2003, Minta et al. 2005, Boukassa et al. 2019, Amona et al. 2021

Hormographiella aspergillata	*Hormographiella aspergillata*	Pulmonary Infection	lungs	Belgium, France, & Switzerland	Lagrou et al. 2005, Godet et al. 2017, Moniot et al. 2020, Tschopp et al. 2021
		Cerebral infection	Brain (cerebral hemispheres and cerebellum)	Germany & USA	Abuali et al. 2009, Hounchonou et al. 2022
		Corneal ulcer with endophthalmitis	left eye	India	Jain et al. 2019
		Pulmonary infection	right upper lobe	Colombia	Isabel Cristina et al. 2020
		Sino-orbito-cerebral infection	brain & sinuses	France	Heiblig et al. 2015
		Necrotizing scleritis	sclera	Spain	Lamas-Francis et al. 2022
		Skin and pulmonary infection	right forearm & lungs	Austria	Bojic et al. 2013
		Disseminated infection	blood, lungs, & brain	USA	Chauhan et al. 2019
Hortaea werneckii	*Hortaea werneckii*	Tinea nigra	palms & left wrist	China & Mexico	Bonifaz et al. 2008, Mei et al. 2020
Irpex laceratus	*Emmia lacerata*	Pneumonia	respiratory Tract Infection	Korea	Lee et al. 2020
Irpex lacteus	*Irpex lacteus*	Pneumonia	respiratory Tract Infection	Korea	Lee et al. 2020
Kazachstania bovina	*Kazachstania bovina*	Bloodstream infection	blood	France	Deroche et al. 2022
Kazachstania slooffiae	*Kazachstania slooffiae*	Pleural infection	lungs	France	Mercier et al. 2021
		Esophagus infection	esophagus	Spain	Gallotti et al. 2023
Knufia epidermidis	*Coniosporium epidermidis*	Superficial and cutaneous infection	axilla, toes, & toe webs	China &Denmark	Li et al. 2008
Lasionectriopsis pteridii	*Acremonium pteridii*	Dermatitis	nails	USA	Perdomo et al. 2011
Lichtheimia corymbifera	*Lichtheimia corymbifera*	Subcutaneous infection	right leg	Spain	Blazquez et al. 2010
		Pulmonary infection (pulmonary zygomycosis)	lungs	Lithuania	Kleinotiene et al. 2013
		Pulmonary infection	lungs	Mexico	Fernández-García et al. 2021
		Otomycosis	ear	India	Vyas and Shah 2011
		Disseminated mucormycosis	liver, lungs, & spleen	France	Grossi et al. 2019
		Subcutaneous mucormycosis	right foot	Morocco	Razouk et al. 2012
Lichtheimia ornata	*Lichtheimia ornata*	Rhinocerebral mucormycosis	brain	China	Pan et al. 2020
Lichtheimia ramosa	*Lichtheimia ramosa*	Intestinal mycosis	colon	Japan	Kaneko et al. 2018
		Mucormycosis (mycotic	lungs	Spain	Mouronte-Roibás et al. 2016

		thromboses)			
		Cutaneous mucormycosis	burn wounds	India	Kaur et al. 2014
		Wound infection	left lumbar region & left foot,	Greece & India	Bibashi et al. 2013, Neelaveni et al. 2017
		Pulmonary mucormycosis	lungs	Turkey	Kutlu et al. 2014
		Mucormycosis	nasal cavity	Tunisia	Houaida et al. 2022
Lomentospora prolificans	*Scedosporium prolifican*	Disseminated infections	blood, lungs, skin, Kidney, & CNS	Spain	Alvarez et al. 1995, Husain et al. 2005
		Endophthalmitis	eye	USA	Vagefi et al. 2005
		Fungemia	blood	Spain	Simarro et al. 2001
		CNS infection	right sylvian fissure	USA	Bhat et al. 2007
Madurella mycetomatis	*Madurella mycetomatis*	Mycetoma	left ankle, hand, gluteal region, foot & knee	Saudi Arabia, Sri Lanka, & Sudan	Fahal et al. 2011, Mufti and Aljhdali 2015, Sigera et al. 2020
Madurella mycetomatis	*Madurella mycetomatis*	Eumycetoma	axilla	India	Subhashini et al. 2019
Malassezia furfur	*Malassezia furfur*	Fungaemia	blood	Hong Kong & USA	Barber et al. 1993, Schleman et al. 2000, Chu and Lai 2002
		Proximal onychomycosis	proximal regions of right hand nails, & toe nails	Iran & Turkey	Kessler et al. 2002, Ertam et al. 2007, Zareei et al. 2013
		Pustulosis	face, neck, & scalp	France	Rapelanoro et al. 1996
Malassezia pachydermatis	*Malassezia pachydermatis*	Fungemia	blood	Korea, Kuwait, Singapore, Taiwan	Al-Sweih et al. 2014, Choudhury and Marte 2014, Lee et al. 2019, Huang et al. 2020
Malassezia sp.	*Malassezia sp.*	Folliculitis	skin (face, neck and upper trunk)	Denmark & Tunisia	Anane et al. 2013, Andersen et al. 2018, Li et al. 2022
Malassezia sympodialis	*Malassezia sympodialis*	Malignant otitis externa	ear (external auditory canal)	Australia	Chai et al. 2000
		Cutaneous infection	back, chest, & neck	USA	Desai et al. 2011
		Pityriasis versicolor circinata	trunk & upper extremities	Brazil	Framil et al. 2010
		Fungemia and interstitial lung compromise	lungs & Blood	Argentina	Aguirre et al. 2015
Megasporoporia setulosa	*Megasporoporia setulosa*	Subcutaneous mycosis	left foot	India	Samaddar et al. 2023b
Meyerozyma guilliermondii	*Meyerozyma guilliermondii*	Bloodstream infections	bood	Brazil & Turkey	Gřler et al. 2017, Chaves et al 2021

Microascus cinereus	*Microascus cinereus*	Brain abscess	brain	India & USA	Baddley et al. 2000, Malik et al. 2020
		Suppurative Cutaneous Granulomata	chest, back, & arm	USA	Marques et al. 1995
		Onychomycosis	big toe of the left foot	Italy	Tullio et al. 2010
Microascus cirrosus	*Microascus cirrosus*	Disseminated fungal infection & with Fungemia	pleura, lungs & blood	France	Krisher et al. 1995, Miossec et al. 2011
		Cutaneous infection	left ankle	China	Gao et al. 2018
		Pulmonary infection	lungs	China	Liu et al. 2021, Cheng et al. 2023
Microascus ennothomasiorum	*Microascus ennothomasiorum*	Subcutaneous infectio	right thumb	Germany	Brasch et al. 2018
Microascus gracilis	*Microascus gracilis*	Scopulariopsis	lungs	China	Huang et al. 2019
		Disseminated infection	pleura, brain, heart, & lungs	USA	Ding et al. 2020
Microascus paisii	*Scopulariopsis brumptii*	Onychomycosis	nails (left hand)	India	Naidu et al. 1991
Microascus trigonosporus	*Microascus trigonosporus*	Pulmonary Infection	lungs	USA	Schoeppler et al. 2015
Microdochium nivale	*Fusarium nivale*	Corneal mycosis	eye	Poland	Perz 1966
Microsphaeropsis arundinis	*Microsphaeropsis arundinis*	Skin and soft tissue infection	skin and soft tissues	Australia	Crawford et al. 2015
		Skin and soft tissue infection	skin and soft tissues	Australia	Crawford et al. 2015
Microsphaeropsis olivacea	*Microsphaeropsis olivacea*	Skin infection	skin	Espanya	Guarro et al. 1999
		Keratitis and consecutive endophthalmitis	aqueous humor of eye	USA	Shah et al. 2001
		Skin infection	skin	Espanya	Guarro et al. 1999
		Keratitis and consecutive endophthalmitis	aqueous humor of eye	USA	Shah et al. 2001
Microsporum audouinii	*Microsporum audouinii*	Tinea capitis	scalp	Nigeria & Spain	Enweani et al. 1996, Rezusta et al. 2011
Microsporum canis	*Microsporum canis*	Tinea corporis	skin (leg)	Italy	Pasquetti et al. 2017
		Pseudomycetoma,	left knee, scalp,	Brazil & USA	Berg et al. 2007, Ruiz et al. 2020
		Kerion Cels	occipital region of the scalp	Croatia	Gorgievska-Sukarovska et al. 2017
Microsporum	*Microsporum*	skin lesions circinate herpes	hand	Tunisia	Neji et al. 2009

ferrugineum	*ferrugineum*				
		Tinea capitis	scalp	Thailand	Wisuthsarewong et al. 1996
		Deep dermatophytosis	head	China	Zhang et al. 2019
Mucor circinelloides	*Mucor circinelloides*	Fungemia and cutaneous infection	blood & right hand	Turkey	Dizbay et al. 2009
		Fungemia	blood	USA	Arroyo et al. 2016
		Cutaneous infection	right leg & forearm	Australia & Canada	Chandra and Woodgyer 2002, Iwen et al. 2007
		Invasive Maxillofacial Zygomycosis	right maxilla	Kuwait	Khan et al. 2009
		Rhinocerebral mucormycosis	sinuses, nasal passages, oral cavity, & brain	Australia	Lazar et al. 2014
Mucor irregularis	*Mucor irregularis*	Disseminated infection	nose, lips, eyelids, & the central face	China	Li et al. 2012
		Cutaneous Mucormycosis	inner canthus, right lower leg & right hand	China, & Japan	Kang et al. 2014, Yamaguchi et al. 2015, Xia et al. 2015, Liang et al. 2018, Tang et al. 2021
		Cutaneous, Rhinofacial, and Pulmonary Mucormycosis	nasal septum, paranasal sinuslungs, & lungs	China	Xia et al. 2013
		Chronic Rhinofacial Mucormycosis	nasal septum	India	Hemashettar et al. 2011
Myrothecium gramineum	*Myrothecium gramineum*	Keratitis	eye	India	Rameshkumar et al. 2019
Naganishia albida	*Cryptococcus albidus*	Cryptococcaemia	CNS	Greece	Kordossis et al. 1998
		Fungemia	blood	USA	Choe et al. 2020
		Pneumonia	lungs	Turkey	Burnik et al. 2007
	Naganishia albida	Superficial cutaneous infection	axilla	Iran	Gharehbolagh et al. 2017
Naganishia diffluens	*Naganishia diffluens*	Tinea cruris	genital, pubic, perineal, and perianal skin	China	Yang et al.2023
		Tinea Cruris	genital, pubic, perineal, and perianal skin	China	Yang et al.2023
Naganishia friedmannii	*Cryptococcus friedmannii*	Onychomycosis	Toe nail	Iran	Ekhtiari et al. 2017

Nakaseomyces glabratus	*Candida glabrata*	Vulvovaginal candidiasis	vagina	USA	Geiger et al. 1995
		Fungemia	blood	USA	Bodey et al. 2002, Lin et al. 2005
		Endocarditis	ventricular endocardium	France	Roger et al. 2000
Nannizzia aenigmatica	*Microsporum aenigmaticum*	Tinea corporis	right dorsal wrist	Czech Republic	Hubka et al. 2014
Nannizzia fulva	*Nannizzia fulva*	Tinea Capiti	scalp	Iraq	Gharib et al. 2022
Nannizzia gypsea	*Microsporum gypseum*	Dermatophytosis	popliteal fossa, knee, gluteus, & face	Brazil	Souza et al. 2016
		Onychomycosis and Tinea Corporis	fingernail	USA	Fike et al. 2018
		Tinea incognito	face & neck	Italy	Polilli et al. 2011
		Kerion Celsi	scalp	Mexico	Torres-Guerrero et al. 2015
	Nannizzia gypsea	Tinea corporis	right upper quadrant & right forearm	Japan & Madagascar	Kobayashi et al. 2018, Soankasina et al. 2018
		Kerion Celsi	occipital region and neck	Japan	Toyosawa et al. 2022
	Arthroderma gypseum	Kerion Celsi	scalp	Japan	Iwasawa et al. 2009, 2012
Nannizzia incurvata	*Nannizzia incurvata*	Favus & tinea corporis	right hip, scalp, & face	Cambodia & Vietnam	Uhrlaß et al. 2021
		Scrotal tinea	scrotum	China	Qiu et al. 2022
Nannizzia nana	*Nannizzia nana*	dermatomycoses (skin infections)	neck, feet, scalp, face, trunk, arms, & nails	Mexico & Poland	Bonifaz et al. 2019, Gnat et al. 2020
		Ringworm	right arm	Spain	Pendones-Ulerio et al. 2023
Nannizzia perplicata	*Nannizzia perplicata*	Tinea corporis	wrist and arm	UK	Borman et al. 2019
	Microsporum persicolor	Tinea corporis	left region of the abdomen	Iran	Naseri et al. 2012
		Cutaneous infections	fingers and left elbow	Germany	Chen et al. 2012
Nannizzia polymorpha	*Nannizzia polymorpha*	Tinea capitis & tinea manuum	right hand & scalp	Taiwan	Sun et al. 2023
Nannizzia praecox	*Microsporum praecox*	Dermatophytosis	right external malleolus	France	Alanio et al. 2011
		Tinea capitis	scalp	USA	Padhye et al. 1989

Neoconidiobolus pachyzygosporus	*Conidiobolus pachyzygosporus*	Pulmonary infection	lungs	Switzerland	Stavropoulou et al. 2022
		Conidiobolomycosis with dysphagia	nasopharyngeal wall	India	Purohit et al. 2021
Neocosmospora cyanescens	*Phialophora cyanescen*	Mycetoma	right foot	Netherlands	De Hoog et al. 1993
Neocosmospora falciformis	*Fusarium falciforme*	Vertebral Abscess and Osteomyelitis	thoracolumbar spine	USA	Edupuganti et al. 2011
		Disseminated angioinvasive infection	blood & both lower legs	South Korea	Yun et al. 2007
	Acremonium falciforme	Eumycetoma	left foot	China	Xiujiao et al. 2012
		Mycetoma	hands and forearms	USA	Van Etta et al. 1983
Neocosmospora keratoplastica	*Fusarium keratoplasticum*	Eumycetoma	legs	Mexico	Al-Hatmi et al. 2017
Neocosmospora lichenicola	*Cylindrocarpon lichenicola*	Cutaneous Infection	right hand, webspaces of both hands	Costa Rica, India, & USA	Iwen et al. 2000, Champa et al. 2013, Lizano-Calvo et al. 2013
		Mycetoma	foot	Canada & Israel	Zoutman et al. 1991, Chazan et al. 2004
		Disseminated	blood, lungs, nails and the skin of toes	Belgium	James et al. 1997, Rodríguez-Villalobos et al. 2003
		Keratomycosis	eye	Argentina, India, Nigeria, & UK	Mangiaterra et al. 2001, Mitra et al. 2009, Suchitra et al. 2020, Irek et al. 2017, Halim et al. 2021
		Onychomycosis	nail of the middle finger of the right hand	Costa Rica	Lizano-Calvo et al. 2013
		Athlete's foot (tinea pedis)	inter-toes areas	Senegal	Diongue et al. 2016
		Peritonitis	abdomen	India	Sharma et al. 1998
Neocosmospora petroliphila	*Fusarium petroliphilum*	Disseminated infection	skin (legs) & blood	Turkey	Ersal et al. 2015
		Fungaemia	blood	France	Dananché et al. 2015
Neocosmospora pseudensiformis	*Fusarium pseudensiforme*	Eumycetoma	legs	Mexico	Al-Hatmi et al. 2017
Neocosmospora solani	*Fusarium solani*	Fungaemia	blood	China, Slovakia, Ireland, Italy, USA	Lodato et al. 2006, Krcmery et al. 1997, O' Connell et al. 2011, Colombo et al. 2013, Esnakula et al. 2013, Kang et al. 2013
		Disseminated infections	Skin (Right elbow),	Canada	Schwartz et al. 2015

			lung, liver, brain		
		Disseminated infection & fungemia	skin lesions (face, neck, and trunk), blood	Italy	Venditti et al. 1988
		Keratomycosis	eye	India	Kulkarni et al. 2017
		Onychomycosis, Cutaneous infections	first toe & toenails	Brazil, Switzerland	Godoy et al. 2004, Jossi et al. 2010
		Mycetoma with osteolytic lesions	hands & foot	Brazil, France	Tomimori-Yamashita et al. 2002, Year et al. 2003
		Cutaneous lesion and swelling	right buttock	India	Katkar et al. 2011
		Burn Wound Infection	face, scalp, chest, abdomen, & back	Vietnam	Tram et al. 2020
		Cutaneous infection & erythematous nodule	left lower leg & right upper arm	UK	Cooke et al. 2009
		Quasi-Invasive Infection	left heel & right forefeet,	India	Kudur et al. 2013
Neocucurbitaria cava	*Phoma cava*	Subcutaneous pheohyphomycosis	skin of the right hand	Brazil	Zaitz et al 1997
		subcutaneous pheohyphomycosis	skin of the right hand	Brazil	Zaitz et al 1997
Ovatospora brasiliensis	*Chaetomium brasiliense*	Phaeohyphomycosis	ear	Czech Republic	Hubka et al 2003
		Phaeohyphomycosis	ear	Czech Republic	Hubka et al 2003
Paecilomyces lilacinus	*Paecilomyces lilacinus*	Keratitis	eye	Brazil, China, & USA	Pastor et al. 2006, Ford et al. 20058, Wu et al. 2010
		Endophthalmitis	eye	Australia, Switzerland, Spain, & USA	Domniz et al. 2001, Pintor et al. 2001, Scott et al. 2001, Garbino et al. 2002
		Pulmonary infection	lungs	USA	Liu et al. 1998
		Cutaneous infection	legs, right knee	Switzerland, & USA	Orth et al. 1996, Hecker et al. 1997, Hall et al. 2004
		Fungaemia	blood	USA	Bernacer et al. 1992
		Vaginitis	vagina	USA	Carey et al. 2003
		Sinusitis	sinuses	India	Nayak et al. 2000
		Osteomyelitis	great toe	USA	Gompf et al. 1999
		Onychomycosis	right great toe nail	UK	Fletcher et al. 1998
Paecilomyces variotii	*Paecilomyces variotii*	Pneumonia	lungs	USA	Feldman et al. 2016
		Rhinosinusitis	nose and paranasal	India	Swami et al. 2016

			sinuse		
		Fungemia	blood	France	Salle et al. 2005, Bellanger et al. 2017
		Mycetoma	lungs	Portugal	Marques et al. 2019
		Chronic suppurative otitis	left ear	India	Dhindsa et al. 1995
		Wound Infection	sternum	China	Lee et al. 2002
		Endophthalmitis	eye	Finland & India	Tarkk et al. 2004, Anita et al. 2010
		Peritonitis	abdomen	Italy & Turkey	Rinaldi et al. 2000, Polat et al. 2015
Papiliotrema flavescens	*Papiliotrema flavescens*	Pulmonary infection	lung	China	Zhang et al.2022
		Pulmonary fungal infection	lung	China	Zhang et al.2022
Papiliotrema laurentii	*Papiliotrema laurentii*	Fungemia/Catheter-related infection	Blood	Brazil & Turkey	Londero et al. 2019, Aydın et al. 2023
Paracoccidioides americana	*Paracoccidioides americana*	Paracoccidioidomycosis/ Disseminated infection	_	Brazil	De Macedo et al. 2019
Paracoccidioides brasiliensis	*Paracoccidioides brasiliensis*	Osteomyelitis	leg (calcaneous bone)	Brazil	Nogueira et al. 2001
		Disseminated infection	mucosal surface & respiratory tract	Brazil	de Macedo et al. 2016
		Disseminated infection	skin (face, lower right hemithorax, right cervical chain and ribs), lungs & brain	Brazil	Peçanha et al. 2021
Paracoccidioides lutzii	*Paracoccidioides lutzii*	Fungemia	blood	Brazil	Hahn et al. 2014
		Pulmonary infection	upper lobes of the lungs	Brazil	Marques-da-Silva et al. 2012
Phaeoacremonium inflatipes	*Phaeoacremonium inflatipes*	Phaeohyphomycosis	left foot	USA	Padhye et al 1998
		Cystic plantar phaeohyphomycosis	left forefoot	India	Sandhu et al. 2022
		Phaeohyphomycosis	left foot	USA	Padhye et al 1998
		cystic plantar phaeohyphomycosis	left forefoot	India	Sandhu et al. 2022
Phaeoacremonium parasiticum	*Phialophora parasitica*	Eumycetoma	right foot	UK	Hood et al. 1997
Phellinus mori	*Phellinus mori*	Subcutaneous infection	right chest	Japan	Shigemura et al. 2015

Phellinus undulatus	*Phellinus undulatus*	Soft tissue infection	right knee	New Zealand	Williamson et al. 2011
Phialemonium atrogriseum	*Acremonium atrogriseum*	Keratitis	left eye	USA	Read et al. 2000
Phialemonium obovatum	*Phialemonium obovatum*	Endocarditis	septal and posterior leaflets of the tricuspid valve	USA	Gavin et al.2002
		Lung infection	blood	USA	Scott et al. 2005
		cutaneous phaeohyphomycosis	skin	india	Sharma et al. 2021
		Endocarditis	septal and posterior leaflets of the tricuspid valve	USA	Gavin et al.2002
		Lung infection	blood	USA	Scott et al. 2005
		cutaneous phaeohyphomycosis	skin	india	Sharma et al. 2021
Phialophora americana	*Phialophora americana*	Phaeohyphomycosis	face	China	Huang et al. 2019
Phialophora chinensis	*Phialophora chinensis*	Keratitis	right eye	USA	Ply et al. 2023
Phialophora europaea	*Phialophora europaea*	Cutaneous & nail infections	Legs & nails	Netherlands	De Hoog et al. 2000
Phialophora richardsiae	*Phialophora richardsiae*	Chromoblastomycosis	right patella & right shin	Canada & Korea	Jumaa et al. 1995, Levenstadt et al. 2012
Phialophora verrucosa	*Phialophora verrucosa*	Phaeohyphomycosis	face & gluteal region	China & Japan	Ohira et al. 2002, Tong et al. 2013
		Chromoblastomycosis	feet & legs	Korea	Park et al. 2005
Phoma herbarum	*Phoma herbarum*	Onychomycosis	Skin & nails	Italy	Tullio et al. 2010
Pichia fermentans	*Candida lambica*	Fungemia	bood	Belgium	Vervaeke et al. 2008
		Polyarthritis	joints	USA	Trowbridge et al. 1999
Pichia kudriavzevii	*Candida krusei*	Vaginitis	vagina	USA	Singh et al. 2002
		Septic arthritis	left and right knees	China & USA	Guyen and Penn 1987, Wang et al. 2007, Lu et al. 2012
		Fungemia	blood	USA	Abbas et al. 2000, Lin et al. 2005
		Sternal osteomyelitis	sternum	Greece	Petrikkos et al. 2001
		Spondylodiscitis	vertebrae	Spain	Pemán et al. 2006
		Candidemia	blood	India	Kaur et al. 2020
Piedraia hortae	*Piedraia hortae*	black piedra	scalp	Spain & Sweden	Gip 1994, Piquero-Casals et al. 2023
Pleurostoma hongkongense	*Pleurostoma hongkongense*	Phaeohyphomycosis	liver	Hong Kong	Tsang et al. 2021

Pleurostoma ootheca	*Pleurostoma ootheca*	Phaeohyphomycosis	left ankle	France	Amazan et al. 2014
Pneumocystis carinii	*Pneumocystis carinii*	Acute diffuse pneumonia	Bronchoalveolar lavage fluid	Zimbabwe	Malin et al. 1995
		Acute diffuse pneumonia	Bronchoalveolar lavage fluid	Zimbabwe	Malin et al. 1995
Pneumocystis jirovecii	*Pneumocystis jirovecii*	COVID-19 and fungal Coinfection	respiratory tract	USA	Menon et al. 2020
		Pneumonia	lungs	France, UK & USA	De Castro et al. 2010, Cawcutt et al. 2014, Chen et al. 2020, Coleman et al. 2020
Porostereum spadiceum	*Porostereum spadiceum*	Pneumonia	respiratory Tract Infection	Korea	Lee et al. 2020
Rhinocladiella aquaspersa	*Rhinocladiella aquaspersa*	Chromoblastomycosis (cutaneous infection)	left hand & right leg	Brazil, Guatemala, & México	González et al. 2013, Campos et al. 2018, Porras-López et al. 2019
Rhinocladiella basitona	*Rhinocladiella basitona*	Phaeohyphomycosis	face	China	Cai et al. 2013
		Endophthalmitis	right eye	China	Liu et al. 2015
Rhinocladiella mackenziei	*Rhinocladiella mackenziei*	Cerebral phaeohyphomycosis	brain	Iran, Saudi Arabia, & Turkey	Mohammadi et al. 2018, Al-Tawfiq and Boukhamseen 2011, Didehdar et al. 2015, Kucukkaya et al. 2023
		Disseminated infection	abdomen, lungs, & brain	Kuwait	Al Otaibi et al. 2021
Rhinocladiella similis	*Rhinocladiella similis*	Chromoblastomycosis	left palm, chest, & foot	Brazil, Spain	Heidrich et al. 2017, de Andrade et al. 2020
Rhizomucor pusillus	*Rhizomucor pusillus*	Disseminated infection	brain, myocardium, liver, pancreas, spleen, colon, & both kidneys	Germany	Schober et al. 2021
		Fungemia	blood	USA	Bard et al. 2014
		Pulmonary infection	lungs (left upper lobe)	Australia	Ma et al. 2001
		Sinus-Orbital infection	right eye & sinuses	Canada	Iwen et al. 2005
		Cerebral infection	left temporal lobe brain	USA	Farid et al. 2017
		Pulmonary, rhino-orbital and cutaneous infection	sinuses, lungs, left neck, & left shoulder	Germany	Hadaschik et al. 2012
Rhizopus arrhizus	*Rhizopus arrhizus*	Rhinosinusitis mucormycosis	necrotic eschars on the palate	Iran	Tabarsi et al. 2021

		Rhino-orbital-cerebral mycosis	midline face ulcers	China	Li et al. 2021
		Primary cerebral zygomycosis	fluid aspirated from a brain mass	Italy	Oliveri et al. 1988
		COVID-19-associated rhinosinusitis mucormycosis	necrotic eschars on the palate	Iran	Tabarsi et al. 2021
		Rhino-Orbital-Cerebral Mycosis	midline face ulcers	China	Li et al. 2021
		primary cerebral zygomycosis	fluid aspirated from a brain mass	Italy	Oliveri et al. 1988
Rhizopus microsporus	*Rhizopus microsporus*	Pulmonary infection	lungs	Mexico	Fernández-García et al. 2021
Saksenaea erythrospora	*Saksenaea erythrospora*	Disseminated Infection	burn wound infections (head, arms, & legs), left orbit	USA	Hospenthal et al. 2011
		Necrotizing skin and soft tissue infections	left shoulder, left axillary region, & right gluteal region	India	Chander et al. 2017
		Skin and soft tissue infection	right breast	Colombia	Rodríguez et al. 2016
		Necrotizing infection	medial canthal region of her right eye	India	Mukherjee and Kundu 2018
		Rhinosinusitis	eye, rightsided facial palsy, & neck	India	Tendolkar et al. 2015
		Periocular Cutaneous infection	right medial canthus & upper cheek	Thailand	Putthirangsiwong et al. 2019
Saksenaea vasiformis	*Saksenaea vasiformis*	Cutaneous infection	abdomen, left cheek, right elbow, & thighs	Greece & India	Chakrabarti et al. 1997, Padmaja et al. 2006, Gkegkes et al. 2019
		Cutaneous infection	right gluteal region	Ecuador & India	Vega et al. 2006, Kaushik et al. 2012
		Subcutaneous infection	anterior abdominal region & left cheek	India	Padmaja et al. 2006, Baradkar and Kumar 2009
		Disseminated infection	mitral valve, lungs, & skin	Australia	Solano et al. 2000
		Osteomyelitis	medial, lateral, & anterior distal tibia	USA	Pierce et al. 1987
Sarocladium kiliense	*Acremonium kiliense*	Keratitis	eye	USA	Weissgold et al. 1998

		Fungemia and lung infection	blood & lungs	Brazil	Júnior et al. 2013
		Fungemia	blood	Greece & USA	Ioakimidou et al. 2013, Perdomo et al. 2011
		Hilar pneumonia	right lung	Brazil	Pastorino et al. 2005
		Peritonitis	abdomen	Brazil	Lopes et al. 1995
		Mycetoma	left fascio-maxillary area	India	Agarwal et al. 2011
		Peritonitis	abdomen	Turkey	Koç et al. 1998, Sener et al. 2008
		Skin infection	legs	Greece & Turkey	Miyakis et al. 2006, Hilmioglu et al. 2015
		Cutaneous Hyalohyphomycosis	right foot	India	Sharma et al. 2013
		Fungaemia	blood	France	Hitoto et al. 2010
		Disseminated infection	gastrointestinal system, skin, & blood	USA	Schell et al. 1996
		Erythema	right cheek	Turkey	Anadolu et al. 2001
Scedosporium apiospermum	*Scedosporium apiospermum*	Disseminated infections,	CNS and cutaneous infections	Belgium & Spain	Montejo et al. 2002, Symoens et al. 2006
		Septic Arthritis	right knee	Spain	Tirado-Miranda et al. 2001
		Keratitis/sclerokeratitis	eye	India & USA	Ramakrishnan et al. 2018, Wu et al. 2002
		Cutaneous infection	right hand	UK	Uenotsuchi et al. 2005
		Disseminated infections	blood, lungs, skin, & CNS	Spain	Husain et al. 2005
Scedosporium boydii	*Scedosporium boydii*	Keratitis	eye	India & USA	Wu et al. 2002, Ramakrishnan et al. 2018
		Pulmonary Infections	lungs	France	Jabado et al. 1998
Schizophyllum commune	*Schizophyllum commune*	Allergic Broncho-Pulmonary Mycosis (ABPM) and pulmonary fungal ball	lungs	India & Japan	Kamei et al. 1994, Ogawa et al. 2012, Chowdhary et al. 2013
		Chronic non-invasive rhinosinusitis	left sphenoid sinus and posterior ethmoid sinus	Italy	Cavanna et al. 2019
		Keratitis	eye	India	Saha et al. 2013
		Pneumonia	lungs	Korea	Kim et al. 2022
		Maxillary Sinusitis	left frontal and maxillary sinuses	Canada, Colombia, & India	Sigler et al. 1997, 1999, Castro et al. 2010, Swain et al. 2011
		Sinusitis and frontal brain abscess	right frontal brain & bilateral sinusitis	Austria	Hoenigl et al. 2013

		Sinusitis	paranasal sinuses	Japan	Taguchi et al. 2007
Sporothrix brasiliensis	*Sporothrix brasiliensis*	Dacryocystitis	lower corner of the left eye	Brazil	Marques de Macedo et al. 2015
		Disseminated infection	mitral valve, eyes, & lymph nodes	Brazil	Silva-Vergara et al. 2012
		disseminated cutaneous sporotrichosis	face and hands	Brazil	Valeriano et al. 2020
		Invasive Sinusitis	paranasal and maxillary sinuses	Brazil	Araújo et al. 2021
Sporothrix globosa	*Sporothrix globosa*	Sporotrichosis scaly erythematous lesion	left arm	Brazil	Gompertz et al. 2016
		Sporotrichosis Skin lesios	hands	Japan	Watanabe et al. 2016
		Cutaneous Sporotrichosis	right upper eyelid	China	Liu et al. 2021
Sporothrix schenckii	*Sporothrix schenckii*	Monoarthritis	right knee	Brazil & USA	Appenzeller et al. 2006, Barbaryan et al. 2018
		Subcutaneous infection	legs	Turkey	Koç et al. 2001
Syncephalastrum racemosum	*Syncephalastrum racemosum*	Pneumonia	lungs	Mexico	Rodríguez-Gutiérrez et al. 2015
		Gastrointestinal infection	gastrointestinal tract	India	Raju et al. 2020
		Subcutaneous mucormycosis	anterior chest wall in the left side	India	Mangaraj et al. 2014
		Onychomycosis	left great toe nail, right great toenail	India & Serbia	Pavlovic and Bulajic 2006, Baby et al. 2015, Jindal et al. 2016
		Intraabdominal Zygomycosis	abdomen	Australia	Schlebusch and Looke et al. 2005
Talaromyces amestolkiae	*Talaromyces amestolkiae*	Lymphadenitis	inguinal lymph node	Taiwan	Wang et al. 2023
		Pulmonary infection	lungs	Mexico	Villanueva-Lozano et al. 2017
Talaromyces marneffei	*Talaromyces marneffei*	Talaromycosis	intestine, oropharynx and larynx	China & Thailand	Pan et al. 2020, Wongkamhla et al. 2019
Talaromyces purpurogenus	*Talaromyces purpurogenus*	Pulmonary infection	lungs	Turkey	Atalay et al. 2016
		Otomycosis	ear canal	Iran	Aboutalebian et al. 2020
Thyridium curvatum	*Phialemonium curvatum*	sporadic infections	bolld and intervertebral disc-space aspirate	Spain	Rivero et al. 2009
		Endophthalmitis	eye	Israel, Norway	Zayit-Soudry et al. 2005, Navaratnam et al.2022

		sporadic infections	bolld and intervertebral disc-space aspirate	Spain	Rivero et al. 2009
		Endophthalmitis	eye	Israel, Norway	Zayit-Soudry et al. 2005, Navaratnam et al.2022
Tintelnotia destructans	*Tintelnotia destructans*	Keratitis	right eye	Belgium & Switzerland	Roels et al. 2020, Kaufmann et al. 2021
		Cornea and nail infections	eye and nails	Germany	Ahmed et al. 2017
Trematosphaeria grisea	*Madurella grisea*	Mycetoma	left wrist	India	Ankad et al. 2019
Trichoderma atroviride	*Trichoderma atroviride*	Ischemic necrosis	liver	France	Ranque et al. 2008
Trichoderma citrinoviride	*Trichoderma citrinoviride*	Pneumonia	right lung	Lithuania	Kviliute et al. 2008
Trichoderma harzianum	*Trichoderma harzianum*	Disseminated infections	blood, brain, lungs, skin, sputum, & throat	Spain & Turkey	Guarro et al. 1999, Kantarcioğlu et al. 2009
Trichoderma koningii	*Trichoderma koningii*	Peritonitis	abdomen	Spain	Ragnaud et al. 1984, Campos-Herrero et al. 1996
Trichoderma longibrachiatum	*Trichoderma longibrachiatum*	Skin graft infection	upper right extremity	Czech Republic	Lipový et al. 2021
		Skin infection	Skin	Tunisia	Trabelsi et al. 2010
		Invasive pulmonary infection	right pulmonary vessels	France	Sautour et al. 2018
		Otitis Externa	right eardrum and a partial anterior perforation on the left side	France	Hennequin et al. 2000
		Necrotizing stomatitis	upper left gingiva	Japan	Myoken et al. 2002
		Chronic sinusitis	paranasal sinuses	Canada	Tang et al. 2003
		Endocarditis	Cardiac Implantable Electronic Device)-Associated	Italy	Tascini et al. 2016
Trichoderma pseudokoningii	*Trichoderma pseudokoningii*	Disseminated infection,	lungs, brain, heart, & stomach	France	Gautheret et al. 1995
		Peritonitis	abdomen	France	Rota et al. 2000
Trichoderma viride	*Trichoderma viride*	Pulmonary infection	right upper lobe lung	Spain	De Miguel et al. 2005
		Perihepatic hematoma	peritoneal fluid	Belgium	Jacobs et al. 1992

Trichophyton benhamiae	*Arthroderma benhamiae*	Tinea corporis	left medial thigh, right ilium, & lumbar region	Brazil & Japan	de Freitas et al. 2019, Nakamura et al. 2020
Trichophyton mentagrophytes	*Trichophyton mentagrophytes*	Superficial dermatophytosis	trunk, groins, face and foot	India	Nenoff et al. 2019
		Dermatophytosis	left breast, arms, face, groin, & ears	Iran & Nigeria	Enweani et al. 1996, Pakshir and Hashemi 2006, Fattahi et al. 2021
		Kerion Celsi	scalp	Korea & Netherlands	Jang et al. 2002, Jaspers et al. 2011
		Tinea Faciei	nostrils	Japan	Kimura et al. 2015
		Tinea corporis	left lateral wall of the chest & left shoulder	India	Raonand Datta 2013
		Kerion	vulva	Canada	Bougrine et al. 2015
		Majocchi's granuloma	vulva	Korea	Chang et al. 2005
	Trichophyton mentagrophytes var. erinacei	Tinea manuum	left hand	Korea	Rhee et al. 2009
	Trichophyton mentagrophytes var. erinacei	Tinea faciei	nose	Chile	Concha et al. 2012
	Arthroderma vanbreuseghemii	Kerion & tinea corporis	scalp, face, & trunk	China	Zhang et al. 2009
		Tinea Faciei	Face (left cheek & nostrils)	China & Japan	Noguchi et al. 2010, Kang et al. 2013, Su-yang et al. 2016
Trichophyton rubrum	*Trichophyton rubrum*	Chronic dermatophytosis	nails, hands, feets, trunk, legs, trunk, & buttocks	Brazil	Sadahiro et al. 2004, Sousa et al. 2015
		Deep dermatophytosis	trunk, ear, scalp, eft groin and scrotum area, ubmandibular area, neck, & chest	China	Gong et al. 2007, Su et al. 2017, Huang et al. 2019
		Dermatophytosis	scalp and skin	Iran & Nigeria	Enweani et al. 1996, Pakshir and Hashemi 2006
		Onychomycosis	toenails	USA	Ploysangam et al. 1997
		Majocchi's granuloma & deep skin Infection	submandibular area, neck, and chest	China	Su et al. 2017
		Deep skin Infection (multiple subcutaneous nodules)	both legs	Israel	Nir-Paz et al. 2003

Trichophyton schoenleinii	*Trichophyton schoenleinii*	Pseudomycetoma	scalp	France & USA	Botterel et al. 2001, Castro-Echeverry et al. 2017
		Tinea capitis	scalp	Italy	Romano et al. 2002
Trichophyton tonsurans	*Trichophyton tonsurans*	Tinea capitis/Kerion Celsi	scalp	France, Iran, Italy, Japan & Nigeria	Enweani et al. 1996, Pakshir and Hashemi 2006,Torres-Guerrero et al. 2015, Gits-Muselli et al. 2017, Yasuda-Sekiguchi et al. 2022
		Tinea corporis	left lateral wall of the chest & left shoulder	India	Raonand Datta 2013
Trichophyton verrucosum	*Trichophyton verrucosum*	Tinea corporis, tinea faciei, & tinea capitis	face, scalp, head, arms & legs	Australia	Maslen 2000
		Skin infections	left mid-calf and thigh, & face	Ireland	O'Gorman et al. 2015
Trichophyton violaceum	*Trichophyton violaceum*	Dermatophytosis (disseminated infections)	chest, face, & extremities	India & Iran	Pakshir and Hashemi 2006, Surpam et al. 2006
		Tinea capitis	scalp & palms	Greece, Italy, & USA	Vazquez-Lopez et al. 2011, Valari et al. 2012, Grigoryan et al. 2019
		Tinea capitis	scalp, neck, & shoulders	China	Yu et al. 2004
		Bullous tinea pedis	left foot	Italy	Romano et al. 2006
		Tinea corporis	forehead & cheeks	India	Smriti et al. 2015
	Trichophyton soudanense	Tinea capitis	Scalp	Africa, Italy, Spain, & USA	Romano et al. 2002, Ghilardi et al. 2007, Cetner et al. 2009, Rezusta et al. 2011, Grigoryan et al. 2019, Markey et al. 2003
		Endonyx onychomycosis	Fingernails	Somalia	Fletcher et al. 2001
Trichosporon asahii	*Trichosporon asahii*	Endocarditis	prosthetic aortic valve	Italy	Mulè et al. 2023
		Bloodstream Co-infection	blood	Brazil	Benelli et al. 2022
		Disseminated trichosporonosis	sputum & blood	India	Silva et al. 2003, Chowdhary et al. 2004
		Urinary tract infection	urinary tract	Chile & India	Sood et al. 2006
		prosthetic infection	knee	China	Zuo et al. 2015
		Fungemia	blood	Japan & Taiwan	Izumi et al. 2009, Shang et al. 2010
		Pneumonia	lungs	Spain	Segrelles-Calvo et al. 2021
Trichosporon beigelii	*Trichosporon beigelii*	Bloodstream Infection	blood	Georgia	Hajjeh and Blumberg 1995
		Disseminated infections	urine, skin, & blood	UK	Fisher et al. 1993, Sweet Reid 1998

Trichosporon cutaneum	*Trichosporon cutaneum*	Eczema	hands	Japan	Nakagawa et al. 2000
		Disseminated Infections	blood	Japan	Kataoka-Nishimura et al. 1998
		Folliculitis and septicaemia	face, chest, & legs	South Korea	Chang et al. 2005
Trichosporon inkin	*Trichosporon inkin*	White Piedra	scalp	India	Shivaprakash et al. 2011, Tendolkar et al. 2014
		Meningitis	brain and spinal cord	Brazil	Milan et al. 2018
		Peritonitis	abdomen	USA	Madariaga et al. 2003
Trichosporon mucoides	*Trichosporon mucoides*	Fungemia	blood	USA	Lacasse and Cleveland 2009
		Onychomycosis	fingernails	India & Italy	Sageerabanoo et al. 2011, Rizzitelli et al. 2016+
		Endocarditis	Prosthetic valve	South Korea, Canada	Oh et al. 2020, Tse et al. 2022
		white piedra	scalp	India	Tendolkar et al. 2014
		Peritonitis	abdomen	Taiwan	Chen et al. 2013
Trichosporon ovoides	*Trichosporon ovoides*	White Piedra	Scalp	India & Netherlands	Taj-Aldeen et al. 2004, Tambe et al. 2009
		Subcutaneous Infection	legs	Malaysia	Tap et al. 2016
		Invasive pulmonary infection	lungs	USA	Wynne et al. 2004, Padhi et al. 2014
Tropicoporus detonsus	*Inonotus tropicalis*	Sacral osteomyelitis	left lumbar spine	USA	Davis et al. 2007
		Keratitis	right eye	India	Gupta et al. 2022
		Osteomyelitis	spine & right knee	USA	Nguyen et al. 2009
Volvariella volvacea	*Volvariella volvacea*	Disseminated infection	skin, lung, & brain	China, Malaysia, & Singapore	Chew et al. 2019
		Disseminated	lungs and brain	Barbados & Singapore	Salit et al. 2010, Da et al. 2021
		Endocarditis and brain infection	heart & brain	Taiwan	Tien et al. 2020
Wickerhamomyces anomalus	*Candida pelliculosa*	Fungemia	blood	Korea, Taiwan, Turkey, & USA	Chan et al. 2013, Lin et al. 2013 Jung et al. 2013, Otag et al. 2015
		Endophthalmitis,	eye	Turkey	Esgin et al. 2014
		Meningitis	left eye and brain	UK	Ratcliffe et al. 2011
		Osteomyelitis	left knee	Korea	Song et al. 2020
	Wickerhamomyces anomalus	Fungemia	blood	Brazil, India, & Iran	Dutra et al. 2020, Mehta et al. 2020, Aboutalebian et al. 2022
		Keratitis	eye	Japan	Kamoshita et al. 2015
		Endophthalmitis	eye	Spain	Galván Ledesma et al. 2022
Xenoacremonium recifei	*Acremonium recifei*	Subcutaneous hyalohyphomycosis	right hand	Brazil	Zaitz et al. 1995

- Studies that have used molecular-based techniques to identify the pathogens are indicated in red.

References

Abbas, J., Bodey, G.P., Hanna, H.A., Mardani, M., Girgawy, E., Abi-Said, D., Whimbey, E., Hachem, R. and Raad, I., 2000. Candida krusei fungemia: an escalating serious infection in immunocompromised patients. Archives of internal medicine, 160(17), pp.2659-2664.

Abolghasemi, S., Hakamifard, A., Sharifynia, S., Pourabdollah Toutkaboni, M. and Azhdari Tehrani, H., 2021. Fatal invasive pulmonary aspergillosis in an immunocompetent patient with COVID-19 due to Aspergillus terreus: A case study. Clinical Case Reports, 9(4), pp.2414-2418.

Aboutalebian, S., Mirhendi, H., Eshaghi, H., Nikmaesh, B. and Charsizadeh, A., 2022. The first case of Wickerhamomyces anomalus fungemia in Iran in an immuneodeficient child, a review on the literature. Journal of Medical Mycology, p.101351.

Abuali, M.M., Posada, R., Del Toro, G., Roman, E., Ramani, R., Chaturvedi, S., Chaturvedi, V. and LaBombardi, V.J., 2009. Rhizomucor variabilis var. regularior and Hormographiella aspergillata infections in a leukemic bone marrow transplant recipient with refractory neutropenia. Journal of clinical microbiology, 47(12), pp.4176-4179.

Adams, E., Quinn, M., Tsay, S., Poirot, E., Chaturvedi, S., Southwick, K., Greenko, J., Fernandez, R., Kallen, A., Vallabhaneni, S. and Haley, V., 2018. Candida auris in healthcare facilities, New York, USA, 2013–2017. Emerging infectious diseases, 24(10), p.1816.

Agrawal, A. and Singh, S.M., 1995. Two cases of cutaneous phaeohyphomycosis caused by Curvularia pallescens: Zwei Fälle kutaner Phaeohyphomykose bedingt durch Curvularia pallescens. Mycoses, 38(7-8), pp.301-303.

Agarwal, S., Capoor, M.R., Ramesh, V., Rajni, R. and Khanna, G., 2011. First case of Acremonium kiliense mycetoma in a New Delhi resident: A brief review. Journal de mycologie médicale, 21(2), pp.130-133.

Agarwal, K., Kathuria, S., Sundar, G., Singh, P., Khanna, G. and Chowdhary, A., 2014. A case of allergic fungal rhinosinusitis due to Ceratocystis adiposa. Diagnostic Microbiology and Infectious Disease, 78(2), pp.196-198.

Aguirre, C., Euliarte, C., Finquelievich, J., de los Ángeles Sosa, M. and Giusiano, G., 2015. Fungemia and interstitial lung compromise caused by Malassezia sympodialis in a pediatric patient. Revista Iberoamericana de Micología, 32(2), pp.118-121.

Ahmed, S.A., Abbas, M.A., Jouvion, G., Al-Hatmi, A.M., de Hoog, G.S., Kolecka, A. and Mahgoub, E.S., 2015. Seventeen years of subcutaneous infection by Aspergillus flavus; eumycetoma confirmed by immunohistochemistry. Mycoses, 58(12), pp.728-734.

Ahmed, S.A., Hofmüller, W., Seibold, M., De Hoog, G.S., Harak, H., Tammer, I., Van Diepeningen, A.D. and Behrens-Baumann, W., 2017. Tintelnotia, a new genus in Phaeosphaeriaceae harbouring agents of cornea and nail infections in humans. Mycoses, 60(4), pp.244-253.

Aït-Ammar, N., Levesque, E., Murat, J.B., Imbert, S., Foulet, F., Dannaoui, E. and Botterel, F., 2018. Aspergillus pseudodeflectus: a new human pathogen in liver transplant patients. BMC Infectious Diseases, 18(1), pp.1-6.

Ajello, L., Padhye, A.A., Chandler, F.W., McGinnis, M.R., Morganti, L. and Alberici, F., 1985. Fusarium moniliforme, a new mycetoma agent restudy of a European case. European journal of epidemiology, 1(1), pp.5-10.

Akhaddar, A., Gazzaz, M., Albouzidi, A., Lmimouni, B., Elmostarchid, B. and Boucetta, M., 2008. Invasive Aspergillus terreus sinusitis with orbitocranial extension: case report. Surgical neurology, 69(5), pp.490-495.

Alanio, A., Romand, S., Penso-Assathiany, D., Foulet, F. and Botterel, F., 2011. Microsporum praecox: molecular identification of a new case and review of the literature. Mycopathologia, 171, pp.61-65.

Albisetti, M., Lauener, R.P., Güngör, T., Schär, G., Niggli, F.K. and Nadal, D., 2004. Disseminated Fusarium oxysporum infection in hemophagocytic lymphohistiocytosis. Infection, 32(6), pp.364-366.

Alčauskas, T., Zablockienė, B., Zablockis, R., Svetikas, L., Bilotaitė, L. and Jančorienė, L., 2022. Pulmonary Coccidioidomycosis: A Case Report and Literature Review. Medicina, 58(5), p.655.

Alegre-González, D., Herrera, S., Bernal, J., Soriano, A. and Bodro, M., 2021. Disseminated Cryptococcus neoformans infection associated to COVID-19. Medical mycology case reports, 34, pp.35-37.

Alex, D., Li, D., Calderone, R. and Peters, S.M., 2013. Identification of Curvularia lunata by polymerase chain reaction in a case of fungal endophthalmitis. Medical mycology case reports, 2, pp.137-140.

Al-Hatmi, A., Bonifaz, A., de Hoog, G.S., Vazquez-Maya, L., Garcia-Carmona, K., Meis, J.F. and van Diepeningen, A.D., 2014. Keratitis by Fusarium temperatum, a novel opportunist. BMC infectious diseases, 14(1), pp.1-9.

Al-Hatmi, A.M., Bonifaz, A., Tirado-Sánchez, A., Meis, J.F., de Hoog, G.S. and Ahmed, S.A., 2017. Fusarium species causing eumycetoma: report of two cases and comprehensive review of the literature. Mycoses, 60(3), pp.204-212.

Aljuboori, Z., Hruska, R., Yaseen, A., Arnold, F., Wojda, B. and Nauta, H., 2017. Fungal brain abscess caused by "Black Mold"(Cladophialophora bantiana)–a case report of successful treatment with an emphasis on how fungal brain abscess may be different from bacterial brain abscess. Surgical Neurology International, 8.

Allaw, F., Kara Zahreddine, N., Ibrahim, A., Tannous, J., Taleb, H., Bizri, A.R., Dbaibo, G. and Kanj, S.S., 2021. First Candida auris outbreak during a COVID-19 pandemic in a tertiary-care center in Lebanon. Pathogens, 10(2), p.157.

Al-Obaid, I., Ahmad, S., Khan, Z.U., Dinesh, B. and Hejab, H.M., 2006. Catheter-associated fungemia due to Exophiala oligosperma in a leukemic child and review of fungemia cases caused by Exophiala species. European Journal of Clinical Microbiology and Infectious Diseases, 25, pp.729-732.

Al-Rashdi A, Al-Maani A, Al-Wahaibi A, Alqayoudhi A, Al-Jardani A, Al-Abri S. Characteristics, risk factors, and survival analysis of Candida auris cases: results of one-year national surveillance data from Oman. Journal of Fungi. 2021 Jan 7;7(1):31.

Alshaya, O.A., Saleh, R.A. and Alshehri, S.D., 2021. Voriconazole-Induced Hepatotoxicity Resolved after Switching to Amphotericin B in Fusarium dimerum Central Line-Associated Bloodstream Infection. The American Journal of Case Reports, 22, pp.e932544-1.

Al Otaibi, T.M., Gheith, O.A., Alobaid, K., Nair, P., Eldein, S.M.Z., Mahmoud, T.S., Halim, M.A., Aboatya, H.H., Balaha, M.A., Nagib, A.M. and Al-Hatmi, A.M., 2021. Disseminated Rhinocladiella mackenziei infection in a kidney transplant recipient: A case report and literature review. Journal of Medical Mycology, 31(4), p.101196.

Al-Sweih, N., Ahmad, S., Khan, S., Joseph, L., Asadzadeh, M. and Khan, Z., 2019. Cyberlindnera fabianii fungaemia outbreak in preterm neonates in Kuwait and literature review. Mycoses, 62(1), pp.51-61.

Al-Sweih, N., Ahmad, S., Joseph, L., Khan, S. and Khan, Z., 2014. Malassezia pachydermatis fungemia in a preterm neonate resistant to fluconazole and flucytosine. Medical mycology case reports, 5, pp.9-11.

Al-Tawfiq, J.A. and Boukhamseen, A., 2011. Cerebral phaeohyphomycosis due to Rhinocladiella mackenziei (formerly Ramichloridium mackenziei): case presentation and literature review. Journal of Infection and Public Health, 4(2), pp.96-102.

Alvarez, M., Lopez Ponga, B., Rayon, C., Garcia Gala, J., Roson Porto, M.C., Gonzalez, M., Martinez-Suarez, J.V. and Rodriguez-Tudela, J.L., 1995. Nosocomial outbreak caused by Scedosporium prolificans (inflatum): four fatal cases in leukemic patients. Journal of clinical microbiology, 33(12), pp.3290-3295.

Al-Zaydani, I.A., Al-Hakami, A.M., Joseph, M.R., Kassem, W.M., Almaghrabi, M.K., Nageeb, A. and Hamid, M.E., 2015. Aggressive cutaneous zygomycosis caused by Apophysomyces variabilis in an immunocompetent child. Medical mycology case reports, 10, pp.11-13.

Amazan, E., Desbois, N., Fidelin, G., Baubion, E., Derancourt, C., Thimon, S., Ekindi, N. and Quist, D., 2014. First case of phaeohyphomycosis due to Pleurostoma ootheca in a kidney transplant recipient in Martinique (French West Indies). Médecine et Santé Tropicales, 24(3), pp.323-325.

Amona, F.M., Denning, D.W., Moukassa, D., Develoux, M. and Hennequin, C., 2021. Histoplasmosis in the Republic of Congo dominated by African histoplasmosis, Histoplasma capsulatum var. duboisii. PLoS Neglected Tropical Diseases, 15(5), p.e0009318.

Anadolu, R., Hilmioğlu, S., Oskay, T., E Boyvat, A., Peksari, Y. and Gürgey, E., 2001. Indolent Acremonium strictum infection in an immunocompetent patient. International journal of dermatology, 40(7), pp.451-453.

Anand, M., Deshmukh, S.D., Pande, D.P., Naik, S. and Ghadage, D.P., 2010. Subcutaneous zygomycosis due to Basidiobolus ranarum: a case report from Maharastra, India. Journal of Tropical Medicine, 2010.

Anane, S., Chtourou, O., Bodemer, C. and Kharfi, M., 2013. Malassezia folliculitis in an infant. Medical mycology case reports, 2, pp.72-74.

Andersen, A.J.B., Fuchs, C., Ardigo, M., Haedersdal, M. and Mogensen, M., 2018. In vivo characterization of pustules in Malassezia Folliculitis by reflectance confocal microscopy and optical coherence tomography. A case series study. Skin Research and Technology, 24(4), pp.535-541.

Anita, K., Fernandez, N. and Rao, R., 2010. Fungal endophthalmitis caused by Paecilomyces variotii, in an immunocompetent patient, following intraocular lens implantation. Indian Journal of Medical Microbiology, 28(3), p.253.

Ankad, B.S., Manjula, R., Tejasvi, T. and Nikam, B.P., 2019. Dermoscopy of eumycotic mycetoma: A case report. Dermatology Practical & Conceptual, 9(4), p.297.

Appenzeller, S., Amaral, T.N., Amstalden, E.M.I., Bertolo, M.B., Neto, J.F.M., Samara, A.M. and Fernandes, S.R.M., 2006. Sporothrix schenckii infection presented as monoarthritis: report of two cases and review of the literature. Clinical rheumatology, 25, pp.926-928.

Ara, M., Aspiroz, C., Zaballos, P., Alcalde, V., Alvarez, R., Rezusta, A. and Giménez, J.A., 2006. Relapse of cutaneous Alternaria infectoria in a renal transplant recipient after 2 years. Acta dermato-venereologica, 86(2), pp.154-155.

Araújo, M.J.C.L.N., Nihei, C.H., Rodrigues, A.M., Higashino, H., Ponzio, V., Pignatari, A.C.C., Barcellos, M.A., Braga, O. and Duayer, I.F., 2021. Case report: Invasive sinusitis due to Sporothrix brasiliensis in a renal transplant recipient. The American Journal of Tropical Medicine and Hygiene, 105(5), p.1218.

Arias, F., Mata-Essayag, S., Landaeta, M.E., de Capriles, C.H., Pérez, C., Núñez, M.J., Carvajal, A. and Silva, M., 2004. Candida albicans osteomyelitis: case report and literature review. International journal of infectious diseases, 8(5), pp.307-314.

Aribandi, M., Bazan Iii, C. and Rinaldi, M.G., 2005. Magnetic resonance imaging findings in fatal primary cerebral infection due to Chaetomium strumarium. *Australasian Radiology*, *49*(2), pp.166-169.

Arroyo, M.A., Schmitt, B.H., Davis, T.E. and Relich, R.F., 2016. Detection of the dimorphic phases of mucor circinelloides in blood cultures from an immunosuppressed female. Case Reports in Infectious Diseases, 2016.

Arunkumar, M.J., Rajshekhar, V., Chandy, M.J., Thomas, P.P. and Jacob, C.K., 2000. Management and outcome of brain abscess in renal transplant recipients. Postgraduate medical journal, 76(894), pp.207-211.

Atalay, A., Koc, A.N., Akyol, G., Cakir, N., Kaynar, L. and Ulu-Kilic, A., 2016. Pulmonary infection caused by Talaromyces purpurogenus in a patient with multiple myeloma. Infez Med, 24(2), pp.153-7.

Aspiroz, C., Gené, J., Rezusta, A., Charlez, L. and Summerbell, R.C., 2007. First Spanish case of onychomycosis caused by Chaetomium globosum. Sabouraudia, 45(3), pp.279-282.

Austen, B., McCarthy, H., Wilkins, B., Smith, A. and Duncombe, A., 2001. Fatal disseminated fusarium infection in acute lymphoblastic leukaemia in complete remission. Journal of clinical pathology, 54(6), pp.488-490.

Baby, S., Ramya, T.G. and Geetha, R.K., 2015. Onychomycosis by Syncephalastrum racemosum: case report from Kerala, India. Dermatology Reports, 7(1).

Baghdadi, J., Hemarajata, P., Humphries, R. and Kelesidis, T., 2015. First report of ventriculoperitoneal shunt infection due to Cyberlindnera fabianii. Case Reports in Infectious Diseases, 2015.

Badali, H., Fernández-González, M., Mousavi, B., Illnait-Zaragozi, M.T., González-Rodríguez, J.C., de Hoog, G.S. and Meis, J.F., 2013. Chromoblastomycosis due to Fonsecaea pedrosoi and F. monophora in Cuba. Mycopathologia, 175, pp.439-444.

Ballestas, S.A., Magliocca, K.R., Balter, L. and Hatcher, J.L., 2020. Laryngeal blastomycosis, an unexpected diagnosis: a case report. Journal of Voice.

Balne, P.K., Nalamada, S., Kodiganti, M. and Taneja, M., 2012. Fungal keratitis caused by Chaetomium atrobrunneum. Cornea, 31(1), pp.94-95.

Banerjee, U., Gupta, K. and Venugopal, P., 1997. A case of prosthetic valve endocarditis caused by Cryptococcus neoformans var. neoformans. Journal of medical and veterinary mycology, 35(2), pp.139-141.

Baradkar, V.P. and Kumar, S., 2009. Cutaneous zygomycosis due to Saksenaea vasiformis in an immunocompetent host. Indian journal of dermatology, 54(4), p.382.

Barba-Gómez, J.F., Mayorga, J., McGinnis, M.R. and González-Mendoza, A., 1992. Chromoblastomycosis caused by Exophiala spinifera. Journal of the American Academy of Dermatology, 26(2), pp.367-370.

Barocas, J.A. and Gauthier, G.M., 2014. Peritonitis caused by B lastomyces dermatitidis in a kidney transplant recipient: case report and literature review. Transplant Infectious Disease, 16(4), pp.634-641.

Barantsevich, N.E., Orlova, O.E., Shlyakhto, E.V., Johnson, E.M., Woodford, N., Lass-Floerl, C., Churkina, I.V., Mitrokhin, S.D., Shkoda, A.S. and Barantsevich, E.P., 2019. Emergence of Candida auris in Russia. Journal of Hospital Infection, 102(4), pp.445-448.

Barber, G.R., Brown, A.E., Kiehn, T.E., Edwards, F.F. and Armstrong, D., 1993. Catheter-related Malassezia furfur fungemia in immunocompromised patients. The American journal of medicine, 95(4), pp.365-370.

Bard, J.D., Mangahis, A., Hofstra, T.C. and Bender, J.M., 2014. First case report of bloodstream infection by Rhizomucor pusillus in a child with hemophagocytic lymphohistiocytosis. Medical mycology case reports, 5, pp.20-23.

Barrs, V.R., van Doorn, T.M., Houbraken, J., Kidd, S.E., Martin, P., Pinheiro, M.D., Richardson, M., Varga, J. and Samson, R.A., 2013. Aspergillus felis sp. nov., an emerging agent of invasive aspergillosis in humans, cats, and dogs. PLoS One, 8(6), p.e64871.

Bartash, R., Guo, Y., Pope, J.B., Levi, M.H., Szymczak, W., Saraiya, N. and Nori, P., 2017. Periprosthetic hip joint infection with Aspergillus terreus: a clinical case and a review of the literature. Medical mycology case reports, 18, pp.24-27.

Batra, N., Kaur, H., Mohindra, S., Singh, S., Shamanth, A.S. and Rudramurthy, S.M., 2019. Cladosporium sphaerospermum causing brain abscess, a saprophyte turning pathogen: Case and review of published reports. Journal de Mycologie Médicale, 29(2), pp.180-184.

Bay, C., Gonzalez, T., Munoz, G., Legarraga, P., Vizcaya, C. and Abarca, K., 2017. Nasal phaeohyphomycosis by Curvularia spicifera in pediatric patient with neutropenia and acute myeloid leukemia. Revista Chilena de Infectologia: Organo Oficial de la Sociedad Chilena de Infectologia, 34(3), pp.280-286.

Behera, B., Singh, R.I., Xess, I., Mathur, P., Hasan, F. and Misra, M.C., 2010. Candida rugosa: a possible emerging cause of candidaemia in trauma patients. Infection, 38(5), pp.387-393.

Bellanda, V.F. and See, C.W., 2023. Coniochaeta mutabilis keratitis with an unusual mode of transmission treated with penetrating keratoplasty and systemic antifungals. *American Journal of Ophthalmology Case Reports*, *32*, p.101930.

Benelli, J.L., Basso, R.P., Grafulha, T.W., Poester, V.R., Munhoz, L.S., Martins, K.B., Zogbi, H.E., Von Groll, A., Severo, C.B., Stevens, D.A. and Xavier, M.O., 2022. Fungal bloodstream co-infection by Trichosporon asahii in a COVID-19 critical patient: case report and literature review. Mycopathologia, 187(4), pp.397-404.

Berbel, R.F., Casella, A.M.B., de Freitas, D. and Höfling-Lima, A.L., 2011. Curvularia lunata endophthalmitis. Journal of ocular pharmacology and therapeutics, 27(5), pp.535-537.

Berejnoi, A., Taverna, C.G., Mazza, M., Vivot, M., Isla, G., Córdoba, S. and Davel, G., 2019. First case report of cryptococcosis due to Cryptococcus decagattii in a pediatric patient in Argentina. Revista da Sociedade Brasileira de Medicina Tropical, 52.

Beresford, R., Dolot, V. and Foo, H., 2019. Cranial aspergillosis in a patient receiving ibrutinib for chronic lymphocytic leukemia. Medical mycology case reports, 24, pp.27-29.

Berg, D., Garcia, J.A., Schell, W.A., Perfect, J.R. and Murray, J.C., 1995. Cutaneous infection caused by Curvularia pallescens: a case report and review of the spectrum of disease. Journal of the American Academy of Dermatology, 32(2), pp.375-378.

Berg, J.C., Hamacher, K.L. and Roberts, G.D., 2007. Pseudomycetoma caused by Microsporum canis in an immunosuppressed patient: a case report and review of the literature. Journal of cutaneous pathology, 34(5), pp.431-434.

Bertumen, J.B., Schell, W.A., Joyce, M., Alley, C. and Woods, C.W., 2016. Diagnostic difficulty identifying Apophysomyces trapeziformis septic arthritis in a patient with multiple myeloma. JMM case reports, 3(6).

Bibashi, E., Sidi, V., Kotsiou, M., Makrigiannaki, E. and Koliouskas, D., 2008. Pulmonary zygomycosis caused by Cunninghamella bertholletiae in a child with acute lymphoblastic leukemia. Hippokratia, 12(1), p.43.

Bibashi, E., de Hoog, G.S., Pavlidis, T.E., Symeonidis, N., Sakantamis, A. and Walther, G., 2013. Wound infection caused by Lichtheimia ramosa due to a car accident. Medical mycology case reports, 2, pp.7-10.

Bigley, V.H., Duarte, R.F., Gosling, R.D., Kibbler, C.C., Seaton, S. and Potter, M., 2004. Fusarium dimerum infection in a stem cell transplant recipient treated successfully with voriconazole. Bone marrow transplantation, 34(9), pp.815-817.

Bonifaz, A., Badali, H., De Hoog, G.S., Cruz, M., Araiza, J., Cruz, M.A., Fierro, L. and Ponce, R., 2008. Tinea nigra by Hortaea werneckii, a report of 22 cases from Mexico. Studies in Mycology, 61, pp.77-82.

Boukassa, L., Ngackosso, O.B., Kinata Bambino, S.B., Ekouele Mbaki, H.B., Ngounda Monianga, S.A., Mavoungou Biatsi, K. and Pecko, J.F., 2019. Cranial and Spinal Locations of Histoplasma capsulatum var. duboisii in Brazzaville, Congo. Iranian Journal of Neurosurgery, 5(2), pp.63-69.

Badali, H., Carvalho, V.O., Vicente, V., Attili-Angelis, D., Kwiatkowski, I.B., Van Den Ende, A.G. and De Hoog, G.S., 2009. Cladophialophora saturnica sp. nov., a new opportunistic species of Chaetothyriales revealed using molecular data. Medical Mycology, 47(1), pp.51-66.

Baddley, J.W., Moser, S.A., Sutton, D.A. and Pappas, P.G., 2000. Microascus cinereus (anamorph Scopulariopsis) brain abscess in a bone marrow transplant recipient. Journal of Clinical Microbiology, 38(1), pp.395-397.

Balne, P.K., Nalamada, S., Kodiganti, M. and Taneja, M., 2012. Fungal keratitis caused by Chaetomium atrobrunneum. *Cornea*, *31*(1), pp.94-95.

Barbaryan, A., El Atrouni, W., Bailuc, S., Jones, M.W., Bhakta, M., Mahmoud, K.H. and Mirrakhimov, A.E., 2018. Isolated Sporothrix schenckii monoarthritis. Case Reports in Infectious Diseases, 2018.

Barron, M.A., Sutton, D.A., Veve, R., Guarro, J., Rinaldi, M., Thompson, E., Cagnoni, P.J., Moultney, K. and Madinger, N.E., 2003. Invasive mycotic infections caused by Chaetomium perlucidum, a new agent of cerebral phaeohyphomycosis. *Journal of clinical microbiology*, *41*(11), pp.5302-5307.

Bellanger, A.P., Cervoni, J.P., Faucher, J.F., Weil-Verhoeven, D., Ginet, M., Deconinck, E. and Grenouillet, F., 2017. Paecilomyces variotii fungemia in a patient with lymphoma needing liver transplant. Mycopathologia, 182(7), pp.761-765.

Benítez, I., Rodríguez, M., Lezcano, V., Morel, Z., Pereira, J., Brizuela, S. and Galleano, H., 2019. Disseminated histoplasmosis with skin lesions and osteomyelitis in a child with acute lymphoblastic leukemia undergoing maintenance treatment. Pediatría (Asunción), 46(1), pp.38-42.

Bernacer, M., Gadea, I., Esteban, J., Gegundez, M.I., Kamal, K. and Soriano, F., 1992. Catheter-related fungemia due to Paecilomyces lilacinus in a leukemic child. Med Microbiol Lett, 1, pp.207-212.

Bhaskaran, A., Obeid, K.M., Arbefeville, S. and Ferrieri, P., 2016. Trichosporon loubieri fungemia in a 39-year-old Caucasian woman with B-cell lymphoblastic leukemia. Laboratory medicine, 47(3), pp.255-258.

Bhat, S.V., Paterson, D.L., Rinaldi, M.G. and Veldkamp, P.J., 2007. Scedosporium prolificans brain abscess in a patient with chronic granulomatous disease: successful combination therapy with voriconazole and terbinafine. Scandinavian journal of infectious diseases, 39(1), pp.87-90.

Bibashi, E., de Hoog, G.S., Pavlidis, T.E., Symeonidis, N., Sakantamis, A. and Walther, G., 2013. Wound infection caused by Lichtheimia ramosa due to a car accident. Medical mycology case reports, 2, pp.7-10.

Bigley, V.H., Duarte, R.F., Gosling, R.D., Kibbler, C.C., Seaton, S. and Potter, M., 2004. Fusarium dimerum infection in a stem cell transplant recipient treated successfully with voriconazole. Bone marrow transplantation, 34(9), pp.815-817.

Blazquez, D., Ruiz-Contreras, J., Fernández-Cooke, E., González-Granado, I., Delgado, M.D., Menendez, M.T., Rodriguez-Gil, Y., Ballen, A. and Del Palacio, A., 2010. Lichtheimia corymbifera subcutaneous infection successfully treated with amphotericin B, early debridement, and vacuum-assisted closure. Journal of pediatric surgery, 45(12), pp.e13-e15.

Boan, P., Pang, S., Mowlaboccus, S., Wrobel, J.P., Musk, M., Lavender, M., Yaw, M.C., Hernest, A., MacQuillan, G., Dembo, L.G. and Coombs, G.W., 2022. Apophysomyces Variabilis Infection in Transplant Recipients due to Unrecognized Infection in an Intravenous Drug–Using Donor. Transplantation, 106(2), pp.e169-e171.

Bodey, G.P., Mardani, M., Hanna, H.A., Boktour, M., Abbas, J., Girgawy, E., Hachem, R.Y., Kontoyiannis, D.P. and Raad, I.I., 2002. The epidemiology of Candida glabrata and Candida albicans fungemia in immunocompromised patients with cancer. The American journal of medicine, 112(5), pp.380-385.

Bojic, M., Willinger, B., Rath, T., Tobudic, S., Thalhammer, F., Böhm, A., Mitterbauer, M., Schulenburg, A., Wöhrer, S., Kalhs, P. and Rabitsch, W., 2013. Fatal skin and pulmonary infection caused by H ormographiella aspergillata in a leukaemic patient: case report and literature overview. Mycoses, 56(6), pp.687-689.

Bonatti, H., Lass-Flörl, C., Zelger, B., Lottersberger, C., Singh, N., Pruett, T.L., Margreiter, R. and Schneeberger, S., 2007. Alternaria alternata soft tissue infection in a forearm transplant recipient. Surgical infections, 8(5), pp.539-544.

Bonifaz, A., Córdoba-García, B., Simancas-Llanos, T., Hernández, M.A., Martínez-Herrera, E. and Tirado-Sánchez, A., 2019. Dermatophytosis caused by Nannizzia nana in two siblings. Revista Iberoamericana de Micología, 36(1), pp.30-33.

Borkar, S.A., Sharma, M.S., Rajpal, G., Jain, M., Xess, I. and Sharma, B.S., 2008. Brain abscess caused by Cladophialophora bantiana in an immunocompetent host: need for a novel cost-effective antifungal agent.

Botterel, F., Romand, S., Cornet, M., Recanati, G., Dupont, B. and Bourée, P., 2001. Dermatophyte pseudomycetoma of the scalp: case report and review. British Journal of Dermatology, 145(1), pp.151-153.

Borman, A.M., Szekely, A., Fraser, M., Lovegrove, S. and Johnson, E.M., 2019. A novel dermatophyte relative, Nannizzia perplicata sp. nov., isolated from a case of tinea corporis in the United Kingdom. Medical Mycology, 57(5), pp.548-556.

Bougrine, A., Villeneuve-Tang, C., Bouffard, D., Rouleau, D. and Chartier, S., 2014. Kerion of the vulva caused by Trichophyton mentagrophytes. Journal of Cutaneous Medicine and Surgery, 18(3), pp.206-209.

Bracca, A., Tosello, M.E., Girardini, J.E., Amigot, S.L., Gomez, C. and Serra, E., 2003. Molecular detection of Histoplasma capsulatum var. capsulatum in human clinical samples. Journal of Clinical Microbiology, 41(4), pp.1753-1755.

Brasch, J., Busch, J.O. and de Hoog, G.S., 2008. Cutaneous phaeohyphomycosis caused by Alternaria infectoria. Acta dermato-venereologica, 88(2), pp.160-161.

Brasch, J., Beck-Jendroschek, V., Iturrieta-González, I., Voss, K. and Gené, J., 2019. A human subcutaneous infection by Microascus ennothomasiorum sp. nov. Mycoses, 62(2), pp.157-164.

Brasch, J., Dressel, S., Müller-Wening, K., Hügel, R., von Bremen, D. and De Hoog, G.S., 2011. Toenail infection by Cladophialophora boppii. Medical Mycology, 49(2), pp.190-193.

Brilhante, R.S., Fechine, M.A., Mesquita, J.R., Cordeiro, R.A., Rocha, M.F., Monteiro, A.J., Lima, R.A., Caetano, É.P., Pereira, J.F., Castelo-Branco, D.S. and Camargo, Z.P., 2012. Histoplasmosis in HIV-positive patients in Ceará, Brazil: clinical-laboratory aspects and in vitro antifungal susceptibility of Histoplasma capsulatum isolates. Transactions of the Royal Society of Tropical Medicine and Hygiene, 106(8), pp.484-488.

Brito, L.R., Guimarães, T., Nucci, M., Rosas, R.C., Paula Almeida, L., Da Matta, D.A. and Colombo, A.L., 2006. Clinical and microbiological aspects of candidemia due to Candida parapsilosis in Brazilian tertiary care hospitals. Medical Mycology, 44(3), pp.261-266.

Brandt, M.E., Harrison, L.H., Pass, M., Sofair, A.N., Huie, S., Li, R.K., Morrison, C.J., Warnock, D.W. and Hajjeh, R.A., 2000. Candida dubliniensis fungemia: the first four cases in North America. Emerging infectious diseases, 6(1), p.46.

Brischetto, A., Kidd, S. and Baird, R., 2015. Case Report: First Reported Australian Case of Cladophilophora arxii: Features Consistent with Possible Primary Pulmonary Chromoblastomycosis. The American journal of tropical medicine and hygiene, 92(4), p.791.

Brown, D.A., Whealy, M.A., Van Gompel, J.J., Williams, L.N. and Klaas, J.P., 2015. Diagnostic dilemma in primary Blastomyces dermatitidis meningitis: role of neurosurgical biopsy. Case Reports in Neurology, 7(1), pp.63-70.

Burnik, C., Altintaş, N.D., Özkaya, G., Serter, T., Selcuk, Z.T., Firat, P., Arikan, S., Cuenca-Estrella, M. and Topeli, A.R.Z.U., 2007. Acute respiratory distress syndrome due to Cryptococcus albidus pneumonia: case report and review of the literature. Medical mycology, 45(5), pp.469-473.

Buchta, V., Feuermannová, A., Váša, M., Bašková, L., Kutová, R., Kubátová, A. and Vejsová, M., 2014. Outbreak of fungal endophthalmitis due to Fusarium oxysporum following cataract surgery. Mycopathologia, 177(1), pp.115-121.

Buchta, V., Nekolová, J., Jirásková, N., Bolehovská, R., Wipler, J. and Hubka, V., 2019. Fungal keratitis caused by Colletotrichum dematium: case study and review. Mycopathologia, 184, pp.441-453.

Bukhari, E. and Alrabiaah, A., 2009. First case of extensive spinal cord infection with Aspergillus nidulans in a child with chronic granulomatous disease. The Journal of Infection in Developing Countries, 3(04), pp.321-323.

Cai, Q., Lv, G.X., Jiang, Y.Q., Mei, H., Hu, S.Q., Xu, H.B., Wu, X.F., Shen, Y.N. and Liu, W.D., 2013. The first case of phaeohyphomycosis caused by Rhinocladiella basitona in an immunocompetent child in China. Mycopathologia, 176(1-2), pp.101-105.

Calvo, B., Melo, A.S., Perozo-Mena, A., Hernandez, M., Francisco, E.C., Hagen, F., Meis, J.F. and Colombo, A.L., 2016. First report of Candida auris in America: clinical and microbiological aspects of 18 episodes of candidemia. Journal of Infection, 73(4), pp.369-374.

Camargo, J.F., Jabr, R.E., Anderson, A.D., Lekakis, L., Diaz-Paez, M., Briski, L.M., Raja, M., Morris, M.I., Komanduri, K.V. and Pereira, D., 2022. Successful treatment of disseminated disease due to highly resistant Aspergillus calidoustus with a novel antifungal therapy. Antimicrobial agents and chemotherapy, 66(3), pp.e02206-21.

Campos, A.G.M., de Hollanda, L.E., Oliveira, L.M., do Valle, F.F. and do Valle, V.A.F., 2018. Squamous cell carcinoma arising from a chromomycosis lesion caused by Rhinocladiella aquaspersa with postsurgical recurrence of chromomycosis. JAAD Case Reports, 4(9), pp.915-917.

Campos-Herrero, M.I., Bordes, A., Perera, A., Ruiz, M.C. and Fernandez, A., 1996. Trichodermia koningii peritonitis in a patient undergoing peritoneal dialysis. Clinical Microbiology Newsletter, 18(19), pp.150-152.

Campos-Macías, P., Arenas-Guzmán, R. and Hernández-Hernández, F., 2013. Fusarium subglutinans: a new eumycetoma agent. Medical mycology case reports, 2, pp.128-131.

Cantón, E., Pemán, J., Quindós, G., Eraso, E., Miranda-Zapico, I., Álvarez, M., Merino, P., Campos-Herrero, I., Marco, F., de la Pedrosa, E.G.G. and Yagüe, G., 2011. Prospective multicenter study of the epidemiology, molecular identification, and antifungal susceptibility of Candida parapsilosis, Candida orthopsilosis, and Candida metapsilosis isolated from patients with candidemia. Antimicrobial agents and chemotherapy, 55(12), pp.5590-5596.

Capoor, M.R., Agarwal, P., Goel, M., Jain, S., Shivaprakash, M.R., Honnavar, P., Gupta, S. and Chakrabarti, A., 2016. Invasive pulmonary mycosis due to Chaetomium globosum with false-positive galactomannan test: a case report and literature review. *Mycoses*, *59*(3), pp.186-193.

Caraway, N.P., Fanning, C.V., Stewart, J.M., Tarrand, J.J. and Weber, K.L., 2003. Coccidioidomycosis osteomyelitis masquerading as a bone tumor. A report of 2 cases. Acta cytologica, 47(5), pp.777-782.

Carey, J., D'Amico, R., Sutton, D.A. and Rinaldi, M.G., 2003. Paecilomyces lilacinus vaginitis in an immunocompetent patient. Emerging Infectious Diseases, 9(9), p.1155.

Carter, E. and Boudreaux, C., 2004. Fatal cerebral phaeohyphomycosis due to Curvularia lunata in an immunocompetent patient. Journal of Clinical Microbiology, 42(11), pp.5419-5423.

Carvalho, V.O., Vicente, V.A., Werner, B., Gomes, R.R., Fornari, G., Herkert, P.F., Rodrigues, C.O., Abagge, K.T., Robl, R. and Camiña, R.H., 2014. Onychomycosis by Fusarium oxysporum probably acquired in utero. Medical mycology case reports, 6, pp.58-61.

Castiglioni, B., Sutton, D.A., Rinaldi, M.G., Fung, J. and Kusne, S., 2002. Pseudallescheria boydii (anamorph Scedosporium apiospermum) infection in solid organ transplant recipients in a tertiary medical center and review of the literature. Medicine, 81(5), pp.333-348.

Castro, L.Á., Álvarez, M.I. and Martínez, E., 2010. Case report of Schizophyllum commune sinusitis in an immunocompetent patient. Colombia Médica, 41(1), pp.71-75.

Castro-Echeverry, E., Fiala, K. and Fernandez, M.P., 2017. Dermatophytic pseudomycetoma of the scalp. The American Journal of dermatopathology, 39(2), pp.e23-e25.

Cavanna, C., Seminari, E., Pusateri, A., Mangione, F., Lallitto, F., Esposto, M.C. and Pagella, F., 2014. Allergic fungal rhinosinusitis due to Curvularia lunata. The new Microbiologica, 37(2), pp.241-245.

Cavanna, C., Pagella, F., Esposto, M.C., Tamarozzi, F., Clemente, L., Marone, P., Matti, E. and Lallitto, F., 2019. Human infections due to Schizophyllum commune: case report and review of the literature. Journal de Mycologie Médicale, 29(4), pp.365-371.

Cetner, A., Krunic, A., Tesic, V. and Janda, W., 2009. Tinea capitis due to Trichophyton soudanense in Chicago, Illinois: report of a case. Pediatric dermatology, 26(2), pp.226-228.

Cawcutt, K., De Moraes, A.G., Lee, S.J., Park, J.G., Schears, G.J. and Nemergut, M.E., 2014. The use of ECMO in HIV/AIDS with Pneumocystis jirovecii Pneumonia: a case report and review of the literature. Asaio Journal, 60(5), pp.606-608.

Chander, J., Singla, N., Kaur, M., Punia, R.S., Attri, A., Alastruey-Izquierdo, A., Cano-Lira, J.F., Stchigel, A.M. and Guarro, J., 2017. Saksenaea erythrospora, an emerging mucoralean fungus causing severe necrotizing skin and soft tissue infections–a study from a tertiary care hospital in north India. Infectious Diseases, 49(3), pp.170-177.

Chakrabarti, A., Marak, R.S.K., Singhi, S., Gupta, S., Hurst, S.F. and Padhye, A.A., 2006. Brain abscess due to Aspergillus nidulans. Journal de Mycologie Médicale, 16(2), pp.100-104.

Chhabra, V., Rastogi, S., Barua, M. and Kumar, S., 2013. Alternaria alternata infection associated osteomyelitis of maxilla: A rare disease entity. Indian Journal of Dental Research, 24(5), p.639.

Chai, F.C., Auret, K., Christiansen, K., Yuen, P.W. and Gardam, D., 2000. Malignant otitis externa caused by Malassezia sympodialis. Head & Neck: Journal for the Sciences and Specialties of the Head and Neck, 22(1), pp.87-89.

Chakrabarti, A., Kumar, P., Padhye, A.A., Chatha, L., Singh, S.K., Das, A., Wig, J.D. and Kataria, R.N., 1997. Primary cutaneous zygomycosis due to Saksenaea vasiformis and Apophysomyces elegans. Clinical infectious diseases, 24(4), pp.580-582.

Chakrabarti, A., SHIVAPRAKASH, M.R., Singh, R., Tarai, B., George, V.K., Fomda, B.A. and Gupta, A., 2008. Fungal endophthalmitis: fourteen years' experience from a center in India. Retina, 28(10), pp.1400-1407.

Champa, H., Sreeshma, P., Prakash, P.Y. and Divya, M., 2013. Cutaneous infection with Cylindrocarpon lichenicola. Medical mycology case reports, 2, pp.55-58.

Chan, A.W., Cartwright, E.J., Reddy, S.C., Kraft, C.S. and Wang, Y.F., 2013. Pichia anomala (Candida pelliculosa) fungemia in a patient with sickle cell disease. Mycopathologia, 176(3), pp.273-277.

Chandra, S. and Woodgyer, A., 2002. Primary cutaneous zygomycosis due to Mucor circinelloides. Australasian journal of dermatology, 43(1), pp.39-42.

Chang, S.E., Lee, D.K., Choi, J.H., Moon, K.C. and Koh, J.K., 2005. Majocchi's granuloma of the vulva caused by Trichophyton mentagrophytes. Mycoses, 48(6), pp.382-384.

Chang, S.E., Kim, K.J., Lee, W.S., Choi, J.H., Sung, K.J., Moon, K.C. and Koh, J.K., 2003. A case of Trichosporon cutaneum folliculitis and septicaemia. Clinical and experimental dermatology, 28(1), pp.37-38.

Chauhan, A., Gruenberg, J., Arbefeville, S., Mettler, T., Brent, C.H. and Ferrieri, P., 2019. Disseminated Hormographiella aspergillata infection with lung and brain involvement after Allogenic hematopoietic stem-cell transplantation in a 54-year-old man. Laboratory Medicine, 50(4), pp.426-431.

Chaves, A.L.S., Trilles, L., Alves, G.M., Figueiredo-Carvalho, M.H.G., Brito-Santos, F., Coelho, R.A., Martins, I.S. and Almeida-Paes, R., 2021. A case-series of bloodstream infections caused by the Meyerozyma guilliermondii species complex at a reference center of oncology in Brazil. Medical mycology, 59(3), pp.235-243.

Chazan, B., Colodner, R., Polacheck, I., Shoufani, A., Rozenman, D. and Raz, R., 2004. Mycetoma of the foot caused by Cylindrocarpon lichenicola in an immunocompetent traveler. Journal of travel medicine, 11(5), pp.331-332.

Chen, Y.T., Yang, W.C., Chen, T.W. and Lin, C.C., 2013. Trichosporon mucoides peritonitis in a continuous ambulatory peritoneal dialysis patient. Peritoneal Dialysis International, 33(3), pp.341-342.

Chen, W., Seidl, H.P., Ring, J. and Schnopp, C., 2012. Two pediatric cases of Microsporum persicolor infection. International Journal of Dermatology, 51(2), pp.204-206.

Chen, J., He, T., Li, X., Wang, X., Peng, L. and Ma, L., 2020. Metagenomic next-generation sequencing in diagnosis of a case of pneumocystis jirovecii pneumonia in a kidney transplant recipient and literature review. Infection and drug resistance, pp.2829-2836.

Cheng, J., Zeng, D., Zhang, T., Zhang, L., Han, X., Zhou, P., Wang, L., He, J. and Han, Q., 2023. Microascus cirrosus SZ 2021: A potentially new genotype of Microascus cirrosus, which can cause fatal pulmonary infection in patients with acute leukemia following haplo-HSCT. Experimental and Therapeutic Medicine, 26(2), pp.1-12.

Chen, WT., Tu, ME. & Sun, PL. Superficial Phaeohyphomycosis Caused by *Aureobasidium melanogenum* Mimicking Tinea Nigra in an Immunocompetent Patient and Review of Published Reports. *Mycopathologia* **181**, 555–560 (2016). https://doi.org/10.1007/s11046-016-9989-3

Chew, K.L., Ng, D.H.L., Teo, J.W.P., Tan, K.B., Poon, L.M., Tambyah, P.A. and Ong, C.W.M., 2019. Disseminated Volvariella volvacea infections in patients with haematological malignancies: a case series. Clinical Microbiology and Infection, 25(1), pp.117-119.

Chi, C.C. and Wang, S.H., 2007. Disseminated cutaneous Fusarium moniliforme infections in a leukemic child. International journal of dermatology, 46(5), pp.487-489.

Chik, K.K. and To, W.K., 2020. Autochthonous Emergomyces pasteurianus pneumonia in an immunocompromised patient in Hong Kong: a case report. Hong Kong Med J, 26(5), pp.446-8.

Chim, C.S., Ho, P.L. and Yuen, K.Y., 1998. Simultaneous Aspergillus fischeri and Herpes simplex pneumonia in a patient with multiple myeloma. Scandinavian journal of infectious diseases, 30(2), pp.190-191.

Choe, Y.J., Blatt, D.B., Yalcindag, A., Geffert, S.F., Bobenchik, A.M. and Michelow, I.C., 2020. Cryptococcus albidus fungemia in an immunosuppressed child: case report and systematic literature review. Journal of the Pediatric Infectious Diseases Society, 9(1), pp.100-105.

Choi, H.I., An, J., Hwang, J.J., Moon, S.Y. and Son, J.S., 2017. Otomastoiditis caused by Candida auris: Case report and literature review. Mycoses, 60(8), pp.488-492.

Choudhury S and Marte RL. Malassezia pachydermatis fungaemia in an adult on posaconazole prophylaxis for acute myeloid leukaemia. Pathology 2014;46:466-7

Chowdhary, A., Randhawa, H.S., Singh, V., Khan, Z.U., Ahmad, S., Kathuria, S., Roy, P., Khanna, G. and Chandra, J., 2011. Bipolaris hawaiiensis as etiologic agent of allergic bronchopulmonary mycosis: first case in a paediatric patient. Medical Mycology, 49(7), pp.760-765.

Chowdhary, A., Randhawa, H.S., Khan, Z.U., Ahmad, S., Khanna, G., Gupta, R., Chakravarti, A. and Roy, P., 2010. Rhinoentomophthoromycosis due to Conidiobolus coronatus. A case report and an overview of the disease in India. Sabouraudia, 48(6), pp.870-879.

Chowdhary, A., Agarwal, K., Randhawa, H.S., Kathuria, S., Gaur, S.N., Najafzadeh, M.J., Roy, P., Arora, N., Khanna, G. and Meis, J.F., 2012. A rare case of allergic bronchopulmonary mycosis caused by Alternaria alternata. Medical Mycology, 50(8), pp.890-896.

Chowdhary, A., Randhawa, H.S., Gaur, S.N., Agarwal, K., Kathuria, S., Roy, P., Klaassen, C.H. and Meis, J.F., 2013. Schizophyllum commune as an emerging fungal pathogen: a review and report of two cases. Mycoses, 56(1), pp.1-10.

Chowdhary, A., Ahmad, S., Khan, Z.U., Doval, D.C. and Randhawa, H.S., 2004. Trichosporon asahii as an emerging etiologic agent of disseminated trichosporonosis: a case report and an update. Indian Journal of Medical Microbiology, 22(1), pp.16-22.

Chowfin, A., Tight, R. and Mitchell, S., 2000. Recurrent blastomycosis of the central nervous system: case report and review. Clinical infectious diseases, 30(6), pp.969-971.

Chow, J.K., Golan, Y., Ruthazer, R., Karchmer, A.W., Carmeli, Y., Lichtenberg, D., Chawla, V., Young, J. and Hadley, S., 2008. Factors associated with candidemia caused by non-albicans Candida species versus Candida albicans in the intensive care unit. Clinical infectious diseases, 46(8), pp.1206-1213.

Chu, C.M. and Lai, R.W., 2002. Malassezia furfur fungaemia in a ventilator-dependent patient without known risk factors. Hong Kong Medical Journal, 8(3), pp.212-215.

Civelek, R., Çakar, M.K., Yegin, Z.A., Erbaş, G., Tunçcan, Ö.G., Kalkancı, A. and Sucak, G.T., 2016. Acremonium Potronii Associated Pneumonia in an Allogeneic Stem Cell Transplantation Recipient. Gazi Medical Journal, 27(4).

Collado, C., Medina, L., Zorraquino, A., Baeza, T., Ferrer, C., Plazas, J. and Colom, M.F., 2013. Cutaneous fusariosis by a species of the Fusarium dimerum species complex in a patient with acute mieloblastic leukemia. Revista Iberoamericana de Micología, 30(2), pp.119-121.

Colombo, A., Maccari, G., Congiu, T., Basso, P., Baj, A. and Toniolo, A., 2013. Colonization of a central venous catheter by the hyaline fungus Fusarium solani species complex: a case report and SEM imaging. Case Reports in Medicine, 2013.

Coelho, D., Silva, S., Vale-Silva, L., Gomes, H., Pinto, E., Sarmento, A. and Pinheiro, M.D., 2011. Aspergillus viridinutans: an agent of adult chronic invasive aspergillosis. Medical Mycology, 49(7), pp.755-759.

Coleman, H., Snell, L.B., Simons, R., Douthwaite, S.T. and Lee, M.J., 2020. COVID-19 and Pneumocystis jirovecii pneumonia: a diagnostic dilemma in HIV. AIDS (London, England).

Comacle, P., Le Govic, Y., Hoche-Delchet, C., Sandrini, J., Aguilar, C., Bouyer, B., Blanchi, S. and Penn, P., 2016. Spondylodiscitis due to Aspergillus terreus in an immunocompetent host: case report and literature review. Mycopathologia, 181(7), pp.575-581.

Concha, M., Nicklas, C., Balcells, E., Guzmán, A.M., Poggi, H., León, E. and Fich, F., 2012. The first case of tinea faciei caused by Trichophyton mentagrophytes var. erinacei isolated in Chile. International journal of dermatology, 51(3), pp.283-285.

Cooke, N.S., Feighery, C., Armstrong, D.K.B., Walsh, M. and Dempsey, S., 2009. Cutaneous Fusarium solani infection in childhood acute lymphoblastic leukaemia. Clinical and Experimental Dermatology: Clinical dermatology, 34(5), pp.e117-e119.

Correa-Martinez, C., Brentrup, A., Hess, K., Becker, K., Groll, A.H. and Schaumburg, F., 2018. First description of a local Coprinopsis cinerea skin and soft tissue infection. New Microbes and New Infections, 21, pp.102-104.

Crawford, S.J., Chen, S.A., Halliday, C., Rangan, G.K., Gottlieb, T. and Reid, A.B., 2015. M icrosphaeropsis arundinis skin and soft tissue infection in renal transplant recipients: three case reports and a review of the literature. *Transplant Infectious Disease*, *17*(6), pp.915-920.

Cunha, D., Amaro, C., Vieira, M.R., da Luz Martins, M., Maduro, A.P., Inácio, J., Afonso, A., Pinto, G.M. and Cardoso, J., 2012. Phaeohyphomycosis caused by Alternaria infectoria presenting as multiple vegetating lesions in a renal transplant patient. Revista Iberoamericana de Micología, 29(1), pp.44-46.

Da, Y., Vathsala, A. and Teo, R.Z.C., 2021. Invasive fungal infection by Volvariella volvacea: First reported case following solid organ transplantation and a unique therapeutic approach. Transplant Infectious Disease, 23(4), p.e13690.

Daboit, T.C., Magagnin, C.M., Heidrich, D., Castrillon, M.R., Mendes, S.D.C., Vettorato, G., Valente, P. and Scroferneker, M.L., 2013. A case of relapsed chromoblastomycosis due to Fonsecaea monophora: antifungal susceptibility and phylogenetic analysis. Mycopathologia, 176, pp.139-144.

Dalcin, D., Rothstein, A., Spinato, J., Escott, N. and Kus, J.V., 2016. Blastomyces gilchristii as cause of fatal acute respiratory distress syndrome. Emerging Infectious Diseases, 22(2), p.306.

Dananché, C., Cassier, P., Sautour, M., Gautheron, N., Wegrzyn, J., Perraud, M., Bienvenu, A.L., Nicolle, M.C., Boibieux, A. and Vanhems, P., 2015. Fungaemia caused by Fusarium proliferatum in a patient without definite immunodeficiency. Mycopathologia, 179(1), pp.135-140.

Davis, S.R., Ellis, D.H., Goldwater, P., Dimitriou, S. and Byard, R., 1994. First human culture-proven Australian case of entomophthoromycosis caused by Basidiobolus ranarum. Journal of medical and veterinary mycology, 32(3), pp.225-230.

Davis, C.M., Noroski, L.M., Dishop, M.K., Sutton, D.A., Braverman, R.M., Paul, M.E. and Rosenblatt, H.M., 2007. Basidiomycetous fungal Inonotus tropicalis sacral osteomyelitis in X-linked chronic granulomatous disease. The Pediatric infectious disease journal, 26(7), pp.655-656.

de Azevedo Bastos, V.R., de Castro Lima Santos, D.W., Padovan, A.C.B., Melo, A.S.A., de Abreu Mazzolin, M., Camargo, L.F.A. and Colombo, A.L., 2015. Early invasive pulmonary aspergillosis in a kidney transplant recipient caused by Aspergillus lentulus: first Brazilian report. Mycopathologia, 179, pp.299-305.

De Bernardis, F., Lorenzini, R., Verticchio, R., Agatensi, L. and Cassone, A., 1989. Isolation, acid proteinase secretion, and experimental pathogenicity of Candida parapsilosis from outpatients with vaginitis. Journal of clinical microbiology, 27(11), pp.2598-2603.

De Castro, N., Xu, F., Porcher, R., Pavie, J., Molina, J.M. and Peraldi, M.N., 2010. Pneumocystis jirovecii pneumonia in renal transplant recipients occurring after discontinuation of prophylaxis: a case–control study. Clinical microbiology and infection, 16(9), pp.1375-1377.

De Hoog, G.S., Mayser, P., Haase, G., Horré, R. and Horrevorts, A.M., 2000. A new species, Phialophora europaea, causing superficial infections in humans Eine neue Art, Phialophora europaea, als Erreger oberflächlicher Infektionen beim Menschen. Mycoses, 43(11-12), pp.409-416.

Degavre, B., Joujoux, J.M., Dandurand, M. and Guillot, B., 1997. First report of mycetoma caused by Arthrographis kalrae: successful treatment with itraconazole. Journal of the American Academy of Dermatology, 37(2), pp.318-320.

De Groote, M.A., Bjerke, R., Smith, H. and Rhodes III, L.V., 2000. Expanding epidemiology of blastomycosis: clinical features and investigation of 2 cases in Colorado. Clinical infectious diseases, 30(3), pp.582-584.

De Hoog, G.S., Buiting, A., Tan, C.S., Stroebel, A.B., Ketterings, C., De Boer, E.J., Naafs, B., Brimicombe, R., Nohlmans-Paulssen, M.K.E., Fabius, G.T.J. and Klokke, A.H., 1993.

De Macedo, P.M., Teixeira, M.D.M., Barker, B.M., Zancopé-Oliveira, R.M., Almeida-Paes, R. and Francesconi do Valle, A.C., 2019. Clinical features and genetic background of the sympatric species Paracoccidioides brasiliensis and Paracoccidioides americana. PLoS neglected tropical diseases, 13(4), p.e0007309.

De Miguel, D., Gómez, P., González, R., García-Suárez, J., Cuadros, J.A., Bañas, M.H., Romanyk, J. and Burgaleta, C., 2005. Nonfatal pulmonary Trichoderma viride infection in an adult patient with acute myeloid leukemia: report of one case and review of the literature. Diagnostic microbiology and infectious disease, 53(1), pp.33-37.

Desai, H.B., Perkins, P.L. and Procop, G.W., 2011. Granulomatous dermatitis due to Malassezia sympodialis. Archives of Pathology & Laboratory Medicine, 135(9), pp.1085-1087.

Diagnostic problems with imported cases of mycetoma in The Netherlands: Schwierigkeiten in der Diagnostik importierter Myzetome in den Niederlanden. Mycoses, 36(3-4), pp.81-87.

Didehdar, M., Gokanian, A., Sofian, M., Mohammadi, S., Mohammadi, R., Aslani, N., Haghani, I. and Badali, H., 2015. First fatal cerebral phaeohyphomycosis due to Rhinocladiella mackenziei in Iran, based on ITS rDNA. Journal de mycologie medicale, 25(1), pp.81-86.

de Andrade, T.S., de Almeida, A.M.Z., Basano, S.D.A., Takagi, E.H., Szeszs, M.W., Melhem, M.S., Albuquerque, M., Camargo, J.D.S.A.A., Gambale, W. and Camargo, L.M.A., 2020. Chromoblastomycosis in the Amazon region, Brazil, caused by Fonsecaea pedrosoi, Fonsecaea nubica, and Rhinocladiella similis: Clinicopathology, susceptibility, and molecular identification. Medical mycology, 58(2), pp.172-180.

Deak, L., Mudalagiriyappa, S., Ballin, A., Saxton, D. and Chakrabarti, A., 2018. A rhinofacial Conidiobolus coronatus fungal infection presenting as an intranasal tumour. Sultan Qaboos University Medical Journal, 18(4), p.e549.

Demaria, R.G., Dürrleman, N., Rispail, P., Margueritte, G., Macia, J.C., Aymard, T., Frapier, J.M., Albat, B. and Chaptal, P.A., 2000. Aspergillus flavus mitral valve endocarditis after lung abscess. The Journal of Heart Valve Disease, 9(6), pp.786-790.

Deroche, L., Buyck, J., Cateau, E., Rammaert, B., Marchand, S. and Brunet, K., 2022. Draft Genome Sequence of Kazachstania bovina Yeast Isolated from Human Infection. Mycopathologia, 187(4), pp.413-415.

Desmet, S., Smets, L., Lagrou, K., Derdelinckx, I., Neyt, J., Maertens, J., Sciot, R., Demaerel, P. and Bammens, B., 2016. Cladophialophora bantiana osteomyelitis in a renal transplant patient. Medical mycology case reports, 12, pp.17-20.

de Azevedo, C.M., Gomes, R.R., Vicente, V.A., Santos, D.W., Marques, S.G., do Nascimento, M.M., Andrade, C.E., Silva, R.R., Queiroz-Telles, F. and de Hoog, G.S., 2015. Fonsecaea pugnacius, a novel agent of disseminated chromoblastomycosis. Journal of clinical microbiology, 53(8), pp.2674-2685.

de Freitas, R.S., de Freitas, T.H.P., Siqueira, L.P.M., Gimenes, V.M.F. and Benard, G., 2019. First report of tinea corporis caused by Arthroderma benhamiae in Brazil. Brazilian Journal of Microbiology, 50, pp.985-987.

de Macedo, P.M., Almeida-Paes, R., de Medeiros Muniz, M., Oliveira, M.M.E., Zancopé-Oliveira, R.M., Costa, R.L.B. and do Valle, A.C.F., 2016. Paracoccidioides brasiliensis PS2: first autochthonous paracoccidioidomycosis case report in Rio de Janeiro, Brazil, and literature review. Mycopathologia, 181, pp.701-708.

de Souza, M., Matsuzawa, T., Lyra, L., Busso-Lopes, A.F., Gonoi, T., Schreiber, A.Z., Kamei, K., Moretti, M.L. and Trabasso, P., 2014. Fusarium napiforme systemic infection: case report with molecular characterization and antifungal susceptibility tests. Springerplus, 3(1), pp.1-8.

Dhindsa, M.K., Naidu, J., Singh, S.M. and Jain, S.K., 1995. Chronic suppurative otitis media caused by Paecilomyces variotii. Journal of medical and veterinary mycology, 33(1), pp.59-61.

Ding, Y., Steed, L.L. and Batalis, N., 2020. First reported case of disseminated Microascus gracilis infection in a lung transplant patient. IDCases, 22, p.e00984.

Diongue, K., Diallo, M.A., Seck, M.C., Ndiaye, M., Badiane, A.S., Diop, A., Ndiaye, Y.D., Ndir, O. and Ndiaye, D., 2016. Tinea pedis due to Cylindrocarpon lichenicola beginning onycholysis. Medical mycology case reports, 11, pp.13-15.

Dizbay, M., Adisen, E., Kustimur, S., Sari, N., Cengiz, B., Yalcin, B., Kalkanci, A., Gonul, I.I. and Sugita, T., 2009. Fungemia and cutaneous zygomycosis due to Mucor circinelloides in an intensive care unit patient: case report and review of literature. Jpn J Infect Dis, 62(2), pp.146-148.

Do Carmo, A., Costa, E., Marques, M., Quadrado, M.J. and Tomé, R., 2016. Fusarium dimerum species complex (Fusarium penzigii) keratitis after corneal trauma. Mycopathologia, 181(11), pp.879-884.

Dogan, M., Pabuçcuoglu, U., Sarioglu, S. and Yücesoy, M., 2004. Isolated nasopharyngeal aspergillosis caused by A flavus and associated with oxalosis. Ear, nose & throat journal, 83(5), pp.331-333.

Domniz, Y., Lawless, M., Sutton, G.L., Rogers, C.M. and Meagher, L.J., 2001. Successful treatment of Paecilomyces lilacinus endophthalmitis after foreign body trauma to the cornea. Cornea, 20(1), pp.109-111.

Dot, J.M., Debourgogne, A., Champigneulle, J., Salles, Y., Brizion, M., Puyhardy, J.M., Collomb, J., Plénat, F. and Machouart, M., 2009. Molecular diagnosis of disseminated adiaspiromycosis due to Emmonsia crescens. Journal of clinical microbiology, 47(4), pp.1269-1273.

Dotis, J., Panagopoulou, P., Filioti, J., Winn, R., Toptsis, C., Panteliadis, C. and Roilides, E., 2003. Femoral osteomyelitis due to Aspergillus nidulans in a patient with chronic granulomatous disease. Infection, 31(2), pp.121-124.

Doymaz, M.Z., Seyithanoglu, M.F., Hakyemez, İ., Gultepe, B.S., Cevik, S. and Aslan, T., 2015. A case of cerebral phaeohyphomycosis caused by Fonsecaea monophora, a neurotropic dematiaceous fungus, and a review of the literature. Mycoses, 58(3), pp.187-192.

Dubois, D., Pihet, M., Clec'h, C.L., Croué, A., Beguin, H., Bouchara, J.P. and Chabasse, D., 2005. Cutaneous phaeohyphomycosis due to Alternaria infectoria. Mycopathologia, 160, pp.117-123.

Dutra, V.R., Silva, L.F., Oliveira, A.N.M., Beirigo, E.F., Arthur, V.M., Bernardes da Silva, R., Ferreira, T.B., Andrade-Silva, L., Silva, M.V., Fonseca, F.M. and Silva-Vergara, M.L., 2020. Fatal case of fungemia by Wickerhamomyces anomalus in a pediatric patient diagnosed in a teaching hospital from Brazil. Journal of Fungi, 6(3), p.147.

Eaton, M.E., Padhye, A.A., Schwartz, D.A. and Steinberg, J.P., 1994. Osteomyelitis of the sternum caused by Apophysomyces elegans. Journal of Clinical Microbiology, 32(11), pp.2827-2828.

Ebeo, C.T., Olive, K., Byrd Jr, R.P., Mirle, G., Roy, T.M. and Mehta, J.B., 2002. Blastomycosis of the vocal folds with life-threatening upper airway obstruction: a case report. Ear, nose & throat journal, 81(12), pp.852-855.

Ebright, J.R., Chandrasekar, P.H., Marks, S., Fairfax, M.R., Aneziokoro, A. and McGinnis, M.R., 1999. Invasive sinusitis and cerebritis due to Curvularia clavata in an immunocompetent adult. Clinical infectious diseases, pp.687-689.

Echaiz, J.F., Burnham, C.A.D. and Bailey, T.C., 2013. A case of Apophysomyces trapeziformis necrotizing soft tissue infection. International Journal of Infectious Diseases, 17(12), pp.e1240-e1242.

Edupuganti, S., Rouphael, N., Mehta, A., Eaton, M., Heller, J.G., Bressler, A., Brandt, M. and O'Donnell, K., 2011. Fusarium falciforme vertebral abscess and osteomyelitis: case report and molecular classification. Journal of clinical microbiology, 49(6), pp.2350-2353.

Egli, A., Fuller, J., Humar, A., Lien, D., Weinkauf, J., Nador, R., Kapasi, A. and Kumar, D., 2012. Emergence of Aspergillus calidoustus infection in the era of posttransplantation azole prophylaxis. Transplantation, 94(4), pp.403-410.

Eickenberg, H.U., Amin, M. and Lich Jr, R., 1975. Blastomycosis of the genitourinary tract. The Journal of Urology, 113(5), pp.650-652.

Eisen, D.P., MacGinley, R., Christensson, B., Larsson, L. and Woods, M.L., 2000. Candida tropicalis vertebral osteomyelitis complicating epidural catheterisation with disease paralleled by elevated D-arabinitol/L-arabinitol ratios. European Journal of Clinical Microbiology and Infectious Diseases, 19(1), pp.61-63.

Ekhtiari, M., Farahyar, S., Falahati, M., Razmjou, E., Ashrafi-Khozani, M., Ghasemi, Z. and Abbasi-Nejat, Z., 2017. The first report of onychomycosis caused by Cryptococcus friedmannii (Naganishia friedmannii) a basidiomycetous yeast. Medical mycology case reports, 15, pp.25-27.

El-Ani, A.S., 1966. A new species of Leptosphaeria, an etiologic agent of mycetoma. Mycologia, 58(3), pp.406-411.

Elsawy, A., Faidah, H., Ahmed, A., Mostafa, A. and Mohamed, F., 2015. Aspergillus terreus meningitis in immunocompetent patient: a case report. Frontiers in Microbiology, 6, p.1353.

El-Sayed Ahmed, M.M., Almanfi, A., Aftab, M., Singh, S.K., Mallidi, H.R. and Frazier, O.H., 2015. Aspergillus mediastinitis after orthotopic heart transplantation: a case report. Texas Heart Institute Journal, 42(5), pp.468-470.

Emara, M., Ahmad, S., Khan, Z., Joseph, L., Al-Obaid, I.M., Purohit, P. and Bafna, R., 2015. Candida auris candidemia in Kuwait, 2014.

Enweani, I.B., Ozan, C.C., Agbonlahor, D.E. and Ndip, R.N., 1996. Dermatophytosis in schoolchildren in Ekpoma, Nigeria: Dermatophytosen bei Schulkindern in Ekpoma, Nigeria. Mycoses, 39(7-8), pp.303-305.

Eren, D., Eroglu, E., Ulu Kilic, A., Atalay, M.A., Mumcu, N., Sipahioglu, M.H., Canoz, O., Koc, A.N. and Oymak, O., 2018. Cutaneous ulcerations caused by Paecilomyces variotii in a renal transplant recipient. Transplant Infectious Disease, 20(3), p.e12871.

Ergene, U., Akcali, Z., Ozbalci, D., Nese, N. and Senol, S., 2013. Disseminated aspergillosis due to Aspergillus niger in immunocompetent patient: a case report. Case reports in infectious diseases, 2013.

Ersal, T., Al-Hatmi, A.S., Dalyan Cilo, B., Curfs-Breuker, I., Meis, J.F., Özkalemkaş, F., Ener, B. and van Diepeningen, A.D., 2015. Fatal disseminated infection with Fusarium petroliphilum. Mycopathologia, 179(1), pp.119-124.

Ertam, I., Aytimur, D. and Alper, S., 2007. Malassezia furfur onychomycosis in an immunosuppressed liver transplant recipient. Indian Journal of Dermatology, Venereology and Leprology, 73, p.425.

Esgin, H., Bulut, E. and Örüm, Ç., 2014. Candida pelliculosa endophthalmitis after cataract surgery: a case report. BMC Research Notes, 7(1), pp.1-3.

Esnakula, A.K., Summers, I. and Naab, T.J., 2013. Fatal disseminated Fusarium infection in a human immunodeficiency virus positive patient. Case reports in infectious diseases, 2013.

Fahal, A.H., Rahman, I.A., El-Hassan, A.M., Rahman, M.A.E. and Zijlstra, E.E., 2011. The safety and efficacy of itraconazole for the treatment of patients with eumycetoma due to Madurella mycetomatis. Transactions of the Royal Society of Tropical Medicine and Hygiene, 105(3), pp.127-132.

Fakih, M.G., Barden, G.E., Oakes, C.A. and Berenson, C.S., 1995. First reported case of Aspergillus granulosus infection in a cardiac transplant patient. Journal of Clinical Microbiology, 33(2), pp.471-473.

Fan, Y.M., Huang, W.M., Li, S.F., Wu, G.F., Li, W. and Chen, R.Y., 2009. Cutaneous phaeohyphomycosis of foot caused by Curvularia clavata. Mycoses, 52(6), pp.544-546.

Fanci, R., Pini, G., Bartolesi, A.M. and Pecile, P., 2013. Refractory disseminated fusariosis by Fusarium verticillioides in a patient with acute myeloid leukaemia relapsed after allogeneic hematopoietic stem cell transplantation: a case report and literature review. Revista Iberoamericana de Micología, 30(1), pp.51-53.

Farid, S., AbuSaleh, O., Liesman, R. and Sohail, M.R., 2017. Isolated cerebral mucormycosis caused by Rhizomucor pusillus. Case Reports, 2017, pp.bcr-2017.

Farina, C., Gotti, E., Parma, A., Naldi, L. and Goglio, A., 2007, June. Pheohyphomycotic soft tissue disease caused by Alternaria alternata in a kidney transplant patient: a case report and literature review. In Transplantation proceedings (Vol. 39, No. 5, pp. 1655-1659). Elsevier.

Fattahi, A., Sayyahfar, S., Lotfali, E., Ghasemi, R. and Mortezaeian, H., 2020. Aspergillus flavus endocarditis and meningitis in a child with marfan syndrome. Current Medical Mycology, 6(4), p.70.

Fattahi, A., Shirvani, F., Ayatollahi, A., Rezaei-Matehkolaei, A., Badali, H., Lotfali, E., Ghasemi, R., Pourpak, Z. and Firooz, A., 2021. Multidrug-resistant Trichophyton mentagrophytes genotype VIII in an Iranian family with generalized dermatophytosis: report of four cases and review of literature. International Journal of Dermatology, 60(6), pp.686-692.

Feldman, R., Cockerham, L., Buchan, B.W., Lu, Z. and Huang, A.M., 2016. Treatment of Paecilomyces variotii pneumonia with posaconazole: case report and literature review. Mycoses, 59(12), pp.746-750.

Feng, X., Ling, B., Yang, G., Yu, X., Ren, D. and Yao, Z., 2012. Prevalence and distribution profiles of Candida parapsilosis, Candida orthopsilosis and Candida metapsilosis responsible for superficial candidiasis in a Chinese university hospital. Mycopathologia, 173, pp.229-234.

Feng, P., Yin, S., Zhu, G., Li, M., Wu, B., Xie, Y., Ma, H., Zhang, J., Cheng, C., de Hoog, G.S. and Lu, C., 2015. Disseminated infection caused by Emmonsia pasteuriana in a renal transplant recipient. The Journal of Dermatology, 42(12), pp.1179-1182.

Fernández-García, O., Guerrero-Torres, L., Roman-Montes, C.M., Rangel-Cordero, A., Martínez-Gamboa, A., Ponce-de-Leon, A. and Gonzalez-Lara, M.F., 2021. Isolation of Rhizopus microsporus and Lichtheimia corymbifera from tracheal aspirates of two immunocompetent critically ill patients with COVID-19. Medical Mycology Case Reports, 33, pp.32-37.

Ferrer, C., Montero, J., Alió, J.L., Abad, J.L., Ruiz-Moreno, J.M. and Colom, F., 2003. Rapid molecular diagnosis of posttraumatic keratitis and endophthalmitis caused by Alternaria infectoria. Journal of Clinical Microbiology, 41(7), pp.3358-3360.

Fianchi, L., Picardi, M., Cudillo, L., Corvatta, L., Mele, L., Trape, G., Girmenia, C. and Pagano, L., 2004. Aspergillus niger infection in patients with haematological diseases: a report of eight cases. Mycoses, 47(3-4), pp.163-167.

Fike, J.M., Kollipara, R., Alkul, S. and Stetson, C.L., 2018. Case report of onychomycosis and tinea corporis due to Microsporum gypseum. Journal of Cutaneous Medicine and Surgery, 22(1), pp.94-96.

Fiona, L., Chew, M., Subrayan, V., Chong, P.P., Goh, M.C. and Ng, K.P., 2009. Cladosporium cladosporioides keratomycosis: a case report. *Japanese journal of ophthalmology*, *53*(6), p.657.

Fisher, D.J., CHRISTY, C., SPAFFORD, P., MANISCALCO, W.M., HARDY, D.J. and GRAMAN, P.S., 1993. Neonatal Trichosporon beigelii infection: report of a cluster of cases in a neonatal intensive care unit. The Pediatric infectious disease journal, 12(2), pp.149-155.

Fischer, N., Ruef, C., Ebnöther, C. and Bächli, E.B., 2008. Rhinofacial Conidiobolus coronatus infection presenting with nasal enlargement. Infection, 36(6), pp.594-596.

Fletcher, C.L., Hay, R.J., Midgley, G. and Moore, M., 1998. Onychomycosis caused by infection with Paecilomyces lilacinus. The British Journal of Dermatology, 139(6), pp.1133-1135.

Fletcher, C.L., Moore, M.K. and Hay, R.J., 2001. Endonyx onychomycosis due to Trichophyton soudanense in two Somalian siblings. British Journal of Dermatology, 145(4), pp.687-688.

Florescu, D.F., Iwen, P.C., Hill, L.A., Dumitru, I., Quader, M.A., Kalil, A.C. and Freifeld, A.G., 2009. Cerebral aspergillosis caused by Aspergillus ustus following orthotopic heart transplantation: case report and review of the literature. Clinical transplantation, 23(1), pp.116-120.

Ford, J.G., Agee, S. and Greenhaw, S.T., 2008. Successful medical treatment of a case of Paecilomyces lilacinus keratitis. Cornea, 27(9), pp.1077-1079.

Framil, V.M.D.S., Melhem, M.S., Szeszs, M.W., Corneta, E.C. and Zaitz, C., 2010. Pityriasis versicolor circinata: isolation of Malassezia sympodialis-Case report. Anais Brasileiros de Dermatologia, 85, pp.227-228.

Friedman, J.A., Wijdicks, E.F., Fulgham, J.R. and Wright, A.J., 2000, April. Meningoencephalitis due to Blastomyces dermatitidis: case report and literature review. In Mayo Clinic Proceedings (Vol. 75, No. 4, pp. 403-408). Elsevier.

Gabriel, F., Noel, T. and Accoceberry, I., 2012. Lindnera (Pichia) fabianii blood infection after mesenteric ischemia. Medical Mycology, 50(3), pp.310-314.

Gacser, A., Salomon, S., Schafer, W., 2005. Direct transformation of an clinical isolate of Candida parapsilosis using a dominant selection marker. FEMS Microbiol. Lett. 245, 117–121.

Gácser, A., Schäfer, W., Nosanchuk, J.S., Salomon, S. and Nosanchuk, J.D., 2007. Virulence of Candida parapsilosis, Candida orthopsilosis, and Candida metapsilosis in reconstituted human tissue models. Fungal Genetics and Biology, 44(12), pp.1336-1341.

Gaisne, R., Jeddi, F., Morio, F., Le Clerc, Q.C., Hourmant, M., Blancho, G., Giral, M., Cantarovich, D., Dantal, J. and Ville, S., 2018. Candida utilis fungaemia following endoscopic intervention on ureteral stent in a kidney transplant recipient: Case report and a review of the literature. Mycoses, 61(8), pp.594-599.

Gaitán, A.C.R., Moret, A., Hontangas, J.L.L., Molina, J.M., López, A.I.A., Cabezas, A.H., Maseres, J.M., Arcas, R.C., Ruiz, M.D.G., Chiveli, M.Á. and Cantón, E., 2017. Nosocomial fungemia by Candida auris: first four reported cases in continental Europe. Revista iberoamericana de micologia, 34(1), pp.23-27.

Gallotti, A.C., Lombera, M., Pinto, K., Pinilla, I., Zaragoza, O. and Cuétara, M.S., 2023. Kazachstania slooffiae, an emerging pathogen to watch for in humans?. Medical Mycology Case Reports, 42, p.100604.

Galván Ledesma, A., Rodríguez Maqueda, M. and Talego Sancha, A., 2022. Wickerhamomyces Anomalus Postoperative Endophthalmitis. Ocular Immunology and Inflammation, pp.1-3.

Garbino, J., Ondrusova, A., Baligvo, E., Lew, D., Bouchuiguir-Wafa, K. and Rohner, P., 2002. Successful treatment of Paecilomyces lilacinus endophthalmitis with voriconazole. Scandinavian journal of infectious diseases, 34(9), pp.701-703.

Garey, K.W., Pendland, S.L., Van Huynh, T., Bunch, T.H., Jensen, G.M. and Pursell, K.J., 2001. Cunninghamella bertholletiae infection in a bone marrow transplant patient: amphotericin lung penetration, MIC determinations, and review of the literature. Pharmacotherapy: The Journal of Human Pharmacology and Drug Therapy, 21(7), pp.855-860.

Gautheret, A., Dromer, F., Bourhis, J.H. and Andremont, A., 1995. Trichoderma pseudokoningii as a cause of fatal infection in a bone marrow transplant recipient. Clinical Infectious Diseases, 20(4), pp.1063-1064.

Gavin, P.J., Sutton, D.A. and Katz, B.Z., 2002. Fatal endocarditis in a neonate caused by the dematiaceous fungus Phialemonium obovatum: case report and review of the literature. *Journal of clinical microbiology*, *40*(6), pp.2207-2212.

Gene, J., Azon-Masoliver, A., Guarro, J., Ballester, F., Pujol, I., Llovera, M. and Ferrer, C., 1995. Cutaneous phaeohyphomycosis caused by Alternaria longipes in an immunosuppressed patient. Journal of clinical microbiology, 33(10), pp.2774-2776.

Geiger, A.M., Foxman, B. and Sobel, J.D., 1995. Chronic vulvovaginal candidiasis: characteristics of women with Candida albicans, C glabrata and no candida. Sexually Transmitted Infections, 71(5), pp.304-307.

Gené, J., Azón-Masoliver, A., Guarro, J., De Febrer, G., Martínez, A., Grau, C., Ortoneda, M. and Ballester, F., 2001. Cutaneous infection caused by Aspergillus ustus, an emerging opportunistic fungus in immunosuppressed patients. Journal of Clinical Microbiology, 39(3), pp.1134-1136.

Gharib[1], S.J., IBRAHIM, D.R. and ABDULLAH, S.K., Molecular Identification of two Isolates of Nannizia Fulva Causing Tinea Capitis in Iraq. Pakistan Journal of Medical & Health Sciences, 16(1), 735-738

Gharehbolagh, S.A., Nasimi, M., Afshari, S.A.K., Ghasemi, Z. and Rezaie, S., 2017. First case of superficial infection due to Naganishia albida (formerly Cryptococcus albidus) in Iran: A review of the literature. Current Medical Mycology, 3(2), p.33.

Ghilardi, A., Massai, L., Gallo, A., Paccagnini, E. and Romano, C., 2007. Tinea capitis due to Trichophyton soudanense mimicking bacterial folliculitis. Mycoses, 50(2), pp.150-152.

Ghorpade, A., Anitha, V. and Ravindran, M., 2022. Colletotrichum asianum: A Novel Phytopathogen Causing Human Infection—A Case Report. Cornea, 41(11), pp.1458-1461.

Gip, L., 1994. Black piedra: the first case treated with terbinafine (Lamisil®). British Journal of Dermatology, 130(s43), pp.26-28.

Gits-Muselli, M., Benderdouche, M., Hamane, S., Mingui, A., Feuilhade de Chauvin, M., Guigue, N., Picat, M.Q., Bourrat, E., Petit, A., Bagot, M. and Alanio, A., 2017. Continuous increase of Trichophyton tonsurans as a cause of tinea capitis in the urban area of Paris, France: a 5-year-long study. Medical mycology, 55(5), pp.476-484.

Gkegkes, I.D., Kotrogiannis, I., Konstantara, F., Karetsou, A., Tsiplakou, S., Fotiou, E., Stamopoulou, S., Papazacharias, C. and Paraskevopoulos, I.A., 2019. Cutaneous mucormycosis by Saksenaea vasiformis: an unusual case report and review of literature. Mycopathologia, 184, pp.159-167.

Gnat, S., Łagowski, D., Nowakiewicz, A. and Dyląg, M., 2020. Unusual dermatomycoses caused by Nannizzia nana: the geophilic origin of human infections. Infection, 48, pp.429-434.

Godet, C., Cateau, E., Rammaert, B., Grosset, M., Le Moal, G., Béraud, G., Martellosio, J.P., Iriart, X., Cadranel, J. and Roblot, F., 2017. Nebulized liposomal amphotericin B for treatment of pulmonary infection caused by Hormographiella aspergillata: case report and literature review. Mycopathologia, 182, pp.709-713.

Godoy, P., Nunes, F., Silva, V., Tomimori-Yamashita, J., Zaror, L. and Fischman, O., 2004. Onychomycosis caused by Fusarium solani and Fusarium oxysporum in São Paulo, Brazil. Mycopathologia, 157(3), pp.287-290.

Gompf, S.G., Paredes, A., Quilitz, R., Greene, J.N., Hiemenz, J.W. and Sandin, R.L., 1999. Paecilomyces lilacinus osteomyelitis in a bone marrow transplant patient. INFECTIONS IN MEDICINE, 16(11), pp.766-+.

Gong, J.Q., Liu, X.Q., Xu, H.B., Zeng, X.S., Chen, W. and Li, X.F., 2007. Deep dermatophytosis caused by Trichophyton rubrum: report of two cases. Mycoses, 50(2), pp.102-108.

González, G.M., Rojas, O.C., González, J.G., Kang, Y. and De Hoog, G.S., 2013. Chromoblastomycosis caused by Rhinocladiella aquaspersa. Medical mycology case reports, 2, pp.148-151.

Gorgievska-Sukarovska, B., Skerlev, M., Žele-Starčević, L., Husar, K. and Halasz, M., 2017. Kerion Celsi due to Microsporum canis with dermatophytide reaction. Acta Dermatovenerologica Croatica, 25(2), pp.151-151.

Gorman, S.R., Magiorakos, A.P., Zimmerman, S.K. and Craven, D.E., 2006. Fusarium oxysporum pneumonia in an immunocompetent host. Southern medical journal, 99(6), pp.613-617.

Govender, N.P., Magobo, R.E., Mpembe, R., Mhlanga, M., Matlapeng, P., Corcoran, C., Govind, C., Lowman, W., Senekal, M. and Thomas, J., 2018. Candida auris in South Africa, 2012–2016. Emerging infectious diseases, 24(11), p.2036.

Gompertz, O.F., Rodrigues, A.M., Fernandes, G.F., Bentubo, H.D., de Camargo, Z.P. and Petri, V., 2016. Case report: atypical clinical presentation of sporotrichosis caused by Sporothrix globosa resistant to itraconazole. The American journal of tropical medicine and hygiene, 94(6), p.1218.

Govender, N.P. and Grayson, W., 2019. Emergomycosis (Emergomyces africanus) in advanced HIV disease. Dermatopathology, 6(2), pp.63-69.

Garcia-Covarrubias, L., Bartlett, R., Barratt, D.M. and Wassermann, R.J., 2001. Rhino-orbitocerebral mucormycosis attributable to Apophysomyces elegans in an immunocompetent individual: case report and review of the literature. Journal of Trauma and Acute Care Surgery, 50(2), pp.353-357.

Gast, K.B., van der Hoeven, A., de Boer, M.G., van Esser, J.W., Kuijper, E.J., Verweij, J.J., van Keulen, P.H. and van der Beek, M.T., 2019. Two cases of Emergomyces pasteurianus infection in immunocompromised patients in the Netherlands. Medical mycology case reports, 24, pp.5-8.

Grava, S., Lopes, F.A.D., Cavallazzi, R.S., Grassi, M.F.N.N. and Svidzinski, T.I.E., 2016. A rare case of hemorrhagic pneumonia due to Cladosporium cladosporioides. Jornal Brasileiro De Pneumologia, 42, pp.392-394.

Grigoryan, K.V., Tollefson, M.M., Olson, M.A. and Newman, C.C., 2019. Pediatric tinea capitis caused by Trichophyton violaceum and Trichophyton soudanense in Rochester, Minnesota, United States. International journal of dermatology, 58(8), pp.912-915.

GŘler, N.C., Tosun, İ.L.K.N.U.R. and Aydin, F.A.R.U.K., 2017. The identification of Meyerozyma guilliermondii from blood cultures and surveillance samples in a university hospital in Northeast Turkey: A ten-year survey. Journal de Mycologie MÚdicale, 27(4), pp.506-513.

Grossi, O., Pineau, S., Sadot-Lebouvier, S., Hay, B., Delaunay, J., Miailhe, A.F., Bretonniere, C., Jeddi, F., Lavergne, R.A. and Le Pape, P., 2019. Disseminated mucormycosis due to Lichtheimia corymbifera during ibrutinib treatment for relapsed chronic lymphocytic leukaemia: a case report. Clinical Microbiology and Infection, 25(2), pp.261-263.

Guppy, K.H., Thomas, C., Thomas, K. and Anderson, D., 1998. Cerebral fungal infections in the immunocompromised host: a literature review and a new pathogen-Chaetomium atrobrunneum: case report. *Neurosurgery*, *43*(6), pp.1463-1468.

Gupta, P., Kaur, H., Dwivedi, S., Agnihotri, S. and Rudramurthy, S.M., 2022. First case of Tropicoporus tropicalis keratitis in an immunocompetent host from India and review of the literature. Journal of Medical Mycology, 32(1), p.101205.

Guarro, J., Antolín-Ayala, M.I., Gené, J., Gutiérrez-Calzada, J., Nieves-Díez, C. and Ortoneda, M., 1999. Fatal case of Trichoderma harzianum infection in a renal transplant recipient. Journal of clinical microbiology, 37(11), pp.3751-3755.

Guarro, J., Nucci, M., Akiti, T., Gené, J., Barreiro, M.D.G.C. and Gonçalves, R.T., 2000. Fungemia due to Fusarium sacchari in an immunosuppressed patient. Journal of clinical microbiology, 38(1), pp.419-421.

Guarro, J., Svidzinski, T.E., Zaror, L., Forjaz, M.H., Gené, J. and Fischman, O., 1998. Subcutaneous hyalohyphomycosis caused by Colletotrichum gloeosporioides. Journal of Clinical Microbiology, 36(10), pp.3060-3065.

Guarro, J., Akiti, T., Horta, R.A., Morizot Leite-Filho, L.A., Gené, J., Ferreira-Gomes, S., Aguilar, C. and Ortoneda, M., 1999. Mycotic keratitis due to Curvularia senegalensis and in vitro antifungal susceptibilities of Curvularia spp. Journal of clinical microbiology, 37(12), pp.4170-4173.

Guarro, J., Mayayo, E., Tapiol, J., Aguilar, C. and Cano, J., 1999. Microsphaeropsis olivacea as an etiological agent of human skin infection. *Medical Mycology*, *37*(2), pp.133-137.

Guccion, J.G., Rohatgi, P.K., Saini, N.B., French, A., Tavaloki, S. and Barr, S., 1996. Disseminated blastomycosis and acquired immunodeficiency syndrome: a case report and ultrastructural study. Ultrastructural pathology, 20(5), pp.429-435.

H. C. Gugnani, V. Ramesh, N. Sood, J. Guarro, Moin-Ul-Haq, A. Paliwal-Joshi, B. Singh & R. Makkar (2006) Cutaneous phaeohyphomycosis caused by Cladosporium oxysporum and its treatment with potassium iodide, Medical Mycology, 44:3, 285-288, DOI: 10.1080/13693780500294824

Guo, J., Zhang, M., Qiao, D., Shen, H., Wang, L., Wang, D., Li, L., Liu, Y., Lu, H., Wang, C. and Ding, H., 2021. Prevalence and Antifungal Susceptibility of Candida parapsilosis Species Complex in Eastern China: A 15-Year Retrospective Study by ECIFIG. Frontiers in microbiology, 12, p.644000.

Gupta, N., Kumar, A., Singh, G., Ratnakar, G., Vinod, K.S. and Wig, N., 2017. Breakthrough mucormycosis after voriconazole use in a case of invasive fungal rhinosinusitis due to Curvularia lunata. Drug Discoveries & Therapeutics, 11(6), pp.349-352.

Gürcan, Ş., Tikveşli, M., Üstündağ, S. and Beyza, E.N.E.R., 2013. A case report on Aspergillus lentulus pneumonia. Balkan medical journal, 2013(4), pp.429-431.

Gyotoku, H., Izumikawa, K., Ikeda, H., Takazono, T., Morinaga, Y., Nakamura, S., Imamura, Y., Nishino, T., Miyazaki, T., Kakeya, H. and Yamamoto, Y., 2012. A case of bronchial aspergillosis caused by Aspergillus udagawae and its mycological features. Medical Mycology, 50(6), pp.631-636.

Hadaschik, E., Koschny, R., Willinger, B., Hallscheidt, P., Enk, A. and Hartschuh, W., 2012. Pulmonary, rhino-orbital and cutaneous mucormycosis caused by Rhizomucor pusillus in an immunocompromised patient. Clinical and experimental dermatology, 37(4), pp.355-357.

Hahn, R.C., Rodrigues, A.M., Fontes, C.J.F., Nery, A.F., Tadano, T., Júnior, L.D.P.Q. and de Camargo, Z.P., 2014. Case report: Fatal fungemia due to Paracoccidioides lutzii. The American journal of tropical medicine and hygiene, 91(2), p.394.

Hajjeh, R.A. and Blumberg, H.M., 1995. Bloodstream infection due to Trichosporon beigelii in a burn patient: case report and review of therapy. Clinical infectious diseases, 20(4), pp.913-916.

Halim, I., Singh, P., Sarfraz, A., Kokkayil, P., Pati, B.K., Thakuria, B. and Raj, A., 2021. Fungal Keratitis Due to Fusarium lichenicola: A Case Report and Global Review of Fusarium lichenicola Keratitis. Journal of Fungi, 7(11), p.889.

Hamal, P., Ostransky, J., Dendis, M., Horváth, R., Ruzicka, F., Buchta, V., Vejsova, M., Sauer, P., Hejnar, P. and Raclavsky, V., 2008. A case of endocarditis caused by the yeast Pichia fabianii with biofilm production and developed in vitro resistance to azoles in the course of antifungal treatment. Sabouraudia, 46(6), pp.601-605.

Hardjasudarma, M., Willis, B., Black-Payne, C. and Edwards, R., 1995. Pediatric spinal blastomycosis: case report. Neurosurgery, 37(3), pp.534-536.

Harris, J.E., Sutton, D.A., Rubin, A., Wickes, B., De Hoog, G.S. and Kovarik, C., 2009. Exophiala spinifera as a cause of cutaneous phaeohyphomycosis: case study and review of the literature. Medical mycology, 47(1), pp.87-93.

Hazen, K.C., Theisz, G.W. and Howell, S.A., 1999. Chronic urinary tract infection due to Candida utilis. Journal of clinical microbiology, 37(3), pp.824-827.

Heslop, O.D., De Ceulaer, K., Rainford, L. and Nicholson, A.M., 2015. A case of Candida orthopsilosis associated septic arthritis in a patient with Systemic Lupus Erythematosus (SLE). Medical mycology case reports, 7, pp.1-3.

Hecker, M.S., Weinberg, J.M., Bagheri, B., Tangoren, I.A. and Rudikoff, D., 1997. Cutaneous Paecilomyces lilacinus infection: report of two novel cases. Journal of the American Academy of Dermatology, 37(2), pp.270-271.

Heiblig, M., Bozzoli, V., Saison, J., Thomas, X., De Croze, D., Traverse-Glehen, A., Cosmidis, A., Chidiac, C., Ferry, T., Alanio, A. and Bienvenu, A.L., 2015. Combined medico-surgical strategy for invasive sino-orbito-cerebral breakthrough fungal infection with Hormographiella aspergillata in an acute leukaemia patient. Mycoses, 58(5), pp.308-312.

Heidrich, D., González, G.M., Pagani, D.M., Ramírez-Castrillón, M. and Scroferneker, M.L., 2017. Chromoblastomycosis caused by Rhinocladiella similis: Case report. Medical mycology case reports, 16, pp.25-27.

Hall, V.C., Goyal, S., Davis, M.D. and Walsh, J.S., 2004. Cutaneous hyalohyphomycosis caused by Paecilomyces lilacinus: report of three cases and review of the literature. International journal of dermatology, 43(9), pp.648-653.

Hallur, V., Prakash, H., Sable, M., Preetam, C., Purushotham, P., Senapati, R., Shankarnarayan, S.A., Bag, N.D. and Rudramurthy, S.M., 2021. Cunninghamella arunalokei a new species of Cunninghamella from India causing disease in an immunocompetent individual. Journal of Fungi, 7(8), p.670.

Hawkes, M., Rennie, R., Sand, C. and Vaudry, W., 2005. Aureobasidium pullulans infection: fungemia in an infant and a review of human cases. *Diagnostic microbiology and infectious disease*, *51*(3), pp.209-213.

He, D., Quan, M., Zhong, H., Chen, Z., Wang, X., He, F., Qu, J., Zhou, T., Lv, X. and Zong, Z., 2021. Emergomyces orientalis emergomycosis diagnosed by metagenomic next-generation sequencing. Emerging Infectious Diseases, 27(10), p.2740.

Hemashettar, B.M., Patil, R.N., O'Donnell, K., Chaturvedi, V., Ren, P. and Padhye, A.A., 2011. Chronic rhinofacial mucormycosis caused by Mucor irregularis (Rhizomucor variabilis) in India. Journal of Clinical Microbiology, 49(6), pp.2372-2375.

Hemo, I., Pe, J. and Polacheck, I., 1989. Fusarium oxysporum keratitis. Ophthalmologica, 198(1), pp.3-7.

Hennequin, C., Bouree, P., Hiesse, C., Dupont, B. and Charpentier, B., 1996. Spondylodiskitis due to Candida albicans: report of two patients who were successfully treated with fluconazole and review of the literature. Clinical infectious diseases, 23(1), pp.176-178.

Hennequin, C., Chouaki, T., Pichon, J.C., Strunski, V. and Raccurt, C., 2000. Otitis externa due to Trichoderma longibrachiatum. European Journal of Clinical Microbiology and Infectious Diseases, 19(8), p.641.

Herbrecht, R., Kessler, R., Kravanja, C., Meyer, M.H., Waller, J. and Letscher-Bru, V., 2004. Successful treatment of Fusarium proliferatum pneumonia with posaconazole in a lung transplant recipient. The Journal of heart and lung transplantation, 23(12), pp.1451-1454.

Hernández-Hernández, F., Vargas-Arzola, J., Ríos-Cruz, O.P., Córdova-Martínez, E., Manzano-Gayosso, P. and Segura-Salvador, A., 2018. First case of chromoblastomycosis due to Phoma insulana. Enfermedades infecciosas y microbiologia clinica (English ed.), 36(2), pp.95-99.

Heslop, O.D., De Ceulaer, K., Rainford, L. and Nicholson, A.M., 2015. A case of Candida orthopsilosis associated septic arthritis in a patient with Systemic Lupus Erythematosus (SLE). Medical mycology case reports, 7, pp.1-3.

Hilmioglu, S., Metin, D.Y., Tasbakan, M., Pullukcu, H., Akalin, T. and Tumbay, E., 2015. Skin infection on both legs caused by Acremonium strictum (case report). Annals of Saudi Medicine, 35(5), pp.406-408.

Hipolito, E., Faria, E., Alves, A.F., De Hoog, G.S., Anjos, J., Gonçalves, T., Morais, P.V. and Estevão, H., 2009. Alternaria infectoria brain abscess in a child with chronic granulomatous disease. European journal of clinical microbiology & infectious diseases, 28, pp.377-380.

Hitoto, H., Pihet, M., Weil, B., Chabasse, D., Bouchara, J.P. and Rachieru-Sourisseau, P., 2010. Acremonium strictum fungaemia in a paediatric immunocompromised patient: diagnosis and treatment difficulties. Mycopathologia, 170(3), pp.161-164.

Holley, K., Muldoon, M. and Tasker, S., 2002. Coccidioides immitis osteomyelitis: a case series review. Orthopedics, 25(8), pp.827-832.

Hood, S.V., Moore, C.B., Cheesbrough, J.S., Mene, A. and Denning, D.W., 1997. Atypical eumycetoma caused by Phialophora parasitica successfully treated with itraconazole and flucytosine. British Journal of Dermatology, 136(6), pp.953-956.

Hoenigl, M., Aspeck, E., Valentin, T., Heiling, B., Seeber, K., Krause, R., Stammberger, H., Beham, A. and Buzina, W., 2013. Sinusitis and frontal brain abscess in a diabetic patient caused by the basidiomycete Schizophyllum commune: case report and review of the literature. Mycoses, 56(3), pp.389-393.

Höfling-Lima, A.L., Guarro, J., Freitas, D.D., Godoy, P., Gené, J., Souza, L.B.D., Zaror, L. and Romano, A.C., 2005. Clinical treatment of corneal infection due to Fonsecaea pedrosoi: case report. Arquivos brasileiros de oftalmologia, 68, pp.270-272.

Homa, M., Manikandan, P., Saravanan, V., Revathi, R., Anita, R., Narendran, V., Panneerselvam, K., Shobana, C.S., Aidarous, M.A., Galgóczy, L. and Vágvölgyi, C., 2018. Exophiala dermatitidis endophthalmitis: case report and literature review. Mycopathologia, 183, pp.603-609.

Hospenthal, D.R., Chung, K.K., Lairet, K., Thompson, E.H., Guarro, J., Renz, E.M. and Sutton, D.A., 2011. Saksenaea erythrospora infection following combat trauma. Journal of clinical microbiology, 49(10), pp.3707-3709.

Houaida, T., Sourour, N., Ines, H., Imen, F., Moez, M., Nahed, K., Hayet, S., Fattouma, M., Moez, E. and Ali, A., 2022. A case report of a mucormycosis caused by Lichtheimia ramosa in a patient with acute myeloid leukemia. Reviews in Medical Microbiology, 33(1), pp.e4-e9.

Hounchonou, F.H., Runge, J., Ganser, A., Hartmann, C., Raab, P. and Krauss, J.K., 2022. Stereotactic biopsy of a brain lesion caused by hormographiella aspergillata. Surgical Neurology International, 13(596), p.1.

Howard, S.J., Walker, S.L., Andrew, S.M., Borman, A.M., Johnson, E.M. and Denning, D.W., 2006. Sub-cutaneous phaeohyphomycosis caused by Cladophialophora devriesii in a United Kingdom resident. Medical Mycology, 44(6), pp.553-556.

Hsieh, T.T., Tseng, H.K., Sun, P.L., Wu, Y.H. and Chen, G.S., 2013. Disseminated zygomycosis caused by Cunninghamella bertholletiae in patient with hematological malignancy and review of published case reports. Mycopathologia, 175, pp.99-106.

Hu, L., Wang, S., Sun, D., Chu, J., Lai, B. and Liu, Y., 2021. First case of bloodstream infection of Trichosporon loubieri in a patient with B-cell lymphocytic leukemia in China. Journal of Infection and Chemotherapy, 27(1), pp.86-89.

Huang, W.M., Fan, Y.M., Li, W. and Yang, W.W., 2011. Brain abscess caused by Cladophialophora bantiana in China. Journal of medical microbiology, 60(12), pp.1872-1874.

Huang, C., Peng, Y., Zhang, Y., Li, R., Wan, Z. and Wang, X., 2019. Deep dermatophytosis caused by Trichophyton rubrum. The Lancet Infectious Diseases, 19(12), p.1380.

Huang, C., Zhang, Y., Song, Y., Wan, Z., Wang, X. and Li, R., 2019. Phaeohyphomycosis caused by Phialophora americana with CARD9 mutation and 20-year literature review in China. Mycoses, 62(10), pp.908-919.

Huang, C.Y., Peng, C.C., Hsu, C.H., Chang, J.H., Chiu, N.C. and Chi, H., 2020. Systemic infection caused by Malassezia pachydermatis in infants: Case series and review of the literature. The Pediatric infectious disease journal, 39(5), pp.444-448.

Huang, Y.T., Liaw, S.J., Liao, C.H., Yang, J.L., Lai, D.M., Lee, Y.C. and Hsueh, P.R., 2008. Catheter-related septicemia due to Aureobasidium pullulans. *International Journal of Infectious Diseases*, *12*(6), pp.e137-e139.

Hubka, V., Dobiášová, S., Dobiáš, R. and Kolařík, M., 2014. Microsporum aenigmaticum sp. nov. from M. gypseum complex, isolated as a cause of tinea corporis. Sabouraudia, 52(4), pp.387-396.

Hubka, V., Mencl, K., Skorepova, M., Lyskova, P. and Zalabska, E., 2011. Phaeohyphomycosis and onychomycosis due to Chaetomium spp., including the first report of Chaetomium brasiliense infection. *Medical Mycology*, *49*(7), pp.724-733.

Husain, S., Muñoz, P., Forrest, G., Alexander, B.D., Somani, J., Brennan, K., Wagener, M.M. and Singh, N., 2005. Infections due to Scedosporium apiospermum and Scedosporium prolificans in transplant recipients: clinical characteristics and impact of antifungal agent therapy on outcome. Clinical Infectious Diseases, 40(1), pp.89-99.

Iwasawa, M., Yorifuji, K., Sano, A., Takahashi, Y. and Nishimura, K., 2009. A case of kerion celsi caused by Microsporum gypseum (Arthroderma gypseum) in a child. Nippon Ishinkin Gakkai Zasshi, 50(3), pp.155-160.

Iwasawa, M.T., Togawa, Y., Akita, F., Kambe, N., Matsue, H., Yaguchi, T. and Nishimura, K., 2012. Kerion celsi due to Arthroderma incurvatum infection in a Sri Lankan child: species identification and analysis of area-dependent genetic polymorphism. Medical Mycology, 50(7), pp.690-698.

Izumi, K., Hisata, Y. and Hazama, S., 2009. A rare case of infective endocarditis complicated by Trichosporon asahii fungemia treated by surgery. Annals of thoracic and cardiovascular surgery: official journal of the Association of Thoracic and Cardiovascular Surgeons of Asia, 15(5), pp.350-353.

Irek, E.O., Obadare, T.O., Udonwa, P.A., Laoye, O., Abiri, O.V., Adeoye, A.O. and Aboderin, A.O., 2017. Cylindrocarpon lichenicola keratomycosis in Nigeria: the challenge of limited access to effective antimicrobials. African Journal of Laboratory Medicine, 6(1), pp.1-3.

Isabel Cristina, R.S., Diana, A. and Karen, A., 2020. Breakthrough Hormographiella aspergillata infection in a patient with acute myeloid leukemia receiving posaconazole prophylaxis: a case report and review. Mycopathologia, 185, pp.1069-1076.

Irles, D., Bonadona, A., Pofelski, J., Laramas, M., Molina, L., Lantuejoul, S., Brenier-Pinchart, M.P., Bagueta, J.P. and Barnoud, D., 2004. Aspergillus flavus endocarditis on a native valve. Archives des Maladies du Coeur et des Vaisseaux, 97(2), pp.172-175.

Istorico, L.J., Sanders, M., Jacobs, R.F., Gilleon, S., Glasier, C. and Bradsher, R.W., 1992. Otitis media due to blastomycosis: report of two cases. Clinical infectious diseases, 14(1), pp.355-358.

Iwen, P.C., Rupp, M.E., Langnas, A.N., Reed, E.C. and Hinrichs, S.H., 1998. Invasive pulmonary aspergillosis due to Aspergillus terreus: 12-year experience and review of the literature. Reviews of Infectious Diseases, 26(5), pp.1092-1097.

Iwen, P.C., Tarantolo, S.R., Sutton, D.A., Rinaldi, M.G. and Hinrichs, S.H., 2000. Cutaneous infection caused by Cylindrocarpon lichenicola in a patient with acute myelogenous leukemia. Journal of clinical microbiology, 38(9), pp.3375-3378.

Iwen, P.C., Freifeld, A.G., Sigler, L. and Tarantolo, S.R., 2005. Molecular identification of Rhizomucor pusillus as a cause of sinus-orbital zygomycosis in a patient with acute myelogenous leukemia. Journal of clinical microbiology, 43(11), pp.5819-5821.

Iwen, P.C., Sigler, L., Noel, R.K. and Freifeld, A.G., 2007. Mucor circinelloides was identified by molecular methods as a cause of primary cutaneous zygomycosis. Journal of Clinical Microbiology, 45(2), pp.636-640.

Jacobs, F., Byl, B., Bourgeois, N., Coremans-Pelseneer, J., Florquin, S., Depre, G., Van De Stadt, J., Adler, M., Gelin, M. and Thys, J.P., 1992. Trichoderma viride infection in a liver transplant recipient: Infektion eines Lebertransplantierten durch Trichoderma viride. Mycoses, 35(11-12), pp.301-303.

Jain, N., Jinagal, J., Kaur, H., Ghosh, A., Gupta, S., Ram, J. and Rudramurthy, S.M., 2019. Ocular infection caused by Hormographiella aspergillata: a case report and review of literature. Journal de Mycologie Médicale, 29(1), pp.71-74.

James, E.A., Orchard, K., McWhinney, P.H.W., Warnock, D.W., Johnson, E.M., Mehta, A.B. and Kibbler, C.C., 1997. Disseminated infection due to Cylindrocarpon lichenicola in a patient with acute myeloid leukemia. Journal of Infection, 34(1), pp.65-67.

James, M.J., Lasker, B.A., McNeil, M.M., Shelton, M., Warnock, D.W. and Reiss, E., 2000. Use of a repetitive DNA probe to type clinical and environmental isolates of Aspergillus flavus from a cluster of cutaneous infections in a neonatal intensive care unit. Journal of Clinical Microbiology, 38(10), pp.3612-3618.

JANG, B.S., JO, J.H., OH, C.K., JANG, H.S. and KWON, K.S., 2002. A case of kerion celsi caused by Trichophyton mentagrophytes. Korean Journal of Medical Mycology, pp.86-91.

Jaramillo, S. and Varon, C.L., 2013. Curvularia lunata endophthalmitis after penetrating ocular trauma. Retinal Cases and Brief Reports, 7(4), pp.315-318.

Jaspers, G.J., Werrij, B.G., Jagtman, B.A. and Loza, B., 2011. Severe kerion celsi due to Trichophyton mentagrophytes: a case report. Acta Paediatrica, 100(10), pp.e181-e183.

Jayasinghe R. D., W. A. M. U. L. Abeysinghe, P. I. Jayasekara, Y. S. Mohomed, B. S. M. S. Siriwardena, "Unilateral Cervical Lymphadenopathy due to *Cladosporium oxysporum*: A Case Report and Review of the Literature", *Case Reports in Pathology*, vol. 2017, Article ID 5036514, 4 pages, 2017. https://doi.org/10.1155/2017/5036514

Jiang, Z., Wang, Y., Jiang, Y., Xu, Y. and Meng, B., 2013. Vertebral osteomyelitis and epidural abscess due to Aspergillus nidulans resulting in spinal cord compression: Case report and literature review. Journal of international medical research, 41(2), pp.502-510.

Joly, V., Belmatoug, N., Leperre, A., Robert, J., Jault, F., Carbon, C. and Yeni, P., 1997. Pacemaker endocarditis due to Candida albicans: case report and review. Clinical infectious diseases, 25(6), pp.1359-1362.

Jossi, M., Ambrosioni, J., Macedo-Vinas, M. and Garbino, J., 2010. Invasive fusariosis with prolonged fungemia in a patient with acute lymphoblastic leukemia: case report and review of the literature. International Journal of Infectious Diseases, 14(4), pp.e354-e356.

Jumaa, P.A., Lightowler, C., Baker, L.R. and Das, S.S., 1995. Cutaneous infection caused by Phialophora richardsiae treated successfully by surgical excision in an immunocompromised patient. The Journal of infection, 30(3), pp.261-262.

Jun, J.H., Ryoo, N.H. and Chang, S.D., 2014. A Case of Phoma glomerata Keratitis Occurred in Recurrent Herpes Simplex Keratitis Cicatrix. Journal of the Korean Ophthalmological Society, 55(8), pp.1229-1232.

Jung, J., Moon, Y.S., Yoo, J.A., Lim, J.H., Jeong, J. and Jun, J.B., 2018. Investigation of a nosocomial outbreak of fungemia caused by Candida pelliculosa (Pichia anomala) in a Korean tertiary care center. Journal of Microbiology, Immunology and Infection, 51(6), pp.794-801.

Júnior, M.C., de Moraes Arantes, A., Silva, H.M., Costa, C.R. and Silva, M.D.R.R., 2013. Acremonium kiliense: case report and review of published studies. Mycopathologia, 176, pp.417-421.

Kabtani, J., Militello, M. and Ranque, S., 2022. Coniochaeta massiliensis sp. nov. Isolated from a Clinical Sampl28. Journal of Fungi, 8(10), p.999.

Kamalam, A., Ajithadass, K., Sentamilselvi, G. and Thambiah, A.S., 1992. Paronychia and black discoloration of a thumb nail caused by Curvularia lunata. Mycopathologia, 118, pp.83-84.

Kamei, K., Unno, H., Nagao, K., Kuriyama, T., Nishimura, K. and Miyaji, M., 1994. Allergic bronchopulmonary mycosis caused by the basidiomycetous fungus Schizophyllum commune. Clinical Infectious Diseases, 18(3), pp.305-309.

Kamoshita, M., Matsumoto, Y., Nishimura, K., Katono, Y., Murata, M., Ozawa, Y., Shimmura, S. and Tsubota, K., 2015. Wickerhamomyces anomalus fungal keratitis responds to topical treatment with antifungal micafungin. Journal of Infection and Chemotherapy, 21(2), pp.141-143.

Kaneko, Y., Oinuma, K.I., Terachi, T., Arimura, Y., Niki, M., Yamada, K., Kakeya, H. and Mizutani, T., 2018. Successful treatment of intestinal mycosis caused by a simultaneous infection with Lichtheimia ramosa and Aspergillus calidoustus. Internal Medicine, 57(16), pp.2421-2424.

Kang, D., Ran, Y., Li, C., Dai, Y. and Lama, J., 2013. Impetigo-Like Tinea Faciei Around the Nostrils Caused by Arthroderma vanbreuseghemii Identified Using Polymerase Chain Reaction–Based Sequencing of Crusts. Pediatric Dermatology, 30(6), pp.e136-e137.

Kang, Y., Li, L., Zhu, J., Zhao, Y. and Zhang, Q., 2013. Identification of Fusarium from a patient with fungemia after multiple organ injury. Mycopathologia, 176(1), pp.151-155.

Kang, D., Jiang, X., Wan, H., Ran, Y., Hao, D. and Zhang, C., 2014. Mucor irregularis infection around the inner canthus cured by amphotericin B: a case report and review of published literatures. Mycopathologia, 178, pp.129-133.

Kantarcioğlu, A.S., Celkan, T., Yücel, A., Mikami, Y., Kurugoglu, S., Mitani, H. and Altas, K., 2009. Fatal Trichoderma harzianum infection in a leukemic pediatric patient. Medical Mycology, 47(2), pp.207-215.

Kantarcioglu, A.S., Guarro, J., de Hoog, G.S., Apaydin, H., Kiraz, N., Balkan, I.I. and Ozaras, R., 2016. A case of central nervous system infection due to Cladophialophora bantiana. Revista Iberoamericana de Micología, 33(4), pp.237-241.

Kataoka-Nishimura, S., Akiyama, H., Saku, K., Kashiwa, M., Mori, S., Tanikawa, S., Sakamaki, H. and Onozawa, Y., 1998. Invasive infection due to Trichosporon cutaneum in patients with hematologic malignancies. Cancer: Interdisciplinary International Journal of the American Cancer Society, 82(3), pp.484-487.

Katkar, V.J., Tankhiwale, S.S. and Kurhade, A., 2011. Fusarium soloni mycetoma. Indian Journal of Dermatology, 56(3), p.315.

Kaufmann, C., Arnold, M., Schipf, A., Bruderer, V.L. and Iselin, K.C., 2021. Tintelnotia destructans keratitis: A clinicopathological report and review of the literature. Cornea, 40(3), pp.380-382.

Kaur, R., Bala, K., Ahuja, R.B., Srivastav, P. and Bansal, U., 2014. Primary cutaneous mucormycosis in a patient with burn wounds due to Lichtheimia ramosa. Mycopathologia, 178(3), pp.291-295.

Kaur, H., Shankarnarayana, S.A., Hallur, V., Muralidharan, J., Biswal, M., Ghosh, A.K., Ray, P., Chakrabarti, A. and Rudramurthy, S.M., 2020. Prolonged outbreak of Candida krusei candidemia in paediatric ward of tertiary care hospital. Mycopathologia, 185(2), pp.257-268.

Kaushik, S., Ram, J., Chakrabarty, A., Dogra, M.R., Brar, G.S. and Gupta, A., 2001. Curvularia lunata endophthalmitis with secondary keratitis. American journal of ophthalmology, 131(1), pp.140-142.

Kaushik, R., Chander, J., Gupta, S., Sharma, R. and Punia, R.S., 2012. Fatal primary cutaneous zygomycosis caused by Saksenaea vasiformis: case report and review of literature. Surgical Infections, 13(2), pp.125-129.

Kebbe, J. and Mador, M.J., 2016. Exophiala pisciphila: a novel cause of allergic bronchopulmonary mycosis. Journal of thoracic disease, 8(7), p.E538.

Kennedy, H.F., Simpson, E.M., Wilson, N., Richardson, M.D. and Michie, J.R., 1998. Aspergillus flavus endocarditis in a child with neuroblastoma. Journal of Infection, 36(1), pp.126-127.

Kessler, A.T., Kourtis, A.P. and Simon, N., 2002. Peripheral thromboembolism associated with Malassezia furfur sepsis. The Pediatric infectious disease journal, 21(4), pp.356-357.

Keyser, A., Schmid, F.X., Linde, H.J., Merk, J. and Birnbaum, D.E., 2002. Disseminated Cladophialophora bantiana infection in a heart transplant recipient. The Journal of heart and lung transplantation, 21(4), pp.503-505.

Khalid, S.N., Rizwan, N., Khan, Z.A., Najam, A., Khan, A.M., Almas, T., Khedro, T., Nagarajan, V.R., Alshamlan, A., Gronfula, A. and Alshehri, R., 2021. Fungal burn wound infection caused by Fusarium dimerum: A case series on a rare etiology. Annals of Medicine and Surgery, 70, p.102848.

Khan, Z.U., Ahmad, S., Brazda, A. and Chandy, R., 2009. Mucor circinelloides as a cause of invasive maxillofacial zygomycosis: an emerging dimorphic pathogen with reduced susceptibility to posaconazole. Journal of clinical microbiology, 47(4), pp.1244-1248.

Khan, S., Pillai, G.S., Vivek, V., Dinesh, K. and Karim, P.M., 2012. Post-operative endophthalmitis due to Fusarium dimerum. Southeast Asian J Trop Med Public Health, 43, pp.1484-1488.

Khan, S., Kumar, A., Bhaskaran, V., Chandran, S. and Dinesh, K., 2019. Chronic fungal osteomyelitis of the tibia due to Acremonium curvulum: A rare case. Pan African Medical Journal, 34(1).

Khazim, R.M., Debnath, U.K. and Fares, Y., 2006. Candida albicans osteomyelitis of the spine: progressive clinical and radiological features and surgical management in three cases. European Spine Journal, 15(9), pp.1404-1410.

Kieselová, K., Gomes, T., Santiago, F. and Martinha, H., 2021. Emerging cutaneous phaeohyphomycosis caused by Alternaria infectoria. Acta Médica Portuguesa, 34(11), pp.774-778.

Kim, D.M., Suh, M.K., Ha, G.Y. and Sohng, S.H., 2012. Fingernail onychomycosis due to Aspergillus niger. Annals of dermatology, 24(4), pp.459-463.

Kim, H., Yi, Y., Cho, S.Y., Lee, D.G., Chun, H.S., Park, C., Kim, Y.J. and Park, Y.J., 2022. Pneumonia due to Schizophyllum commune in a patient with acute myeloid leukemia: Case report and literature review. Infection & Chemotherapy, 54(1), p.195.

Kim, D.M., Lee, M.H., Suh, M.K., Ha, G.Y., Kim, H. and Choi, J.S., 2013. Onychomycosis caused by Chaetomium globosum. Annals of dermatology, 25(2), pp.232-236.

Kimura, M., Yaguchi, T., Sutton, D.A., Fothergill, A.W., Thompson, E.H. and Wickes, B.L., 2011. Disseminated human conidiobolomycosis due to Conidiobolus lamprauges. Journal of Clinical Microbiology, 49(2), pp.752-756.

Kimura, U., Yokoyama, K., Hiruma, M., Kano, R., Takamori, K. and Suga, Y., 2015. Tinea faciei caused by Trichophyton mentagrophytes (molecular type Arthroderma benhamiae) mimics impetigo: a case report and literature review of cases in Japan. Medical mycology journal, 56(1), pp.E1-E5.

Kiryu, H., 1985. A case of cutaneous Curvularia infection caused by Curvularia trifolii. In Typical cutaneous fungus disease-rare case, Proceedings of the 37th meeting of Japanese Society of Medical Mycology, Yokohama, 1985 (pp. 63-66).

Kitaura, T., Chikumi, H., Murota, H., Fujiwara, H., Touge, H., Okada, K., Nakamoto, M., Igishi, T., Burioka, N., Yaguchi, T. and Shimizu, E., 2014. A case of lung abscess due to Aspergillus viridinutans in a patient with aplastic anemia. Kansenshogaku zasshi. The Journal of the Japanese Association for Infectious Diseases, 88(6), pp.855-860.

Kleinotiene, G., Posiunas, G., Raistenskis, J., Zurauskas, E., Stankeviciene, S., Daugelaviciene, V. and Machaczka, M., 2013. Liposomal amphotericin B and surgery as successful therapy for pulmonary Lichtheimia corymbifera zygomycosis in a pediatric patient with acute promyelocytic leukemia on antifungal prophylaxis with posaconazole. Medical Oncology, 30(1), pp.1-7.

Kobayashi, H., Sano, A., Aragane, N., Fukuoka, M., Tanaka, M., Kawaura, F., Fukuno, Y., Matsuishi, E. and Hayashi, S., 2008. Disseminated infection by Bipolaris spicifera in an immunocompetent subject. Medical mycology, 46(4), pp.361-365.

Kobayashi, M., Kitahara, H., Yaguchi, T. and Sato, T., 2018. A case of tinea corporis on the arm caused by Nannizzia gypsea with dermatoscopic images. JDDG-Journal der Deutschen Dermatologischen Gesellschaft, 16(6), pp.784-786.

Koç, A.N., Utaş, C., Oymak, O. and Sehmen, E., 1998. Peritonitis due to Acremonium strictum in a patient on continuous ambulatory peritoneal dialysis. Nephron, 79(3), pp.357-358.

Kocsube, S., Toth, M., Vagvolgyi, C., Doczi, I., Pesti, M., Pocsi, I., Szabo, J., Varga, J., 2007. Occurrence and genetic variability of Candida parapsilosis sensu lato in Hungary. J. Med. Microbiol. 56, 190–195.

Kocyigit, I., Unal, A., Sipahioglu, M.H., Tokgoz, B., Oymak, O. and Utas, C., 2010. Peritonitis due to Candida rugosa: the first case report. Peritoneal Dialysis International, 30(5), pp.576-577.

Koç, A.N., Ukşal, Ü. and Oymak, O.K.T.A.Y., 2001. Case report. Successfully treated subcutaneous infection with Sporothrix schenckii in Turkey. Mycoses, 44(7-8), pp.330-333.

Kondo, M., Hiruma, M., Nishioka, Y., Mayuzumi, N., Mochida, K., Ikeda, S. and Ogawa, H., 2005. A case of chromomycosis caused by Fonsecaea pedrosoi and a review of reported cases of dematiaceous fungal infection in Japan. Mycoses, 48(3), pp.221-225.

Konidaris, V., Mersinoglou, A., Vyzantiadis, T.A., Papadopoulou, D., Boboridis, K.G. and Ekonomidis, P., 2013. Corneal transplant infection due to Alternaria alternata: a case report. Case Reports in Ophthalmological Medicine, 2013.

Koo, S., Klompas, M. and Marty, F.M., 2010. Fonsecaea monophora cerebral phaeohyphomycosis: case report of successful surgical excision and voriconazole treatment and review. Medical mycology, 48(5), pp.769-774.

Kordossis, T., Avlami, A., Velegraki, A., Stefanou, I., Georgakopoulos, G., Papalambrou, C. and Legakis, N.J., 1998. First report of Cryptococcus laurentii meningitis and a fatal case of Cryptococcus albidus cryptococcaemia in AIDS patients. Medical mycology, 36(5), pp.335-339.

Kordy, F.N., Al-Mohsen, I.Z., Hashem, F., Almodovar, E., Al Hajjar, S. and Walsh, T.J., 2004. Successful treatment of a child with posttraumatic necrotizing fasciitis caused by Apophysomyces elegans: case report and review of literature. The Pediatric infectious disease journal, 23(9), pp.877-879.

Korres, G., Meletiadis, J., Delides, A., Antoniadou, A., Petrikkos, G., Zerva, L., Yiotakis, I., Siopi, M., Kalogeropoulou, E., Georgantis, I. and Nikolopoulos, T.P., 2015. Fungal malignant otitis externa caused by Alternaria chlamydospora: first case report. JMM Case Reports, 2(1), p.e000021.

Kosa, P., Valach, M., Tomaska, L., Wolfe, K.H. and Nosek, J., 2006. Complete DNA sequences of the mitochondrial genomes of the pathogenic yeasts Candida orthopsilosis and Candida metapsilosis: insight into the evolution of linear DNA genomes from mitochondrial telomere mutants. Nucleic acids research, 34(8), pp.2472-2481.

Krcmery Jr, V., Jesenska, Z., Spanik, S., Gyarfas, J., Nogova, J., Botek, R., Mardiak, J., Sufliarsky, J., Sisolakova, J., Vanickova, M. and Kunova, A., 1997. Fungaemia due to Fusarium spp. in cancer patients. Journal of hospital infection, 36(3), pp.223-228.

Krisher, K.K., Holdridge, N.B., Mustafa, M.M., Rinaldi, M.G. and McGough, D.A., 1995. Disseminated Microascus cirrosus infection in pediatric bone marrow transplant recipient. Journal of clinical microbiology, 33(3), pp.735-737.

Krizsan, K., Toth, E., Nagy, L.G., Galgoczy, L., Manikandan, P., Chandrasekaran, M., Kadaikunnan, S., Alharbi, N.S., Vágvölgyi, C. and Papp, T., 2015. Molecular identification and antifungal susceptibility of Curvularia australiensis, C. hawaiiensis and C. spicifera isolated from human eye infections. Mycoses, 58(10), pp.603-609.

Kucukkaya, I.C., Gulsever, C.I., Dolas, I., Genc, G.E., Kuskucu, M.A., Sabanci, P.A. and Erturan, Z., 2023. First case of Rhinocladiella mackenziei brain abscess in Turkey: Case report and review of the literature. Mycoses, 66(9), pp.755-766.

Kutlu, M., Ergin, Ç., Bir, F., Hilmioğlu-Polat, S., Gümral, R., Necan, C., Koçyiğit, A. and Sayın-Kutlu, S., 2014. Pulmonary mucormycosis due to Lichtheimia ramosa in a patient with HIV infection. Mycopathologia, 178(1), pp.111-115.

Kutleša, M., Mlinarić-Missoni, E., Hatvani, L., Voncina, D., Simon, S., Lepur, D. and Baršić, B., 2012. Chronic fungal meningitis caused by Aureobasidium proteae. *Diagnostic microbiology and infectious disease*, *73*(3), pp.271-272.

Kviliute, R., Paskevicius, A., Gulbinovic, J., Stulpinas, R. and Griskevicius, L., 2008. Nonfatal Trichoderma citrinoviride pneumonia in an acute myeloid leukemia patient. Annals of Hematology, 87(6), p.501.

Kwok, H.K., Chan, J.W., Li, I.W., Chu, S.Y. and Lam, C.W., 2009. Coccidioidomycosis as a rare cause of pneumonia in non-endemic areas: a short exposure history should not be ignored. Respirology, 14(4), pp.617-620.

Kudur, M.H., Prakash, P.Y. and Savitha, M., 2013. Fusarium solani causing quasi-invasive infection of the foot in an immunocompetent middle-aged man from South India. Indian Journal of Dermatology, 58(3), p.241.

Kueter, J.C., MacDiarmid, S.A. and Redman, J.F., 2002. Anuria due to bilateral ureteral obstruction by Aspergillus flavus in an adult male. Urology, 59(4), p.601.

Kulkarni, V.L., Kinikar, A.G., Bhalerao, D.S. and Roushani, S., 2017. A case of keratomycosis caused by Fusarium solani at rural tertiary care center. Journal of clinical and diagnostic research: JCDR, 11(9), p.DD01.

Kusenbach, G., Skopnik, H., Haase, G., Friedrichs, F. and Döhmen, H., 1992. Exophiala dermatitidis pneumonia in cystic fibrosis. European journal of pediatrics, 151, pp.344-346.

Lagrou, K., Massonet, C., Theunissen, K., Meersseman, W., Lontie, M., Verbeken, E., Van Eldere, J. and Maertens, J., 2005. Fatal pulmonary infection in a leukaemic patient caused by Hormographiella aspergillata. Journal of medical microbiology, 54(7), pp.685-688.

Lai, C.C., Lin, W.R., Chen, C.Y., Chang, K., Lu, P.L., Chen, Y.H., Lee, K.M., Chang, T.C. and Lin, C.Y., 2013. Acute meningitis caused by Cladosporium sphaerospermum. *The American Journal of the Medical Sciences*, *346*(6), pp.523-525.

Lamas-Francis, D., Llovo-Taboada, J., Navarro, D., Touriño, R. and Rodríguez-Ares, T., 2022. Necrotizing scleritis due to Hormographiella aspergillata. European Journal of Ophthalmology, p.11206721221118209.

Lampert, R.P., Hutto, J.H., Donnelly, W.H. and Shulman, S.T., 1977. Pulmonary and cerebral mycetoma caused by Curvularia pallescens. Journal of Pediatrics, 91(4), pp.603-605.

Lang, R., Stokes, W., Lemaire, J., Johnson, A. and Conly, J., 2019. A case report of Coccidioides posadasii meningoencephalitis in an immunocompetent host. BMC Infectious Diseases, 19(1), pp.1-5.

Lastoria, C., Cascina, A., Bini, F., Di Matteo, A., Cavanna, C., Farina, C., Carretto, E. and Meloni, F., 2009. Pulmonary Cladophialophora boppii infection in a lung transplant recipient: case report and literature review. The Journal of heart and lung transplantation, 28(6), pp.635-637.

Lacasse, A. and Cleveland, K.O., 2009. Trichosporon mucoides fungemia in a liver transplant recipient: case report and review. Transplant Infectious Disease, 11(2), pp.155-159.

Latha, R., Sasikala, R., Muruganandam, N. and Prakash, M.S., 2010. Onychomycosis due to ascomycete Chaetomium globosum: a case report. Indian Journal of Pathology and Microbiology, 53(3), p.566.

Lazar, S.P., Lukaszewicz, J.M., Persad, K.A. and Reinhardt, J.F., 2014. Rhinocerebral Mucor circinelloides infection in immunocompromised patient following yogurt ingestion. Delaware medical journal, 86(8), pp.245-248.

Lee, J., Yew, W.W., Chiu, C.S.W., Wong, P.C., Wong, C.F. and Wang, E.P., 2002. Delayed sternotomy wound infection due to Paecilomyces variotii in a lung transplant recipient. The Journal of heart and lung transplantation, 21(10), pp.1131-1134.

Lee, W.G., Shin, J.H., Uh, Y., Kang, M.G., Kim, S.H., Park, K.H. and Jang, H.C., 2011. First three reported cases of nosocomial fungemia caused by Candida auris. Journal of clinical microbiology, 49(9), pp.3139-3142.

Lee, J., Cho, Y.G., Kim, D.S., Choi, S.I. and Lee, H.S., 2019. First case of catheter-related Malassezia pachydermatis fungemia in an adult. Annals of laboratory medicine, 39(1), pp.99-101.

Lee, J.M., Han, E., Kim, J., Park, J.H., Sung, G.H., Shin, J.H. and Park, Y.J., 2020. Five Korean cases of respiratory tract infection by filamentous basidiomycetes. Annals of Laboratory Medicine, 40(1), pp.84-87.

Letscher-Bru, V., Campos, F., Waller, J., Randriamahazaka, R., Candolfi, E. and Herbrecht, R., 2002. Successful outcome of treatment of a disseminated infection due to Fusarium dimerum in a leukemia patient. Journal of clinical microbiology, 40(3), pp.1100-1102.

Levenstadt, J.S., Poutanen, S.M., Mohan, S., Zhang, S. and Silverman, M., 2012. Pleurostomophora richardsiae–an insidious fungus presenting in a man 44 years after initial inoculation: a case report and review of the literature. Canadian Journal of Infectious Diseases and Medical Microbiology, 23, pp.110-113.

Levin, T.P., Baty, D.E., Fekete, T., Truant, A.L. and Suh, B., 2004. Cladophialophora bantiana brain abscess in a solid-organ transplant recipient: case report and review of the literature. Journal of clinical microbiology, 42(9), pp.4374-4378.

Li DM, Lun LD, Ge J, Zhang GJ, Li XL and de Hoog GS (2021) Case Report: Rhizopus arrhizus Rhino-Orbital-Cerebral Mycosis and Lethal Midline Granuloma: Another Fungal Etiological Agent. Front. Med. 8:578684. doi: 10.3389/fmed.2021.578684

Li, S., Perlman, J.I., Edward, D.P. and Weiss, R., 1998. Unilateral Blastomyces dermatitidis endophthalmitis and orbital cellulitis: a case report and literature review. Ophthalmology, 105(8), pp.1466-1470.

Li, D.M., Li, R.Y., De Hoog, G.S., Sudhadham, M. and Wang, D.L., 2011. Fatal Exophiala infections in China, with a report of seven cases. Mycoses, 54(4), pp.e136-e142.

Li, D.M., Li, R.Y., De Hoog, G.S., Wang, Y.X. and Wang, D.L., 2009. Exophiala asiatica, a new species from a fatal case in China. Medical Mycology, 47(1), pp.101-109.

Li, D.M., De Hoog, G.S., Saunte, D.L., van den Ende, A.G. and Chen, X.R., 2008. Coniosporium epidermidis sp. nov., a new species from human skin. Studies in Mycology, 61, pp.131-136.

Li, D.M. and Lun, L.D., 2012. Mucor irregularis infection and lethal midline granuloma: a case report and review of published literature. Mycopathologia, 174, pp.429-439.

Li Pomi, F., Di Bartolomeo, L., Vaccaro, M., Lentini, M., Cristadoro, S., Lucanto, M.C., Lombardo, M., Costa, S. and Borgia, F., 2022. Malassezia Folliculitis following Triple Therapy for Cystic Fibrosis. Medicina, 58(9), p.1204.

Liang, K.P., Tleyjeh, I.M., Wilson, W.R., Roberts, G.D. and Temesgen, Z., 2006. Rhino-orbitocerebral mucormycosis caused by Apophysomyces elegans. Journal of clinical microbiology, 44(3), pp.892-898.

Liang, G.Z., Xu, W.Q., Zheng, X.L., Mei, H., Lv, G.X., Shen, Y.N., Li, D.M. and Liu, W.D., 2018. Successful treatment by surgery of a primary cutaneous mucormycosis caused by mucor irregularis. Mycopathologia, 183, pp.445-449.

Lief, M.H., Caplivski, D., Bottone, E.J., Lerner, S., Vidal, C. and Huprikar, S., 2011. Exophiala jeanselmei infection in solid organ transplant recipients: report of two cases and review of the literature. Transplant Infectious Disease, 13(1), pp.73-79.

Lin, M.Y., Carmeli, Y., Zumsteg, J., Flores, E.L., Tolentino, J., Sreeramoju, P. and Weber, S.G., 2005. Prior antimicrobial therapy and risk for hospital-acquired Candida glabrata and Candida krusei fungemia: a case-case-control study. Antimicrobial agents and chemotherapy, 49(11), pp.4555-4560.

Liu, M., Xin, X., Li, J. and Chen, S., 2015. The first case of endophthalmitis due to Rhinocladiella basitona in an immunocompetent patient. Diagnostic Microbiology and Infectious Disease, 83(1), pp.49-52.

Liu, Q., Kong, L., Hua, L. and Xu, S., 2021. Pulmonary Microascus cirrosus infection in an immunocompetent patient with bronchiectasis: A case report. Respiratory Medicine Case Reports, 34, p.101484.

Lu, H., Marengo, M.F., Mihu, C.N., Garcia-Manero, G. and Suarez-Almazor, M.E., 2012. Rare case of septic arthritis caused by Candida krusei: case report and literature review. The Journal of Rheumatology, 39(6), pp.1308-1309.

Ioakimidou, A., Vyzantiadis, T.A., Sakellari, I., Arabatzis, M., Smias, C., Douka, V., Velegraki, A., Anagnostopoulos, A. and Malissiovas, N., 2013. An unusual cluster of Acremonium kiliense fungaemias in a haematopoietic cell transplantation unit. Diagnostic Microbiology and Infectious Disease, 75(3), pp.313-316.

Lodato, F., Tamé, M.R., Montagnani, M., Sambri, V., Liguori, G., Azzaroli, F., Costigliola, P., Grazi, G., Roda, E. and Mazzella, G., 2006. Systemic fungemia and hepatic localizations of Fusarium solani in a liver transplanted patient: an emerging fungal agent. Liver transplantation, 12(11), pp.1711-1714.

Lipový, B., Raška, F., Kocmanová, I., Hanslianová, M., Hladík, M., Holoubek, J., Bezdíček, M. and Macháček, C., 2021. Trichoderma longibrachiatum and Aspergillus fischeri infection as a cause of skin graft failure in a patient with critical burns after liver transplantation. Journal of Fungi, 7(6), p.487.

Liang, G., Shen, Y., Lv, G., Zheng, H., Mei, H., Zheng, X., Kong, X., Blechert, O., Li, D. and Liu, W., 2018. Coccidioidomycosis: Imported and possible domestic cases in China: A case report and review, 1958-2017. Mycoses, 61(7), pp.506-513.

Lin, H.C., Lin, H.Y., Su, B.H., Ho, M.W., Ho, C.M., Lee, C.Y., Lin, M.H., Hsieh, H.Y., Lin, H.C., Li, T.C. and Hwang, K.P., 2013. Reporting an outbreak of Candida pelliculosa fungemia in a neonatal intensive care unit. Journal of Microbiology, Immunology and Infection, 46(6), pp.456-462.

Linsangan, L.C. and Ross, L.A., 1999. Coccidioides immitis infection of the neonate: two routes of infection. The Pediatric infectious disease journal, 18(2), pp.171-173.

Liu, P.Y.F., 1998. Cryptococcal osteomyelitis: case report and review. Diagnostic microbiology and infectious disease, 30(1), pp.33-35.

Liu, K., Howell, D.N., Perfect, J.R. and Schell, W.A., 1998. Morphologic criteria for the preliminary identification of Fusarium, Paecilomyces, and Acremonium species by histopathology. American Journal of Clinical Pathology, 109(1), pp.45-54.

Liu, H., Lei, X., Chen, L., Hu, S., Li, G. and Deng, Z., 2021. Keratomycosis caused by a rare pathogen, myrothecium verrucaria. Mycopathologia, 186(6), pp.893-895.

Liu, F., Liu, Y., Yuan, N., Zhang, X., Cao, M., Dong, J. and Zhang, J., 2021. Fixed cutaneous sporotrichosis due to Sporothrix globosa. Clinical, Cosmetic and Investigational Dermatology, pp.91-96.

Lizano-Calvo, M., Brenes-Angulo, A. and Gómez-Alpízar, L., 2013. First case of onychomycosis caused by Cylindrocarpon lichenicola in an immunosuppressed patient in Costa Rica. Acta Médica Costarricense, 55(4), pp.199-204.

Lo Cascio, G., Ligozzi, M., Maccacaro, L. and Fontana, R., 2004. Utility of molecular identification in opportunistic mycotic infections: a case of cutaneous Alternaria infectoria infection in a cardiac transplant recipient. Journal of clinical microbiology, 42(11), pp.5334-5336.

Lonial, S., Williams, L., Carrum, G. and Ostrowski, M., 1997. Neosartorya fischeri: an invasive fungal pathogen in an allogeneic bone marrow transplant patient. Bone marrow transplantation, 19(7), pp.753-755.

Lopes, J.O., Alves, S.H., Benevenga, J.P., Brauner, F.B., Castro, M.S. and Melchiors, E., 1994. Curvularia lunata peritonitis complicating peritoneal dialysis. Mycopathologia, 127(2), pp.65-67.

Lopes, J.O., Alves, S.H., Rosa, A.C., Silva, C.B., Sarturi, J.C. and Souza, C.A.R., 1995. Acremonium kiliense peritonitis complicating continuous ambulatory peritoneal dialysis: report of two cases. Mycopathologia, 131(2), pp.83-85.

Lopes, L., Borges-Costa, J., Soares-Almeida, L., Filipe, P., Neves, F., Santana, A., Guerra, J. and Kutzner, H., 2013, October. Cutaneous alternariosis caused by Alternaria infectoria: three cases in kidney transplant patients. In Healthcare (Vol. 1, No. 1, pp. 100-106). MDPI.

Lu, H., Marengo, M.F., Mihu, C.N., Garcia-Manero, G. and Suarez-Almazor, M.E., 2012. Rare case of septic arthritis caused by Candida krusei: case report and literature review. The Journal of Rheumatology, 39(6), pp.1308-1309.

Lukić-Grlić, A., Mlinarić-Missoni, E., Škarić, I., Važić-Babić, V. and Svetec, I.K., 2011. Candida utilis candidaemia in neonatal patients. Journal of medical microbiology, 60(6), pp.838-841.

Ma, B., Seymour, J.F., Januszewicz, H. and Slavin, M.A., 2001. Cure of pulmonary Rhizomucor pusillus infection in a patient with hairy-cell leukemia: role of liposomal amphotericin B and GM-CSF. Leukemia & lymphoma, 42(6), pp.1393-1399.

Magobo, R.E., Corcoran, C., Seetharam, S. and Govender, N.P., 2014. Candida auris–associated candidemia, South Africa. Emerging infectious diseases, 20(7), p.1250.

MacDonald, H.J., Fong, I.W., Gardiner, G.W. and Soutter, D.I., 1992. Splenic abscess caused by Blastomyces dermatitidis in association with peritoneal involvement: case report and review. Clinical infectious diseases, 14(1), pp.348-349.

Madigan, T., Fatemi, Y., Theel, E.S., Moodley, A. and Boyce, T.G., 2017. Central nervous system blastomycosis in children: a case report and review of the literature. The Pediatric Infectious Disease Journal, 36(7), pp.679-684.

Madariaga, M.G., Tenorio, A. and Proia, L., 2003. Trichosporon inkin peritonitis treated with caspofungin. Journal of clinical microbiology, 41(12), pp.5827-5829.

Maduri A, Patnayak R, Verma A, Mudgeti N, Kalawat U, Asha T. Subcutaneous infection by Cladosporium sphaerospermum-A rare case report. Indian J Pathol Microbiol [serial online] 2015 [cited 2023 Nov 30];58:406-7. Available from: https://www.ijpmonline.org/text.asp?2015/58/3/406/162936

Mai, H., Champion, L., Ouali, N., Hertig, A., Peraldi, M.N., Glotz, D., Rondeau, E., Costa, M.A., Snanoudj, R., Benoit, G. and Charpentier, B., 2006. Candida albicans arteritis transmitted by conservative liquid after renal transplantation: a report of four cases and review of the literature. Transplantation, 82(9), pp.1163-1167.

Malik, S., Bajpai, V., Betai, S., Pal, L. and Marak, R.S., 2020. An unusual case of Microascus brain abscess in an immunocompetent child and a review of the literature. Journal of Family Medicine and Primary Care, 9(2), p.1244.

Malik, R., Capoor, M.R., Vanidassane, I., Gogna, A., Singh, A., Sen, B., Rudramurthy, S.M., Honnavar, P., Gupta, S. and Chakrabarti, A., 2016. Disseminated Emmonsia pasteuriana infection in India: a case report and a review. Mycoses, 59(2), pp.127-132.

Malin, A.S., Gwanzura, L.K., Robertson, V.J., Musvaire, P., Mason, P.R. and Klein, S., 1995. Pneumocystis carinii pneumonia in Zimbabwe. *The Lancet*, *346*(8985), pp.1258-1261.

Mangaraj, S., Sethy, G., Patro, M.K. and Padhi, S., 2014. A rare case of subcutaneous mucormycosis due to Syncephalastrum racemosum: Case report and review of literature. Indian J Med Microbiol, 32(4), p.448.

Mangiaterra, M., Giusiano, G., Smilasky, G., Zamar, L., Amado, G. and Vicentán, C., 2001. Keratomycosis caused by Cylindrocarpon lichenicola. Sabouraudia, 39(1), pp.143-145.

Mansour, A. and Jordan, K., 2014. Disseminated Cladophialophora bantiana disease in a patient with prediabetes. Case Reports, 2014, p.bcr2014206426.

Mao, Y., Li, X., Lou, H., Shang, X., Mai, Y., Yang, L., Peng, F. and Fu, X., 2021. Detection of Coccidioides posadasii in a patient with meningitis using metagenomic next-generation sequencing: a case report. BMC Infectious Diseases, 21(1), pp.1-5.

Maphanga, T.G., Birkhead, M., Muñoz, J.F., Allam, M., Zulu, T.G., Cuomo, C.A., Schwartz, I.S., Ismail, A., Naicker, S.D., Mpembe, R.S. and Corcoran, C., 2020. Human blastomycosis in South Africa caused by Blastomyces percursus and Blastomyces emzantsi sp. nov., 1967 to 2014. Journal of clinical microbiology, 58(3), pp.10-1128.

Marcio, N., Tiyomi, A., Gloria, B., Fernanda, S., Revankar, S.G., Brian, L.W., Sutton, D.A. and Patterson, T.F., 2002. Nosocomial outbreak of Exophiala jeanselmei fungemia associated with contamination of hospital water. Clinical infectious diseases, 34(11), pp.1475-1480.

Marques-da-Silva, S.H., Rodrigues, A.M., de Hoog, G.S., Silveira-Gomes, F. and de Camargo, Z.P., 2012. Case report: occurrence of Paracoccidioides lutzii in the Amazon region: description of two cases. The American journal of tropical medicine and hygiene, 87(4), p.710.

Marques, A.R., Kwon-Chung, K.J., Holland, S.M., Turner, M.L. and Gallin, J.I., 1995. Suppurative cutaneous granulomata caused by Microascus cinereus in a patient with chronic granulomatous disease. Clinical infectious diseases, 20(1), pp.110-114.

Martins Castro, L.G., da Silva Lacaz, C., Guarro, J., Gené, J., Heins-Vaccari, E.M., de Freitas Leite, R.S., Hernández Arriagada, G.L., Ozaki Reguera, M.M., Ito, E.M., Valente, N.Y.S. and Spina Nunes, R., 2001. Phaeohyphomycotic cyst caused by Colletotrichum crassipes. Journal of Clinical Microbiology, 39(6), pp.2321-2324.

Maslen, M.M., 2000. Human cases of cattle ringworm due to Trichophyton verrucosum in Victoria, Australia. Australasian journal of dermatology, 41(2), pp.90-94.

Mathur, P., Hasan, F., Singh, P.K., Malhotra, R., Walia, K. and Chowdhary, A., 2018. Five-year profile of candidaemia at an Indian trauma centre: high rates of Candida auris blood stream infections. Mycoses, 61(9), pp.674-680.

Matsuzaki, T., Kasai, H., Ikeda, H., Tajiri, Y., Suzuki, K., Watanabe, A., Kamei, K. and Urushibara, T., 2021. Voriconazole treatment of pulmonary mycosis caused by Chrysosporium zonatum after treatment for pulmonary tuberculosis. Respirology Case Reports, 9(6), p.e00763.

Marcus, L., Vismer, H.F., Van der Hoven, H.J., Gove, E. and Meewes, P., 1992. Mycotic keratitis caused by Curvularia brachyspora (Boedjin). Mycopathologia, 119(1), pp.29-33.

Markey, R.J., Staat, M.A., Gerrety, M.J.T. and Lucky, A.W., 2003. Tinea capitis due to Trichophyton soudanense in Cincinnati, Ohio, in internationally adopted children from Liberia. Pediatric dermatology, 20(5), pp.408-410.

Marques, D.P., Carvalho, J., Rocha, S. and Domingos, R., 2019. A case of pulmonary mycetoma caused by Paecilomyces variotii. European Journal of Case Reports in Internal Medicine, 6(2).

Marques de Macedo, P., Sztajnbok, D.C.N., Camargo, Z.P., Rodrigues, A.M., Lopes-Bezerra, L.M., Bernardes-Engemann, A.R. and Orofino-Costa, R., 2015. Dacryocystitis due to Sporothrix brasiliensis: a case report of a successful clinical and serological outcome with low-dose potassium iodide treatment and oculoplastic surgery. British Journal of Dermatology, 172(4), pp.1116-1119.

Martínez-Herrera, E., Frías-De-León, M.G., Julián-Castrejón, A., Cruz-Benítez, L., Xicohtencatl-Cortes, J. and Hernández-Castro, R., 2020. Rhino-orbital mucormycosis due to Apophysomyces ossiformis in a patient with diabetes mellitus: a case report. BMC Infectious Diseases, 20(1), pp.1-4.

Marty, F.M., Barouch, D.H., Coakley, E.P. and Baden, L.R., 2003. Disseminated trichosporonosis caused by Trichosporon loubieri. Journal of clinical microbiology, 41(11), pp.5317-5320.

Maruyama, R., Katoh, T. and Nishioka, K., 1999. A Case of Unna-Thost Disease Accompained by Epidermophyton floccosum Infection. The Journal of Dermatology, 26(1), pp.63-66.

Mayser, P., Nilles, M. and De Hoog, G.S., 2002. Case report. Cutaneous phaeohyphomycosis due to Alternaria alternata. Mycoses, 45(7-8), pp.338-340.

Medrano, D. J., R. S. Brilhante, A. Cordeiro Rde, M. F. Rocha, S. H. Rabenhorst, and J. J. Sidrim.2006. Candidemia in a Brazilian hospital: the importance of Candida parapsilosis. Rev. Inst. Med. Trop. Sao Paulo48:17-20.

Mei, J.I., Xue-hua, S.H.E.N., Jing-wen, T.A.N., Zhi-qin, G.A.O. and Lian-juan, Y.A.N.G., 2020. The first case of Tinea Nigra on wrist caused by Hortaea werneckii in Chinese mainland and literature review. Chinese Journal of Mycology, 15(4), p.193.

Meis, J.F., Kullberg, B.J., Pruszczynski, M. and Veth, R.P., 1994. Severe osteomyelitis due to the zygomycete Apophysomyces elegans. Journal of Clinical Microbiology, 32(12), pp.3078-3081.

Mehta, V., Mohanty, A., Meena, S., Rahul, J.S., Uttam Kumar, N., Chattopadhyay, D., Bakliwal, A., Choudhary, R. and Gupta, P., 2020. Wickerhamomyces anomalous: a rare cause of fungemia causing febrile neutropenia in acute lymphoblastic leukemia. Case reports in infectious diseases, 2020.

Menon, A.A., Berg, D.D., Brea, E.J., Deutsch, A.J., Kidia, K.K., Thurber, E.G., Polsky, S.B., Yeh, T., Duskin, J.A., Holliday, A.M. and Gay, E.B., 2020. A case of COVID-19 and Pneumocystis jirovecii coinfection. American journal of respiratory and critical care medicine, 202(1), pp.136-138.

Mendiratta, V., Karmakar, S., Jain, A. and Jabeen, M., 2012. Severe cutaneous zygomycosis due to Basidiobolus ranarum in a young infant. Pediatric Dermatology, 29(1), pp.121-123.

Mendoza, M.A., Anderson, A., Morris, M.I., Lekakis, L., Simkins, J., Prado, C.E., Martinez, O.V., Komanduri, K.V. and Camargo, J.F., 2020. Successful treatment of invasive fungal infection due to highly resistant Aspergillus calidoustus in an allogeneic hematopoietic cell transplant recipient. Mycopathologia, 185(2), pp.399-403.

Mercier, V., Desnos-Ollivier, M., Lamy, A., Mahul, M. and Sasso, M., 2021. Kazachstania slooffiae: an unexpected journey to a human pleural sample. Journal of Medical Mycology, 31(2), p.101109.

Milan, E.P., Silva-Rocha, W.P., de Almeida, J.J.S., Fernandes, T.U.G., de Araújo Prudente, A.L., de Azevedo, M.F., Francisco, E.C., de Azevedo Melo, A.S., Colombo, A.L. and Chaves, G.M., 2018. Trichosporon inkin meningitis in Northeast Brazil: first case report and review of the literature. BMC infectious diseases, 18(1), pp.1-8.

Miller, D.J. and Mejicano, G.C., 2001. Vertebral osteomyelitis due to Candida species: case report and literature review. Clinical infectious diseases, 33(4), pp.523-530.

Minces, L.R., Ho, K.S., Veldkamp, P.J. and Clancy, C.J., 2009. Candida rugosa: a distinctive emerging cause of candidaemia. A case report and review of the literature. Scandinavian journal of infectious diseases, 41(11-12), pp.892-897.

Minta, D.K., Dembélé, M., Lorre, G., Diallo, D.A., Traoré, H.A. and Chabasse, D., 2005. African histoplasmosis (Histoplasma capsulatum var. duboisii): a case report from Mali. Cahiers d'études et de recherches francophones/Santé, 15(3), pp.195-199.

Miossec, C., Morio, F., Lepoivre, T., Le Pape, P., Garcia-Hermoso, D., Gay-Andrieu, F., Haloun, A., Treilhaud, M., Leclair, F. and Miegeville, M., 2011. Fatal invasive infection with fungemia due to Microascus cirrosus after heart and lung transplantation in a patient with cystic fibrosis. Journal of clinical microbiology, 49(7), pp.2743-2747.

Mise, N., Ono, Y., Kurita, N., Sai, K., Nishi, T., Tagawa, H. and Sugimoto, T., 2008. Aureobasidium pullulans peritonitis: case report and review of the literature. *Peritoneal Dialysis International*, *28*(6), pp.679-681.

Mitra, A., Savant, V., Aralikatti, A., Dean, S. and Shah, S., 2009. The use of voriconazole in the treatment of cylindrocarpon keratomycosis. Cornea, 28(2), pp.217-218.

Miyakis, S., Velegraki, A., Delikou, S., Parcharidou, A., Papadakis, V., Kitra, V., Papadatos, I. and Polychronopoulou, S., 2006. Invasive Acremonium strictum infection in a bone marrow transplant recipient. The Pediatric infectious disease journal, 25(3), pp.273-275.

Mlinarić-Missoni, E., Hatvani, L., Kocsube, S., Vágvölgyi, C., Škarić, I. and Lukić-Grlić, A., 2015. Cyberlindnera fabianii in the neonatal and paediatric intensive care unit. JMM Case Reports, 2(3), p.e000032.

Mohsin, J., Hagen, F., Al-Balushi, Z.A., de Hoog, G.S., Chowdhary, A., Meis, J.F. and Al-Hatmi, A.M., 2017. The first cases of Candida auris candidaemia in Oman. Mycoses, 60(9), pp.569-575.

Moin, S., Farooqi, J., Rattani, S., Nasir, N., Zaka, S. and Jabeen, K., 2021. C. auris and non-C. auris candidemia in hospitalized adult and pediatric COVID-19 patients; single center data from Pakistan. Medical Mycology, 59(12), pp.1238-1242.

Moniot, M., Lavergne, R.A., Morel, T., Guieze, R., Morio, F., Poirier, P. and Nourrisson, C., 2020. Hormographiella aspergillata: an emerging basidiomycete in the clinical setting? A case report and literature review. BMC Infectious Diseases, 20, pp.1-7.

Montejo, M., Muniz, M.L., Zarraga, S., Aguirrebengoa, K., Amenabar, J.J., López-Soria, L. and Gonzalez, R., 2002. Infection due to Scedosporium apiospermum in renal transplant recipients: a report of two cases and literature review of central nervous system and cutaneous infections by Pseudallescheria boydii/Sc. apiospermum. Mycoses, 45(9-10), pp.418-427.

Mohammadi, R., Mohammadi, A., Ashtari, F., Khorvash, F., Hakamifard, A., Vaezi, A., Javidnia, J., Meis, J.F. and Badali, H., 2018. Cerebral phaeohyphomycosis due to Rhinocladiella mackenziei in Persian Gulf region: a case and review. Mycoses, 61(4), pp.261-265.

Motswaledi, H.M., Monyemangene, F.M., Maloba, B.R. and Nemutavhanani, D.L., 2012. Blastomycosis: a case report and review of the literature. International Journal of Dermatology, 51(9), pp.1090-1093.

Mouronte-Roibás, C., Leiro-Fernández, V., Botana-Rial, M., Ramos-Hernández, C., Lago-Preciado, G., Fiaño-Valverde, C. and Fernández-Villar, A., 2016. Lichtheimia ramosa: a fatal case of mucormycosis. Canadian respiratory journal, 2016.

Mhmoud NA, Santona A, Fiamma M, Siddig EE, Deligios M, Bakhiet SM, et al. (2019) *Chaetomium atrobrunneum* causing human eumycetoma: The first report. PLoS Negl Trop Dis 13(5): e0007276. https://doi.org/10.1371/journal.pntd.0007276

Mukherjee, B. and Kundu, D., 2018. Necrotizing fungal infection due to Saksenaea erythrospora: A case report and review of literature. Indian Journal of Ophthalmology, 66(10), p.1513.

Mulè, A., Rossini, F., Sollima, A., Lenzi, A., Fumarola, B., Amadasi, S., Chiari, E., Lorenzotti, S., Saccani, B., Van Hauwermeiren, E. and Lanza, P., 2023. Trichosporon asahii infective endocarditis of prosthetic valve: A case report and literature review. Antibiotics, 12(7), p.1181.

Murray, C.K., Beckius, M.L. and McAllister, C.K., 2003. Fusarium proliferatum superficial suppurative thrombophlebitis. Military medicine, 168(5), pp.426-427.

Myoken, Y., Sugata, T., Fujita, Y., Asaoku, H., Fujihara, M. and Mikami, Y., 2002. Fatal necrotizing stomatitis due to Trichoderma longibrachiatum in a neutropenic patient with malignant lymphoma: a case report. International journal of oral and maxillofacial surgery, 31(6), pp.688-691.

Myoken, Y., Sugata, T., Fujita, Y., Kyo, T.I., Fujihara, M., Kohara, T., Katsu, M. and Mikami, Y., 2003. Molecular epidemiology of invasive stomatitis due to Aspergillus flavus in patients with acute leukemia. Journal of oral pathology & medicine, 32(4), pp.215-218.

Naidu, J., Singh, S.M. and Pouranik, M., 1991. Onychomycosis caused by Scopulariopsis brumptii. Mycopathologia, 113(3), pp.159-164.

Najafzadeh, M.J., Sun, J., Vicente, V., Xi, L., van Den Ende, A.G. and De Hoog, G.S., 2010. Fonsecaea nubica sp. nov, a new agent of human chromoblastomycosis revealed using molecular data. Sabouraudia, 48(6), pp.800-806.

Nakagawa, T., Nakashima, K., Takaiwa, T. and Negayama, K., 2000. Trichosporon cutaneum (Trichosporon asahii) infection mimicking hand eczema in a patient with leukemia. Journal of the American Academy of Dermatology, 42(5), pp.929-931.

Nakai, K., Kanda, Y., Mineishi, S., Hori, A., Chizuka, A., Niiya, H., Tanimoto, T., Ohnishi, M., Kami, M., Makimoto, A. and Tanosaki, R., 2002. Primary cutaneous aspergillosis caused by Aspergillus ustus following reduced-intensity stem cell transplantation. Annals of hematology, 81(10), pp.593-596.

Nakamura, T. and Takahashi, H., 2006. Epidemiological study of Candida infections in blood: susceptibilities of Candida spp. to antifungal agents, and clinical features associated with the candidemia. Journal of infection and chemotherapy, 12(3), pp.132-138.

Nakamura, Y., Kano, R., Nakamura, E., Saito, K., Watanabe, S. and Hasegawa, A., 2002. Case report. First report on human ringworm caused by Arthroderma benhamiae in Japan transmitted from a rabbit. Mycoses, 45(3-4), pp.129-131.

Narula, H., Meena, S., Jha, S., Kaistha, N., Pathania, M. and Gupta, P., 2020. Curvularia lunata causing orbital cellulitis in a diabetic patient: An old fungus in a new territory. Current Medical Mycology, 6(1), p.51.

Nayak DR, Balakrishnan R, Nainani S, Siddique S. Paecilomyces fungus infection of the paranasal sinuses. Int J Pediatr Otorhinolaryngol 2000; 52: 183–187.

Nascimento, E., Bonifácio da Silva, M.E.N., Martinez, R. and von Zeska Kress, M.R., 2014. Primary cutaneous cryptococcosis in an immunocompetent patient due to Cryptococcus gattii molecular type VGI in Brazil: a case report and review of literature. Mycoses, 57(7), pp.442-447.

Naseri, A., Fata, A. and Khosravi, A.R., 2012. The first case of Microsporum persicolor infection in Iran.

Navaratnam, J., Bærland TP, Solberg EL, Hermansen NO, Andersen CT, Eide N. 2022. A Case Report of Phialemonium Curvatum Endophthalmitis. *J Ophthalmol Adv Res*, *3*(1), pp.1-6.

Nazir, Z., Hasan, R., Pervaiz, S., Alam, M. and Moazam, F., 1997. Invasive retroperitoneal infection due to Basidiobolus ranarum with response to potassium iodide—case report and review of the literature. Annals of tropical paediatrics, 17(2), pp.161-164.

Neblett Fanfair, R., Benedict, K., Bos, J., Bennett, S.D., Lo, Y.C., Adebanjo, T., Etienne, K., Deak, E., Derado, G., Shieh, W.J. and Drew, C., 2012. Necrotizing cutaneous mucormycosis after a tornado in Joplin, Missouri, in 2011. New England journal of medicine, 367(23), pp.2214-2225.

Neelaveni, V., Tupaki-Sreepurna, A., Thanneru, V. and Kindo, A.J., 2017. Lichtheimia ramosa isolated from a young patient from an infected wound after a road traffic accident. Journal of The Academy of Clinical Microbiologists, 19(1), p.59.

Negroni, R., Helou, S.H., Petri, N., Robles, A.M., Arechavala, A. and Bianchi, M.H., 2004. Case study: posaconazole treatment of disseminated phaeohyphomycosis due to Exophiala spinifera. Clinical Infectious Diseases, 38(3), pp.e15-e20.

Neji, S., Makni, F., Sellami, H., Cheikhrouhou, F., Sellami, A. and Ayadi, A., 2009. First case of Microsporum ferrugineum from Tunisia. Mycopathologia, 167(6), pp.351-353.

Nenoff, P., Verma, S.B., Vasani, R., Burmester, A., Hipler, U.C., Wittig, F., Krüger, C., Nenoff, K., Wiegand, C., Saraswat, A. and Madhu, R., 2019. The current Indian epidemic of superficial dermatophytosis due to Trichophyton mentagrophytes—A molecular study. Mycoses, 62(4), pp.336-356.

Ng, T.T., Campbell, C.K., Rothera, M., Houghton, J.B., Hughes, D. and Denning, D.W., 1994. Successful treatment of sinusitis caused by Cunninghamella bertholletiae. Clinical infectious diseases, 19(2), pp.313-316.

Nguyen, V.Q. and Penn, R.L., 1987. Candida krusei infectious arthritis: a rare complication of neutropenia. The American journal of medicine, 83(5), pp.963-965.

Nguyen, D.K., Davis, C.M., Chinen, J., Vallejo, J.G. and Noroski, L.M., 2009. Basidiomycetous Inonotus (Phellinus) tropicalis osteomyelitis in pediatric and adult X-linked chronic granulomatous disease. Journal of Allergy and Clinical Immunology, 123(2), p.S13.

Nir-Paz, R., Elinav, H., Pierard, G.E., Walker, D., Maly, A., Shapiro, M., Barton, R.C. and Polacheck, I., 2003. Deep infection by Trichophyton rubrum in an immunocompromised patient. Journal of clinical microbiology, 41(11), pp.5298-5301.

Noguchi, H., Sakae, H., Hattori, M. and Hiruma, M., 2010. Review of Two Japanese Cases with Tinea Faciei Identified by Molecular Biological Techniques as Arthroderma vanbreuseghemii. Japanese Journal of Medical Mycology, 51(4).

Nogueira, S.A., Guedes, A.L., Wanke, B., Capella, S., Rodrigues, K., Abreu, T.F., Morais, J.C. and Lambert, J.S., 2001. Osteomyelitis caused by Paracoccidioides brasiliensis in a child from the metropolitan area of Rio de Janeiro. Journal of tropical pediatrics, 47(5), pp.311-315.

Nulens, E., De Laere, E., Vandevelde, H., Hilbrands, L.B., Rijs, A.J., Melchers, W.J. and Verweij, P.E., 2006. Alternaria infectoria phaeohyphomycosis in a renal transplant patient. Sabouraudia, 44(4), pp.379-382.

Ogawa, H., Fujimura, M., Amaike, S., Matsumoto, Y., Kitagawa, M. and Matsuda, T., 1997. Eosinophilic pneumonia caused by Alternaria alternata. Allergy, 52(10), pp.1005-1008.

Ogawa, H., Fujimura, M., Takeuchi, Y., Makimura, K. and Satoh, K., 2012. The definitive diagnostic process and successful treatment for ABPM caused by Schizophyllum commune: a report of two cases. Allergology International, 61(1), pp.163-169.

O'Gorman, S.M., Britton, D. and Collins, P., 2015. An uncommon dermatophyte infection: two cases of cutaneous infection with Trichophyton verrucosum. Clinical and Experimental Dermatology, 40(4), pp.395-398.

Oh, T.H., Shin, S.U., Kim, S.S., Kim, S.E., Kim, U.J., Kang, S.J., Jang, H.C., Jung, S.I., Shin, J.H. and Park, K.H., 2020. Prosthetic valve endocarditis by Trichosporon mucoides: A case report and review of literature. Medicine, 99(41).

Ohira, S., Isoda, K., Hamanaka, H., Takahashi, K., Nishimoto, K. and Mizutani, H., 2002. Case report. Phaeohyphomycosis caused by Phialophora verrucosa developed in a patient with non-HIV acquired immunodeficiency syndrome. Mycoses, 45(1-2), pp.50-54.

Okhuysen, P.C., Rex, J.H., Kapusta, M. and Fife, C., 1994. Successful treatment of extensive posttraumatic soft-tissue and renal infections due to Apophysomyces elegans. Clinical infectious diseases, 19(2), pp.329-331.

Olenski, M., Halliday, C., Gullifer, J., Martinez, E., Crowe, A., Sheorey, H. and Darby, J., 2021. A Case of Trauma-Induced Falciformispora lignatilis Eumycetoma in a Renal Transplant Recipient. Tropical Medicine and Infectious Disease, 6(3), p.144.

Oliveri, S., Cammarata, E., Augello, G. *et al.* *Rhizopus arrhizus* in Italy as the causative agent of primary cerebral zygomycosis in a drug addict. *Eur J Epidemiol* **4**, 284–288 (1988). https://doi.org/10.1007/BF00148911

Olorunnipa, O., Zhang, A.Y. and Curtin, C.M., 2010. Invasive aspergillosis of the hand caused by Aspergillus ustus: a case report. Hand, 5(1), pp.102-105.

O'Quinn, R.P., Hoffmann, J.L. and Boyd, A.S., 2001. Colletotrichum species as emerging opportunistic fungal pathogens: a report of 3 cases of phaeohyphomycosis and review. Journal of the American Academy of Dermatology, 45(1), pp.56-61.

Oremosu, J., Ung, L., Chodosh, J., Cañete-Gibas, C., Wiederhold, N.P., Davies, E.C. and Bispo, P.J., 2023. Fungal keratitis caused by Coniochaeta mutabilis—A case report. *Journal of Medical Mycology*, *33*(2), p.101384.

Orth, B., Frei, R., Itin, P.H., Rinaldi, M.G., Speck, B., Gratwohl, A. and Widmer, A.F., 1996. Outbreak of invasive mycoses caused by Paecilomyces lilacinus from a contaminated skin lotion. Annals of internal medicine, 125(10), pp.799-806.

Otag, F., Yarpuzlu, M., Gulbudak, H., ARSLANKÖYLÜ, A., Fouad, A. and Emekdaş, G., 2015. Candida Pelliculosa Fungemia cases in pediatric intensive care unit. Journal of Pediatric Infection, 9(2).

Ozer, B., Kalaci, A., Duran, N., Dogramaci, Y. and Yanat, A.N., 2009. Cutaneous infection caused by Aspergillus terreus. Journal of medical microbiology, 58(7), pp.968-970.

Paccoud, O., Vignier, N., Boui, M., Migaud, M., Vironneau, P., Kania, R., Méchaï, F., Brun, S., Alanio, A., Tauziède-Espariat, A. and Adle-Biassette, H., 2022. Invasive Rhinosinusitis Caused by Alternaria infectoria in a Patient with Autosomal Recessive CARD9 Deficiency and a Review of the Literature. Journal of Fungi, 8(5), p.446.

Padhi, S., Dash, M., Pattanaik, S. and Sahu, S., 2014. Fungemia due to Trichosporon mucoides in a diabetes mellitus patient: a rare case report. Indian J Med Microbiol, 32(1), pp.72-4.

Padhye, A.A., Detweiler, J.G., Frumkin, A., Bulmer, G.S., Ajello, L. and McGinnis, M.R., 1989. Tinea capitis caused by Microsporum praecox in a patient with sickle cell anaemia. Journal of Medical and Veterinary Mycology, 27(5), pp.313-317.

Padhye, A.A., Davis, M.S., Baer, D., Reddick, A., Sinha, K.K. and Ott, J., 1998. Phaeohyphomycosis caused by Phaeoacremonium inflatipes. *Journal of Clinical Microbiology*, *36*(9), pp.2763-2765.

Padhye, A.A., Verghese, S., Ravichandran, P., Balamurugan, G., Hall, L., Padmaja, P. and Fernandez, M.C., 2003. Trichosporon loubieri infection in a patient with adult polycystic kidney disease. Journal of clinical microbiology, 41(1), pp.479-482.

Padmaja, I.J., Ramani, T.V. and Kalyani, S., 2006. Case Report-Cutaneous zygomycosis-Necrotising fascitis due to Saksenaea vasiformis. Indian Journal of Medical Microbiology, 24(1), pp.58-60.

Pakshir, K. and Hashemi, J., 2006. Dermatophytosis in Karaj, Iran. Indian journal of dermatology, 51(4), p.262.

Palmore, T.N., Shea, Y.R., Childs, R.W., Sherry, R.M. and Walsh, T.J., 2010. Fusarium proliferatum soft tissue infection at the site of a puncture by a plant: recovery, isolation, and direct molecular identification. Journal of clinical microbiology, 48(1), pp.338-342.

Pariseau, B., Lucarelli, M.J. and Appen, R.E., 2007. Unilateral blastomyces dermatitidis optic neuropathy: Case report and systematic literature review. Ophthalmology, 114(11), pp.2090-2094.

Parra-Giraldo, C.M., Valderrama, S.L., Cortes-Fraile, G., Garzón, J.R., Ariza, B.E., Morio, F., Linares-Linares, M.Y., Ceballos-Garzón, A., de la Hoz, A., Hernandez, C. and Alvarez-Moreno, C., 2018. First report of sporadic cases of Candida auris in Colombia. International Journal of Infectious Diseases, 69, pp.63-67.

Paccoud, O., Guery, R., Poirée, S., Jouvion, G., Bougnoux, M.E., Catherinot, E., Hermine, O., Lortholary, O. and Lanternier, F., 2019. Aspergillus felis in patient with chronic granulomatous disease. Emerging Infectious Diseases, 25(12), p.2319.

Padhye, A.A., Helwig, W.B., Warren, N.G., Ajello, L., Chandler, F.W. and McGinnis, M.R., 1988. Subcutaneous phaeohyphomycosis caused by Xylohypha emmonsii. Journal of clinical microbiology, 26(4), pp.709-712.

Pan, J., Tsui, C., Li, M., Xiao, K., de Hoog, G.S., Verweij, P.E., Cao, Y., Lu, H. and Jiang, Y., 2020. First case of rhinocerebral mucormycosis caused by Lichtheimia ornata, with a review of Lichtheimia infections. Mycopathologia, 185(3), pp.555-567.

Paniz-Mondolfi, A.E., Agemy, S., Cañete-Gibas, C., Gitman, M.R., Iacob, C.E., Necula, I., Wang, C.Y., Noguera, L.A.D., Sanders, C., Wiederhold, N.P. and Sordillo, E.M., 2021. First report of human infection caused by Colletotrichum chlorophyti occurring in a post-corneal transplant patient with endophthalmitis. Medical Mycology Case Reports, 32, pp.73-76.

Pan, W., Liao, W., Hagen, F., Theelen, B., Shi, W., Meis, J.F. and Boekhout, T., 2012. Meningitis caused by Filobasidium uniguttulatum: case report and overview of the literature. Mycoses, 55(2), pp.105-109.

Pan, M., Huang, J., Qiu, Y., Zeng, W., Li, Z., Tang, S., Wei, X. and Zhang, J., 2020, June. Assessment of Talaromyces marneffei infection of the intestine in three patients and a systematic review of case reports. In Open Forum Infectious Diseases (Vol. 7, No. 6, p. ofaa128). US: Oxford University Press.

Pelaez, T., Alvarez-Perez, S., Mellado, E., Serrano, D., Valerio, M., Blanco, J.L., Garcia, M.E., Munoz, P., Cuenca-Estrella, M. and Bouza, E., 2013. Invasive aspergillosis caused by cryptic Aspergillus species: a report of two consecutive episodes in a patient with leukaemia. Journal of medical microbiology, 62(3), pp.474-478.

Parente, J.N.T., Talhari, C., Ginter-Hanselmayer, G., Schettini, A.P.M., da Costa Eiras, J., de Souza, J.V.B., Tavares, R., Buzina, W., Brunasso, A.M.G. and Massone, C., 2011. Subcutaneous phaeohyphomycosis in immunocompetent patients: two new cases caused by Exophiala jeanselmei and Cladophialophora carrionii. Mycoses, 54(3), pp.265-269.

Park, S.G., Oh, S.H., Suh, S.B., Lee, K.H. and Chung, K.Y., 2005. A case of chromoblastomycosis with an unusual clinical manifestation caused by Phialophora verrucosa on an unexposed area: treatment with a combination of amphotericin B and 5-flucytosine. British Journal of Dermatology, 152(3), pp.560-564.

PARK, Y.W., CHOI, B.R., SHIN, J.P. and CHO, H.T., 2006. A Case of Chrysosporium Parvum Endophthalmitis. Journal of the Korean Ophthalmological Society, pp.858-862.

Parsi, K., Itgampalli, R.K., Vittal, R. and Kumar, A., 2013. Perineural spread of rhino-orbitocerebral mucormycosis caused by Apophysomyces elegans. Annals of Indian Academy of Neurology, 16(3), p.414.

Parkes-Smith, J., Bauer, M.J., Bergh, H., Eidan, A., Forde, B.M., Hilton, J., Kidd, T.J., Schmidt, C., Stewart, A.G. and Harris, P.N., 2022. Case report: a fatal case of Aspergillus felis infection in an immunocompetent host. Access Microbiology, 4(11), p.000453.

Parry, M.F., Grant, B., Yukna, M., Adler-Klein, D., McLeod, G.X., Taddonio, R. and Rosenstein, C., 2001. Candida osteomyelitis and diskitis after spinal surgery: an outbreak that implicates artificial nail use. Clinical Infectious Diseases, 32(3), pp.352-357.

Pasquetti, M., Min, A.R.M., Scacchetti, S., Dogliero, A. and Peano, A., 2017. Infection by Microsporum canis in paediatric patients: A veterinary perspective. Veterinary sciences, 4(3), p.46.

Passos, X.S., Sales, W.S., Maciel, P.J., Costa, C.R., Ferreira, D.M. and do Silva, M.R.R., 2006. Nosocomial invasive infection caused by Cunninghamella bertholletiae: case report. Mycopathologia, 161, pp.33-35.

Pastor, F.J. and Guarro, J., 2006. Clinical manifestations, treatment and outcome of Paecilomyces lilacinus infections. Clinical Microbiology and Infection, 12(10), pp.948-960.

Pastorino, A.C., Menezes, U.P.D., Marques, H.H.D.S., Vallada, M.G., Cappellozi, V.L., Carnide, E.M.G. and Jacob, C.M.A., 2005. Acremonium kiliense infection in a child with chronic granulomatous disease. Brazilian Journal of Infectious Diseases, 9, pp.529-534.

Pavlovic, M.D. and Bulajic, N., 2006. Great toenail onychomycosis caused by Syncephalastrum racemosum. Dermatology online journal, 12(1).

Peçanha, P.M., Bahiense, I.C., Kruschewsky, W.L.L., Biasutti, C., Júnior, C.U.G.F., Pinheiro, B.G., Maifrede, S.B., de Camargo, Z.P., Rodrigues, A.M., Grão-Velloso, T.R. and Falqueto, A., 2021. Paracoccidioidomycosis due to Paracoccidioides brasiliensis S1 associated with acquired immunodeficiency syndrome: A case report. Revista Iberoamericana de Micología, 38(1), pp.5-8.

Peltroche-Llacsahuanga, H., Manegold, E., Kroll, G. and Haase, G., 2000. Case report. Pathohistological findings in a clinical case of disseminated infection with Fusarium oxysporum. Mycoses, 43(9-10), pp.367-372.

Pemán, J., Jarque, I., Bosch, M., Cantón, E., Salavert, M., de Llanos, R. and Molina, A., 2006. Spondylodiscitis caused by Candida krusei: case report and susceptibility patterns. Journal of clinical microbiology, 44(5), pp.1912-1914.

Pendones-Ulerio, J., Martins-Lopes, M., García-Garrote, F., Hernández-Calvo, P., Yuste-Chaves, M. and Gutiérrez-Zufiaurre, M.N., 2023. Ringworm by Nannizzia nana: Clinical case and literature review. Enfermedades infecciosas y microbiologia clinica (English ed.).

Perdomo, H., Sutton, D.A., García, D., Fothergill, A.W., Cano, J., Gené, J., Summerbell, R.C., Rinaldi, M.G. and Guarro, J., 2011. Spectrum of clinically relevant Acremonium species in the United States. Journal of Clinical Microbiology, 49(1), pp.243-256.

Pereiro Jr, M., Abalde, M.T., Zulaica, A., Caeiro, J.L., Flórez, A., Peteiro, C. and Toribio, J., 2001. Chronic infection due to Fusarium oxysporum mimicking lupus vulgaris: case report and review of cutaneous involvement in fusariosis. Acta dermato-venereologica, 81(1).

Person, A.K., Chudgar, S.M., Norton, B.L., Tong, B.C. and Stout, J.E., 2010. Aspergillus niger: an unusual cause of invasive pulmonary aspergillosis. Journal of medical microbiology, 59(7), pp.834-838.

Perz M. Fusarium nivale as a cause of corneal mycosis. Klin Oczna 1966; 36: 609–612.

Petrikkos, G., Skiada, A., Sabatakou, H., Antoniadou, A., Dosios, T. and Giamarellou, H., 2001. Case Report. Successful treatment of two cases of post-surgical sternal osteomyelitis, due to Candida krusei and Candida albicans, respectively, with high doses of triazoles (fluconazole, itraconazole). Mycoses, 44(9-10), pp.422-425.

Piepenbring, M., Cáceres Mendez, O.A., Espino Espinoza, A.A., Kirschner, R. and Schöfer, H., 2007. Chromoblastomycosis caused by Chaetomium funicola: a case report from Western Panama. British Journal of Dermatology, 157(5), pp.1025-1029.

Piepenbring, M., Cáceres Mendez, O.A., Espino Espinoza, A.A., Kirschner, R. and Schöfer, H., 2007. Chromoblastomycosis caused by Chaetomium funicola: a case report from Western Panama. *British Journal of Dermatology*, *157*(5), pp.1025-1029.

Pierce, P.F., Wood, M.B., Roberts, G.D., Fitzgerald Jr, R.H., Robertson, C. and Edson, R.S., 1987. Saksenaea vasiformis osteomyelitis. Journal of Clinical Microbiology, 25(5), pp.933-935.

Pillay, T., Pillay, D.G. and Bramdev, A., 1997. Disseminated histoplasmosis in a human immunodeficiency virus-infected African child. The Pediatric infectious disease journal, 16(4), pp.417-418.

Pimentel, J.D., Mahadevan, K., Woodgyer, A., Sigler, L., Gibas, C., Harris, O.C., Lupino, M. and Athan, E., 2005. Peritonitis due to Curvularia inaequalis in an elderly patient undergoing peritoneal dialysis and a review of six cases of peritonitis associated with other Curvularia spp. Journal of clinical microbiology, 43(8), pp.4288-4292.

Pimentel, J.D., Dreyer, G. and Lum, G.D., 2006. Peritonitis due to Cunninghamella bertholletiae in a patient undergoing continuous ambulatory peritoneal dialysis. Journal of medical microbiology, 55(1), pp.115-118.

Pinel, C., Fricker-Hidalgo, H., Lebeau, B., Garban, F., Hamidfar, R., Ambroise-Thomas, P. and Grillot, R., 2003. Detection of circulating Aspergillus fumigatus galactomannan: value and limits of the Platelia test for diagnosing invasive aspergillosis. Journal of clinical microbiology, 41(5), pp.2184-2186.

Pintor, E., Martin, M., Garcia, P. and Gonzalez, M., 2001. Endophthalmitis due to Paecilomyces lilacinus after non-surgical penetrating trauma. Enfermedades infecciosas y microbiologia clinica, 19(7), pp.347-348.

Piquero-Casals, J., Sesto-Casals, D., Savino-Asprino, J.S., Rozas-Muñoz, E., Mir-Bonafé, J.F. and Morgado-Carrasco, D., 2023. Black Piedra in an Amerindian Girl with Oculocutaneous Albinism Type 2. Dermatology Practical & Conceptual, pp.e2023165-e2023165.

Pelaez, T., Alvarez-Perez, S., Mellado, E., Serrano, D., Valerio, M., Blanco, J.L., Garcia, M.E., Munoz, P., Cuenca-Estrella, M. and Bouza, E., 2013. Invasive aspergillosis caused by cryptic Aspergillus species: a report of two consecutive episodes in a patient with leukaemia. Journal of medical microbiology, 62(3), pp.474-478.

Ploysangam, T. and Lucky, A.W., 1997. Childhood white superficial onychomycosis caused by Trichophyton rubrum: report of seven cases and review of the literature. Journal of the American Academy of Dermatology, 36(1), pp.29-32.

Ply, B., Cañete-Gibas, C.F., Sanders, C., Wiederhold, N.P., Dandar, R.A., Sheppard, J.D. and Cheung, A.Y., 2023. Phialophora chinensis fungal keratitis: An initial case report and species identification. American Journal of Ophthalmology Case Reports, 29, p.101800.

Polat, M., Kara, S.S., Tapısız, A., Demirtaş, Z., Sarı, S., Kalkancı, A., Tezer, H. and Dalgıç, B., 2015. Successful treatment of Paecilomyces variotii peritonitis in a liver transplant patient. Mycopathologia, 179(3), pp.317-320.

Polilli, E., Fazii, P., Ursini, T., Fantini, F., Di Masi, F., Tontodonati, M., Sozio, F. and Parruti, G., 2011. Tinea incognito caused by Microsporum gypseum in a patient with advanced HIV infection: a case report. Case Reports in Dermatology, 3(1), pp.55-59.

Porras-López, C., Frías-De-León, M.G., Arenas, R. and Martínez-Herrera, E., 2019. Chromoblastomycosis caused by Rhinocladiella aquaspersa: first case report in Guatemala. Anais Brasileiros de Dermatologia, 94, pp.574-577.

Posteraro, B., Scarano, E., La Sorda, M., Torelli, R., De Corso, E., Mulé, A., Paludetti, G., Fadda, G. and Sanguinetti, M., 2010. Eosinophilic fungal rhinosinusitis due to the unusual pathogen Curvularia inaequalis. Mycoses, 53(1), pp.84-88.

Pote, S.T., Chakraborty, A., Lahiri, K.K., Patole, M.S., Deshmukh, R.A. and Shah, S.R., 2017. Keratitis by a rare pathogen Colletotrichum gloeosporioides: a case report. Journal de Mycologie Médicale, 27(3), pp.407-411.

Pradeepkumar, N.S. and Joseph, N.M., 2011. Chromoblastomycosis caused by Cladophialophora carrionii in a child from India. The Journal of Infection in Developing Countries, 5(07), pp.556-560.

Prasanna, S., Grover, N., Bhatt, P. and Sahni, A.K., 2016. A case of Aspergillus nidulans causing white granule mycetoma. Medical Journal, Armed Forces India, 72(1), p.88.

Premamalini, T., Anitha, S., Rajyoganandh, S.V., Veena, H. and Kindo, A.J., 2019. Complicated urinary tract infection by Trichosporon loubieri. Medical mycology case reports, 24, pp.86-89.

Purohit, G., Sable, M., Rudramurthy, S.M., Sarkar, S., Parida, P., Deshmukh, V. and Hallur, V., 2021. A rare case of condiobolomycosis due to Conidiobolus coronatus presenting with dysphagia. Indian Journal of Medical Microbiology, 39(4), pp.558-560.

Putthirangsiwong, B., Mahaisavariya, P., Chokthaweesak, W. and Selva, D., 2019. Periocular cutaneous mucormycosis caused by saksenaea erythrospora. Journal of Pediatric Infectious Diseases, 14(04), pp.209-212.

Qiangqiang, Z., Limo, Q. and Qixian, Q., 2001. Case report. Disseminated tinea of the verrucous type due to epidermophyton floccosum. Mycoses, 44(7-8), pp.326-329.

Qiu, F., Zhang, C.H., Wang, J.D. and Fan, Y.M., 2022. Scrotal tinea caused by Nannizzia incurvata in two men using molecular identification. Journal of the European Academy of Dermatology and Venereology, 36(11), pp.e957-e959.

Quinio, D., Karam, A., Leroy, J.P., Moal, M.C., Bourbigot, B., Masure, O., Sassolas, B. and Le Flohic, A.M., 2004. Zygomycosis caused by Cunninghamella bertholletiae in a kidney transplant recipient. Medical mycology, 42(2), pp.177-180.

Raad, I. and Hachem, R., 1995. Treatment of central venous catheter-related fungemia due to Fusarium oxysporum. Clinical infectious diseases, 20(3), pp.709-711.

Ragnaud JM, Marcesu C, Roche-Bezian MC, Wone C (1984) Infection pe´ritone´ale a´ Trichoderma koningii sur dialyse sontinue ambulatoire. Med Mal Infect 14:402 – 405.

Rajendran, C., Khaitan, B.K., Mittal, R., Ramam, M., Bhardwaj, M. and Datta, K.K., 2003. Phaeohyphomycosis caused by Exophiala spinifera in India. Medical mycology, 41(5), pp.437-441.

Raju, B., Santhanakumar, K.S. and Kesavachandran, U., 2020. Gastrointestinal involvement of unusual Mucormycete Syncephalastrum racemosum in a diabetic patient with adenocarcinoma: Rare case presentation with review of literature. Infection, 48, pp.791-797.

Ramakrishnan, S., Mandlik, K., Sathe, T.S., Gubert, J., Krishnan, T. and Baskaran, P., 2018. Ocular infections caused by Scedosporium apiospermum: a case series. Indian Journal of Ophthalmology, 66(1), p.137.

Ramakrishnan, S., Mandlik, K., Sathe, T.S., Gubert, J., Krishnan, T. and Baskaran, P., 2018. Ocular infections caused by Scedosporium apiospermum: a case series. Indian Journal of Ophthalmology, 66(1), p.137.

Rameshkumar, G., Sikha, M., Ponlakshmi, M. and Lalitha, P., 2019. A rare case of Myrothecium species causing mycotic keratitis: Diagnosis and management. Medical mycology case reports, 25, pp.53-55.

Ranawaka, R.R., Nagahawatte, A. and Gunasekara, T.A., 2015. Fusarium onychomycosis: prevalence, clinical presentations, response to itraconazole and terbinafine pulse therapy, and 1-year follow-up in nine cases. International Journal of Dermatology, 54(11), pp.1275-1282.

Randhawa, H.S., Khan, Z.U. and Gaur, S.N., 1983. Blastomyces dermatitidis in India: first report of its isolation from clinical material. Sabouraudia: Journal of Medical and Veterinary Mycology, 21(3), pp.215-221.

Ranque, S., Garcia-Hermoso, D., Michel-Nguyen, A. and Dumon, H., 2008. Isolation of Trichoderma atroviride from a liver transplant. Journal de Mycologie Médicale, 18(4), pp.234-236.

Rao, K. and Saha, V., 2000. Medical management of Aspergillus flavus endocarditis. Pediatric hematology and oncology, 17(5), pp.425-427.

Rao, S.S., Panda, N.K., Pragache, G., Chakrabarti, A. and Saravanan, K., 2006. Sinoorbital mucormycosis due to Apophysomyces elegans in immunocompetent individuals—an increasing trend. American journal of otolaryngology, 27(5), pp.366-369.

Rao, A. and Datta, N., 2013. Tinea corporis due to Trichophyton mentagrophytes and Trichophyton tonsurans mimicking tinea imbricata. Indian Journal of Dermatology, Venereology and Leprology, 79(4), p.554.

Rapelanoro, R., Mortureux, P., Couprie, B., Maleville, J. and Taïeb, A., 1996. Neonatal Malassezia furfur pustulosis. Archives of Dermatology, 132(2), pp.190-193.

Rasamoelina, T., Maubon, D., Andrianarison, M., Ranaivo, I., Sendrasoa, F., Rakotozandrindrainy, N., Rakotomalala, F.A., Bailly, S., Rakotonirina, B., Andriantsimahavandy, A. and Rabenja, F.R., 2020. Endemic chromoblastomycosis caused predominantly by Fonsecaea nubica, Madagascar. Emerging Infectious Diseases, 26(6), p.1201.

Rasheeduddin, M. and Visalakshi, P., 2017. Cutaneous phaeohyphomycosis of foot web by Curvularia lunata. Glob. J. Med. Clin. Case Rep, 4, pp.074-075.

Ratcliffe, L., Davies, J., Anson, J., Hales, S., Beeching, N.J. and Beadsworth, M.B.J., 2011. Candida pelliculosa meningitis as an opportunistic infection in HIV: the first reported case. International journal of STD & AIDS, 22(1), pp.54-56.

Ray, R., Ghosh, M., Chatterjee, M., Chatterjee, N. and Banerjee, M., 2016. Case report: onychomycosis caused by Fusarium dimerum. J Clin Sci Res, 5, pp.44-48.

Razouk, S., Sebbani, S., Agoumi, A., Benouchen, T., Malihi, A., Nacir, A., Abouhafsse, A., Al Hamany, Z. and Tligui, H., 2012. The subcutaneous mucormycosis due to Lichtheimia corymbifera: A case report in an immunocompetent child. Journal De Mycologie Medicale, 22(2), pp.185-188.

Read, R.W., Chuck, R.S., Rao, N.A. and Smith, R.E., 2000. Traumatic Acremonium atrogriseum keratitis following laser-assisted in situ keratomileusis. Archives of Ophthalmology, 118(3), pp.418-421.

Reddy, M., Venugopal, R., Prakash, P.Y. and Kamath, Y.S., 2017. Corneal ulcer due to a rare coelomycetes fungus Chaetomium strumarium: Case report and global review of Chaetomium keratomycosis. *Indian journal of ophthalmology*, *65*(9), p.871.

Rezusta, A., Betrán, A., Querol, I., Palacián, M.P. and Revillo, M.J., 2011. Tinea capitis caused by Trichophyton soudanense and Microsporum audouinii in an adult: a case report. Mycoses, 54(1), pp.89-90.

Rhee, D.Y., Kim, M.S., Chang, S.E., Lee, M.W., Choi, J.H., Moon, K.C., Koh, J.K. and Choi, J.S., 2009. A case of tinea manuum caused by Trichophyton mentagrophytes var. erinacei: the first isolation in Korea. Mycoses, 52(3), pp.287-290.

Riat, A., Neofytos, D., Coste, A., Harbarth, S., Bizzini, A., Grandbastien, B., Pugin, J. and Lamoth, F., 2018. First case of Candida auris in Switzerland: discussion about preventive strategies. Swiss medical weekly, 148.

Rickerts, V., Böhme, A., Viertel, A., Behrendt, G., Jacobi, V., Tintelnot, K. and Just-Nübling, G., 2000. Cluster of pulmonary infections caused by Cunninghamella bertholletiae in immunocompromised patients. Clinical Infectious Diseases, 31(4), pp.910-913.

Rimawi, B.H., Rimawi, R.H., Mirdamadi, M., Steed, L.L., Marchell, R., Sutton, D.A., Thompson, E.H., Wiederhold, N.P., Lindner, J.R. and Boger, M.S., 2013. A case of Exophiala oligosperma successfully treated with voriconazole. Medical mycology case reports, 2, pp.144-147.

Rinaldi, S., Fiscarelli, E. and Rizzoni, G., 2000. Paecilomyces variotii peritonitis in an infant on automated peritoneal dialysis. Pediatric nephrology, 14(5), pp.365-366.

Rippon, J.W., Larson, R.A., Rosenthal, D.M. and Clayman, J., 1988. Disseminated cutaneous and peritoneal hyalohyphomycosis caused by Fusarium species: three cases and review of the literature. Mycopathologia, 101(2), pp.105-111.

Ritterband, D.C., Shah, M. and Seedor, J.A., 1997. Colletotrichum graminicola: a new corneal pathogen. Cornea, 16(3), pp.362-364.

Rivero, M., Hidalgo, A., Alastruey-Izquierdo, A., Cía, M., Torroba, L. and Rodríguez-Tudela, J.L., 2009. Infections due to Phialemonium species: case report and review. *Sabouraudia*, *47*(7), pp.766-774.

Rizzitelli, G., Guanziroli, E., Moschin, A., Sangalli, R. and Veraldi, S., 2016. Onychomycosis caused by Trichosporon mucoides. International Journal of Infectious Diseases, 42, pp.61-63.

Robert, T., Talarmin, J.P., Leterrier, M., Cassagnau, E., Pape, P.L., Danner-Boucher, I., Malard, O., Brocard, A., Gay-Andrieu, F., Miegeville, M. and Morio, F., 2012. Phaeohyphomycosis due to Alternaria infectoria: a single-center experience with utility of PCR for diagnosis and species identification. Medical Mycology, 50(6), pp.594-600.

Robertshaw, H. and Higgins, E., 2005. Cutaneous infection with Alternaria tenuissima in an immunocompromised patient. British Journal of Dermatology, 153(5), pp.1047-1049.

Rodriguez-Ares, T., De Rojas Silva, V., Ferreiros, M.P., Becerra, E.P., Tome, C.C. and Sanchez-Salorio, M., 2000. Acremonium keratitis in a patient with herpetic neurotrophic corneal disease. Acta Ophthalmologica Scandinavica, 78(1), pp.107-109.

Rodríguez-Villalobos, H., Georgala, A., Beguin, H., Heymans, C., Pye, G., Crokaert, F. and Aoun, M., 2003. Disseminated infection due to Cylindrocarpon (Fusarium) lichenicola in a neutropenic patient with acute leukaemia: report of a case and review of the literature. European Journal of Clinical Microbiology and Infectious Diseases, 22(1), pp.62-65.

Rodríguez-Gutiérrez, G., Carrillo-Casas, E.M., Arenas, R., García-Méndez, J.O., Toussaint, S., Moreno-Morales, M.E., Schcolnik-Cabrera, A.A., Xicohtencatl-Cortes, J. and Hernández-Castro, R., 2015. Mucormycosis in a non-hodgkin lymphoma patient caused by Syncephalastrum racemosum: case report and review of literature. Mycopathologia, 180, pp.89-93.

Rodríguez, J.Y., Rodríguez, G.J., Morales-López, S.E., Cantillo, C.E., Le Pape, P. and Álvarez-Moreno, C.A., 2016. Saksenaea erythrospora infection after medical tourism for esthetic breast augmentation surgery. International Journal of Infectious Diseases, 49, pp.107-110.

Rodríguez, J.Y., Morales-López, S.E., Rodríguez, G.J., Álvarez-Moreno, C.A., Ocampo, W., Cepeda, M.L. and Mora-Valderrama, M.A., 2018. Necrotizing fasciitis caused by Apophysomyces variabilis in an immunocompetent patient. Medical mycology case reports, 20, pp.4-6.

Roels, D., Coorevits, L. and Lagrou, K., 2020. Tintelnotia destructans as an emerging opportunistic pathogen: First case of T. destructans superinfection in herpetic keratitis. American Journal of Ophthalmology Case Reports, 19, p.100791.

Roilides, E., Sigler, L., Bibashi, E., Katsifa, H., Flaris, N. and Panteliadis, C., 1999. Disseminated infection due to Chrysosporium zonatum in a patient with chronic granulomatous disease and review of non-Aspergillus fungal infections in patients with this disease. Journal of Clinical Microbiology, 37(1), pp.18-25.

Rojas, O.C., González, G.M., Moreno-Treviño, M. and Salas-Alanis, J., 2015. Chromoblastomycosis by Cladophialophora carrionii associated with squamous cell carcinoma and review of published reports. Mycopathologia, 179, pp.153-157.

Roger, P.M., Boissy, C., Gari-Toussaint, M., Foucher, R., Mondain, V., Vandenbos, F., Le Fichoux, Y., Michiels, J. and Dellamonica, P., 2000. Medical treatment of a pacemaker endocarditis due to Candida albicans and to Candida glabrata. Journal of Infection, 41(2), pp.176-178.

Romano, C., Fimiani, M., Pellegrino, M., Valenti, L., Casini, L., Miracco, C. and Faggi, E., 1996. Cutaneous phaeohyphomycosis due to Alternaria tenuissima: Alternaria tenuissima-bedingte kutane Phaeohyphomykose. Mycoses, 39(5-6), pp.211-215.

Romano, C., Valenti, L., Miracco, C., Alessandrini, C., Paccagnini, E., Faggi, E. and Difonzo, E.M., 1997. Two cases of cutaneous phaeohyphomycosis by Alternaria alternata and Alternaria tenuissima. Mycopathologia, 137, pp.65-74.

Romano, C., Miracco, C. and Difonzo, E.M., 1998. Skin and nail infections due to Fusarium oxysporum in Tuscany, Italy: Fusarium oxysporum als Erreger von Haut-und Nagelinfektionen in der Toskana, Italien. Mycoses, 41(9-10), pp.433-437.

Romano, C., De Aloe, G., Calcaterra, R. and Gianni, C., 2002. Tinea capitis due to Trichophyton soudanense and Trichophyton schoenleinii. Mycoses, 45(11-12), pp.518-521.

Romano, C., Rubegni, P., Ghilardi, A. and Fimiani, M., 2006. A case of bullous tinea pedis with dermatophytid reaction caused by Trichophyton violaceum. Mycoses, 49(3), pp.249-250.

Romano, C., Caposciutti, P., Ghilardi, A., Miracco, C. and Fimiani, M., 2010. A case of primary localized cutaneous infection due to Fusarium oxysporum. Mycopathologia, 170(1), pp.39-46.

Romano, C., Bilenchi, R., Alessandrini, C. and Miracco, C., 1999. Case report. Cutaneous phaeohyphomycosis caused by Cladosporium oxysporum. *Mycoses*, *42*(1-2), pp.111-115.

Ross, J.J. and Keeling, D.N., 2000. Cutaneous blastomycosis in New Brunswick: case report. CMAJ, 163(10), pp.1303-1305.

Rota, S., Marchesi, D., Farina, C. and De Bièvre, C., 2000. Trichoderma pseudokoningii peritonitis in automated peritoneal dialysis patient successfully treated by early catheter removal. Peritoneal Dialysis International, 20(1), pp.91-93.

Ruan, Q., Zhu, Y., Chen, S., Zhu, L., Zhang, S. and Zhang, W., 2017. Disseminated cryptococcosis with recurrent multiple abscesses in an immunocompetent patient: a case report and literature review. BMC Infectious Diseases, 17(1), pp.1-6.

Rudramurthy, S.M., Chakrabarti, A., Paul, R.A., Sood, P., Kaur, H., Capoor, M.R., Kindo, A.J., Marak, R.S., Arora, A., Sardana, R. and Das, S., 2017. Candida auris candidaemia in Indian ICUs: analysis of risk factors. Journal of Antimicrobial Chemotherapy, 72(6), pp.1794-1801.

Ruiz, G.B. and Lorenz, A., 2021. What do we know about the biology of the emerging fungal pathogen of humans Candida auris?. *Microbiological Research*, *242*, p.126621.

Ruiz, L.R.B., Zaitz, C., Lellis, R.F. and Veasey, J.V., 2020. Pseudomycetoma of the scalp caused by Microsporum canis. Anais Brasileiros de Dermatologia, 95, pp.372-375.

Ryan, M.E., Kirchner, J.P., Sell, T. and Swanson, M., 1989. Cholangitis due to Blastomyces dermatitidis. Gastroenterology, 96(5), pp.1346-1349.

Ryan, K., Cañete-Gibas, C., Sanders, C. *et al.* *Pseudocanariomyces americanus*, gen. nov., sp. nov., A New Thielavia-Like Species in the Chaetomiaceae: Identification and Management of a Prosthetic Hip Infection. *Mycopathologia* **186**, 441–447 (2021). https://doi.org/10.1007/s11046-021-00555-z

Sadahiro, A., Moraes, J.R.F., Moraes, M.E.H., Romero, M., Gouvea, N.A.D.L., Gouvea, C.J., Ogusku, M.M., Campbell, I. and Zaitz, C., 2004. HLA in Brazilian Ashkenazic Jews with chronic dermatophytosis caused by Trichophyton rubrum. Brazilian journal of microbiology, 35, pp.69-73.

Saeedi, O.J., Iyer, S.A., Mohiuddin, A.Z. and Hogan, R.N., 2013. Exophiala jeanselmei keratitis: case report and review of literature. Eye & Contact Lens, 39(6), pp.410-412.

Saenz, R.E., Brown, W.D. and Sanders, C.V., 2001. Allergic bronchopulmonary disease caused by Bipolaris hawaiiensis presenting as a necrotizing pneumonia: case report and review of literature. The American journal of the medical sciences, 321(3), pp.209-212.

Safneck, J.R., Hogg, G.R. and Napier, L.B., 1990. Endophthalmitis due to Blastomyces dermatitidis: case report and review of the literature. Ophthalmology, 97(2), pp.212-216.

Sageerabanoo, A.M., Oudeacoumar, P. and Udayashankar, C., 2011. Onychomycosis due to Trichosporon mucoides. Indian Journal of Dermatology, Venereology and Leprology, 77, p.76.

Saha, S., Sengupta, J., Banerjee, D., Khetan, A. and Mandal, S.M., 2013. Schizophyllum commune: a new organism in eye infection. Mycopathologia, 175, pp.357-360.

Saiz, P., Gitelis, S., Virkus, W., Piasecki, P., Bengana, C. and Templeton, A., 2004. Blastomycosis of long bones. Clinical Orthopaedics and Related Research®, 421, pp.255-259.

Salem, M.B., Hamouda, M., Mohamed, M., Aloui, S., Letaief, A., Moussa, A., Skhiri, H., Zakahama, A. and Dhia, N.B., 2017, September. Blastomyces dermatitidis in a renal transplant recipient: a case report. In Transplantation Proceedings (Vol. 49, No. 7, pp. 1583-1586). Elsevier.

Salit, R.B., Shea, Y.R., Gea-Banacloche, J., Fahle, G.A., Abu-Asab, M., Sugui, J.A., Carpenter, A.E., Quezado, M.M., Bishop, M.R. and Kwon-Chung, K.J., 2010. Death by edible mushroom: first report of Volvariella volvacea as an etiologic agent of invasive disease in a patient following double umbilical cord blood transplantation. Journal of clinical microbiology, 48(11), pp.4329-4332.

Salle, V., Lecuyer, E., Chouaki, T., Lescure, F.X., Smail, A., Vaidie, A., Dayen, C., Schmit, J.L., Ducroix, J.P. and Douadi, Y., 2005. Paecilomyces variotii fungemia in a patient with multiple myeloma: case report and literature review. Journal of Infection, 51(3), pp.e93-e95.

Samaddar, A., Sharma, A., Maurya, V.K. and Tak, V., 2019. Necrotizing fasciitis caused by Apophysomyces variabilis in a burn patient. IDCases, 18, p.e00660.

Samaddar, A., Shrimali, T., Tiwari, S. and Sharma, A., 2023a. First report of human infection caused by Curvularia warraberensis, manifesting as invasive sinusitis with intracranial involvement. Journal of Medical Mycology, 33(1), p.101337.

Samaddar, A., Shrimali, T. and Sharma, A., 2023b. Subcutaneous mycosis caused by filamentous basidiomycete Megasporoporia setulosa-first report of human infection. Medical Mycology Case Reports, 41, pp.27-32.

Samaddar, A. and Sharma, A., 2023. First case of neonatal fungemia caused by Aureobasidium melanogenum. *Journal of Medical Mycology*, *33*(1), p.101334.

Sandhu, J., Kohli, J.K., Gupta, S.K. and Gupta, V., 2022. Case Report: Plantar Cyst Caused by Phaeoacremonium inflatipes in an Immunocompetent Male, Resolving with Combination Potassium Iodide and Itraconazole Therapy. *The American Journal of Tropical Medicine and Hygiene*, *107*(1), p.113.

Satoh, K., Makimura, K., Hasumi, Y., Nishiyama, Y., Uchida, K. and Yamaguchi, H., 2009. Candida auris sp. nov., a novel ascomycetous yeast isolated from the external ear canal of an inpatient in a Japanese hospital. Microbiology and immunology, 53(1), pp.41-44.

Sautour, M., Chrétien, M.L., Valot, S., Lafon, I., Basmaciyan, L., Legouge, C., Verrier, T., Gonssaud, B., Abou-Hanna, H., Dalle, F. and Caillot, D., 2018. First case of proven invasive pulmonary infection due to Trichoderma longibrachiatum in a neutropenic patient with acute leukemia. Journal de mycologie medicale, 28(4), pp.659-662.

Schlebusch, S. and Looke, D.F., 2005. Intraabdominal zygomycosis caused by Syncephalastrum racemosum infection successfully treated with partial surgical debridement and high-dose amphotericin B lipid complex. Journal of clinical microbiology, 43(11), pp.5825-5827.

Schelenz, S., Hagen, F., Rhodes, J.L., Abdolrasouli, A., Chowdhary, A., Hall, A., Ryan, L., Shackleton, J., Trimlett, R., Meis, J.F. and Armstrong-James, D., 2016. First hospital outbreak of the globally emerging Candida auris in a European hospital. Antimicrobial Resistance & Infection Control, 5(1), pp.1-7.

Schwartz, I.S., Muñoz, J.F., Kenyon, C.R., Govender, N.P., McTaggart, L., Maphanga, T.G., Richardson, S., Becker, P., Cuomo, C.A., McEwen, J.G. and Sigler, L., 2021. Blastomycosis in Africa and the Middle East: a comprehensive review of reported cases and reanalysis of historical isolates based on molecular data. Clinical Infectious Diseases, 73(7), pp.e1560-e1569.

Schwartz, I.S., Wiederhold, N.P., Hanson, K.E., Patterson, T.F. and Sigler, L., 2019. Blastomyces helicus, a new dimorphic fungus causing fatal pulmonary and systemic disease in humans and animals in Western Canada and the United States. Clinical Infectious Diseases, 68(2), pp.188-195.

Schwartz, I.S., Sanche, S., Wiederhold, N.P., Patterson, T.F. and Sigler, L., 2018. Emergomyces canadensis, a dimorphic fungus causing fatal systemic human disease in North America. Emerging Infectious Diseases, 24(4), p.758.

Shigemura, T., Agematsu, K., Yamazaki, T., Eriko, K., Yasuda, G., Nishimura, K. and Koike, K., 2009. Femoral osteomyelitis due to Cladophialophora arxii in a patient with chronic granulomatous disease. Infection, 37(5), pp.469-473.

Schleman, K.A., Tullis, G. and Blum, R., 2000. Intracardiac mass complicating Malassezia furfur fungemia. Chest, 118(6), pp.1828-1829.

Schell, W.A. and Perfect, J.R., 1996. Fatal, disseminated Acremonium strictum infection in a neutropenic host. Journal of clinical microbiology, 34(5), pp.1333-1336.

Schoeppler, K.E., Zamora, M.R., Northcutt, N.M., Barber, G.R., O'Malley-Schroeder, G. and Lyu, D.M., 2015. Invasive Microascus trigonosporus species complex pulmonary infection in a lung transplant recipient. Case Reports in Transplantation, 2015.

Schober, S., Stanchi, K.M.C., Riecker, A., Pfeiffer, M., Tsiflikas, I., Wiegand, G., Quintanilla-Martinez, L., Haen, S., Ebinger, M., Lang, P. and Handgretinger, R., 2021. Fulminant Rhizomucor pusillus mucormycosis during anti-leukemic treatment with blinatumomab in a child: A case report and review of the literature. Medical mycology case reports, 32, pp.4-9.

Scott, R.S., Sutton, D.A. and Jagirdar, J., 2005. Lung infection due to opportunistic fungus, Phialemonium obovatum, in a bone marrow transplant recipient: an emerging infection with fungemia and Crohn disease–like involvement of the gastrointestinal tract. *Annals of Diagnostic Pathology*, *9*(4), pp.227-230.

Scott, I.U., Flynn, H.W., Miller, D., Speights, J.W., Snip, R.C. and Brod, R.D., 2001. Exogenous endophthalmitis caused by amphotericin B–resistant Paecilomyces lilacinus: treatment options and visual outcomes. Archives of Ophthalmology, 119(6), pp.916-919.

Sebbane, I., Lahlimi, F., Tazi, I., Lahrougui, A. and Amal, S., 2022. Cutaneous Fusariosis by a Species of the Fusarium Dimerum in Acute Myeloblastic Leukemia Patient: A Case Report. American Journal of Laboratory Medicine, 7(2), pp.28-31.

Segrelles-Calvo, G., Glauber, R.D.S., Llopis-Pastor, E. and Frasés, S., 2021. Trichosporon asahii as cause of nosocomial pneumonia in patient with COVID-19: a triple co-infection. Archivos de bronconeumologia, 57, p.46.

Seki, A., Yoshida, A., Matsuda, Y., Kawata, M., Nishimura, T., Tanaka, J., Misawa, Y., Nakano, Y., Asami, R., Chida, K. and Kikuchi, K., 2017. Fatal fungal endocarditis by Aspergillus udagawae: an emerging cause of invasive aspergillosis. Cardiovascular Pathology, 28, pp.14-17.

Siddiqui, A.S. and Zimmerman, J.L., 2016. Pulmonary infection secondary to Chrysosporium zonatum in an immunocompetent man. Annals of the American Thoracic Society, 13(5), pp.757-758.

Gamze Sener, A., Yucesoy, M., Senturkun, S., Afsar, I., Gul Yurtsever, S. and Turk, M., 2008. A case of Acremonium strictum peritonitis. Sabouraudia, 46(5), pp.495-497.

Shah, A.V., McColley, S.A., Weil, D. and Zheng, X., 2014. Trichosporon mycotoxinivorans infection in patients with cystic fibrosis. Journal of clinical microbiology, 52(6), pp.2242-2244.

Shah, C.V., Jones, D.B. and Holz, E.R., 2001. Microspaeropsis olivacea keratitis and consecutive endophthalmitis. *American journal of ophthalmology*, *131*(1), pp.142-143.

Shang, S.T., Yang, Y.S. and Peng, M.Y., 2010. Nosocomial Trichosporon asahii fungemia in a patient with secondary hemochromatosis: a rare case report. Journal of Microbiology, Immunology and Infection, 43(1), pp.77-80.

Sharma, R., Farmer, C.K., Gransden, W.R. and Ogg, C.S., 1998. Peritonitis in continuous ambulatory peritoneal dialysis due to Cylindrocarpon lichenicola infection. Nephrology, dialysis, transplantation: official publication of the European Dialysis and Transplant Association-European Renal Association, 13(10), pp.2662-2664.

Sharma, B. and Nonzom, S., 2021. Novel cases of cutaneous phaeohyphomycosis by Alternaria alstromeriae, Epicoccum tritici and Phialemonium obovatum from North India. *Mycoses*, *64*(12), pp.1489-1497.

Sharmin, S., Ohori, A., Sano, A., Kamei, K., Yamaguchi, M., Takeo, K., Uno, J., Nishimura, K. and Miyaji, M., 2003. Histoplasma capsulatum variety duboisii isolated in Japan from an HIV-infected Ugandan patient. Nippon Ishinkin Gakkai Zasshi, 44(4), pp.299-306.

Sharma, N., Batra, H., Mehta, M. and Chander, J., 2014. Maxillary Osteomyelitis Caused by Apophysomyces Variabilis-Emerging Trends. Open Access Macedonian Journal of Medical Sciences, 2(2), pp.303-308.

Sharma, A., Hazarika, N.K., Barua, P., Shivaprakash, M.R. and Chakrabarti, A., 2013. Acremonium strictum: report of a rare emerging agent of cutaneous hyalohyphomycosis with review of literatures. Mycopathologia, 176, pp.435-441.

Sharma, B. and Nonzom, S., 2021. Novel cases of cutaneous phaeohyphomycosis by Alternaria alstromeriae, Epicoccum tritici and Phialemonium obovatum from North India. *Mycoses*, *64*(12), pp.1489-1497.

Shaukat, A., Al Ansari, N., Al Wali, W., Karic, E., El Madhoun, I., Mitwally, H., Hamed, M. and Alutra-Visan, F., 2021. Experience of treating Candida auris cases at a general hospital in the state of Qatar. IDCases, 23, p.e01007.

Shigemura, T., Nakazawa, Y., Amano, Y., Sudo, A., Watanabe, M., Kobayashi, M., Kobayashi, N., Koike, K., Agematsu, K. and Nishimura, K., 2015. Subcutaneous abscess due to the basidiomycete Phellinus mori in a patient with chronic granulomatous disease. Infection, 43, pp.371-375.

Shigeyasu, C., Yamada, M., Nakamura, N., Mizuno, Y., Sato, T. and Yaguchi, T., 2012. Keratomycosis caused by Aspergillus viridinutans: an Aspergillus fumigatus-resembling mold presenting distinct clinical and antifungal susceptibility patterns. Medical Mycology, 50(5), pp.525-528.

Shih, M.H., Sheu, M.M., Chen, H.Y. and Lin, S.R., 1999. Fungal keratitis caused by Candida utilis--case report. The Kaohsiung journal of medical sciences, 15(3), pp.171-174.

Shiraishi, A., Araki-Sasaki, K., Mitani, A., Miyamoto, H., Sunada, A., Ueda, A., Asari, S., Zheng, X., Yamamoto, Y., Hara, Y. and Ohashi, Y., 2011. Clinical characteristics of keratitis due to Colletotrichum gloeosporioides. Journal of Ocular Pharmacology and Therapeutics, 27(5), pp.487-491.

Shivadasan, J., Raksha, K. and Urs, P.S., 2016. Candida utilis causing neonatal Candidemia–A case report and literature review. Apollo Medicine, 13(1), pp.55-58.

Shivaprakash, M.R., Singh, G., Gupta, P., Dhaliwal, M., Kanwar, A.J. and Chakrabarti, A., 2011. Extensive white piedra of the scalp caused by Trichosporon inkin: A case report and review of literature. Mycopathologia, 172, pp.481-486.

Shivaprakash, M.R., Appannanavar, S.B., Dhaliwal, M., Gupta, A., Gupta, S., Gupta, A. and Chakrabarti, A., 2011. Colletotrichum truncatum: an unusual pathogen causing mycotic keratitis and endophthalmitis. Journal of Clinical Microbiology, 49(8), pp.2894-2898.

Shivasabesan, G., Logan, B., Brennan, X., Lau, C., Vaze, A., Bennett, M., Gorrie, N., Mirdad, F., Deveza, R., Koo, C.M. and McCluskey, P., 2022. Disseminated Aspergillus lentulus infection in a heart transplant recipient: a case report. Clinical Infectious Diseases, 75(7), pp.1235-1238.

Sigera, L.S.M., Narangoda, K.U.L., Dahanayake, M.Y., Shabri, U.L.F., Malkanthi, M.A., Jayasekera, P.I. and Kolambage, H.A.L.P., 2020. Mycetoma due to Madurella mycetomatis. IDCases, 21, p.e00857.

Sigler, L., Estrada, S., Montealegre, N.A., Jaramillo, E., Arango, M., De Bedout, C. and Restrepo, A., 1997. Maxillary sinusitis caused by Schizophyllum commune and experience with treatment. Journal of Medical and Veterinary Mycology, 35(5), pp.365-370.

Sigler, L., Bartley, J.R., Parr, D.H. and Morris, A.J., 1999. Maxillary sinusitis caused by medusoid form of Schizophyllum commune. Journal of Clinical Microbiology, 37(10), pp.3395-3398.

Silva, M.E., Malogolowkin, M.H., Hall, T.R., Sadeghi, A.M. and Krogstad, P., 2000. Mycotic aneurysm of the thoracic aorta due to Aspergillus terreus: case report and review. Clinical infectious diseases, 31(5), pp.1144-1148.

Silva, V., Zepeda, G. and Alvareda, D., 2003. Nosocomial urinary infection due to Trichosporon asahii. First two cases in Chile. Revista Iberoamericana de Micologia, 20(1), pp.21-23.

Silva-Vergara, M.L., de Camargo, Z.P., Silva, P.F., Abdalla, M.R., Sgarbieri, R.N., Rodrigues, A.M., dos Santos, K.C., Barata, C.H. and Ferreira-Paim, K., 2012. Case report: disseminated Sporothrix brasiliensis infection with endocardial and ocular involvement in an HIV-infected patient. The American journal of tropical medicine and hygiene, 86(3), p.477.

Simarro, E., Marin, F., Morales, A., Sanz, E., Perez, J. and Ruiz, J., 2001. Fungemia due to Scedosporium prolificans: a description of two cases with fatal outcome. Clinical microbiology and infection, 7(11), pp.645-647.

Singal, A., Pandhi, D., Bhattacharya, S.N., Das, S., Aggarwal, S. and Mishra, K., 2008. Pheohyphomycosis caused by Exophiala spinifera: a rare occurrence. International journal of dermatology, 47(1), pp.44-47.

Singh, S.M., Naidu, J. and Pouranik, M., 1990. Ungual and cutaneous phaeohyphomycosis caused by Alternaria alternata and Alternaria chlamydospora. Journal of Medical and Veterinary Mycology, 28(4), pp.275-278.

Simon, L., Gastaud, L., Martiano, D., Bailleux, C., Hasseine, L. and Gari-Toussaint, M., 2018. First endogenous fungal endophthalmitis due to Fusarium dimerum: A severe eye infection contracted during induction chemotherapy for acute leukemia. Journal de Mycologie Médicale, 28(2), pp.403-406.

Singh, S., Sobel, J.D., Bhargava, P., Boikov, D. and Vazquez, J.A., 2002. Vaginitis due to Candida krusei: epidemiology, clinical aspects, and therapy. Clinical infectious diseases, 35(9), pp.1066-1070.

Slomka, M. and Doub, J., 2020. A rare case of Blastomyces dermatitidis brain abscess in an immunocompetent host. Medical mycology case reports, 28, pp.8-11.

Smriti, C., Anuradha, S., Kamlesh, T., Isampreet, K. and Nitin, K., 2015. Tinea corporis due to Trichophyton violaceum: A report of two cases. Indian Journal of Medical Microbiology, 33(4), pp.596-598.

Soankasina, A.H., Rakotozandrindrainy, N., Andrianteloasy, S., Zafindraibe, N.J., Rasamoelina, T., Rafalimanana, C., Cornet, M., Razanakolona, L.R., Rasamindrakotroka, A. and Andrianarivelo, M.R., 2018. Dermatophyte infection caused by Nannizzia gypsea: a rare case report from Madagascar. Medical mycology case reports, 20, pp.7-9.

Solano, T., Atkins, B., Tambosis, E., Mann, S. and Gottlieb, T., 2000. Disseminated mucormycosis due to Saksenaea vasiformis in an immunocompetent adult. Clinical infectious diseases, 30(6), pp.942-943.

Song, K.Y., Park, C., Byun, J.H., Chun, H.S., Choi, J.H., Han, E.H., Lee, S.O., Jeong, Y., Kim, Y.J. and Kim, S.H., 2020. Fungal arthritis with adjacent osteomyelitis caused by Candida pelliculosa: a case report. Bmc Infectious Diseases, 20(1), pp.1-6.

Song, Y., Liu, X., de Hoog, G.S. and Li, R., 2021. Disseminated cryptococcosis presenting as cellulitis diagnosed by laser capture microdissection: A case report and literature review. Mycopathologia, 186(3), pp.423-433.

Sood, S., Pathak, D., Sharma, R. and Rishi, S., 2006. Case Report-Urinary tract infection by Trichosporon a sahii. Indian Journal of Medical Microbiology, 24(4), pp.294-296.

Souza, B.D.S., Sartori, D.S., Andrade, C.D., Weisheimer, E. and Kiszewski, A.E., 2016. Dermatophytosis caused by Microsporum gypseum in infants: report of four cases and review of the literature. Anais brasileiros de dermatologia, 91, pp.823-825.

Stavropoulou, E., Coste, A.T., Beigelman-Aubry, C., Letovanec, I., Spertini, O., Lovis, A., Krueger, T., Burger, R., Bochud, P.Y. and Lamoth, F., 2020. Conidiobolus pachyzygosporus invasive pulmonary infection in a patient with acute myeloid leukemia: case report and review of the literature. BMC infectious diseases, 20, pp.1-6.

Suankratay, C., Dhissayakamol, O., Uaprasert, N. and Chindamporn, A., 2015. Invasive pulmonary infection caused by Chrysosporium articulatum: the first case report. Mycoses, 58(1), pp.1-3.

Subhashini BSVV, S., Ramana, P.V. and Kamala, P., A Clinical and Diagnostic andApproach of Eumycetoma in the Axilla of a Patient at Tertiary Care Hospital. Volume 18, Issue 7 Ser. 11 (July. 2019)

Subramanian, C. and Sobel, J.D., 2011. A case of Conidiobolus coronatus in the vagina. Medical Mycology, 49(4), pp.427-429.

Sughayer, M., DeGirolami, P.C., Khettry, U., Korzeniowski, D., Grumney, A., Pasarell, L. and McGinnis, M.R., 1991. Human infection caused by Exophiala pisciphila: case report and review. Reviews of infectious diseases, 13(3), pp.379-382.

Suchitra, S.M., Nayak, R.R., Pai, V. and Archana, B.K., 2020. Mycotic keratitis due to Cylindrocarpon lichenicola: Successful salvage of the eye. Indian journal of medical microbiology, 38(3-4), pp.472-474.

Sousa, M.D.G.T.D., Santana, G.B., Criado, P.R. and Benard, G., 2015. Chronic widespread dermatophytosis due to Trichophyton rubrum: a syndrome associated with a Trichophyton-specific functional defect of phagocytes. Frontiers in microbiology, 6, p.801.

Sridhar, M.S., Garg, P., Bansal, A.K. and Gopinathan, U., 2000. Aspergillus flavus keratitis after laser in situ keratomileusis. American journal of ophthalmology, 129(6), pp.802-804.

Steinbach, W.J., Benjamin Jr, D.K., Kontoyiannis, D.P., Perfect, J.R., Lutsar, I., Marr, K.A., Lionakis, M.S., Torres, H.A., Jafri, H. and Walsh, T.J., 2004. Infections due to Aspergillus terreus: a multicenter retrospective analysis of 83 cases. Clinical Infectious Diseases, 39(2), pp.192-198.

Su, H., Li, L., Cheng, B., Zhu, J., Zhang, Q., Xu, J. and Zhu, M., 2017. Trichophyton rubrum infection characterized by Majocchi's granuloma and deeper dermatophytosis: case report and review of published literature. Mycopathologia, 182, pp.549-554.

Su, H., Li, L., Cheng, B., Zhu, J., Zhang, Q., Xu, J. and Zhu, M., 2017. Trichophyton rubrum infection characterized by Majocchi's granuloma and deeper dermatophytosis: case report and review of published literature. Mycopathologia, 182, pp.549-554.

Suchitha, S., Sheeladevi, C.S., Sunila, R. and Manjunath, G.V., 2012. Disseminated cryptococcosis in an immunocompetent patient: a case report. Case Reports in Pathology, 2012.

Sun, P.L., Chi, C.C., Shih, I.H. and Fan, Y.C., 2023. Nannizzia polymorpha as Rare Cause of Skin Dermatophytosis. Emerging Infectious Diseases, 29(7), p.1451.

Surash, S., Tyagi, A., De Hoog, G.S., Zeng, J.S., Barton, R.C. and Hobson, R.P., 2005. Cerebral phaeohyphomycosis caused by Fonsecaea monophora. Medical mycology, 43(5), pp.465-472.

Surpam, R.B., Deshpande, S.D., Saraswathi, K., Amladi, S. and Savant, S., 2006. Trichophyton violaceum: A rare isolate in 18-day-old neonate. Indian J Med Microbiol, 24, pp.292-3.

Su-yang, H.U.A.N.G., Qing-tao, K.O.N.G., Xue, D.U., Rui, Y.A.N.G. and Hong, S.A.N.G., 2016. A case of misdiagnosed tinea faciei caused by Arthroderma vanbreuseghemii. Chinese Journal of Mycology, 11(4), p.219.

Sujatha, S., Sheeladevi, C., Khyriem, A.B., Parija, S.C. and Thappa, D.M., 2003. Subcutaneous zygomycosis caused by Basidiobolus ranarum-a case report. Indian Journal of Medical Microbiology, 21(3), pp.205-206.

Swain B, Panigrahy R, Panigrahi D. Schizophyllum commune sinusitis in an immunocompetent host. Indian Journal of Medical Microbiology. 2011 Oct 1;29(4):439.

Swami, T., Pannu, S., Kumar, M. and Gupta, G., 2016. Chronic invasive fungal rhinosinusitis by Paecilomyces variotii: a rare case report. Indian Journal of Medical Microbiology, 34(1).

Swartz, J. and Stoller, J.K., 2009. Acute eosinophilic pneumonia complicating Coccidioides immitis pneumonia: a case report and literature review. Respiration, 77(1), pp.102-106.

Sweet, D. and Reid, M., 1998. Disseminated neonatal Trichosporon beigelii infection: successful treatment with liposomal amphotericin B. Journal of Infection, 36(1), pp.120-121.

Symoens, F., Knoop, C., Schrooyen, M., Denis, O., Estenne, M., Nolard, N. and Jacobs, F., 2006. Disseminated Scedosporium apiospermum infection in a cystic fibrosis patient after double-lung transplantation. The Journal of heart and lung transplantation, 25(5), pp.603-607.

Tabarsi P, Khalili N, Pourabdollah M, Sharifynia S, Safavi Naeini A, Ghorbani J, Mohamadnia A, Abtahian Z, Askari E. Case Report: COVID-19-associated Rhinosinusitis Mucormycosis Caused by Rhizopus arrhizus: A Rare but Potentially Fatal Infection Occurring After Treatment with Corticosteroids. Am J Trop Med Hyg. 2021 Jul 8;105(2):449-453. doi: 10.4269/ajtmh.21-0359. PMID: 34237015; PMCID: PMC8437195.

Taj-Aldeen, S.J., Hilal, A.A. and Schell, W.A., 2004. Allergic fungal rhinosinusitis: a report of 8 cases. American journal of otolaryngology, 25(3), pp.213-218.

Taj-Aldeen, S.J., Gene, J., Bozom, I.A., Buzina, W., Cano, J.F. and Guarro, J., 2006. Gangrenous necrosis of the diabetic foot caused by Fusarium acutatum. Medical Mycology, 44(6), pp.547-552.

Taj-Aldeen, S.J., Al-Ansari, H.I., Boekhout, T. and Theelen, B., 2004. Co-isolation of Trichosporon inkin and Candida parapsilosis from a scalp white piedra case. Medical mycology, 42(1).

Taguchi, K., Oharaseki, T., Yokouchi, Y., Kawabata, T., Wakayama, M., Ogoshi, T., Iwabuchi, S., Shibuya, K., Nishimura, K. and Takahashi, K., 2007. Allergic fungal sinusitis caused by Bipolaris spicifera and Schizophyllum commune. Medical Mycology, 45(6), pp.559-564.

Tambe, S.A., Dhurat, S.R., Kumar, C.A., Thakare, P., Lade, N., Jerajani, H. and Mathur, M., 2009. Two cases of scalp white piedra caused by Trichosporon ovoides. Indian Journal of Dermatology, Venereology and Leprology, 75, p.293.

Taner Yildiran, S., Mehmet Mutlu, F., Ali Saracli, M., Uysal, Y., Gonlum, A., Sobaci, G. and Sutton, D.A., 2006. Fungal endophthalmitis caused by Aspergillus ustus in a patient following cataract surgery. Medical Mycology, 44(7), pp.665-669.

Tang, P., Mohan, S., Sigler, L., Witterick, I., Summerbell, R., Campbell, I. and Mazzulli, T., 2003. Allergic fungal sinusitis associated with Trichoderma longibrachiatum. Journal of clinical microbiology, 41(11), pp.5333-5336.

Tang, X., Guo, P., Wong, H., Xie, J., Han, J., Xu, Y. and Zhou, H., 2021. Vacuum-assisted closure and skin grafting combined with amphotericin B for successful treatment of an immunocompromised patient with cutaneous mucormycosis caused by Mucor irregularis: a case report and literature review. Mycopathologia, 186(3), pp.449-459.

Tap, R.M., Sabaratnam, P., Ramli, N.Y., Hashim, R., Mohd Fuat, A.R., Ng, P.P., Khairam, H. and Ahmad, N., 2016. Subcutaneous infection associated with Trichosporon ovoides: a case report and review of literature. Mycopathologia, 181, pp.285-290.

Tascini, C., Cardinali, G., Barletta, V., Di Paolo, A., Leonildi, A., Zucchelli, G., Corte, L., Colabella, C., Roscini, L., Consorte, A. and Pasticci, M.B., 2016. First case of Trichoderma longibrachiatum CIED (Cardiac Implantable Electronic Device)-associated endocarditis in a non-immunocompromised host: biofilm removal and diagnostic problems in the light of the current literature. Mycopathologia, 181, pp.297-303.

Tarkkanen, A., Raivio, V., Anttila, V.J., Tommila, P., Ralli, R., Merenmies, L. and Immonen, I., 2004. Fungal endophthalmitis caused by Paecilomyces variotii following cataract surgery: a presumed operating room air-conditioning system contamination. Acta Ophthalmologica Scandinavica, 82(2), pp.232-235.

Tavakoli, M., Hedayati, M.T., Mirhendi, H., Nouripour-Sisakht, S., Hedayati, N., Saghafi, F. and Mamishi, S., 2020. The first rare and fatal case of invasive aspergillosis of spinal cord due to Aspergillus nidulans in an Iranian child with chronic granulomatosis disease: review of literature. Current medical mycology, 6(1), p.55.

Tavanti, A., Davidson, A.D., Gow, N.A., Maiden, M.C. and Odds, F.C., 2005. Candida orthopsilosis and Candida metapsilosis spp. nov. to replace Candida parapsilosis groups II and III. Journal of clinical microbiology, 43(1), pp.284-292.

Tendolkar, U., van Diepeningen, A., Joshi, A., Koomen, J., Bradoo, R., Baveja, S. and Agrawal, S., 2015. Rhinosinusitis caused by Saksenaea erythrospora in an immunocompetent patient in India: a first report. JMM Case Reports, 2(3), p.e000044.

Tendolkar, U., Shinde, A., Baveja, S., Dhurat, R. and Phiske, M., 2014. Trichosporon inkin and Trichosporon mucoides as unusual causes of white piedra of scalp hair. Indian Journal of Dermatology, Venereology and Leprology, 80, p.324.

Thomas, A., Shah, S., Mathews, M. and Chacko, N., 2008. Apophysomyces elegans-renal mucormycosis in a healthy host: a case report from south India. Indian journal of medical microbiology, 26(3), p.269.

Tien JZ, Chou CH, Ho MW, Chen TT. Lethal mushroom: volvariella volvacea infective endocarditis in a patient after allogeneic peripheral blood stem cell transplantation. J Formos Med Assoc. 2020;119(2):664–666.

Tirado-Miranda, R., Solera-Santos, J., Brasero, J.C., Haro-Estarriol, M., Cascales-Sanchez, P. and Igualada, J.B., 2001. Septic arthritis due to Scedosporium apiospermum: case report and review. Journal of Infection, 43(3), pp.210-212.

Todokoro, D., Miyakubo, T., Komori, A., Makimura, K. and Akiyama, H., 2021. A case of contact lens-induced fungal keratitis caused by Didymella heteroderae. *Mycopathologia*, *186*, pp.309-311.

Tomimori-Yamashita, J., Ogawa, M.M., Hirata, S.H., Fischman, O., Michalany, N.S., Yamashita, H.K. and Alchorne, M., 2002. Mycetoma caused by Fusarium solani with osteolytic lesions on the hand: case report. Mycopathologia, 153(1), pp.11-14.

Tong, Z., Chen, S.C.A., Chen, L., Dong, B., Li, R., Hu, Z., Jiang, P., Li, D. and Duan, Y., 2013. Generalized subcutaneous phaeohyphomycosis caused by Phialophora verrucosa: report of a case and review of literature. Mycopathologia, 175, pp.301-306.

Torda, A.J. and Jones, P.D., 1997. Necrotizing cutaneous infection caused by Curvularia brachyspora in an immunocompetent host. Australasian journal of dermatology, 38(2), pp.85-87.

Torres-Guerrero, E., Martínez-Herrera, E., Arroyo-Camarena, S., Porras, C. and Arenas, R., 2015. Kerion Celsi: A report of two cases due to Microsporum gypseum and Trichophyton tonsurans. Our Dermatology Online, 6(4), p.424.

Toyosawa, Y., Kimura, U., Kurihara, M., Noguchi, H., Matsumoto, T., Hiruma, M., Nakazawa, T., Takamori, K. and Suga, Y., 2022. Kerion Celsi caused by nannizzia gypsea in a two-year-old child who had been hospitalized since birth. Medical mycology journal, 63(1), pp.21-23.

Trabelsi, S., Hariga, D. and Khaled, S., 2010. First case of Trichoderma longibrachiatum infection in a renal transplant recipient in Tunisia and review of the literature. La Tunisie Medicale, 88(1), pp.52-57.

Tram QA, Minh NT, Anh DN, Lam NN, Dung TN, Thi Minh Chau N, Tran-Anh L. A rare case of fungal burn wound infection caused by Fusarium solani in Vietnam. Journal of Investigative Medicine High Impact Case Reports. 2020 Mar;8:2324709620912122.

Treguier, P., David, M., Gargala, G., Camus, V., Stamatoullas, A., Menard, A.L., Lenain, P., Contentin, N., Lemasle, É., Lanic, H. and Tilly, H., 2018. Cyberlindnera jadinii (teleomorph Candida utilis) candidaemia in a patient with aplastic anaemia: a case report. JMM Case reports, 5(8).

Trofa, D., Gácser, A. and Nosanchuk, J.D., 2008. Candida parapsilosis, an emerging fungal pathogen. Clinical microbiology reviews, 21(4), pp.606-625.

Trowbridge, J., Ludmer, L.M., Riddle, V.D., Levy, C.S. and Barth, W.F., 1999. Candida lambica polyarthritis in a patient with chronic alcoholism. The Journal of Rheumatology, 26(8), pp.1846-1848.

Tsang, C.C., Chan, K.F., Chan, W., Chan, J.F., Au-Yeung, R.K., Ngan, A.H., Lin, K.P., Lau, S.K. and Woo, P.C., 2021. Hepatic phaeohyphomycosis due to a novel dematiaceous fungus, Pleurostoma hongkongense sp. nov., and importance of antifungal susceptibility testing. Emerging Microbes & Infections, 10(1), pp.81-96.

Torda, A.J. and Jones, P.D., 1997. Necrotizing cutaneous infection caused by Curvularia brachyspora in an immunocompetent host. Australasian journal of dermatology, 38(2), pp.85-87.

Tschopp, J., Perentes, J.Y., Beigelman-Aubry, C., Berezowska, S., Lovis, A., Spertini, O., Bochud, P.Y. and Lamoth, F., 2021. Invasive Hormographiella aspergillata infection in patients with acute myeloid leukemia: Report of two cases successfully treated and review of the literature. Medical Mycology Case Reports, 32, pp.68-72.

Tse, C., Boodman, C. and Wuerz, T., 2022. Trichosporon mucoides prosthetic valve endocarditis managed with antifungal suppression therapy. Medical Mycology Case Reports, 36, pp.10-12.

Uenotsuchi, T., Moroi, Y., Urabe, K., Tsuji, G., Kogas, T., Matsuda, T. and Furue, M., 2005. Cutaneous Scedosporium apiospermum infection in an immunocompromised patient and a review of the literature. Acta dermato-venereologica, 85(2).

Tullio, V., Banche, G., Allizond, V., Roana, J., Mandras, N., Scalas, D., Panzone, M., Cervetti, O., Valle, S., Carlone, N. and Cuffini, A.M., 2010. Non-dermatophyte moulds as skin and nail foot mycosis agents: Phoma herbarum, Chaetomium globosum and Microascus cinereus. *Fungal biology*, *114*(4), pp.345-349.

Uhrlaß, S., Mey, S., Storch, S., Wittig, F., Koch, D., Krüger, C. and Nenoff, P., 2021. Nannizzia incurvata as a rare cause of favus and tinea corporis in Cambodia and Vietnam. Indian Journal of Dermatology, Venereology and Leprology, 87(4), pp.515-521.

Vachharajani, T.J., Zaman, F., Latif, S., Penn, R. and Abreo, K.D., 2005. Curvularia geniculata fungal peritonitis: a case report with review of literature. International urology and nephrology, 37, pp.781-784.

Vagefi, M.R., Kim, E.T., Alvarado, R.G., Duncan, J.L., Howes, E.L. and Crawford, J.B., 2005. Bilateral endogenous Scedosporium prolificans endophthalmitis after lung transplantation. American journal of ophthalmology, 139(2), pp.370-373.

Vagefi, P.A., Cosimi, A.B., Ginns, L.C. and Kotton, C.N., 2008. Cutaneous Aspergillus ustus in a lung transplant recipient: emergence of a new opportunistic fungal pathogen. The Journal of heart and lung transplantation, 27(1), pp.131-134.

Vahidi, S., Beckman, A., Albrecht, K., Arbefeville, S., Ferrieri, P. and Amin, K., 2018. Urinary tract blastomycosis diagnosed by urine cytology. Diagnostic Cytopathology, 46(8), pp.698-701.

Valari, M., Stathi, A., Petropoulou, T., Kakourou, T., Pangali, A. and Arabatzis, M., 2012. Cases of Tinea capitis due to pale isolates of Trichophyton violaceum (Trichophyton glabrum) in South-East Europe. A challenge to the clinical laboratory. Medical mycology case reports, 1(1), pp.66-68.

Vallabhaneni, S., Kallen, A., Tsay, S., Chow, N., Welsh, R., Kerins, J., Kemble, S.K., Pacilli, M., Black, S.R., Landon, E. and Ridgway, J., 2016. Investigation of the first seven reported cases of Candida auris, a globally emerging invasive, multidrug-resistant fungus—United States, May 2013–August 2016. Morbidity and Mortality Weekly Report, 65(44), pp.1234-1237.

Van Etta, L.L., Peterson, L.R. and Gerding, D.N., 1983. Acremonium falciforme (Cephalosporium falciforme): Mycetoma in a renal transplant patient. Archives of dermatology, 119(8), pp.707-708.

Venkateshwar, S., Ambroise, M.M., Asir, G.J., Mudhigeti, N., Ramdas, A., Authy, K., Shivaprakash, M.R. and Kanungo, R., 2014. A rare case report of subcutaneous phaeohyphomycotic cyst caused by Exophiala oligosperma in an immunocompetent host with literature review. Mycopathologia, 178, pp.117-121.

Vásquez-del-Mercado, E., Lammoglia, L. and Arenas, R., 2013. Subcutaneous phaeohyphomycosis due to Curvularia lunata in a renal transplant patient. Revista Iberoamericana de Micología, 30(2), pp.116-118.

Valle, A.C.F.D., Wanke, B., LazÉra, M.D.S., Monteiro, P.C.F. and Viegas, M.D.L., 2001. Entomophthoramycosis by Conidiobolus coronatus. Report of a case successfully treated with the combination of itraconazole and fluconazole. Revista do Instituto de Medicina Tropical de São Paulo, 43, pp.233-236.

Vazquez-Lopez, F., Palacios-Garcia, L. and Argenziano, G., 2011. Dermoscopic corkscrew hairs dissolve after successful therapy of Trichophyton violaceum tinea capitis: A case reportajd_850 118.. 119. Australasian journal of dermatology, 53, pp.118-119.

Vecilla, D.F. and Laredo, C.S., Corneal abscess caused by Filobasidium uniguttulatum. Case report and literature review on cryptococcal keratitis. Revista espanola de quimioterapia: publicacion oficial de la Sociedad Espanola de Quimioterapia, p.fernandez12dec2023.

Vega, W., Orellana, M., Zaror, L., Gené, J. and Guarro, J., 2006. Saksenaea vasiformis infections: case report and literature review. Mycopathologia, 162, pp.289-294.

Velázquez, R., Muñoz-Hernández, B., Arenas, R., Taylor, M.L., Hernández-Hernández, F., Manjarrez, M.E. and López-Martínez, R., 2003. An imported case of Blastomyces dermatitidis infection in Mexico. Mycopathologia, 156(4), pp.263-267.

Valenza, G., Valenza, R., Brederlau, J., Frosch, M. and Kurzai, O., 2006. Identification of Candida fabianii as a cause of lethal septicaemia. Mycoses, 49(4), pp.331-334.

Valeriano, C.A.T., Ferraz, C.E., Oliveira, M.M.E., Inácio, C.P., de Oliveira, E.P., Lacerda, A.M., Neves, R.P. and de Lima-Neto, R.G., 2020. Cat-transmitted disseminated cutaneous sporotrichosis caused by Sporothrix brasiliensis in a new endemic area: Case series in the northeast of Brazil. JAAD Case Reports, 6(10), pp.988-992.

Venditti, M., Micozzi, A., Gentile, G., Polonelli, L., Morace, G., Bianco, P., Avvisati, G., Papa, G. and Martino, P., 1988. Invasive Fusarium solani infections in patients with acute leukemia. Reviews of infectious diseases, 10(3), pp.653-660.

R. Verma, B. Vasudevan, S. Badwal, R. Sriram, S. Neema, V. Kharayat, Rare case of subcutaneous mycosis with intrathoracic extension due to *Chaetomium strumarium*, *Clinical and Experimental Dermatology*, Volume 40, Issue 6, 1 August 2015, Pages 622–625, https://doi.org/10.1111/ced.12604

Vermeulen, E., Maertens, J., Meersseman, P., Saegeman, V., Dupont, L. and Lagrou, K., 2014. Invasive Aspergillus niger complex infections in a Belgian tertiary care hospital. Clinical Microbiology and Infection, 20(5), pp.O333-O335.

Vervaeke, S., Vandamme, K., Boone, E., De Laere, E., Swinne, D. and Surmont, I., 2008. A case of Candida lambica fungemia misidentified as Candida krusei in an intravenous drug abuser. Sabouraudia, 46(8), pp.853-856.

Vieira, M.R., Milheiro, A. and Pacheco, F.A., 2001. Phaeohyphomycosis due to Cladosporium cladosporioides. Sabouraudia, 39(1), pp.135-137.

Vijaya, D., 2001. Keratomycosis due to Fusarium oxysporum--a case report. Indian journal of pathology & microbiology, 44(3), pp.337-338.

Villanueva, D.M., Venkatesan, B. and Figueroa, N., 2022. Cladosporium sphaerospermum as a Rare Cause of Pneumonia. *Cureus*, *14*(6).

Villanueva-Lozano, H., Trevino-Rangel, R.D.J., Renpenning-Carrasco, E.W. and González, G.M., 2017. Successful treatment of Talaromyces amestolkiae pulmonary infection with voriconazole in an acute lymphoblastic leukemia patient. Journal of infection and chemotherapy, 23(6), pp.400-402.

Vinh, D.C., Shea, Y.R., Jones, P.A., Freeman, A.F., Zelazny, A. and Holland, S.M., 2009b. Chronic invasive aspergillosis caused by Aspergillus viridinutans. Emerging infectious diseases, 15(8), p.1292.

Vismer, H.F., Marasas, W.F.O., Rheeder, J.P. and Joubert, J.J., 2002. Fusarium dimerum as a cause of human eye infections. Medical mycology, 40(4), pp.399-406.

Vinh, D.C., Shea, Y.R., Sugui, J.A., Parrilla-Castellar, E.R., Freeman, A.F., Campbell, J.W., Pittaluga, S., Jones, P.A., Zelazny, A., Kleiner, D. and Kwon-Chung, K.J., 2009a. Invasive aspergillosis due to Neosartorya udagawae. Clinical infectious diseases, 49(1), pp.102-111.

Vogelzang, E.H., Weersink, A.J., Van Mansfeld, R., Chow, N.A., Meis, J.F. and Van Dijk, K., 2019. The first two cases of Candida auris in The Netherlands. Journal of Fungi, 5(4), p.91.

Vyas, D.H. and Shah, P.D., 2011. A case of otomycosis caused by Lichtheimia corymbifera (syn. Absidia corymbifera, Mycocladus corymbifer) in a healthy immunocompetent individual. Indian Journal of Otology, 17(1), p.33.

Wagoner, M.D., Badr, I.A. and Hidayat, A.A., 1999. Chrysosporium parvum keratomycosis. Cornea, 18(5), pp.616-620.

Walker, S.D., Clark, R.V., King, C.T., Humphries, J.E., Lytle, L.S. and Butkus, D.E., 1992. Fatal disseminated Conidiobolus coronatus infection in a renal transplant patient. American journal of clinical pathology, 98(6), pp.559-564.

Walker, K., Skelton, H. and Smith, K., 2002. Cutaneous lesions showing giant yeast forms of Blastomyces dermatitidis. Journal of cutaneous pathology, 29(10), pp.616-618.

Walkty, A., Keynan, Y., Karlowsky, J., Dhaliwal, P. and Embil, J., 2018. Central nervous system blastomycosis diagnosed using the MVista® Blastomyces quantitative antigen enzyme immunoassay test on cerebrospinal fluid: a case report and review of the literature. Diagnostic Microbiology and Infectious Disease, 90(2), pp.102-104.

Wankhade, A., SAhu, V., ShArmA, P. and DAS, P., 2021. Keratitis due to a rare fungus Colletotrichum dematium: a case report. J Clin Diag Res, 15, pp.3-5.

Wang, J., Li, L., Xiao, J., Zhu, M. and Zhang, Q., 2003. Subcutaneous Phaeohyphomycosis Caused by Curvularia clavata: The First Case Report in China. Chinese Journal of Dermatology.

Wang, D.L., Nishimura, K. and Miyaji, M., 1991. Taxonomy and identification of Exophiala dermatitidis. Chin J Dermatol, 24, pp.22-23.

Wang, X., Qu, Y., Yang, Z., Zhang, T., Feng, Y., Li, D., Yan, H. and Shi, D., 2023. Surgery plus photodynamic therapy for a diabetic patient with cutaneous infectious granuloma caused by Curvularia lunata. Photodiagnosis and Photodynamic Therapy, 41, p.103253.

Wang, H.P., Yen, Y.F., Chen, W.S., Chou, Y.L., Tsai, C.Y., Chang, H.N. and Chou, C.T., 2007. An unusual case of Candida tropicalis and Candida krusei arthritis in a patient with acute myelogenous leukemia before chemotherapy. Clinical rheumatology, 26(7), pp.1195-1197.

Wang, P., Kenyon, C., de Hoog, S., Guo, L., Fan, H., Liu, H., Li, Z., Sheng, R., Yang, Y., Jiang, Y. and Zhang, L., 2017. A novel dimorphic pathogen, Emergomyces orientalis (Onygenales), agent of disseminated infection. Mycoses, 60(5), pp.310-319.

Watanabe, M., Hayama, K., Fujita, H., Yagoshi, M., Yarita, K., Kamei, K. and Terui, T., 2016. A case of sporotrichosis caused by sporothrix globosa in Japan. Annals of Dermatology, 28(2), pp.251-252.

Weers-Pothoff, G., Havermans, J.F., Kamphuis, J., Sinnige, H.A.M. and Meis, J.F.G.M., 1997. Candida tropicalis arthritis in a patient with acute myeloid leukemia successfully treated with fluconazole: case report and review of the literature. Infection, 25(2), pp.109-111.

Weissgold, D.J., Orlin, S.E., Sulewski, M.E., Frayer, W.C. and Eagle Jr, R.C., 1998. Delayed-onset fungal keratitis after endophthalmitis. Ophthalmology, 105(2), pp.258-262.

Wen, Y.M., Rajendran, R.K., Lin, Y.F., Kirschner, R. and Hu, S., 2016. Onychomycosis associated with Exophiala oligosperma in Taiwan. Mycopathologia, 181, pp.83-88.

Werbel, W.A., Baroncelli, R., Shoham, S. and Zhang, S.X., 2019. Angioinvasive, cutaneous infection due to Colletotrichum siamense in a stem cell transplant recipient: Report and review of prior cases. Transplant Infectious Disease, 21(5), p.e13153.

Wheat, L.J., Connolly-Stringfield, P., Blair, R., Connolly, K., Garringer, T., Katz, B.P. and Gupta, M., 1992. Effect of successful treatment with amphotericin B on Histoplasma capsulatum variety capsulatum polysaccharide antigen levels in patients with AIDS and histoplasmosis. The American journal of medicine, 92(2), pp.153-160.

Wilhelmus, K.R. and Jones, D.B., 2001. Curvularia keratitis. Transactions of the American Ophthalmological Society, 99, p.111.

Williams, P.L., Johnson, R., Pappagianis, D., Einstein, H., Slager, U., Koster, F.T., Eron, J.J., Morrison, J., Aguet, J. and River, M.E., 1992. Vasculitic and encephalitic complications associated with Coccidioides immitis infection of the central nervous system in humans: report of 10 cases and review. Clinical infectious diseases, 14(3), pp.673-682.

Williamson, J.D., Silverman, J.F., Mallak, C.T. and Christie, J.D., 1996. Atypical cytomorphologic appearance of Cryptococcus neoformans: a report of five cases. Acta cytologica, 40(2), pp.363-370.

Williamson, D., Pandey, S., Taylor, S., Rogers, K., Storey, L., Marshall, M.R. and Holland, D., 2011. A case of infection caused by the basidiomycete Phellinus undulatus. Journal of medical microbiology, 60(2), pp.256-258.

Wisuthsarewong, W., Chaiprasert, A. and Viravan, S., 1996. Outbreak of Tinea capitis caused by Microsporum ferrugineum in Thailand. Mycopathologia, 135, pp.157-161.

Wolkow, N., Jakobiec, F.A., Stagner, A.M., Cunnane, M.E., Piantadosi, A.L., Basgoz, N. and Lefebvre, D., 2017. Chronic orbital and calvarial fungal infection with Apophysomyces variabilis in an immunocompetent patient. Survey of Ophthalmology, 62(1), pp.70-82.

Wong, B., Kiehn, T.E., Edwards, F., Bernard, E.M., Marcove, R.C., De Harven, E. and Armstrong, D., 1982. Bone infection caused by Debaryomyces hansenii in a normal host: a case report. *Journal of clinical microbiology*, *16*(3), pp.545-548.

Wongkamhla, T., Chongtrakool, P. and Jitmuang, A., 2019. A case report of Talaromyces marneffei Oro-pharyngo-laryngitis: a rare manifestation of Talaromycosis. BMC Infectious Diseases, 19, pp.1-6.

Woo, P.C., Ngan, A.H., Tsang, C.C., Ling, I.W., Chan, J.F., Leung, S.Y., Yuen, K.Y. and Lau, S.K., 2013. Clinical spectrum of Exophiala infections and a novel Exophiala species, Exophiala hongkongensis. Journal of Clinical Microbiology, 51(1), pp.260-267.

Wu, Z., Ying, H., Yiu, S., Irvine, J. and Smith, R., 2002. Fungal keratitis caused by Scedosporium apiospermum: report of two cases and review of treatment. Cornea, 21(5), pp.519-523.

Wu, P.C., Lai, C.H., Tan, H.Y., Ma, D.H. and Hsiao, C.H., 2010. The successful medical treatment of a case of Paecilomyces lilacinus keratitis. Cornea, 29(3), pp.357-358.

Wu, Y., Wang, J., Li, W., Jia, H., Che, J., Lu, J., Liu, L. and Cheng, Y., 2013. Pichia fabianii blood infection in a premature infant in China: case report. BMC Research Notes, 6(1), pp.1-4.

Wu, Z., Ying, H., Yiu, S., Irvine, J. and Smith, R., 2002. Fungal keratitis caused by Scedosporium apiospermum: report of two cases and review of treatment. Cornea, 21(5), pp.519-523.

Wüppenhorst, N., Lee, M.K., Rappold, E., Kayser, G., Beckervordersandforth, J., de With, K. and Serr, A., 2010. Rhino-orbitocerebral zygomycosis caused by Conidiobolus incongruus in an immunocompromised patient in Germany. Journal of clinical microbiology, 48(11), pp.4322-4325.

Wylen, E.L. and Nanda, A., 1999. Blastomyces dermatitidis occurring as an isolated cerebellar mass. Neurosurgical review, 22(2), pp.152-154.

Wynne, S.M., Kwon-Chung, K.J., Shea, Y.R., Filie, A.C., Varma, A., Lupo, P. and Holland, S.M., 2004. Invasive infection with Trichosporon inkin in 2 siblings with chronic granulomatous disease. Journal of allergy and clinical immunology, 114(6), pp.1418-1424.

Xess, I., N. Jain, F. Hasan, P. Mandal, and U. Banerjee.2007. Epidemiology of candidemia in a tertiary care centre of north India: 5-year study. Infection35:256-259.

Xi, L., Lu, C., Sun, J., Li, X., Liu, H., Zhang, J., Xie, Z. and Hoog, G.D., 2009. Chromoblastomycosis caused by a meristematic mutant of Fonsecaea monophora. Medical Mycology, 47(1), pp.77-80.

Xia, Z.K., Wang, W.L. and Yang, R.Y., 2013. Slowly progressive cutaneous, rhinofacial, and pulmonary mucormycosis caused by Mucor irregularis in an immunocompetent woman. Clinical infectious diseases, 56(7), pp.993-995.

Xia, X.J., Shen, H. and Liu, Z.H., 2015. Primary cutaneous mucormycosis caused by Mucor irregularis. Clinical and Experimental Dermatology, 40(8), pp.875-878.

Xiujiao, X., Hong, S. and Ai-e, X., 2012. Eumycetoma due to Acremonium falciforme acquired in China. Mycoses, 55(2), pp.e4-e7.

Xue, X., Deng, H., Zhao, L., Zang, X., Asuquo, I.P., Meng, M., Ma, X., Qin, C., Meng, Y., Wu, C. and Gao, J., 2020. Cryptococcosis caused by cryptococcus gattii: 2 case reports and literature review. Medicine, 99(50).

Yagi, K., Ushikubo, M., Maeshima, A., Konishi, M., Fujimoto, K., Tsukamoto, M., Araki, K., Kamei, K., Oyamada, Y. and Oshima, H., 2019. Invasive pulmonary aspergillosis due to Aspergillus lentulus in an adult patient: a case report and literature review. Journal of Infection and Chemotherapy, 25(7), pp.547-551.

Yamairi, K., Ido, K., Nakamura, S., Niki, M., Imoto, W., Shibata, W., Namikawa, H., Fujimoto, H., Yamada, K., Nakamae, H. and Hino, M., 2019. Successful treatment of invasive pulmonary aspergillosis caused by Aspergillus felis, a cryptic species within the Aspergillus section Fumigati: a case report. Journal of Infection and Chemotherapy, 25(4), pp.307-310.

Yamaguchi, S., Okubo, Y., Katano, A., Sano, A., Uezato, H. and Takahashi, K., 2015. Primary cutaneous mucormycosis caused by Mucor irregularis in an elderly person. The Journal of Dermatology, 42(2), pp.210-214.

Yamamoto, Y., Osanai, S., Fujiuchi, S., Yamazaki, K., Nakano, H., Ohsaki, Y. and Kikuchi, K., 2002. Extrinsic allergic alveolitis induced by the yeast Debaryomyces hansenii. European Respiratory Journal, 20(5), pp.1351-1353.

Yamamoto, S., Ikeda, M., Ohama, Y. *et al.* *Aureobasidium melanigenum* catheter-related bloodstream infection: a case report. *BMC Infect Dis* **22**, 335 (2022). https://doi.org/10.1186/s12879-022-07310-9

Yasuda-Sekiguchi, F., Kamata, A., Hosokawa, R., Kouno, M., Takahashi, S., Yaguchi, T., Aoyama, K. and Sato, T., 2022. A Case of Kerion Celsi Caused by Trichophyton tonsurans, a Plate Culture of Which Showed Yellow-Green Fluorescence Under UVA Light. Medical Mycology Journal, 63(2), pp.37-41.

Yang, S., Shan, B., Fan, D. *et al.* Tinea Cruris Caused by *Naganishia diffluens*. *Mycopathologia* **188**, 837–839 (2023). https://doi.org/10.1007/s11046-023-00778-2

Yegneswaran, P.P., Pai, V., Bairy, I. and Bhandary, S., 2010. Colletotrichum graminicola keratitis: first case report from India. Indian Journal of Ophthalmology, 58(5), p.415.

Yera, H., Bougnoux, M.E., Jeanrot, C., Baixench, M.T., De Pinieux, G. and Dupouy-Camet, J., 2003. Mycetoma of the foot caused by Fusarium solani: identification of the etiologic agent by DNA sequencing. Journal of clinical microbiology, 41(4), pp.1805-1808.

Yoshida, H., Seki, M., Umeyama, T., Urai, M., Kinjo, Y., Nishi, I., Toyokawa, M., Kaneko, Y., Ohno, H., Miyazaki, Y. and Tomono, K., 2015. Invasive pulmonary aspergillosis due to Aspergillus lentulus: successful treatment of a liver transplant patient. Journal of Infection and Chemotherapy, 21(6), pp.479-481.

You, Z., Yang, X., Yu, J., Zhang, J. and Ran, Y., 2019. Chromoblastomycosis caused by Fonsecaea nubica: first report in northern China and literature review. Mycopathologia, 184, pp.97-105.

Young, E.J., Hirsh, D.D., Fainstein, V. and Williams, T.W., 1980. Pleural effusions due to Cryptococcus neoformans: a review of the literature and report of two cases with cryptococcal antigen determinations. American Review of Respiratory Disease, 121(4), pp.743-747.

Yu, J., Chen, W., Wan, Z. and Li, R.Y., 2004. Adult tinea capitis due to Trichophyton violaceum in China. Mycopathologia, 157(1), pp.49-52.

Yu, S.Y., Guo, L.N., Xiao, M., Zhou, M.L., Yuan, Y., Wang, Y., Zhang, L., Sun, T.S., Ning, Y.T., Jia, P.Y. and Kang, W., 2020. Clinical and microbiological characterization of invasive pulmonary aspergillosis caused by Aspergillus lentulus in China. Frontiers in microbiology, 11, p.1672.

Yun, S.J., Shin, M.G., Choi, C., Kim, H.J., Lee, J.B., Kim, S.J., Lee, S.C. and Won, Y.H., 2007. Fatal disseminated angioinvasive Fusarium falciforme infection in a patient with acute myeloid leukaemia. British Journal of Dermatology, 157(2), pp.407-409.

Yusuf, N.W., Assaf, H.M. and Rotowa, N.A., 2003. Invasive gastrointestinal Basidiobolus ranarum infection in an immunocompetent child. The Pediatric infectious disease journal, 22(3), pp.281-282.

Zahra, L.V., Mallia, D., Hardie, J.G., Bezzina, A. and Fenech, T., 2002. Case report. Keratomycosis due to Alternaria alternata in a diabetic patient. Mycoses, 45(11-12), pp.512-514.

Zarei, F., Mahmoudi, S., Amanizadeh, A. and Afshari, S.A.K., 2023. First report of onychomycosis caused by Phoma glomerata (Didymella glomerata): case report and literature review. Reviews and Research in Medical Microbiology, 34(1), pp.1-5.

Zghair, F.S., 2020. First record of Cyberlindnera fabianii that causes Vulvovaginitis in Iraq. EurAsian Journal of BioSciences, 14(1).

Zhang, J., Xi, L., Lu, C., Li, X., Xie, T., Zhang, H., Xie, Z. and Sybren, D.H., 2009. Successful treatment for chromoblastomycosis caused by Fonsecaea monophora: a report of three cases in Guangdong, China. Mycoses, 52(2), pp.176-181.

Zhang, R., Zhang, J.W. and Szerlip, H.M., 2002. Endocarditis and hemorrhagic stroke caused by Cunninghamella bertholletiae infection after kidney transplantation. American journal of kidney diseases, 40(4), pp.842-846.

Zhang, H., Ran, Y., Liu, Y., Zhang, R., Lin, X., Yan, W. and Dai, Y., 2009. Arthroderma vanbreuseghemii infection in three family members with kerion and tinea corporis. Medical Mycology, 47(5), pp.539-544.

Zhang, Y., Mijiti, J., Huang, C., Song, Y., Wan, Z., Li, R., Kang, X. and Wang, X., 2019. Deep dermatophytosis caused by Microsporum ferrugineum in a patient with CARD9 mutations. British Journal of Dermatology, 181(5), pp.1093-1095.

Zaitz, C., Porto, E., Heins-Vaccari, E.M., Sadahiro, A., Ruiz, L.R.B., Müller, H. and Lacaz, C.D.S., 1995. Subcutaneous hyalohyphomycosis caused by Acremonium recifei: case report. Revista do Instituto de Medicina Tropical de São Paulo, 37, pp.267-270.

Zareei, M., Zibafar, E., Daie Ghazvini, R., Geramishoar, M., Borjian Borujeni, Z., Hossein Pour, L. and Jamal Hashemi, S., 2013. Proximal onychomycosis due to Malassezia furfur: a case report. Tehran University Medical Journal, 70(12).

Zhu Y, Shan Y, Fan S, Li J, Liu X. Candida parapsilosis sensu stricto and the closely related species Candida orthopsilosis and Candida metapsilosis in vulvovaginal candidiasis. Mycopathologia. 2015 Feb;179:111-8.

Zoutman, D.E. and Sigler, L., 1991. Mycetoma of the foot caused by Cylindrocarpon destructans. Journal of clinical Microbiology, 29(9), pp.1855-1859.

Zuo, Q., Dong, L., Mu, W., Zhou, L., Hu, T. and Zhang, H., 2015. Trichosporon asahii infection after total knee arthroplasty: a case report and review of the literature. Canadian Journal of Infectious Diseases and Medical Microbiology, 26, pp.47-51.

Yu, J., Yang, S., Zhao, Y. and Li, R., 2006. A case of subcutaneous phaeohyphomycosis caused by Chaetomium globosum and the sequences analysis of C. globosum. *Medical mycology*, *44*(6), pp.541-545.

Zhang, H., Ran, Y., Li, D. et al. Clavispora lusitaniae and Chaetomium atrobrunneum as Rare Agents of Cutaneous Infection. Mycopathologia 169, 373–380 (2010). https://doi.org/10.1007/s11046-009-9266-9

Zaitz, C., Heins-vaccari, E.M., Freitas, R.S.D., Arriagada, G.L.H., Ruiz, L., Totoli, S.A., Marques, A.C., Rezze, G.G., Muller, H., Valente, N.S. and Lacaz, C.D.S., 1997. Subcutaneous pheohyphomycosis caused by Phoma cava: report of a case and review of the literature. *Revista do Instituto de Medicina Tropical de São Paulo*, *39*, pp.43-48.

Zhang, S., Wang, L., Han, Q. *et al.* Lung cancer coexisting with *Papiliotrema flavescens* infection diagnosed by next-generation sequencing: a case report. *BMC Infect Dis* **22**, 684 (2022). https://doi.org/10.1186/s12879-022-07591-0

Zayit-Soudry, S., Neudorfer, M., Barak, A., Loewenstein, A., Bash, E. and Siegman-Igra, Y., 2005. Endogenous Phialemonium curvatum endophthalmitis. *American journal of ophthalmology*, *140*(4), pp.755-757.

Zhou, Y.B., Chen, P., Sun, T.T., Wang, X.J. and Li, D.M., 2016. Acne-like subcutaneous phaeohyphomycosis caused by Cladosporium cladosporioides: a rare case report and review of published literatures. *Mycopathologia*, *181*, pp.567-573.
